# Homo‐/Heterodimeric Substrates Bias the Type and Multiplicity of Hydroxamic Acid Chelators Assembled by a NIS Synthetase DesD

**DOI:** 10.1002/chem.71115

**Published:** 2026-05-09

**Authors:** Callum A. Rosser, Todd E. Markham, Rachel Codd

**Affiliations:** ^1^ School of Medical Sciences Faculty of Medicine and Health The University of Sydney Sydney New South Wales Australia

**Keywords:** chemoenzymatic synthesis, hydroxamic acid, metal chelators, siderophore synthetase DesD

## Abstract

The siderophore synthetase DesD generates hydroxamic acid chelators with applications in metal‐based radiopharmaceuticals and sequestering toxic or commodity metals. DesD has been used in chemoenzymatic syntheses with native substrates, with less focus on non‐native substrates. This study investigated masking the modest activity of the non‐native substrate *N*‐hydroxy‐*N*‐glutarylcadaverine (**2**) by forming a heterodimer with the native DesD substrate *N*‐hydroxy*‐N*‐succinylcadaverine (**1**). Recombinant DesD from *Salinispora tropica* (*St*DesD) was evaluated with combinations of homo‐ and heterodimers of **1** and **2** (**3–6**) as substrates, including *N*‐to‐*C* positional isomers. Chemoenzymatic reactions using heterodimers of **1** and **2** (**5**, **6**) showed similar substrate consumption and product types to the native **1** homodimer (**3**), with substrate consumption about three times greater than **2** alone, demonstrating the success of the masking approach. Furthermore, dimeric substrates ablated the ability of *St*DesD to generate the odd‐numbered trimeric hexadentate macrocycle desferrioxamine E (DFOE) as its native major product, instead generating the even‐numbered tetrameric macrocycles of dimeric substrates (**3**, **5**, **6**) as major products. While the *St*DesD upper limit of iterations per substrate was similar for monomeric and dimeric substrates, the latter generated chelators with unprecedented cavity sizes and denticities, including an icosadentate chelator that formed a 3:1 metal:ligand complex with Ga(III).

## Graphical abstract

Chemoenzymatic reactions using the siderophore synthetase DesD from *Salinispora tropica* and hydroxamic acid substrates (native, non‐native) with different multiplicities (monomer, dimer) and components (homo‐/heterodimer) followed different reaction trajectories, which biased the architectures of the chelator major products and indicated DesD contains a site that governs the binding of dimeric substrates.

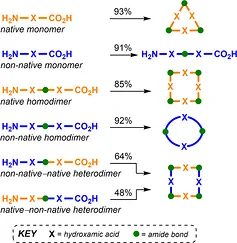

## 1 Introduction

Current metal chelators used in clinical, industrial, and environmental settings occupy a narrow chemical space, which can limit their applications. Exploring wider chemical space [1, 2] could allow the discovery of novel chelators with improved properties, with reservoirs of natural products providing rich search opportunities [3, 4, 5].

Siderophores are a group of natural product chelators produced by bacteria and fungi that have high affinity for Fe(III) (*K*_a_ = 10^25^–10^50^ M^−1^) to allow these organisms to access this poorly bioavailable essential nutrient [6, 7, 8, 9, 10, 11, 12]. Desferrioxamine B (DFOB) is a hydroxamic acid‐containing siderophore used clinically for the treatment of transfusion‐dependent iron overload disease [13, 14]. DFOB is also used as a ^89^Zr(IV) or ^68^Ga(III) chelator in agents being developed for positron emission tomography (PET) imaging of cancer [15, 16, 17, 18, 19, 20, 21], or infection [22, 23, 24, 25]. Exploiting the biosynthetic machinery that produces siderophores such as DFOB offers a potential platform to discover and bioengineer novel metal chelators with diverse functions and applications [26, 27].

**FIGURE 1:**
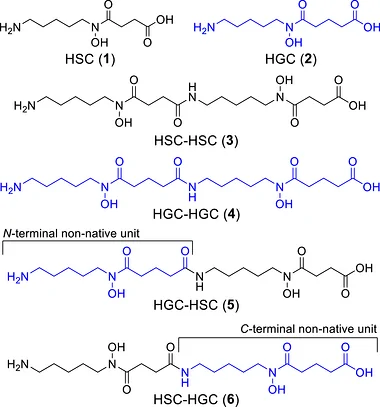
Structures of monomeric substrates HSC (**1**) and HGC (**2**); and dimeric substrates HSC‐HSC (**3**), HGC‐HGC (**4**), HGC‐HSC (**5**), and HSC‐HGC (**6**).

**SCHEME 1:**
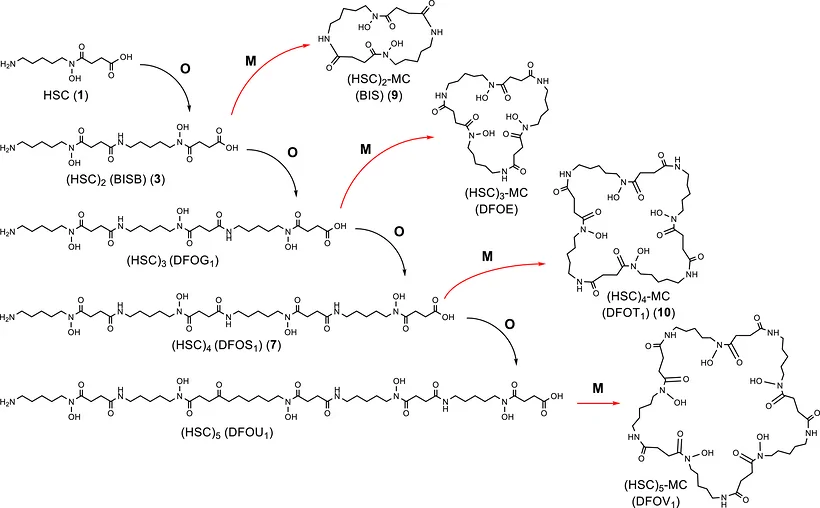
Linear and macrocyclic multimers (O = oligomerization; M = macrocyclization) generated by *St*DesD from chemoenzymatic reactions using **1** [31].

The DesABCD biosynthetic gene cluster is responsible for the biosynthesis of DFOB and other desferrioxamine‐type siderophores [28, 29]. The final enzyme in the cluster is DesD, which is an NRPS‐independent siderophore (NIS) synthetase that catalyzes amide bond formation between *endo*‐hydroxamic acid amino carboxylic acid units to produce multimeric products. DesD catalyzes the formation of oligomeric and end‐to‐end macrocyclic chelators from the native substrate monomer *N*‐hydroxy‐*N*‐succinylcadaverine (HSC) (**1**) (Figure 1), which is produced upstream by DesABC [29, 30, 31]. Recombinant DesD from *Salinispora tropica* CNB‐440 (*St*DesD) processes **1** to form a wide array of linear multimers and the cognate end‐to‐end macrocycles (Scheme 1), which suggests an elasticity in the *St*DesD active site designed to accommodate these higher‐order multimers [31].

DesD and functional analogues operate by activating the carboxylic acid group of one substrate to generate an acyl‐adenylate intermediate primed for nucleophilic attack by the amine group of a second substrate to form an amide bond [29, 30, 31, 32, 33]. DesD has been proposed to have two distinct substrate binding sites, an A‐site (A = adenylation) responsible for binding the substrate that undergoes adenylation, and a C‐site (C = condensation) responsible for positioning the *N*‐terminus of the second substrate as the nucleophile [32]. Reactions of the **1** analogue *N*‐hydroxy‐*N*‐glutarylcadaverine (HGC) (**2**) (Figure 1) with recombinant DesD from *Streptomyces coelicolor* (*Sc*DesD) showed that compared to **1**, the increased length of the alkyl spacer of the di‐acid region reduced the substrate affinity towards the A‐site, thereby limiting adenylation and product formation [32].

This study designed an approach that could increase the capacity of *St*DesD to process substrates with a structurally modified non‐native *C*‐terminus. It was conceived that the *C‐*terminus modification of **2** could be masked by coupling **2** with the native substrate **1** to produce an HGC‐HSC heterodimer (**5**) (*N‐* to *C*‐direction) (Figure 1). The similarity of the *C*‐terminal regions between **1** and **5** could promote the adenylation of **5** by *St*DesD. To evaluate this approach, all combinations of homo‐ and heterodimers of **1** and **2** (Figure 1) were synthesized, and the product profiles of chemoenzymatic reactions with *St*DesD were analyzed by liquid chromatography‐tandem high‐resolution mass spectrometry (LC‐HRMS/MS). The biocombinatorial pools of chelators were subsequently incubated with Ga(III) and Zr(IV) to establish chelator function and evaluate coordination chemistry.

## 2 Results and Discussion

Monomeric (**1**, **2**) and dimeric (**3**–**6**) substrates were synthesized using adapted literature methods (Scheme S2) [34, 35, 36]. Recombinant *St*DesD was prepared using previously established methods (Supporting Information) [31, 37], and enzyme function was assessed by comparing product profiles using the native substrate **1** to those previously established (Figure S1) [31]. Chemoenzymatic reaction solutions contained co‐factors MgCl_2_ (15 mM) and ATP (4 mM), buffer Tris/HCl (145 mM, pH = 8), and equimolar concentrations of substrate (2.4 mM), and were incubated at 37°C for 24 h. Reactions were quenched by adding formic acid (2% *v*/*v*), followed by centrifugation and filtration to remove the denatured enzyme. The reaction solutions were then analyzed by LC‐HRMS/MS. All chemoenzymatic reactions were performed with a matched control containing no *St*DesD.

### 2.1 *St*DesD and the Native Homodimeric Substrate (**3**)

**FIGURE 2:**
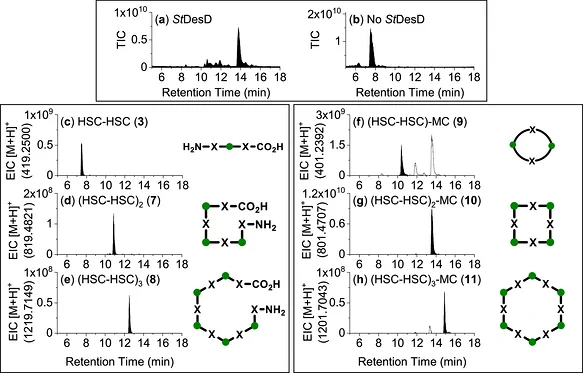
LC‐MS TICs from a reaction solution of **3** with *St*DesD (a) or without *St*DesD (b); and EICs of the [M+H]^+^ adduct of the linear multimers: **3** (c), **7** (d), and **8** (e); and macrocyclic multimers: **9** (f), **10** (g), and **11** (h). The shaded signals were identified as the target multimer *via* the MS^2^ fragmentation pattern (**ESI**, Figures S10 and S11). Multimers are depicted as free ligands with the green circle representing an amide bond and the X representing a hydroxamic acid group.

Initial work evaluated the capacity of *St*DesD to process the native dimeric substrate HSC‐HSC (**3**). LC‐HRMS/MS analysis of the reaction solution containing *St*DesD and **3** identified several signals between 10–16 min in the total ion chromatogram (TIC) that were absent in the enzyme‐free matched control (Figure 2). Further examination of each signal and the corresponding MS/MS fragmentation pattern identified the *St*DesD‐mediated assembly of the linear tetramer (HSC‐HSC)_2_ (**7**) and hexamer (HSC‐HSC)_3_ (**8**). The respective cognate macrocycles (HSC‐HSC)_2_‐MC (**10**, DFOT_1_) and (HSC‐HSC)_3_‐MC (**11**) were also identified, along with the dimeric macrocycle (HSC‐HSC)‐MC (**9**) (Figure 2 and Scheme S2). The relative abundance of each multimer was estimated using area under the curve (AUC) measurements of the extracted ion chromatogram (EIC) signal of a given multimer, noting this assumes the products have similar ionization behavior [38]. This assumption was supported by comparable AUC measurements from a solution containing equimolar **3** and (HSC)_3_‐MC (DFOE) as authentic standards (Figure S7a). The octadentate macrocycle **10** was the most abundant multimer (note the difference in magnitudes of the *y*‐axes in Figure 2c–h), accounting for 85% of the multimers detected (Table S1). During the preparation of **3**, the acid lability of the *C*‐terminal succinic acid formed HSC‐HC (**12**) (Scheme S1). This resulted in the formation of the des‐succinyl species (HSC‐HSC)‐HSC‐HC (**13**), and (HSC‐HSC)_2_‐HSC‐HC (**14**), resulting from the de‐succinylation of **7** and **8**, respectively, and/or the *St*DesD‐mediated elongation of **12** (Figure S5).

**FIGURE 3:**
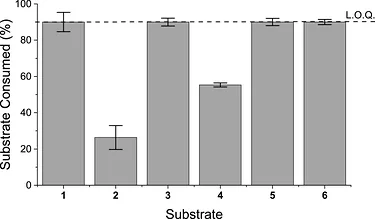
Percentage of substrate (**1–6**) consumed after the 24 h incubation period with *St*DesD determined from a standard curve of the corresponding substrate (Figure S9) (L.O.Q. = Limit of quantitation = 90%).

A standard curve (ESI, Figure S9) was used to quantify substrate consumption as a measure of reaction efficiency. This determined ≥90% (the upper limit of quantification) of the native dimeric substrate **3** was consumed by *St*DesD, which was similar to the consumption of the native monomeric substrate **1** (Figure 3) [36, 39].

### 2.2 *St*DesD and the Non‐Native Monomeric (**2**) and Non‐Native Homodimeric (**4**) Substrates

**FIGURE 4:**
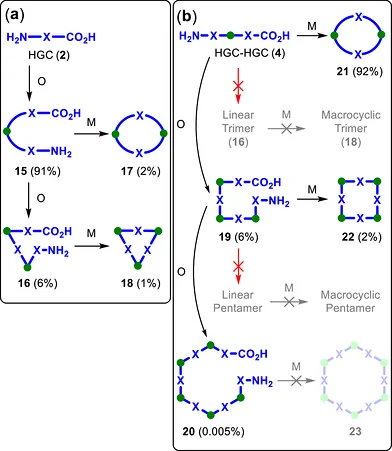
Network of reaction trajectories for **2** (a) and **4** (b); and the percentage of total AUC of each multimer determined by the integral of the respective EIC (O = oligomerization; M = macrocyclization; gray = not formed). Multimers are depicted as free ligands with the green circle representing an amide bond and the X representing a hydroxamic acid group.

The capacity of *St*DesD to process the *C*‐terminal modified monomeric substrate **2** and the corresponding homodimeric substrate **4** was examined under the standard chemoenzymatic reaction conditions. A previous study demonstrated *Sc*DesD had reduced capacity to process **2** [32], with variability in product profiles generated by different DesD homologues warranting examination using *St*DesD [36, 39]. Analysis of the chemoenzymatic reaction solution of *St*DesD, **2**, and co‐factors by LC‐HRMS/MS showed the *St*DesD‐mediated assembly of the linear homodimer (HGC)_2_ (**15**), and trimer (HGC)_3_ (**16**) (Figures 4a and S2). The cognate dimeric (HGC)_2_‐MC (**17**) and trimeric (HGC)_3_‐MC (**18**) macrocycles were also detected (Figures 4a and S2). The product profile generated by *St*DesD was mostly consistent with the reported product profile generated by *Sc*DesD [32], except for **18**, which was detected in the *St*DesD system but not in the *Sc*DesD system. Quantitation of substrate consumption showed 26% of **2** was consumed by *St*DesD, which was significantly less than the native substrate 1 (Figure 3).

Next, the activity of *St*DesD was evaluated using **4**. The reaction solution of *St*DesD and **4** identified the assembly of the linear tetramer (HGC‐HGC)_2_ (**19**) and hexamer (HGC‐HGC)_3_ (**20**) (Figures 4b and S3, and Scheme S3). The dimeric macrocycle (HGC‐HGC)‐MC (**21**) and tetrameric macrocycle (HGC‐HGC)_2_‐MC (**22**) were also detected (Figure 4b; Figure S3, Scheme S3). The cognate macrocycle (HGC‐HGC)_3_‐MC (**23**) of the linear hexamer **20** was not detected. The consumption of substrate **4** was 55% and less than the native substrates **1** and **3** (Figure 3), which aligns with previous work showing the *C*‐terminal glutaric acid region of **2** attenuated adenylation by *Sc*DesD [32].

### 2.3 *St*DesD and the Native–Non‐Native Heterodimeric Substrates (**5** and **6**)

**FIGURE 5:**
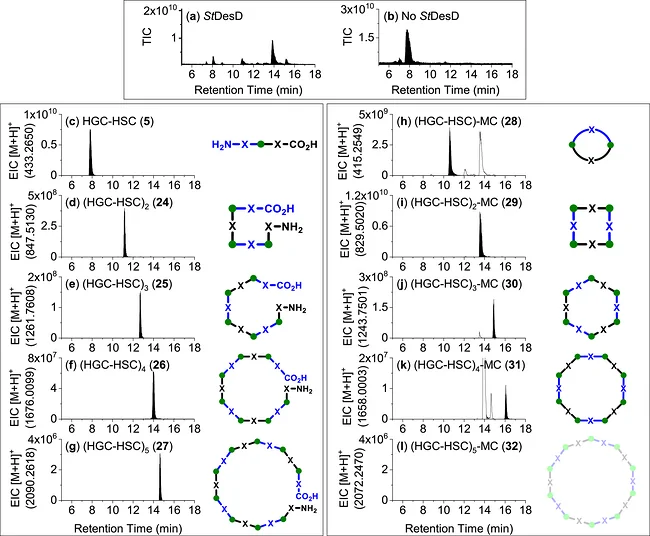
LC‐MS TICs from a reaction solution of **5** with *St*DesD (a) or without *St*DesD (b); and EICs of the [M+H]^+^ adduct of the linear multimers: **5** (c), **24** (d), **25** (e), **26** (f), and **27** (g); and macrocyclic multimers: **28** (h), **29** (i), **30** (j), **31** (k), and **32** (l). The shaded signals were identified as the target multimer via the MS^2^ fragmentation pattern (Figures S15–S18). Multimers are illustrated as free ligands; the green circle represents an amide bond, and the X represents a hydroxamic acid group.

Having established the reduced capacity of *St*DesD to adenylate non‐native monomeric **2** and dimeric **4** substrates compared to the respective native substrates **1** and **3**, methods to increase non‐native substrate adenylation were investigated. An HGC‐HSC heterodimer (**5**) was synthesized, with the aim of masking the non‐native *C*‐terminal modification of **2** and promoting adenylation at the region mimicking the native substrate. Analysis of the reaction solution containing *St*DesD and **5** showed the assembly of the linear tetramer (HGC‐HSC)_2_ (**24**), hexamer (HGC‐HSC)_3_ (**25**), octamer (HGC‐HSC)_4_ (**26**), and decamer (HGC‐HSC)_5_ (**27**) (Figure 5 and Scheme S4). The dimeric macrocycle (HGC‐HSC)‐MC (**28**) and the respective cognate macrocycles of the tetramer (HGC‐HSC)_2_‐MC (**29**), hexamer (HGC‐HSC)_3_‐MC (**30**), and octamer (HGC‐HSC)_4_‐MC (**31**) were also detected (Figure 5 and Scheme S4). The cognate macrocycle (HGC‐HSC)_5_‐MC (**32**) of the linear decamer **27** was not detected. The major product was the tetrameric macrocycle **29** (64%), which was present at significantly greater levels than **28** (27%) and the total abundance of all other products (1.9%) (Table S2). Due to the presence of the *C*‐terminal succinic acid of **5**, des‐succinyl linear chelators were also detected (Figure S6). Quantitation of substrate consumption determined that ≥ 90% of **5** was consumed, similar to reactions using the native dimeric substrate **3**, and significantly greater than **4** (Figure 3).

**FIGURE 6:**
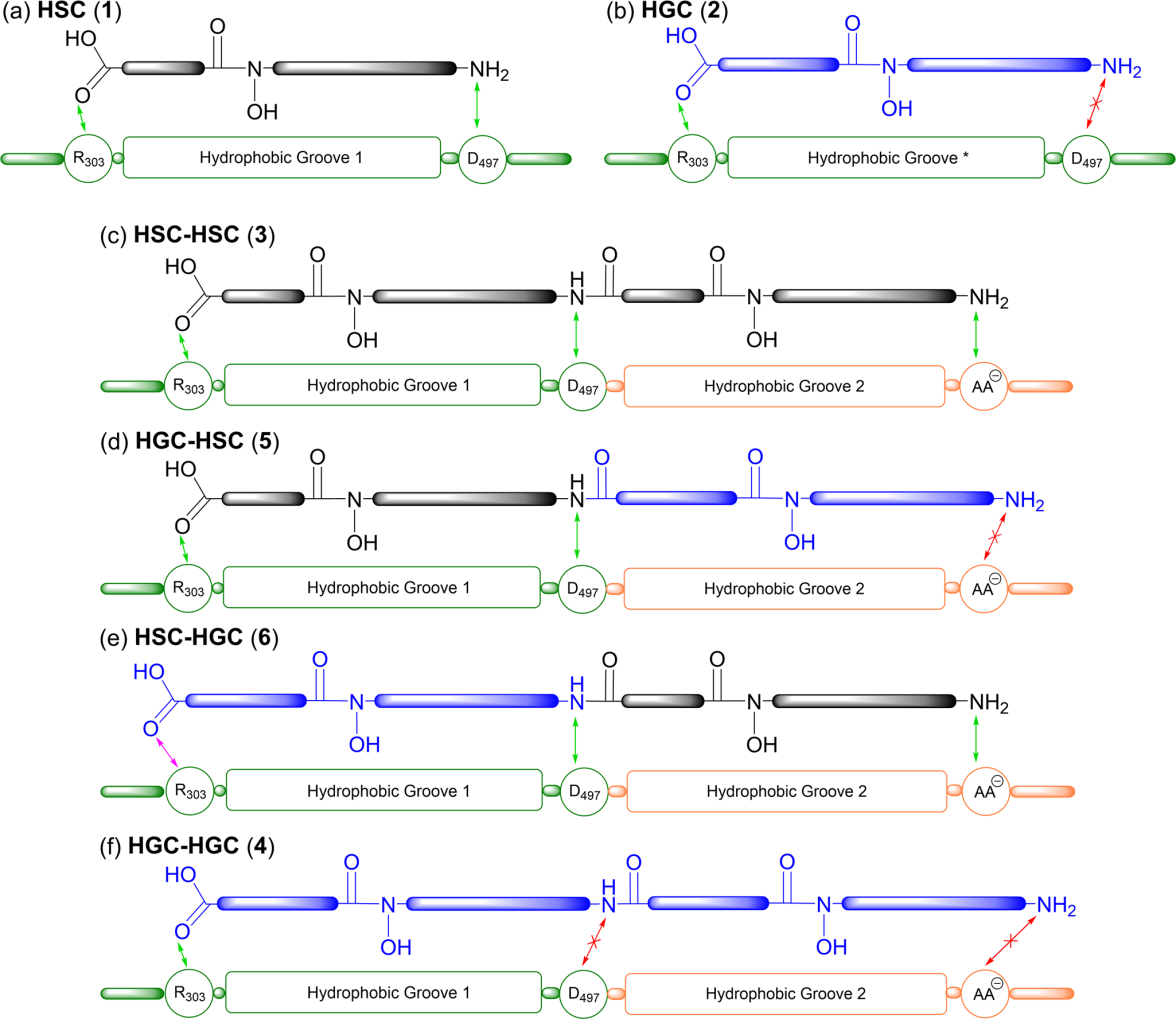
The illustration of putative binding modes of monomeric substrates **1** (a) and **2** (b); and dimeric substrates **3** (b), **5** (c), **6** (d), and **4** (e) at monomeric and dimeric binding sites in the active site of *St*DesD. Amino acid residues shown correspond to the binding site of **1** identified in *Sg*DesD [30]. Green arrows = electrostatic and/or hydrogen bonding interactions; pink arrow = weaker electrostatic and/or hydrogen bonding interactions; red arrows = detrimental interactions.

The observation of a diverse array of linear and macrocyclic multimers formed from **5**, along with the comparable substrate consumption of **5**, **1**, and **3**, suggested *St*DesD could readily adenylate **5**. The improved substrate adenylation of **5**, compared to **2** and **4**, demonstrated the effectiveness of the design intent to increase the A‐site affinity of a non‐native substrate by dimerization with native **1**. An x‐ray crystal structure of recombinant DesD from *Streptomyces griseoflavus* [30] (*Sg*DesD) bound to an inactive adenylated **1** analogue elucidated the substrate binding mode (Figure 6a) as a combination of hydrophobic, hydrogen bonding, and electrostatic interactions with an arginine (*R*_303_) and aspartic acid (*D*_497_) residue (PDB: 7TGM) [30]. From this and the results of the current study, it is proposed that the **1** unit located at the *C*‐terminus of **5** interacts with the A‐site of *St*DesD in a similar manner as **1** (Figure 6d) to enable adenylation.

The alternative heterodimer HSC‐HGC (**6**) was synthesized and evaluated as a *St*DesD substrate to examine any *N*‐ to *C*‐positional dependency of the heterodimeric system. Analysis of the reaction solution by LC‐HRMS/MS showed the assembly of the linear tetramer (HSC‐HGC)_2_ (**33**), hexamer (HSC‐HGC)_3_ (**34**), and octamer (HSC‐HGC)_4_ (**35**) (Figure S4 and Scheme S5). The linear decamer (HSC‐HGC)_5_ (**36**) was identified via a characteristic MS^2^ fragmentation pattern of the peak at 14.8 min, which was one of several signals detected in the EIC at *m*/*z* 2090.2622 (Figure S18). While each pair of same‐order linear multimers generated from **5** or **6** occurs as distinct constitutional isomers (e.g., (HGC‐HSC)_2_ (**24**) and (HSC‐HGC)_2_ (**33**)), these form a unique end‐to‐end macrocyclic product (e.g., (HGC‐HSC)‐MC (**28**)≡(HSC‐HGC)‐MC (**28**)). In addition to the set of linear multimers, the macrocyclic dimer (HSC‐HGC)‐MC (**28**), tetramer (HSC‐HGC)_2_‐MC (**29**), and hexamer (HSC‐HGC)_3_‐MC (**30**) were identified (Figure S4 and Scheme S5). The cognate macrocycles **31** and **32**, generated from **35** or **36**, respectively, were not detected.

The level of substrate consumption and the trends in the product profile from **6** were similar with **5** (Figure 3, and Tables S2 and S3), aside from (HSC‐HGC)_4_‐MC (**31**), which was not detected. These results suggested that **6** had a similar affinity to **5** for the A‐site, which was unexpected since experiments using the monomer **2** showed significant attenuation of adenylation due to the *C*‐terminal glutaric acid unit. This prompted consideration of the subtleties of the *St*DesD binding pocket that could be specific to processing dimeric substrates.

### 2.4 An Extended Binding Site in *St*DesD for Dimeric Substrates

It could be that *St*DesD has a region within the substrate binding pocket, named here as the dimeric substrate binding site, that interacts with the *N‐*terminal region of the **1** unit present in **3** and **6**, which stabilizes the overall positioning of the substrate, including the region in the distal A site to increase adenylation (Figure 6c,e). Interactions maintaining the increased reactivity of **6** (Figure 6e) compared with **2** (Figure 6b) could include an electrostatic interaction between the terminal amine group of **6** with an acidic amino acid residue AA^−^ and/or other hydrophobic or hydrogen bonding interactions. Given that *St*DesD processed **5** and **6** to similar levels, it could be that any binding penalties incurred by the molecular overhang at the *N*‐ (**5**) or *C‐*termini (**6**), respectively (Figure 6d,e), are offset by stabilizing effects from interactions between the internal amide group in the substrate to D_497_ and/or hydrophobic interactions involving the methylene groups. In contrast, the processing of **4** could be compromised by binding penalties induced by the two non‐native di‐acid units, causing length distortions at both the *N*‐ and *C*‐termini (Figure 6f) in addition to a reduction in the stabilizing effects of the internal amide to *D*_497_ interaction due to its offset. This analysis of monomeric and dimeric substrate binding to *St*DesD aligns with earlier work on monomeric substrates, which showed the influence of the spacing patterns of the polar binding groups on substrate competency [32]. We are progressing with x‐ray crystal structure analyses of *St*DesD, with these data coupled with *in silico* modelling, enabling further evaluation of the dimeric substrate binding site.

### 2.5 Dimeric Substrates Bias the *St*DesD‐Mediated Production of Octadentate Chelators

**FIGURE 7:**
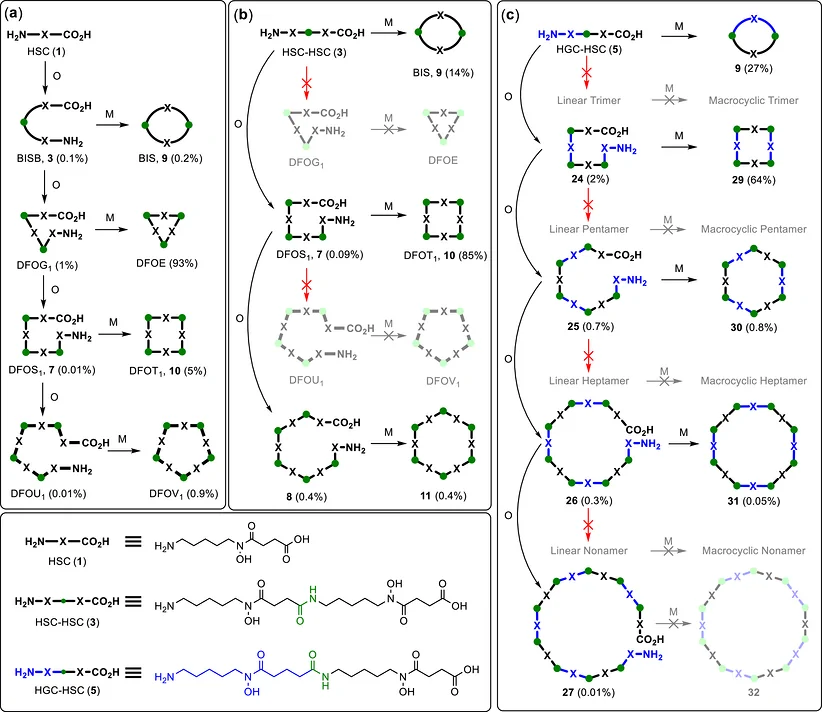
Network of reaction trajectories for **1** (a), **3** (b), and **5** (c); and the percentage of total AUC of each multimer determined by the integral of the respective EIC (O = oligomerization; M = macrocyclization; gray = not formed).

The use of dimeric substrates prevented the capacity of *St*DesD to form odd‐numbered multimers, such as its major biosynthetic product (HSC)_3_‐MC (DFOE), formed from (HSC)_3_ (DFOG_1_) (Scheme 1). This biased the substrate pool to undergo elongation to form even‐numbered multimers (Figure 7), noting low production levels of the dimeric macrocycle **9** or **9** analogues due to steric strain [32, 38]. This directed the *St*DesD‐mediated generation of the tetrameric octadentate macrocycle **10** from **3**, and **29** from **5** or **6** (with the directionality in **5** and **6** lost in the unique macrocycle **29**) as the major products. Macrocycles **10** and **29** possess cavity sizes and denticities well suited to complex metal ions with larger ionic radii, such as Zr(IV) [40, 41]. Reported yields of **10** using total or metal‐templated synthesis ranged between 7%–11% [40, 42]. The 50%–85% yields of **10** and analogues generated from the *St*DesD system (Tables S1–S3) highlight the potential to use an in‐parallel chemoenzymatic experimental set‐up as an approach to access these chelators.

### 2.6 Formation of High Cavity Volume‐High Denticity Chelators

*St*DesD generated hexameric dodecadentate multimers from **3**, **5**, and **6**, as well as octameric hexadecadentate and decameric icosadentate multimers from **5** and **6**, albeit in low abundance (Figure 7). The relative abundance of linear and macrocyclic multimers showed that as multimers were extended, they became less competent substrates for macrocyclization (Figure S7b). The reduced macrocyclization rate of hexameric, octameric, and decameric multimers could be due to higher‐order multimers exceeding the volume of the *St*DesD active site, resulting in a portion of the multimeric substrate external to the active site as necessary to position the *C*‐ and *N*‐termini at the A‐ and C‐ sites, respectively. It would be expected that an increase in multimer length would increase the degrees of freedom and, coupled with substrate concentration effects, reduce the abundance of the cognate end‐to‐end macrocycle. There was a significant difference between the cavity volume and denticities of the upper limit oligomeric and macrocyclic products formed using *St*DesD with monomeric **1** and dimeric **3**–**6** substrates. In the case of **1**, the highest order multimer observed was a pentameric decadentate chelator [31]. In the case of **3**–**6**, while the highest‐order multimer observed was also a pentamer, the resulting product was an icosadentate chelator by virtue of its assembly from a dimeric starting substrate. This suggests differences in the chelator product (volume, denticity) can be directed by the multiplicity of the feed substrate.

### 2.7 Screening the Coordination Chemistry of the Chelator Products

The coordination chemistry of the chelator pools was examined using Ga(III) and Zr(IV) as surrogates of the medically relevant radiometal isotopes ^68^Ga(III) and ^89^Zr(IV). The biocombinatorial mixture of chelators generated using **5** (products: **24**–**32**) was incubated for 2 h with Ga(III)(NO_3_)_3_ in a 3:1 ratio, or Zr(IV)Cl_4_ in a 5:1 ratio. The metal:chelator stoichiometry was designed to generate saturated coordination complexes for analysis by LC‐HRMS [31].

**FIGURE 8:**
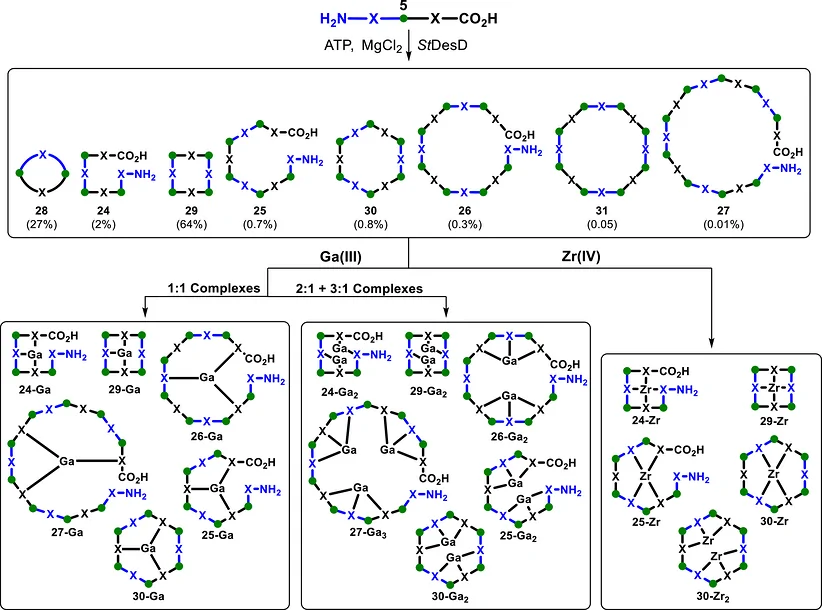
Biocombinatorial mixture of high‐denticity chelators generated by *St*DesD from **5** (**24–31**) illustrated as free ligands and as coordination complexes characterised in situ after incubation with Ga(III) or Zr(IV). The green circle represents an amide bond, the X represents a hydroxamic acid group, with the single X–metal bond representing bidentate chelation, noting that coordination isomers cannot be delineated.

**FIGURE 9:**
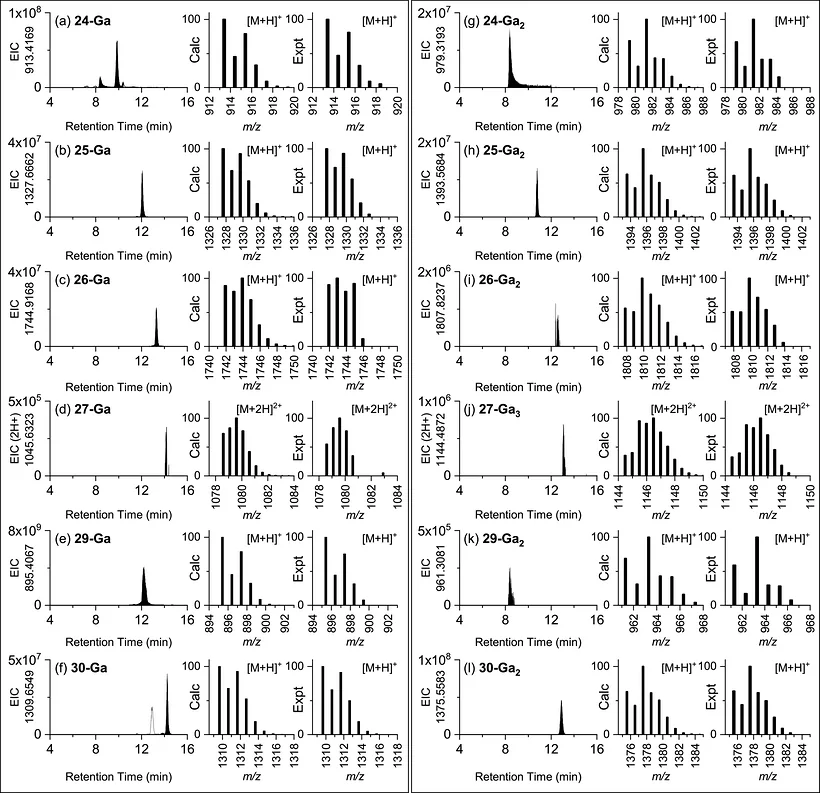
EICs from a biocombinatorial mixture of high denticity chelators generated by *St*DesD from **5** incubated with an excess of Ga(III) with EIC values set to the [M+H]^+^ adduct (a–c, e–f) or [M+2H]^2+^ adduct (d) of 1:1 metal:ligand complexes, [M+H]^+^ adduct of 2:1 metal:ligand complexes (g–i, k–l), or the [M+2H]^2+^ adduct of 3:1 metal:ligand complex (j) with Ga(III) and **24**–**30**, and isotope patterns (left: calculated; right: experimental) extracted from shaded peak.

Analysis of the mixture of Ga(III)(NO_3_)_3_ and chelators generated from **5** by *St*DesD showed the formation of mono‐, bi‐, and tri‐metallic Ga(III) complexes (Figure 8). Signals for the 1:1 metal:ligand complexes **4**‐Ga, **24**‐Ga, **25**‐Ga, **26**‐Ga, **27**‐Ga, **29**‐Ga, and **30**‐Ga were identified by a 65.9031 *m/z* shift in the mass spectrum, shifts in the signals in the chromatograms compared to the matched free chelators, and characteristic Ga(III) isotope patterns (Figure 9). Chelators with increased cavity size and denticities enabled the formation of multi‐metallic complexes. Signals for the 2:1 metal:ligand complexes **24**‐Ga_2_, **25**‐Ga_2_, **26**‐Ga_2_, **29**‐Ga_2_, and **30**‐Ga_2_ were identified, with the icosadentate chelator **27** forming a 3:1 metal:ligand complex **27**‐Ga_3_ (Figure 9).

**FIGURE 10:**
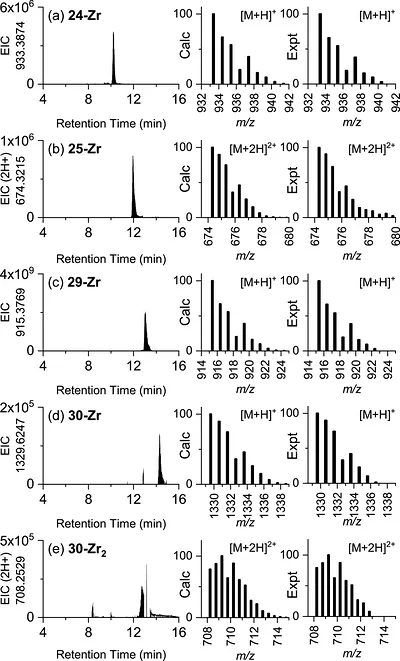
EICs from a biocombinatorial mixture of high denticity chelators generated by *St*DesD from **5** incubated with an excess of Zr(IV) with EIC values set to the [M+H]^+^ adduct (a, c–d) or [M+2H]^2+^ adduct (b) of 1:1 metal:ligand complexes or the [M+2H]^2+^ adduct of 2:1 metal:ligand complexes (e) with Zr(IV) and **24**–**30**, and isotope patterns (left: calculated; right: experimental) extracted from the shaded peak.

Analysis of the mixture of Zr(IV)Cl_4_ and chelators showed the formation of mono‐ and bi‐metallic Zr(IV) complexes (Figure 8). Complexes identified with a 1:1 metal:ligand stoichiometry included **24**‐Zr, **25**‐Zr, **29**‐Zr, and **30**‐Zr, with the 2:1 metal:ligand complex **30**‐Zr_2_ formed with the hexameric dodecadentate macrocycle **30** (Figure 10).

## 3 Conclusions

This study evaluated the activity of the recombinant NIS synthetase from *S. tropica* CNB‐440 (*St*DesD) with dimeric substrates. *St*DesD retained substrate competency in processing the native dimeric substrate **3** similar with the native monomeric substrate **1**. The reduced substrate competency of **2** was reversed by forming heterodimeric substrates of **1** and **2**, with **2** positioned at the *N*‐ or *C*‐termini giving **5** or **6**, respectively. The product profile suggested the presence of a *St*DesD binding site configured to bind the *N‐*terminal **1** unit of a dimeric substrate to increase the affinity of the *C*‐terminal region of **5** or **6** for adenylation. A reduction in the binding interaction between the internal amide group in the dimeric substrate and the partner acidic amino acid residue, coupled with the additional molecular overhang from the *C*‐ and *N*‐terminal regions in **4** attenuating binding interactions, could contribute to its lowest ranking among the dimeric substrates **3**–**6**.

Using dimeric substrates in this chemoenzymatic reaction system prevented the ability of *St*DesD to form odd‐numbered multimers. This ablated the ability to form trimeric macrocyclic hydroxamic acids, such as DFOE, and instead biased the formation of the tetrameric hydroxamic acid DFOT_1_ (**10**). The coordination chemistries of the chelator mixtures were evaluated in the presence of Ga(III) or Zr(IV). Octadentate (**24**, **29**), dodecadentate (**25**, **30**), hexadecadentate (**26**, **31**), and icosadentate (**27**) ligands formed 1:1 or 2:1 metal:ligand complexes with Ga(III), with **27** forming a 3:1 metal:ligand complex, as new coordination chemistry. Octadentate (**24**, **29**) and dodecadentate (**25**, **30**) chelators formed 1:1 metal:ligand complexes with Zr(IV), with **30** forming a 2:1 metal:ligand complex. The use of dimeric substrates **3**–**6** enabled the *St*DesD‐mediated production of high cavity volume‐high denticity chelators. This work demonstrates the utility of *St*DesD as a biocatalyst for the chemoenzymatic exploration of chemical space and provides a platform to identify novel chelators with useful functions.

## Conflicts of Interest

The authors declare no conflicts of interest.

## Supporting information




**Supporting File**: The authors have cited additional references within the Supporting Information [29, 31, 32, 35, 36, 37, 43].

## Data Availability

The data that support the findings of this study are available in the Supporting Information of this article.
