# Supplementary material for: Homo‐/Heterodimeric Substrates Bias the Type and Multiplicity of Hydroxamic Acid Chelators Assembled by a NIS Synthetase DesD
Source: Chemistry. 2026 May 9;32(32):e71115. doi: 10.1002/chem.71115 (PMC13505702; doi:10.1002/chem.71115)
Supplement: Supplementary file 1 [file CHEM-32-e71115-s001.pdf]

Supporting Information

Homo-/Heterodimeric Substrates Bias the Type and Multiplicity of Hydroxamic Acid Chelators Assembled by a NIS Synthetase DesD

Callum A. Rosser[a], Todd E. Markham[a], Rachel Codd*[a]

[a] Dr. C.A. Rosser; Dr. T.E. Markham; Prof. R. Codd.
School of Medical Sciences, Faculty of Medicine and Health
The University of Sydney
Darlington, Sydney, NSW, 2008
Email: rachel.codd@sydney.edu.au

Table of Contents

Item	Page
**LC-HRMS/MS Analysis of Chemoenzymatic Reactions of 1, 2, 4, and 6**…………..………………………………	**S2**
**De-succinylation of 3 and 5 Multimers in Chemoenzymatic Reactions**……….……….……………………....	**S4**
**Structures of Linear and Macrocyclic Multimers in Chemoenzymatic Reactions of 3–6**……………………....	**S6**
**Relative Abundance of Multimers Detected in Chemoenzymatic Reactions of 1–6**………...………….……..…	**S9**
**Relative Abundance of Linear and Macrocyclic Multimers in Chemoenzymatic Reaction of 5**………………	**S11**
**Structures of Ga(III) and Zr(IV) Complexes identified**……………………...………………………………………	**S12**
**Substrate Standard Curves**……………………………………………………………………………….……………	**S13**
**Fragmentation Patterns of Multimers Assembled by *St*DesD**………………...………………………...…………...	**S14**
**Materials**………………………………………………………………………………………….………...…………………	**S25**
**General Methodology**……………………………………………………………………………………...………………..	**S25**
**Recombinant Expression and Purification of *St*DesD**………………………………………………...………………	**S26**
**Chemoenzymatic Reactions**………………………………………………………………….………………………….…	**S26**
**SDS-PAGE Gel of Purified *St*DesD**…………………………………………………………………………………........	**S27**
**Synthetic Procedures**…………………………………………………………………………………………………….…	**S28**
**Characterisation Data of Synthesised Compounds**…………………………………………………………………..…	**S37**
**References**………....	**S66**

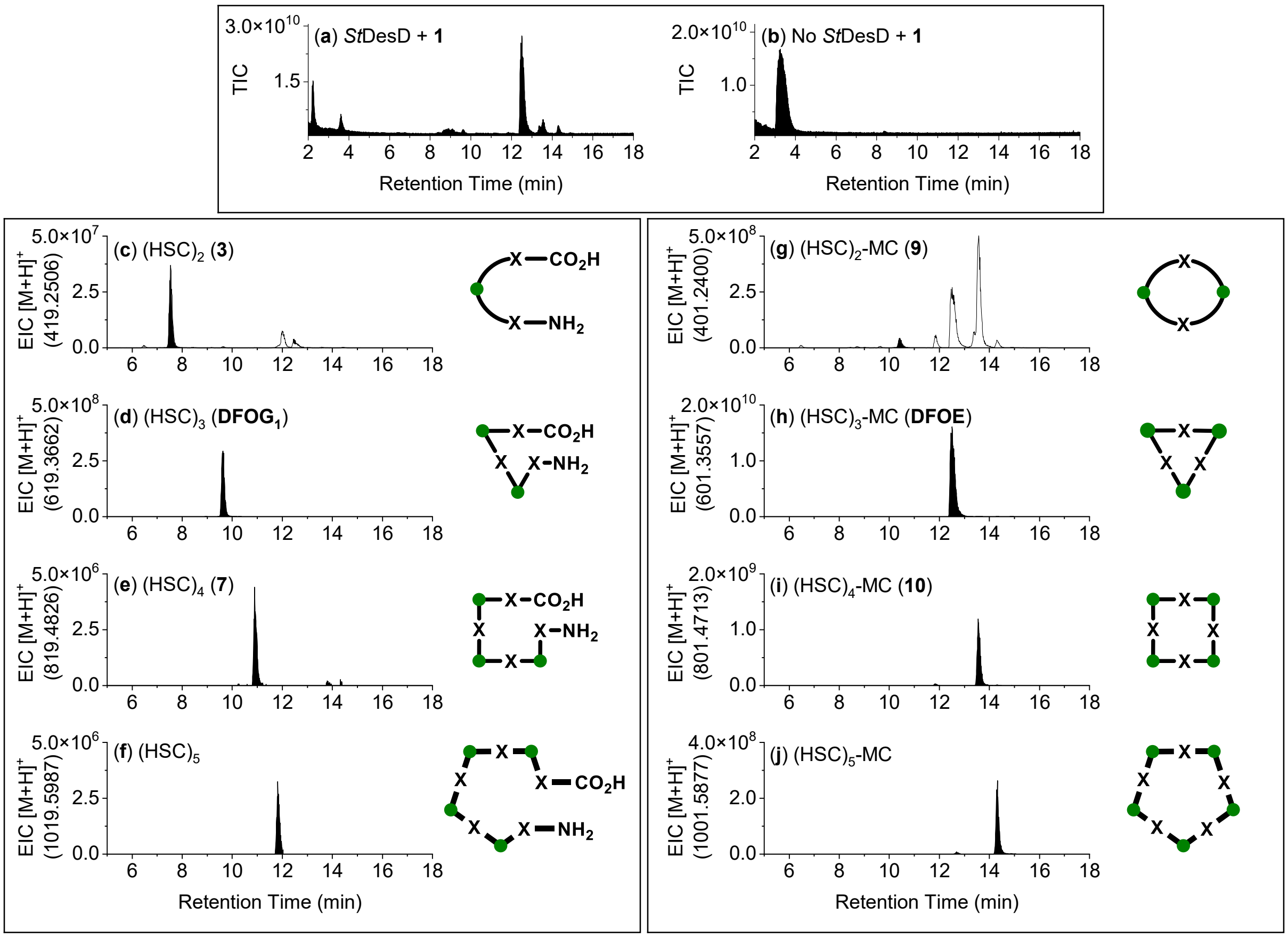

Figure S1. LC-MS TIC from a reaction solution of **1** with *St*DesD (a) or without *St*DesD (b); EIC of the $[M+H]^+$ adduct of the linear multimers: **3** (c), **DFOG$_1$** (d), **7** (e) and **(HSC)$_5$** (f); and macrocyclic multimers: **9** (g), **DFOE** (h), **10** (i), and **(HSC)$_5$-MC** (j). Shaded signal identified as multimer *via* the MS^2 fragmentation pattern.

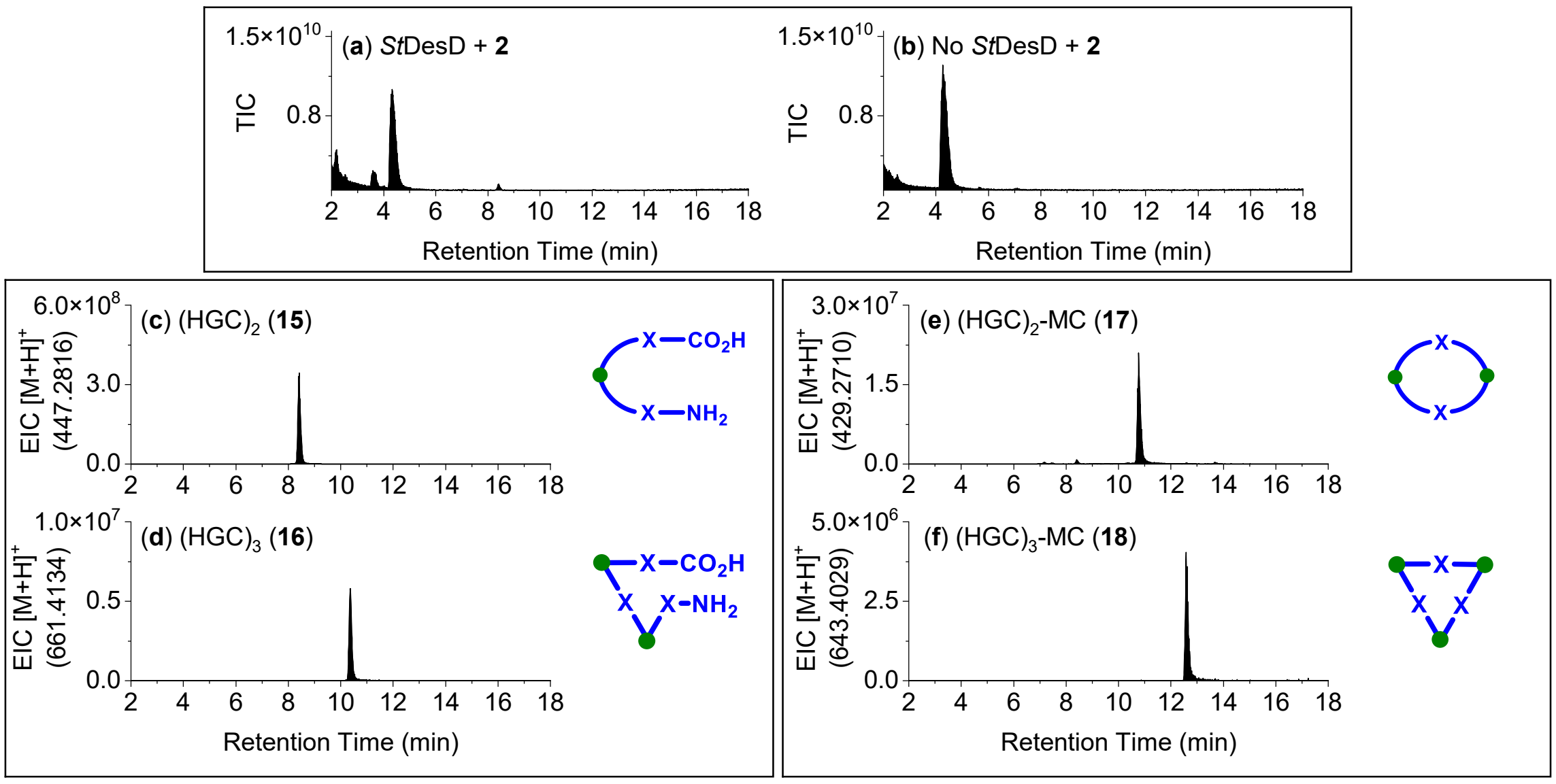

Figure S2. LC-MS TIC from a reaction solution of **2** with *St*DesD (a) or without *St*DesD (b); EIC of the $[M+H]^+$ adduct of the multimers detected from reaction of **2**: **15** (c), **16** (d), **17** (e), and **18** (f). Shaded signal identified as multimer *via* the MS^2 fragmentation pattern (**Fig S13**).

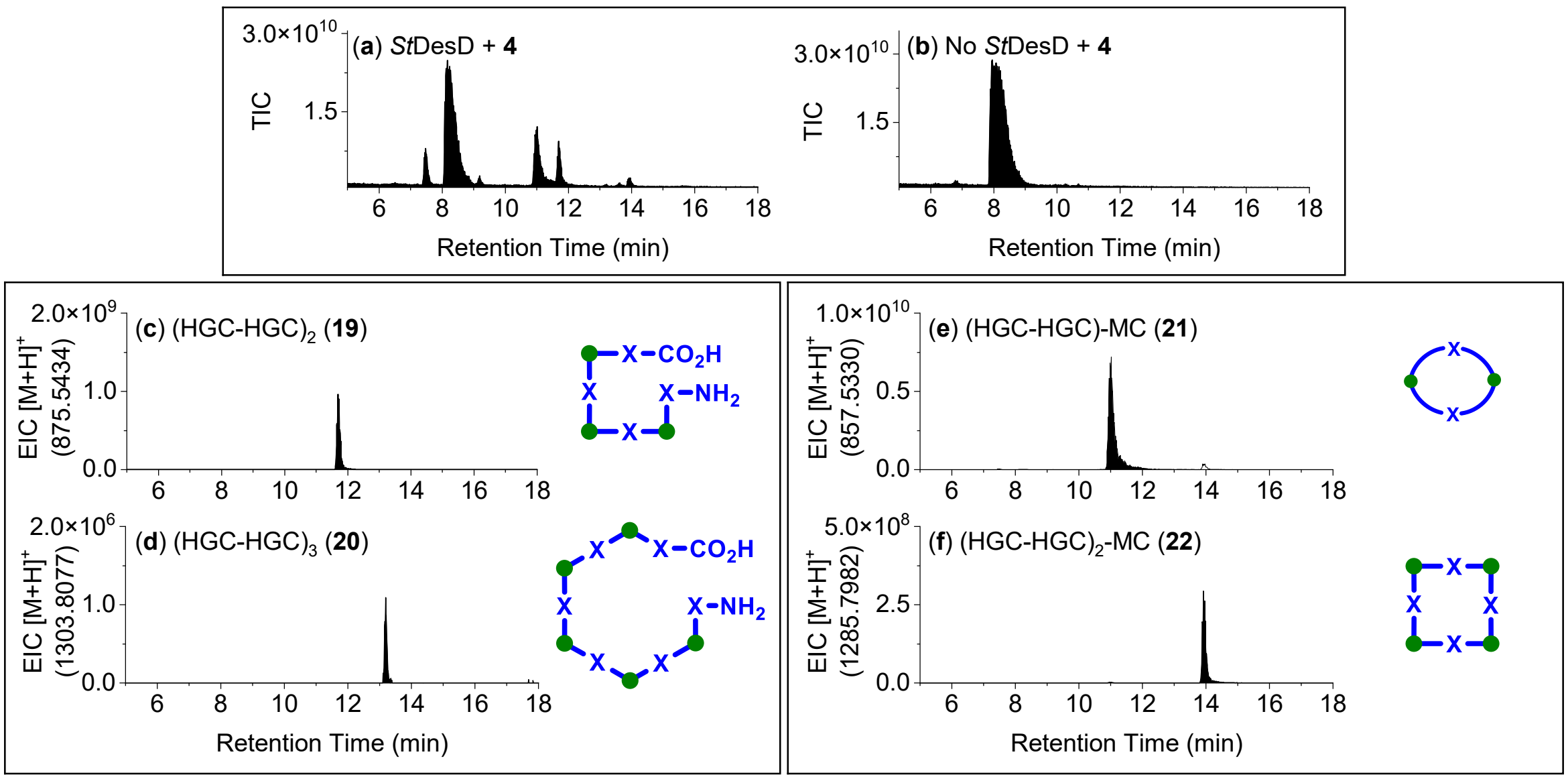

Figure S3. LC-MS TIC from a reaction solution of **4** with *St*DesD (a) or without *St*DesD (b); EIC of the $[M+H]^+$ adduct of the multimers detected from reaction of **4**: **19** (c), **20** (d), **21** (e), and **22** (f). Shaded signal identified as multimer *via* the MS^2 fragmentation pattern (**Fig. S14**).

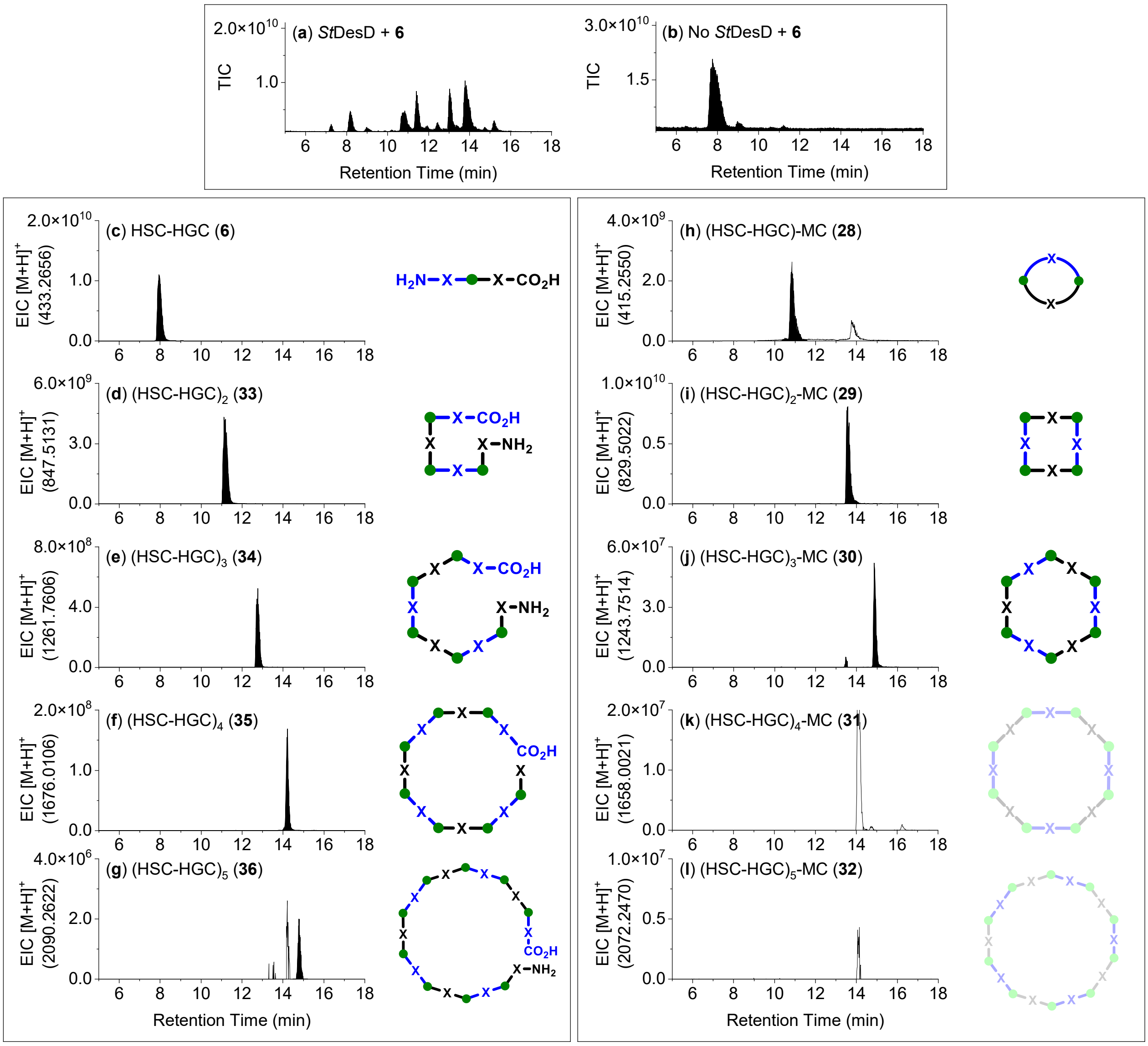

Figure S4. LC TIC from a reaction solution of **6** with *St*DesD (a) or without *St*DesD (b); and EIC of the $[M+H]^+$ adduct of the linear multimers: **6** (c), **33** (d), **34** (e), **35** (f), and **36** (g); macrocyclic multimers: **28** (h), **29** (i), **30** (j), **31** (k), and **32** (l). Shaded signal identified as multimer *via* the MS^2 fragmentation pattern (**Fig. S19–20**).

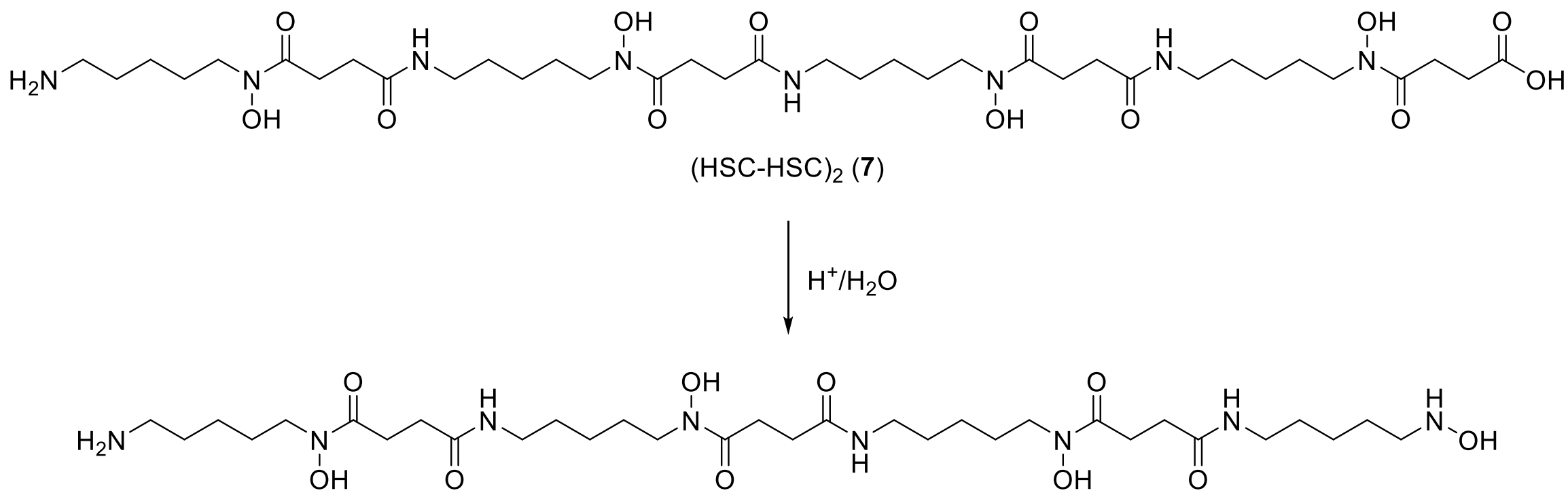

Scheme S1. De-succinylation of **7** under acidic conditions present during chemoenzymatic reaction termination forming **13**.

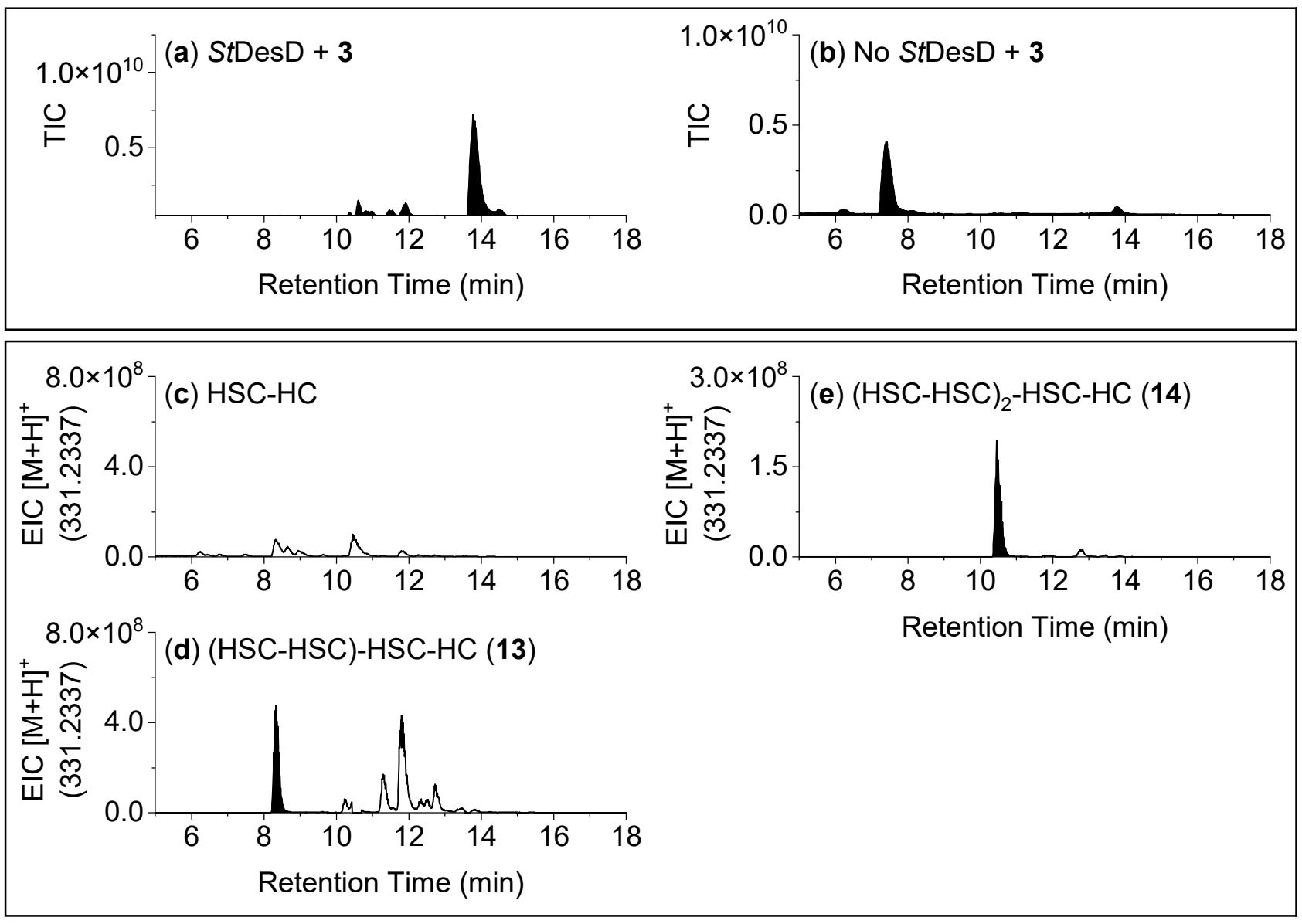

Figure S5. LC-MS TIC from a reaction solution of **3** with *St*DesD (a) or without *St*DesD (b); EIC of the $[M+H]^+$ adduct of the des-succinyl multimers from reaction of **3**: HSC-HC (c), **13** (d), and **14** (e). Shaded signal identified as multimer *via* the MS^2 fragmentation pattern (**Fig. S12**).

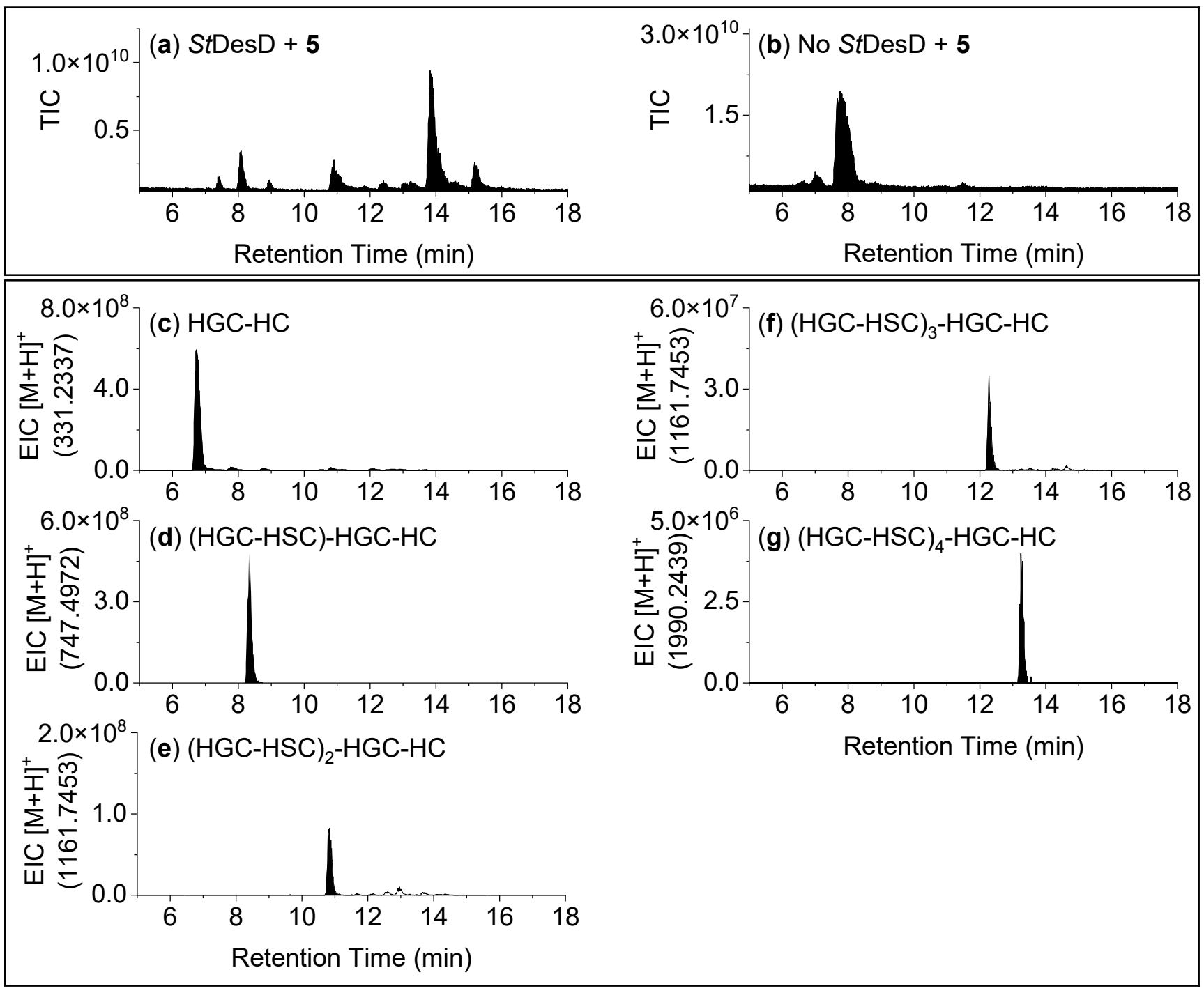

Figure S6. LC-MS TIC from a reaction solution of **5** with *St*DesD (a) or without *St*DesD (b); EIC of the $[M+H]^+$ adduct of des-succinyl multimers from reaction of **5**: HGC-HC (c), (HGC-HSC)-HGC-HC (d), $(HGC\text{-}HSC)_2$-HGC-HC (e), $(HGC\text{-}HSC)_3$-HGC-HC (f), and $(HGC\text{-}HSC)_4$-HGC-HC (g). Shaded signal identified as multimer *via* the MS^2 fragmentation pattern.

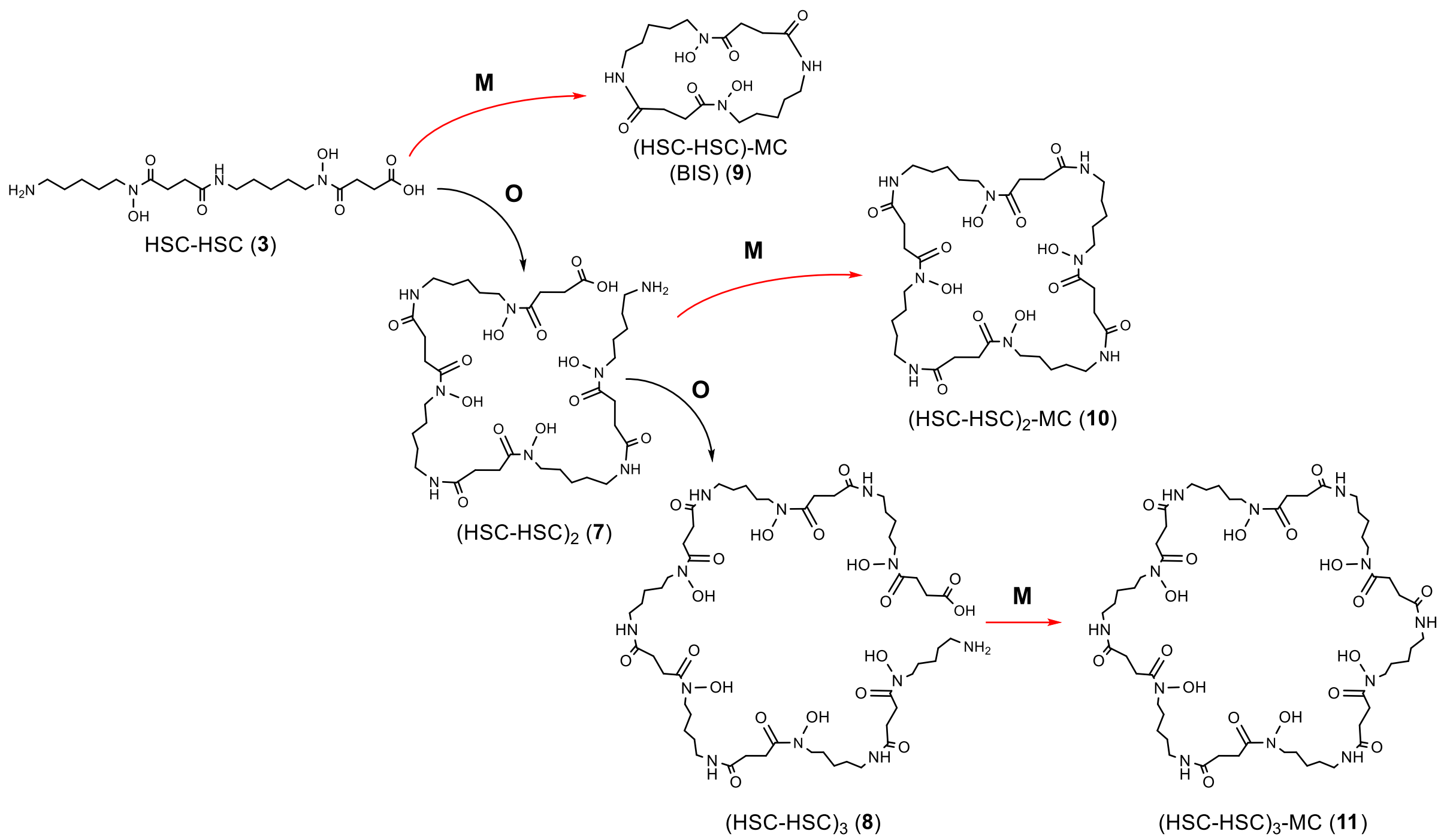

Scheme S2. Linear and macrocyclic multimers (O = oligomerization; M = macrocyclization) generated by *St*DesD from chemoenzymatic reactions using **3**.

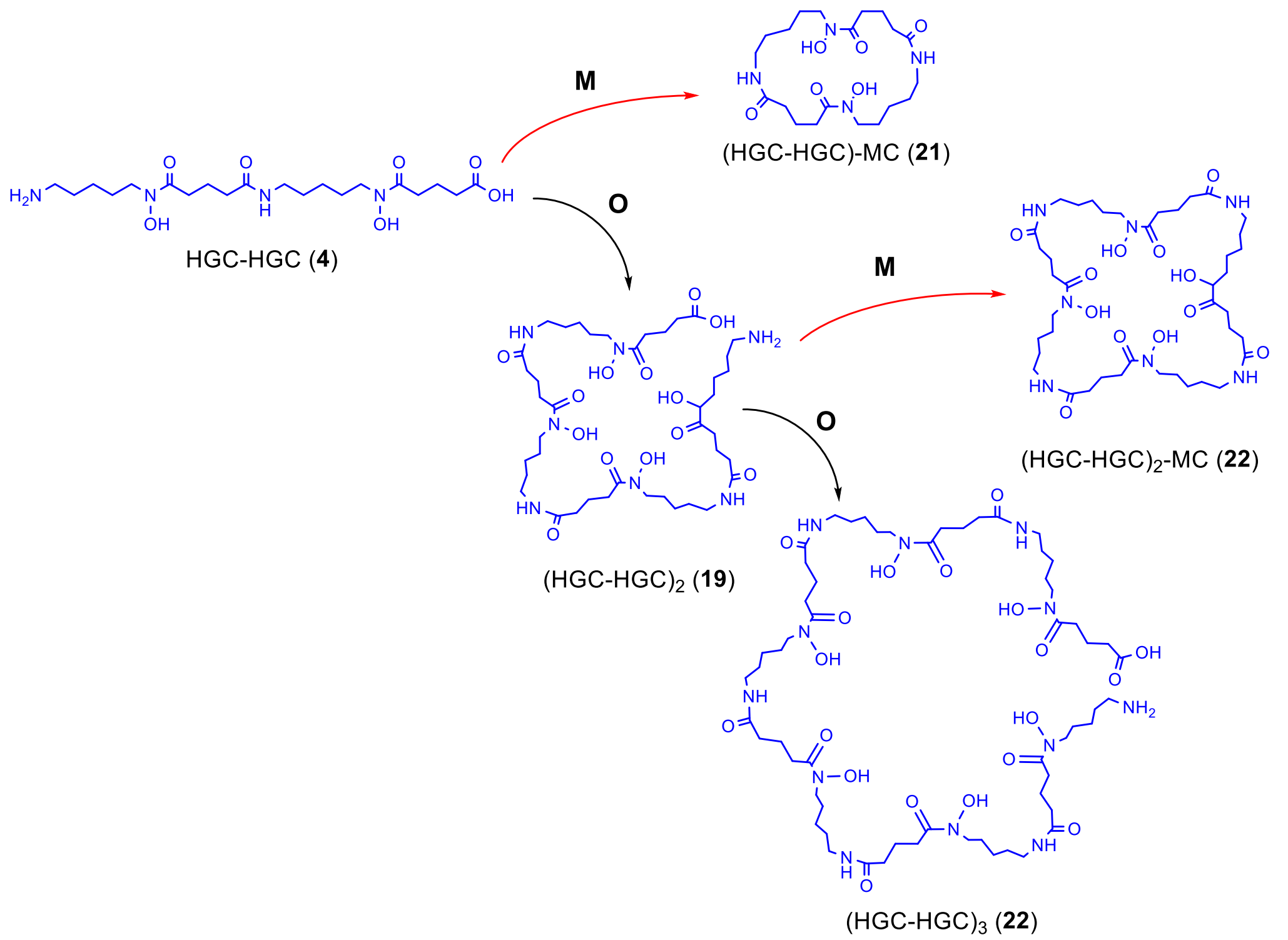

Scheme S3. Linear and macrocyclic multimers (O = oligomerization; M = macrocyclization) generated by *St*DesD from chemoenzymatic reactions using **4**.

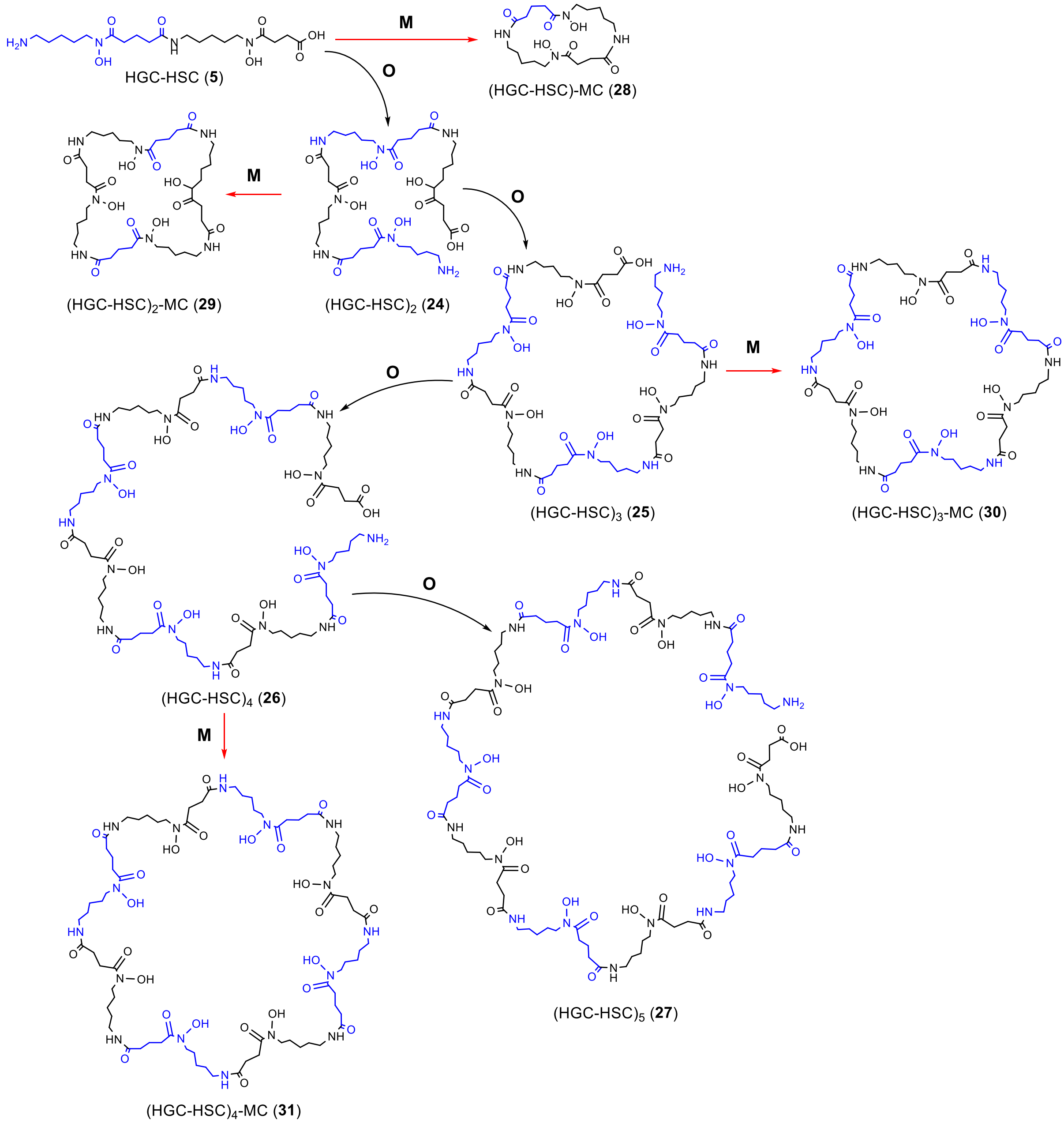

Scheme S4. Linear and macrocyclic multimers (O = oligomerization; M = macrocyclization) generated by *St*DesD from chemoenzymatic reactions using **5**.

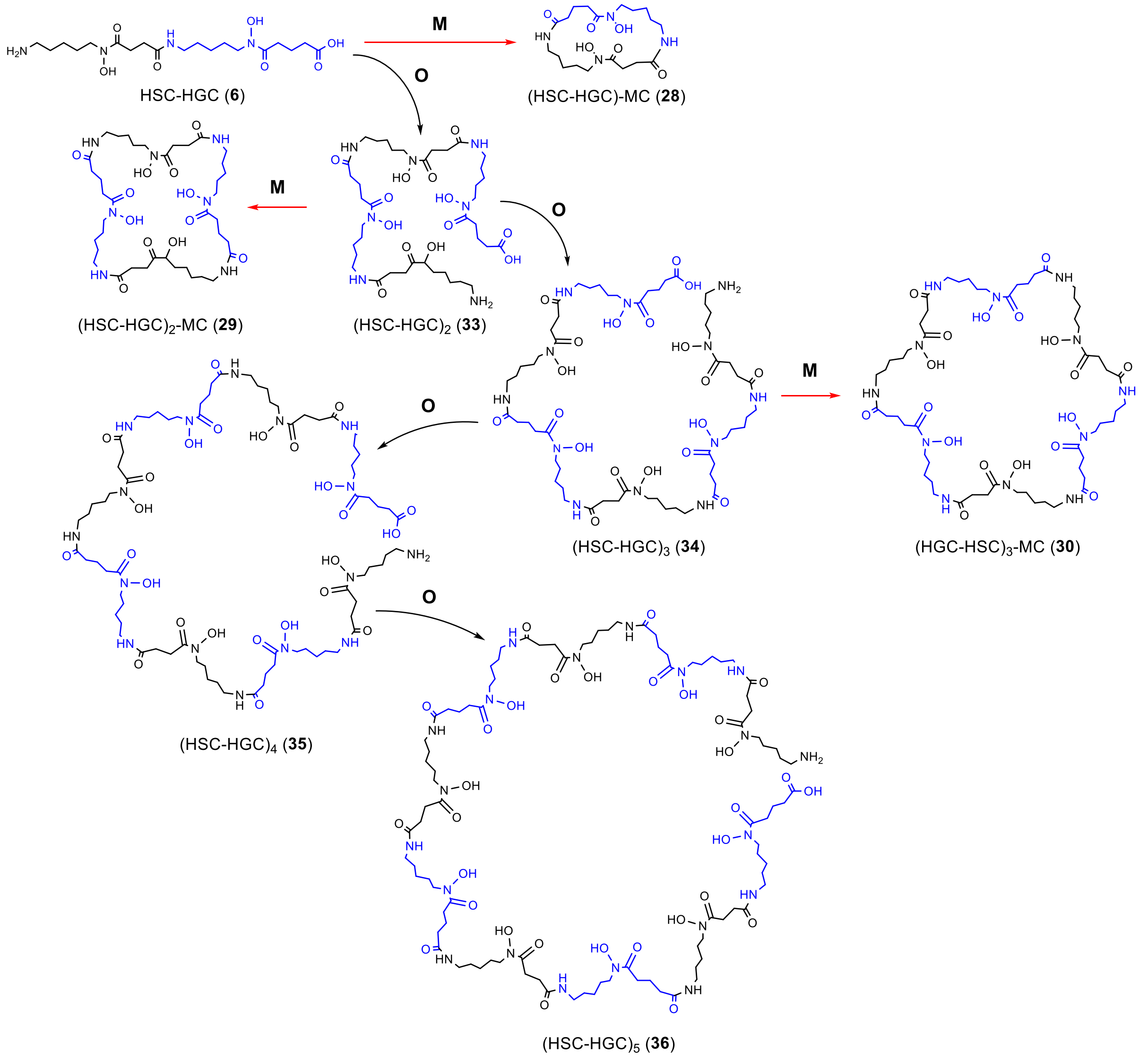

Scheme S5. Linear and macrocyclic multimers (O = oligomerization; M = macrocyclization) generated by *St*DesD from chemoenzymatic reactions using **6**.

Table S1. Number of hydroxamic acid (HXA) groups, number of amide bonds formed by *St*DesD, and the percentage of total AUC of each multimer, determined by integral of EIC traces, assembled when *St*DesD was inscubated with **1** with relevant co-factors for 24 h.

Chelator assembled from **1** by *St*DesD	# HXA groups	# Amide bonds formed by *St*DesD	Total AUC (%)*
$(HSC)_2$ (**3**)	2	1	0.1
$(HSC)_3$ ($DFOG_1$)	3	2	1
$(HSC)_4$ (**7**, $DFOS_1$)	4	3	0.01
$(HSC)_5$ ($DFOU_1$)	5	4	0.01
$(HSC)_2$-MC (**9**, BIS)	2	2	0.2
$(HSC)_3$-MC (DFOE)	3	3	93
$(HSC)_4$-MC (**10**, $DFOT_1$)	4	4	5
$(HSC)_5$-MC ($DFOV_1$)	5	5	0.9

*Total AUC = Sum of AUC of chelators identified in LC-MS analysis. HXA = Hydroxamic acid.

Table S2. Number of hydroxamic acid (HXA) groups, number of amide bonds formed by *St*DesD, and the percentage of total AUC of each multimer, determined by integral of EIC traces, assembled when *St*DesD was inscubated with **2** with relevant co-factors for 24 h.

Chelator assembled from **2** by *St*DesD	# HXA groups	# Amide bonds formed by *St*DesD	Total AUC (%)*
$(HGC)_2$ (**15**)	2	1	91
$(HGC)_3$ (**16**)	3	2	6
$(HGC)_2$-MC (1**7**)	2	2	2
$(HGC)_3$-MC (**18**)	3	3	1

*Total AUC = Sum of AUC of chelators identified in LC-MS analysis. HXA = Hydroxamic acid.

Table S3. Number of hydroxamic acid (HXA) groups, number of amide bonds formed by *St*DesD, and the percentage of total AUC of each multimer, determined by integral of EIC traces, assembled when *St*DesD was incubated with **3** with relevant co-factors for 24 h.

Multimer assembled from **3** by *St*DesD	# HXA groups	# Amide bonds formed by *St*DesD	Total AUC (%)*
$(HSC\text{-}HSC)_2$ (**7**)	4	1	0.9
$(HSC\text{-}HSC)_3$ (**8**)	6	2	0.4
(HSC-HSC)-MC (**9**)	2	1	14
$(HSC\text{-}HSC)_2$-MC (**10**)	4	2	85
$(HSC\text{-}HSC)_3$-MC (**11**)	6	3	0.4

*Total AUC = Sum of AUC of multimers identified in LC-MS analysis. HXA = Hydroxamic acid.

Table S4. Number of hydroxamic acid (HXA) groups, number of amide bonds formed by *St*DesD, and the percentage of total AUC of each multimer, determined by integral of EIC traces, assembled when *St*DesD was incubated with **4** with relevant co-factors for 24 h.

Chelator assembled from **4** by *St*DesD	# HXA groups	# Amide bonds formed by *St*DesD	Total AUC (%)*
$(HGC\text{-}HGC)_2$ (**19**)	4	1	6
$(HGC\text{-}HGC)_3$ (**20**)	6	2	0.005
(HGC-HGC)-MC (**21**)	2	1	92
$(HGC\text{-}HGC)_2$-MC (**22**)	4	2	2

*Total AUC = Sum of AUC of chelators identified in LC-MS analysis. HXA = Hydroxamic acid.

Table S5. Number of hydroxamic acid (HXA) groups, number of amide bonds formed by *St*DesD, and the percentage of total AUC of each multimer, determined by integral of EIC traces, assembled when *St*DesD was incubated with **5** with relevant co-factors for 24 h.

Multimer assembled from **5** by *St*DesD	# HXA groups	# Amide bonds formed by *St*DesD	Total AUC (%)*
$(HGC\text{-}HSC)_2$ (**24**)	4	1	2
$(HGC\text{-}HSC)_3$ (**25**)	6	2	0.7
$(HGC\text{-}HSC)_4$ (**26**)	8	3	0.3
$(HGC\text{-}HSC)_5$ (**27**)	10	4	0.01
(HGC-HSC)-MC (**28**)	2	1	27
$(HGC\text{-}HSC)_2$-MC (**29**)	4	2	64
$(HGC\text{-}HSC)_3$-MC (**30**)	6	3	0.8
$(HGC\text{-}HSC)_4$-MC (**31**)	8	4	0.05

*Total AUC = Sum of AUC of multimers identified in LC-MS analysis. HXA = Hydroxamic acid.

Table S6. Number of hydroxamic acid (HXA) groups, number of amide bonds formed by *St*DesD, and the percentage of total AUC of each multimer, determined by integral of EIC traces, assembled when *St*DesD was incubated with **6** with relevant co-factors for 24 h.

Multimer assembled from **6** by *St*DesD	# HXA groups	# Amide bonds formed by *St*DesD	Total AUC (%)*
$(HSC\text{-}HGC)_2$ (**33**)	4	1	29
$(HSC\text{-}HGC)_3$ (**34**)	6	2	2
$(HSC\text{-}HGC)_4$ (**35**)	8	3	0.6
$(HSC\text{-}HGC)_5$ (**36**)	10	4	0.007
(HSC-HGC)-MC (**28**)	2	1	20
$(HSC\text{-}HGC)_2$-MC (**29**)	4	2	48
$(HSC\text{-}HGC)_3$-MC (**30**)	6	3	0.2

*Total AUC = Sum of AUC of multimers identified in LC-MS analysis. HXA = Hydroxamic acid.

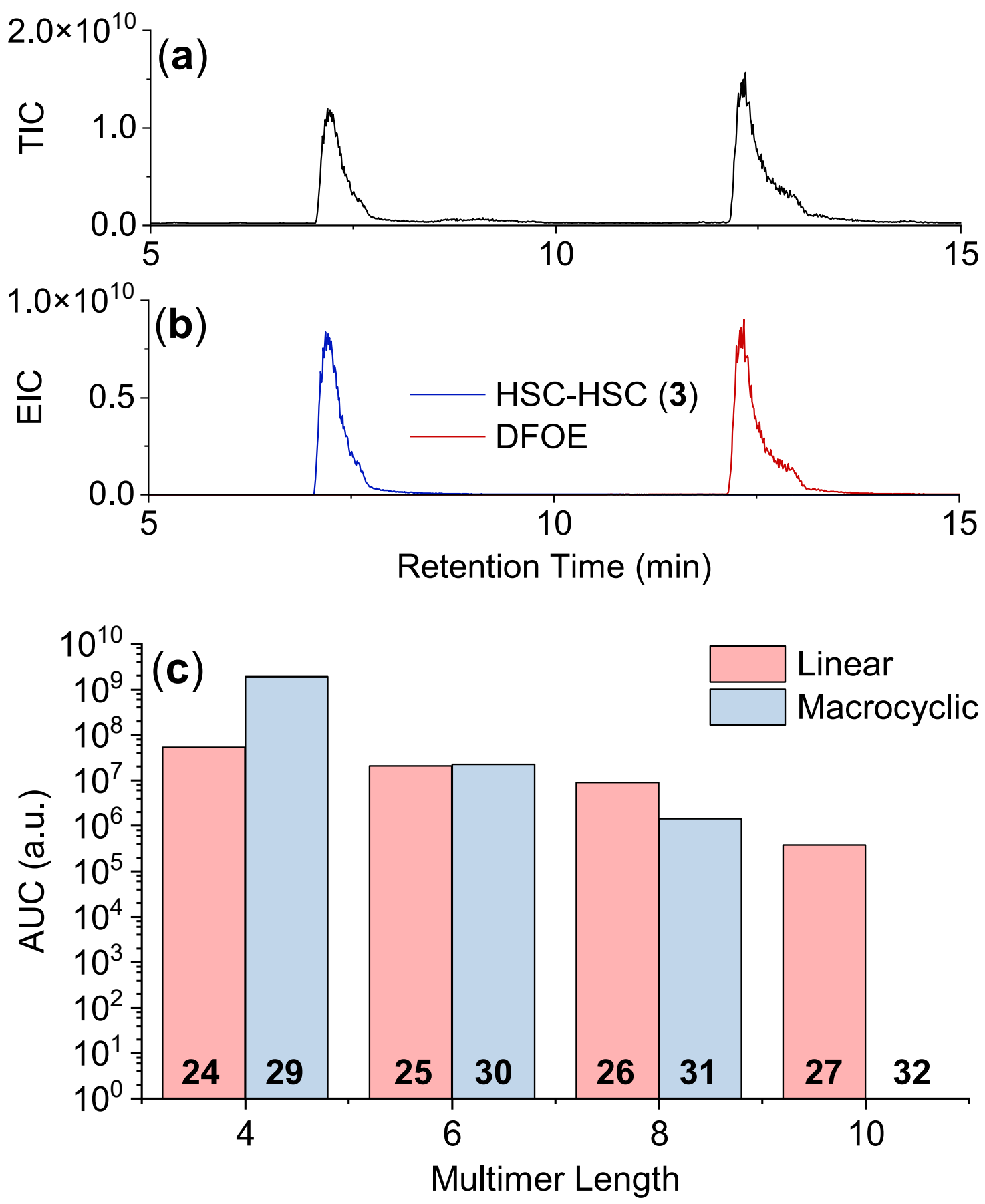

Figure S7. TIC from an equimolar (0.5 mM) solution of **3** and DFOE (**a**); EIC ($[M+H]^+$ adducts) of **3** and DFOE (b); and AUC values of EIC peaks assigned to **24**, **25**, **26**, **27**, **29**, **30**, **31**, and **32** detected in the reaction solution from *St*DesD and **5** (c).

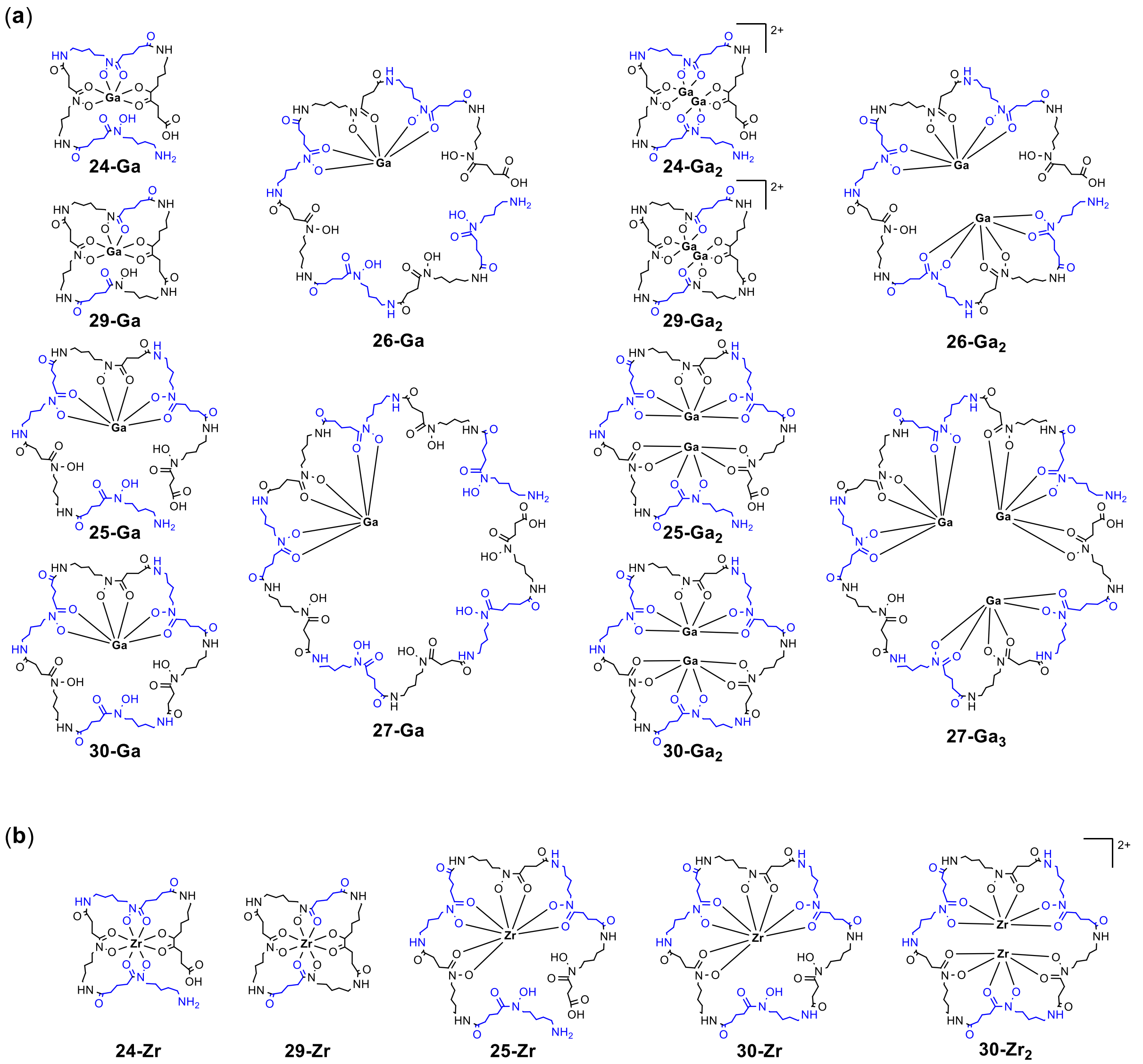

Figure S8. Representative metal complexes (noting the possibility of coordinate isomers) generated by incubation of mixture of high denticity multimers (**24 – 31)** with Ga(III) (**a**) or Zr(IV) (**b**) characterised *in situ* by LC-MS[2].

Substrate Standard Curves

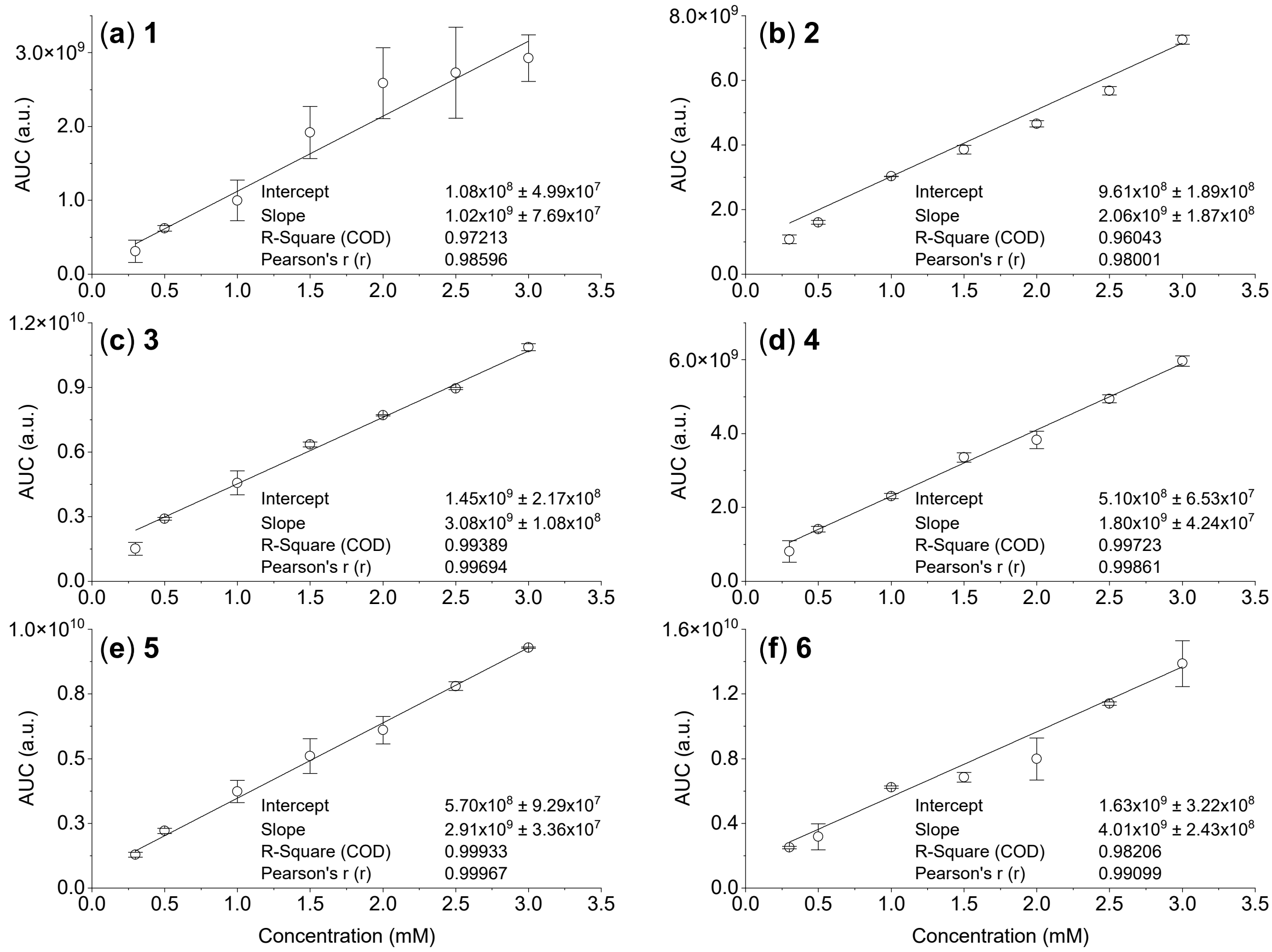

Figure S9. Substrate standard curves for **1** (a), **2**, (b), **3** (c), **4** (d), **5** (e), and **6** (f) covering concentrations ranging for 0.5 mM to 3.0 mM. AUC determined by integration of EIC of the $[M+H]^+$ adduct.

Fragmentation Patterns of Multimers Assembled by *St*DesD

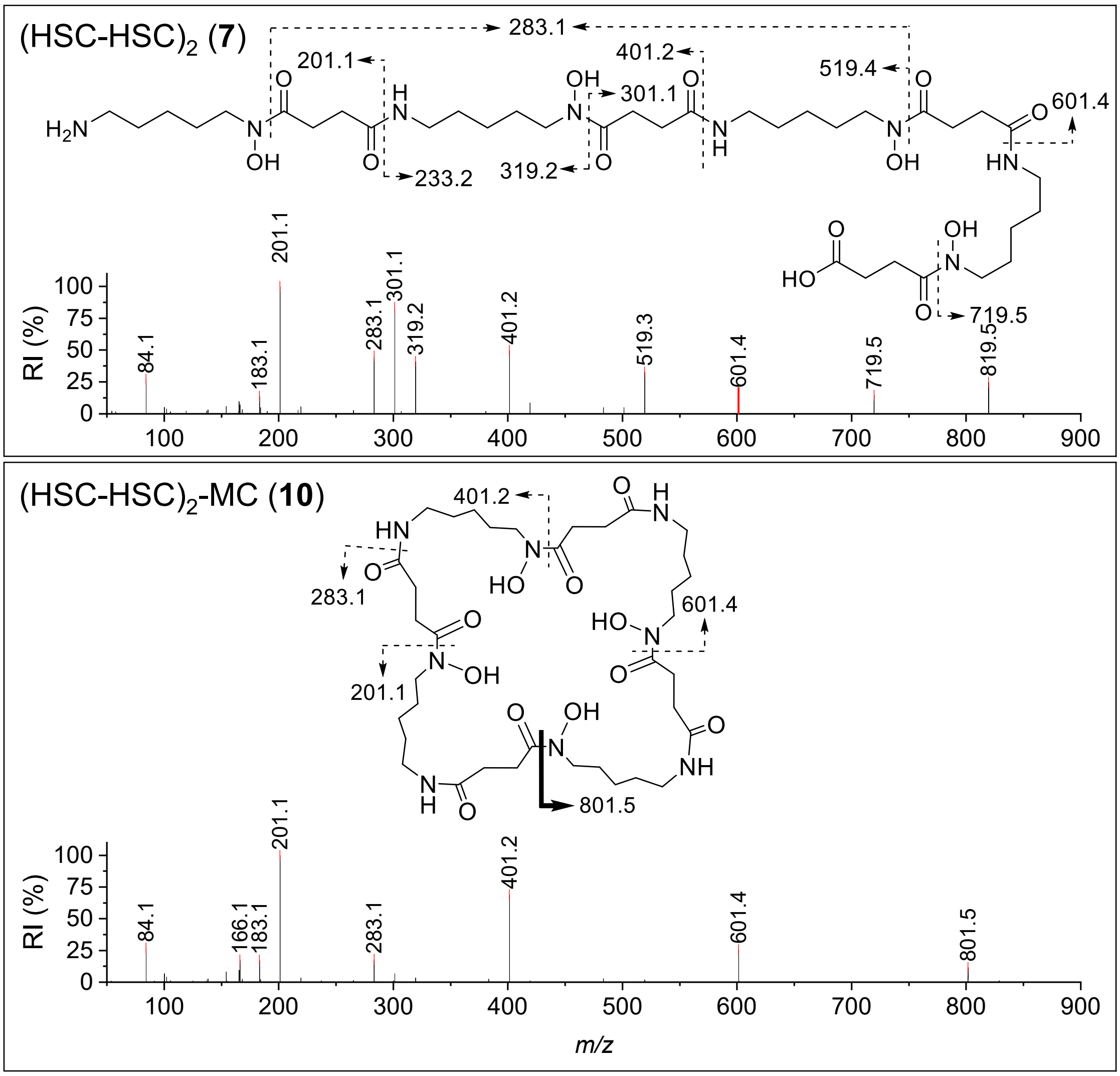

Figure S10. MS[2] fragmentation pattern and fragmentation diagram of the chemoenzymatically assembled multimers **7**, and **10** produced when *St*DesD was incubated with **3**. Bolded arrow of macrocycle indicates all fragments end at the arrow.

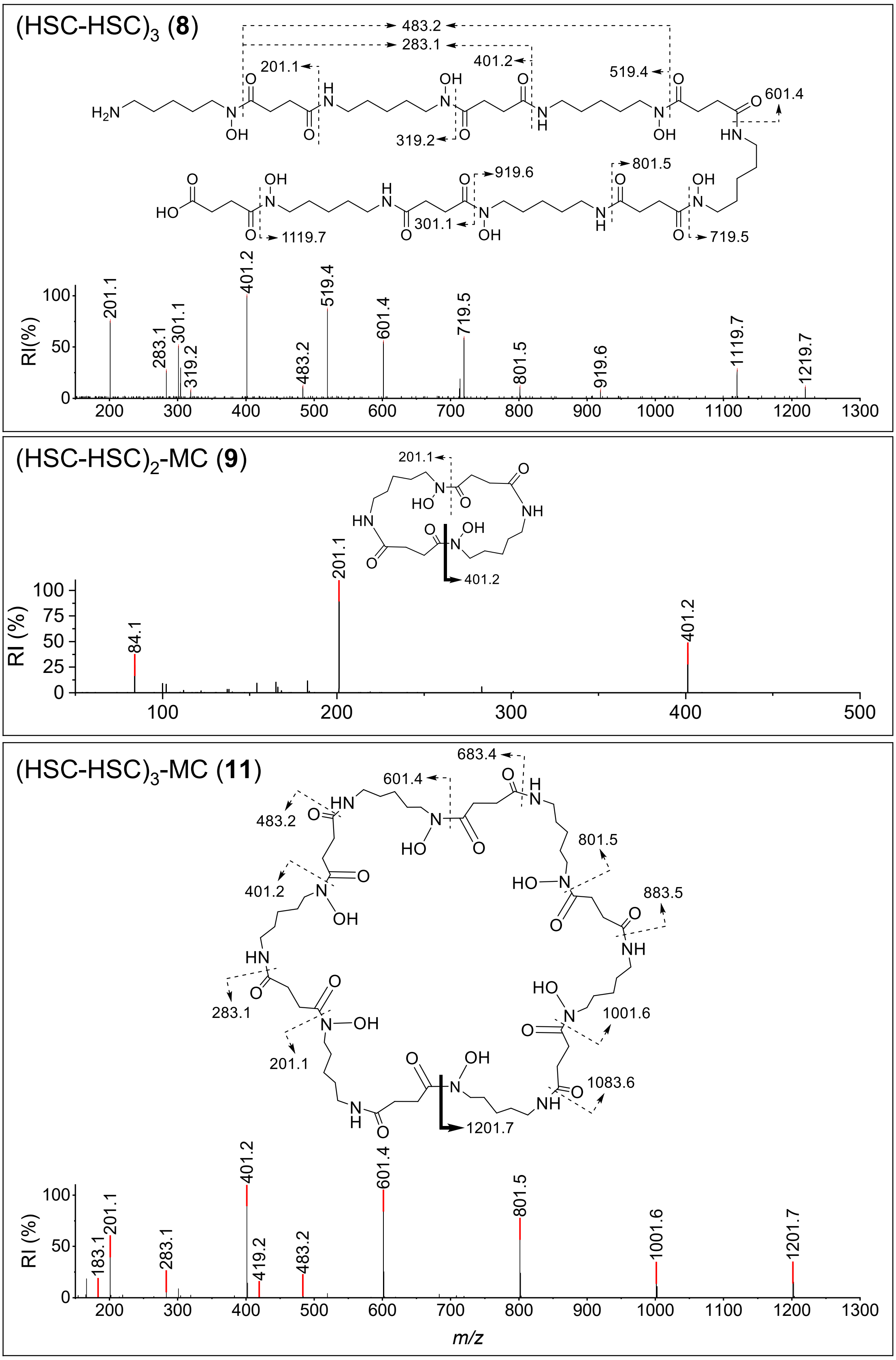

Figure S11. MS^2 fragmentation patterns and fragmentation diagrams of the chemoenzymatically assembled hexameric multimers **8**, and **11** produced when *St*DesD was incubated with **3**. Bolded arrow of macrocycle indicates all fragments end at the arrow.

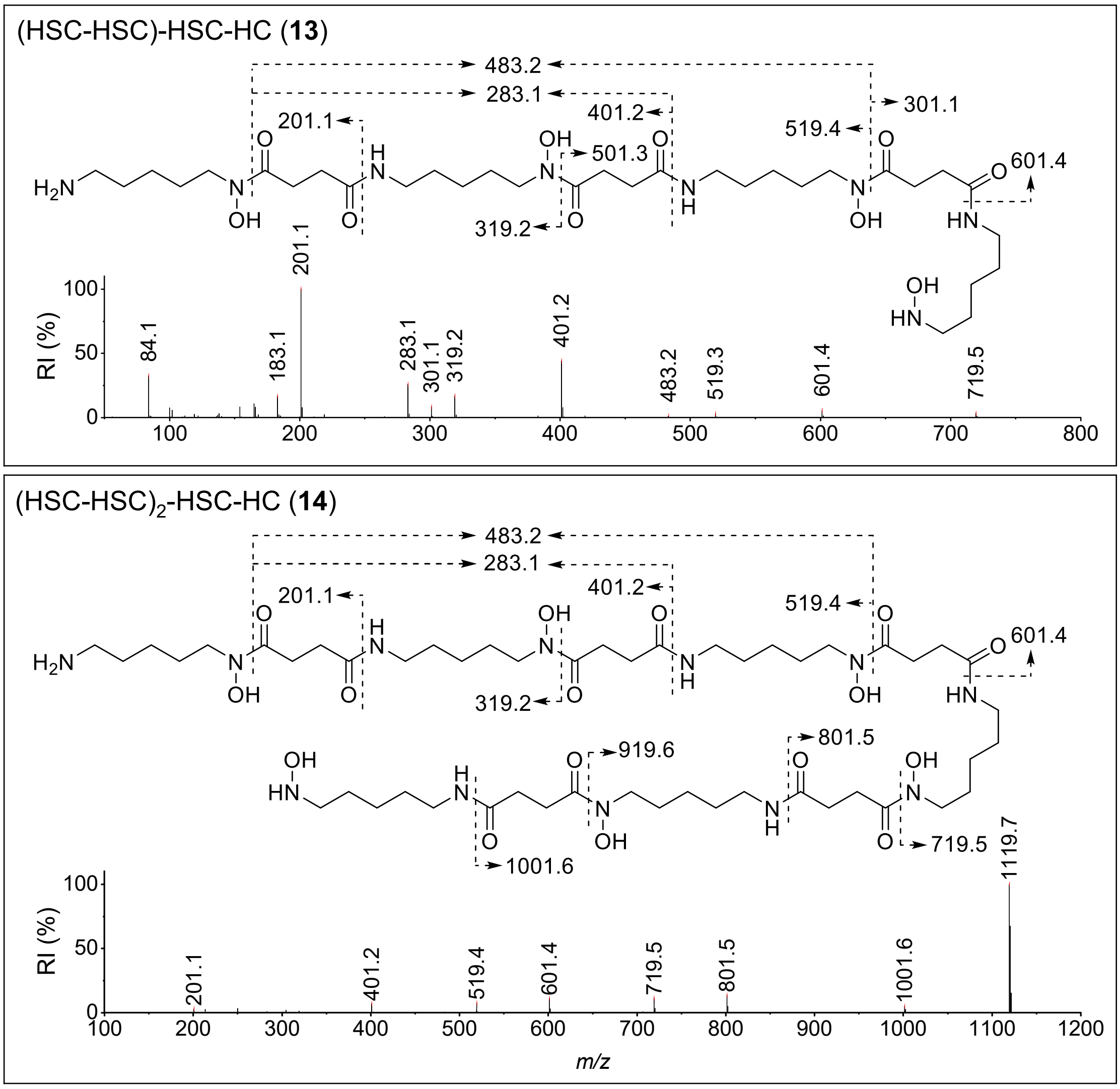

Figure S12. MS[2] fragmentation patterns and fragmentation diagrams of the chemoenzymatically assembled de-succinylated multimers **13**, and **14** produced when *St*DesD was incubated with **3**.

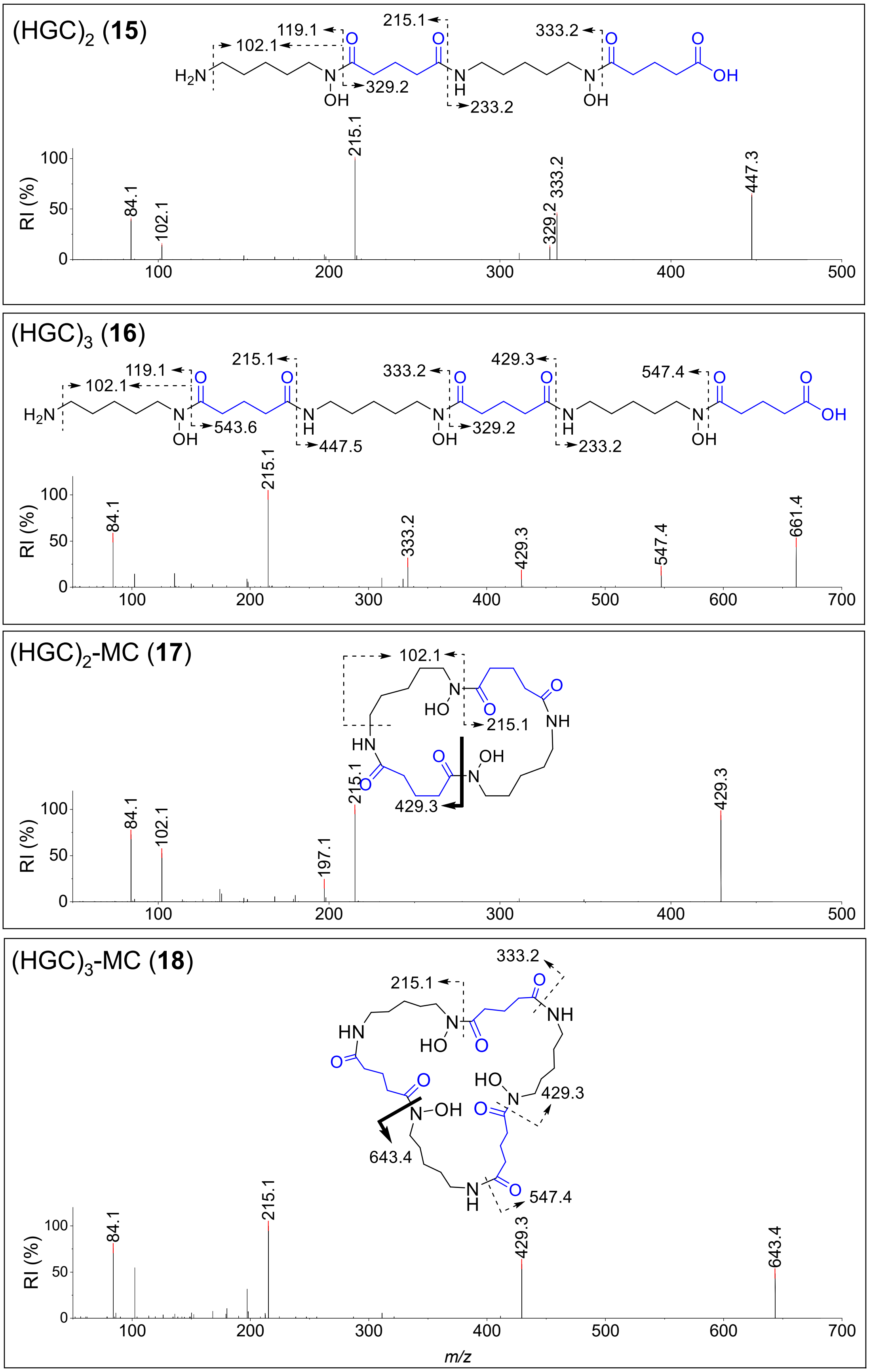

Figure S13. MS^2 fragmentation pattern and fragmentation diagram of the chemoenzymatically assembled multimers **15**, **16**, **17**, and **18** produced when *St*DesD was incubated with **2**. Bolded arrow of macrocycle indicates all fragments end at the arrow.

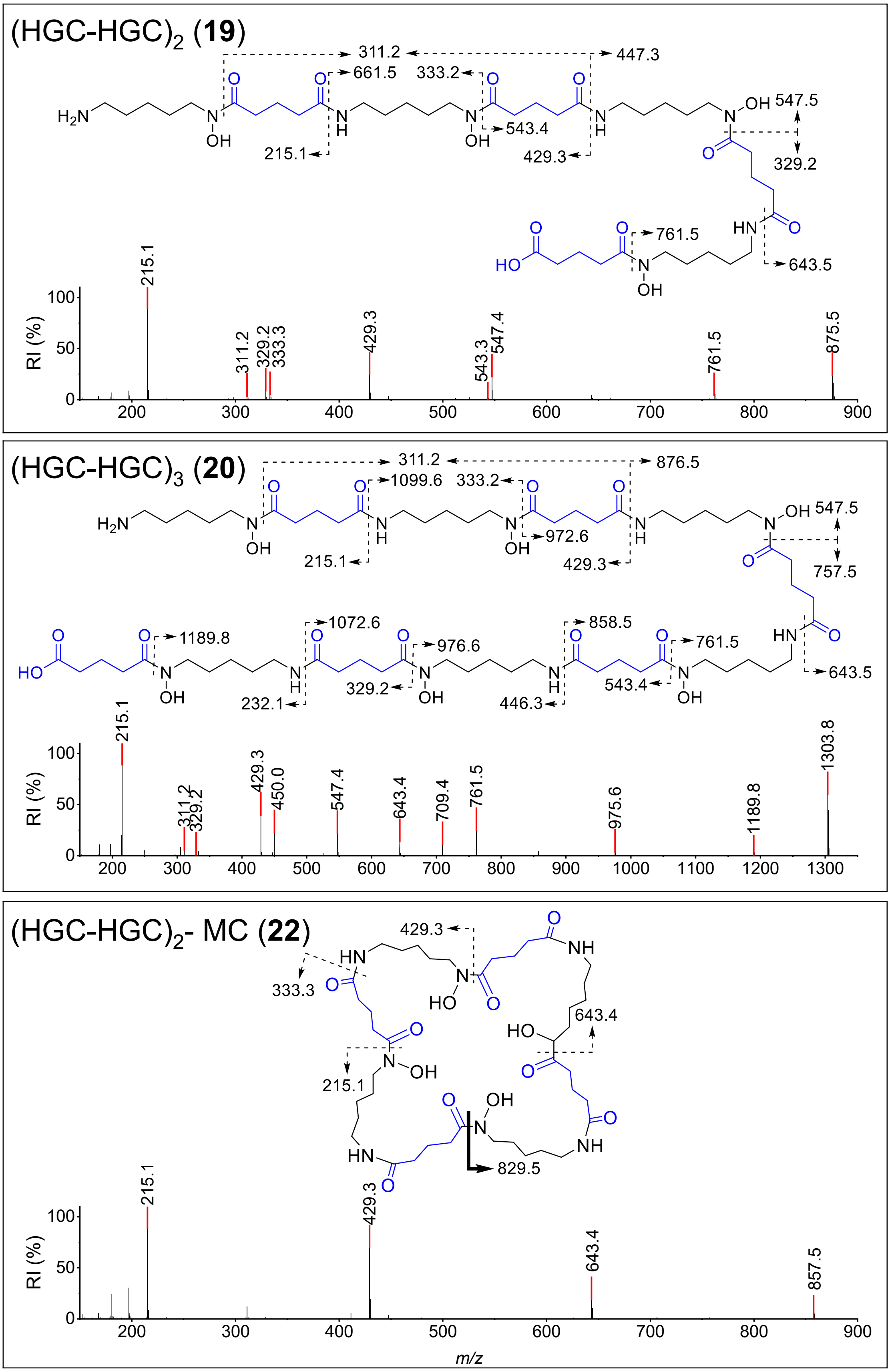

Figure S14. MS^2 fragmentation patterns and fragmentation diagrams of the chemoenzymatically assembled multimers **19**, **20**, and **22** produced when *St*DesD was incubated with **4**. Bolded arrow of macrocycle indicates all fragments end at the arrow.

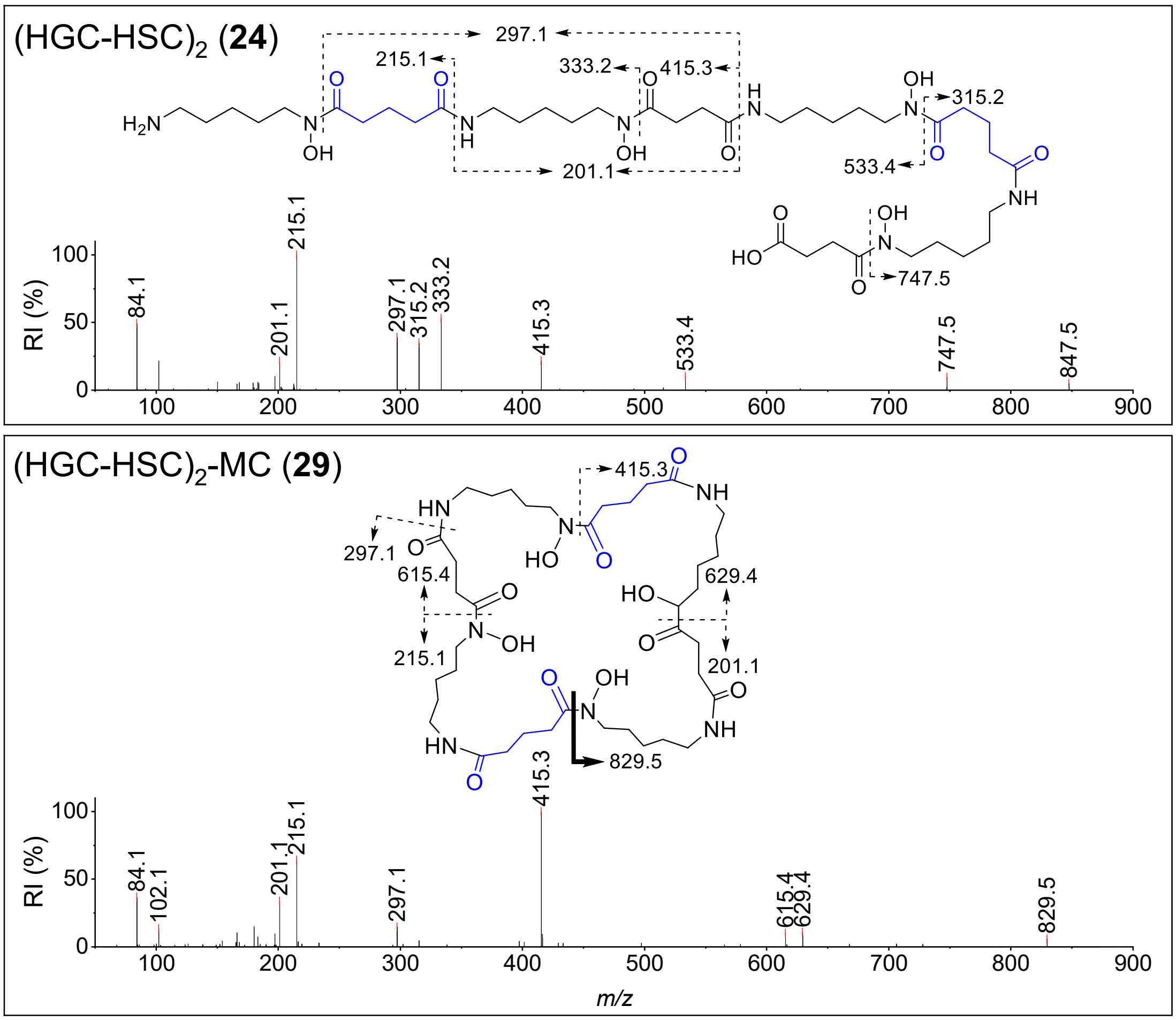

Figure S15. MS[2] fragmentation patterns and fragmentation diagrams of the chemoenzymatically assembled dimeric and tetrameric multimers **24**, and **29** produced when *St*DesD was incubated with **5**. Bolded arrow of macrocycle indicates all fragments end at the arrow.

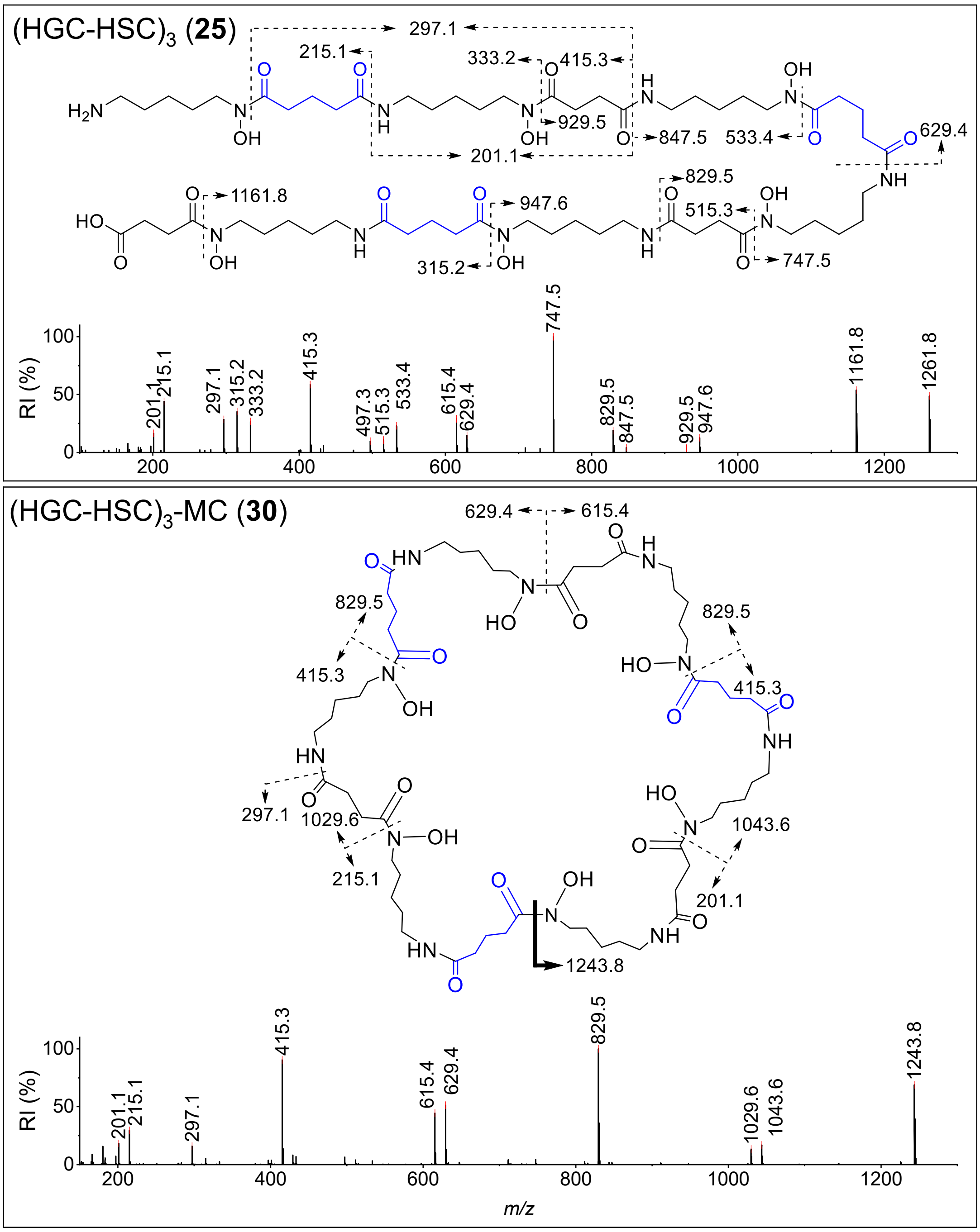

Figure S16. MS[2] fragmentation patterns and fragmentation diagrams of the chemoenzymatically assembled hexameric multimers **25**, and **30** produced when *St*DesD was incubated with **5**. Bolded arrow of macrocycle indicates all fragments end at the arrow.

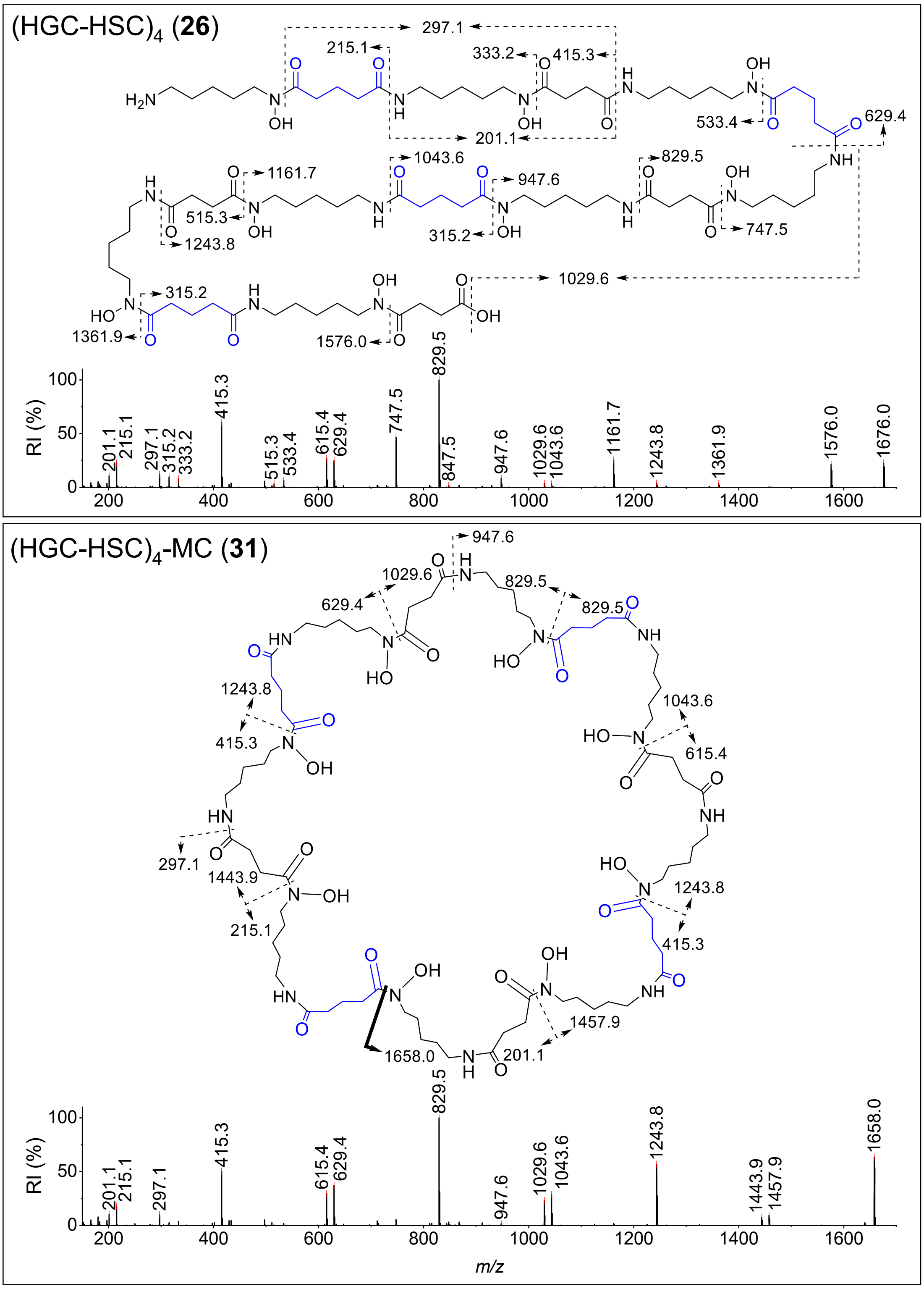

Figure S17. MS^2 fragmentation patterns and fragmentation diagrams of the chemoenzymatically assembled octameric multimers **26**, and **31** produced when *St*DesD was incubated with **5**. Bolded arrow of macrocycle indicates all fragments end at the arrow.

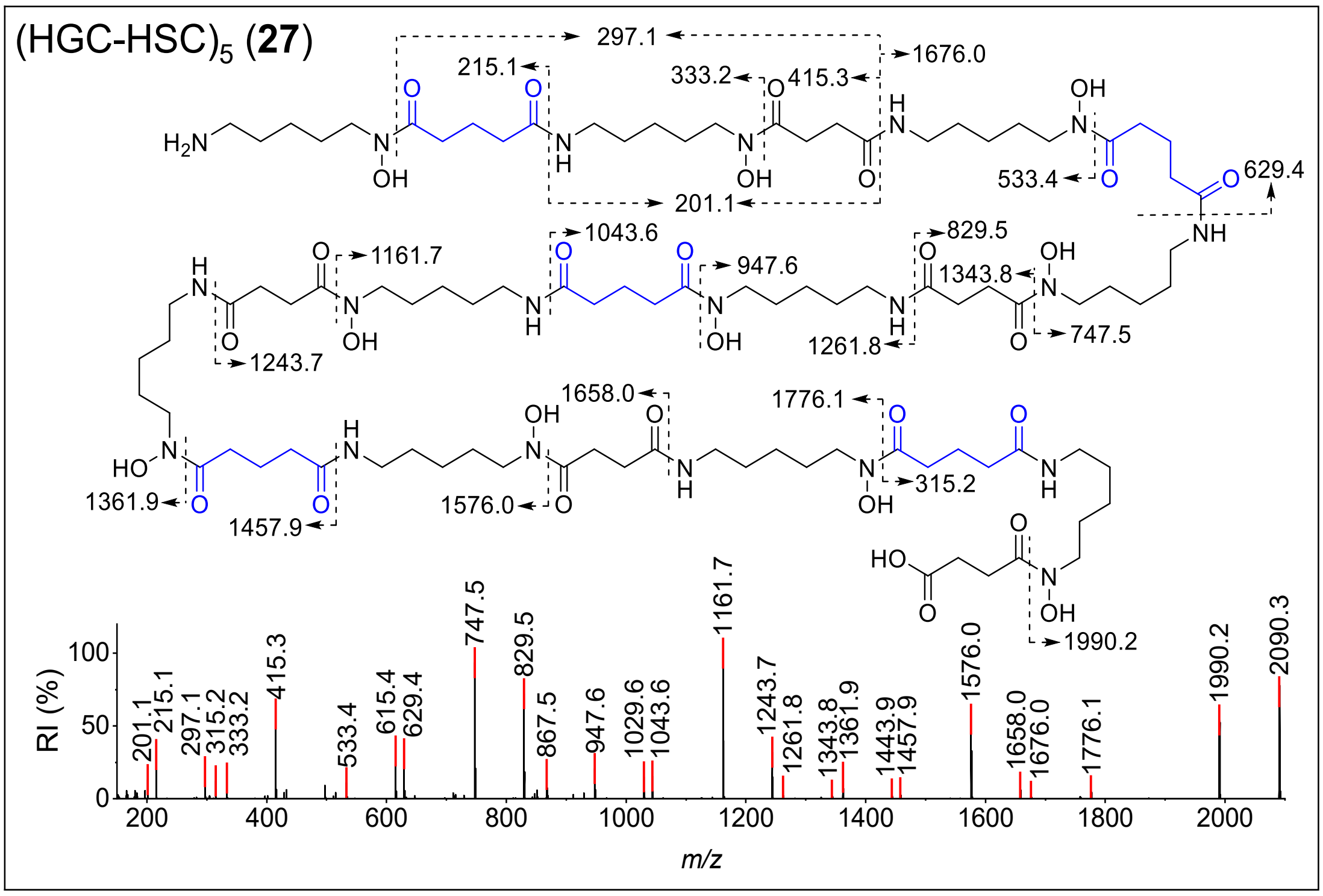

Figure S18. MS[2] fragmentation pattern and fragmentation diagram of the chemoenzymatically assembled decameric multimer **27** produced when *St*DesD was incubated with **5**.

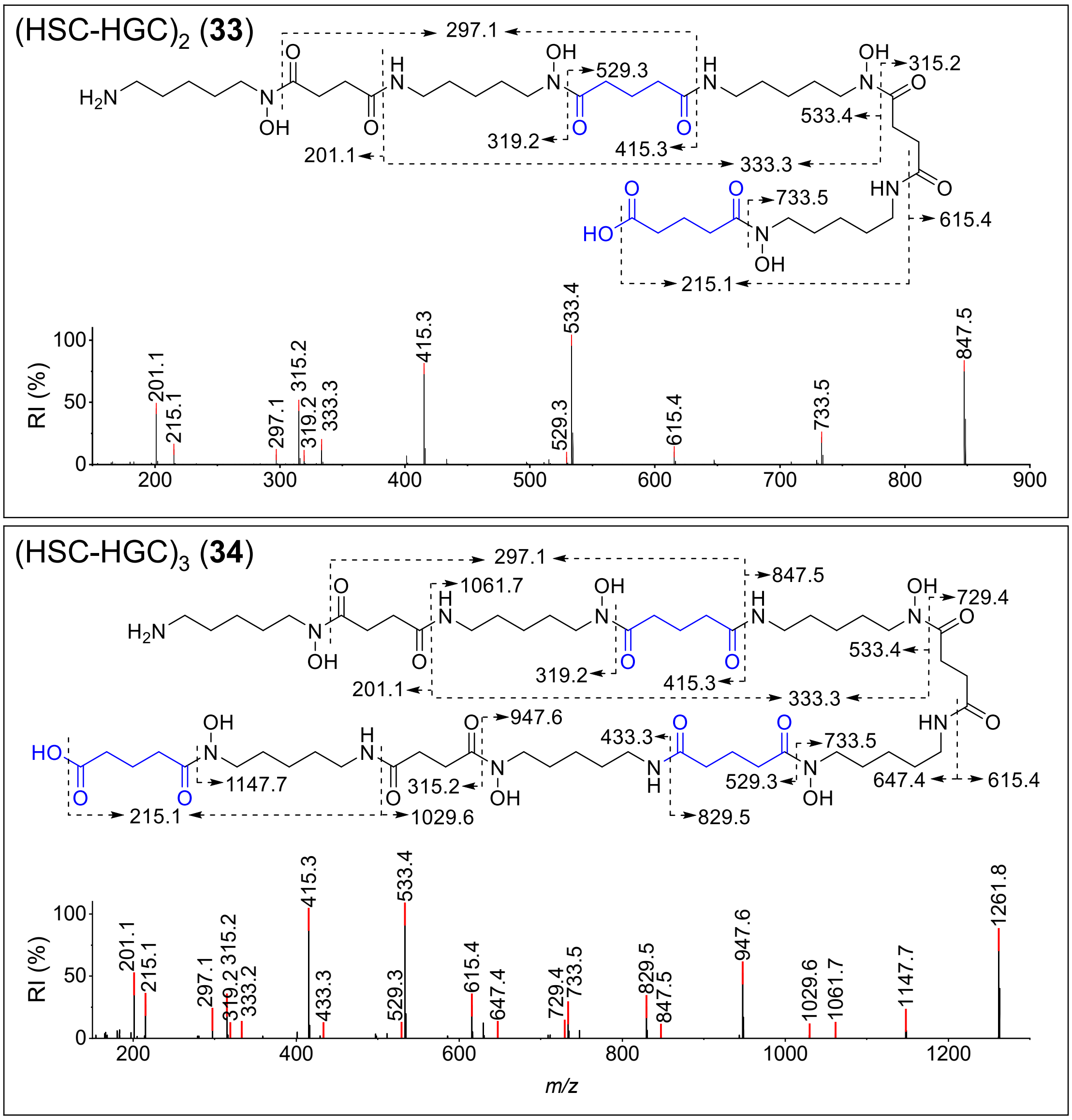

Figure S19. MS[2] fragmentation patterns and fragmentation diagrams of the chemoenzymatically assembled linear multimers **33** and **34** produced when *St*DesD was incubated with **6**.

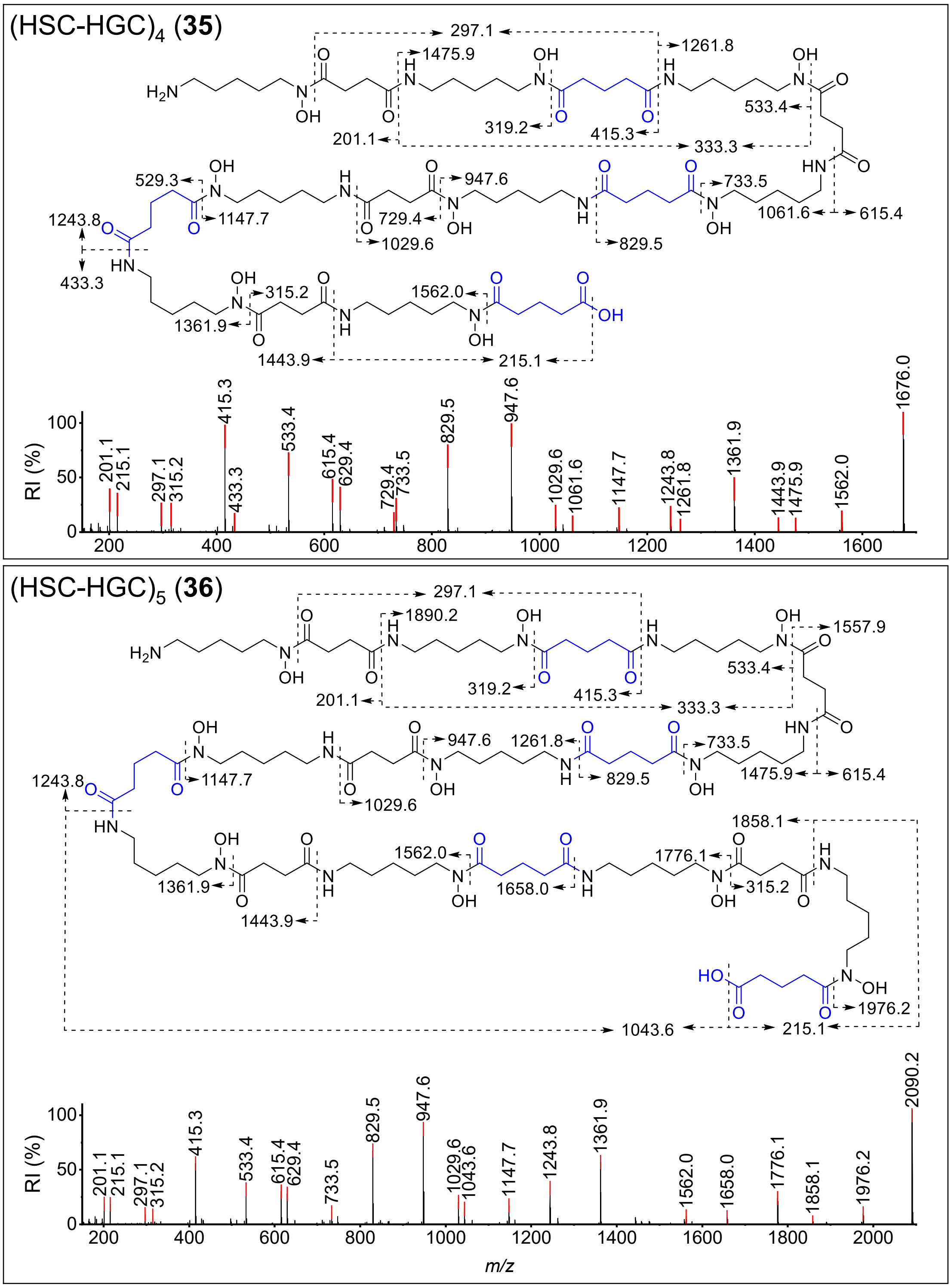

Figure S20. MS² fragmentation patterns and fragmentation diagrams of the chemoenzymatically assembled linear multimers **35** and **36** produced when *St*DesD was incubated with **6**.

Materials

Adenosine 5'-triphosphate disodium salt hydrate (Grade II, 98.5%), AEBSF (97%), 5-aminopentan-1-ol (92%), ammonium formate (97%), deoxyribonuclease I from bovine pancreas, 1,8-diazabicyclo[5.4.0]undec-7-ene (DBU) (98%), dichloromethane (DCM) (99%), diisopropyl azodicarboxylate (DIAD) (98%), 4-(dimethylamino)pyridine (DMAP) (99%), di-*tert*-butyl dicarbonate (≥97%), glutaric anhydride (95%), IPTG (≥99%), kanamycin sulfate (≤5% kanamycin B), imidazole (99%), *N,N*-diisopropylethylamine (DIPEA) (99%), magnesium chloride heptahydrate (99%), magnesium sulphate anhydrous (97%), 2-nitrobenzenesulphonyl chloride (97%), palladium on carbon (Pd/C) (10 wt%), sodium hydrogen carbonate anhydrous (95%), succinic anhydride (95%), tetrahydrofuran (THF) (99%), triethylamine (99%), trifluoroacetic acid (TFA) (99%), triphenylphosphine (99%), TRIS/HCl (≥99%), and yeast extract were obtained from Sigma Aldrich. *O*-benzylhydroxylamine hydrochloride (97%), and *O*-*tert*-butylhydroxylamine hydrochloride (97%) were obtained from Ambeed Inc. Celite 545, ethanol (99%), ethyl acetate (99%), glycerol (99.5%), piperidine (99%), and sodium chloride (95%) were obtained from ChemSupply Australia. Acetonitrile (99%), and *N,N*-dimethylformamide (DMF) (99%) were obtained from ACI LabScan. Chloroform (99%), n-hexane (95%), hydrochloric acid (37%), 2-mercaptoethanol (97%), and pyridine (99%) were obtained from Ajax FineChem. Hexafluorophosphate benzotriazole tetramethyl uranium (HBTU) (95%) was obtained from Alfa Aesar. Acetonitrile LCMS grade, Optima water, and Oxoid™ tryptone were obtained from Thermo-fisher Scientific. All reagents were used without purification unless stated otherwise.

General Methodology

Auto-Flash chromatography. Normal phase auto-flash chromatography was performed using a Grace Reveleris X2 or Biotage® Selekt Auto-Flash system using hexane (hex) and ethyl acetate (EtOAc) as eluting solvents with pre-packed Buchi Reveleris FlashPure or Biotage® Sfär HC cartridges respectively. Reverse phase auto-flash chromatography was performed using a Biotage® Selekt Auto-Flash system using milli-Q water and acetonitrile (MeCN) as eluting solvents with pre-packed Biotage® Sfär C18 cartridges. Auto-flash column chromatography solvent programs are expressed in column volumes (CV) and are stated below for each compound.

Preparative HPLC. Preparative HPLC purification was performed using a Shimadzu LC-20 series high performance liquid chromatography system with two LC-20AP preparative pumps, a SIL-10AP autosampler, FRC-10A fraction collector, and a SPD-20A UV/Vis detector. A Shim-pack GIS-C18 column (150 mm x 20 mm ID, 5 µm) was used at 20 mL min^{-1}, unless otherwise stated. Mobile phase A: 0.1% formic acid in Milli-Q water; mobile phase B: 0.1% formic acid in acetonitrile. Solvent programs are expressed in time (min) as stated below for each compound.

NMR spectroscopy. ^{1}H and ^{13}C NMR spectra were acquired on a 600 MHz Bruker Avance III NMR spectrometer equipped with a TCI probe at 600 MHz and 150 MHz, respectively. Spectra were recorded at 298 K using DMSO-d_6, $CDCl_3$, or D_2O as the solvent and internal lock. All spectra were referenced to the residual solvent signal (DMSO-d_6: ^{1}H 2.50 ppm, ^{13}C 39.52 ppm; $CDCl_3$: ^{1}H 7.26 ppm, ^{13}C 77.16 ppm; D_2O: ^{1}H 4.79 ppm) and are reported as follows: (1) chemical shift (ppm), (2) integration, (3) multiplicity (s, singlet; brs, broad singlet; d, doublet; dd, doublet of doublets; t, triplet; m, multiplet; quin, quintet); (4) coupling constant (Hz).

Liquid chromatography-high resolution tandem mass spectrometry (LC-HRMS/MS). An Agilent Zorbax Eclipse XBD-C18 column (150 mm x 2.1 mm; I.D. 3.5 µm) was maintained at 30 °C and a sample injection of 5 µL was used for all experiments. HRMS and LC-MS^2 analysis was performed using a Thermo-Fisher Q-Exactive Hybrid Quadrupole-Orbitrap mass spectrometer coupled to a Thermo-Fisher Vanquish Horizon UHPLC with inbuilt degasser, temperature-controlled column compartment and autoinjector. Solvent A: 0.1% formic acid in Optima water; solvent B: 0.1% formic acid in 80% MeCN:Optima water. A gradient of 2–62% mobile phase B from 0–25 min was applied at the 0.2 mL min^{-1} flowrate. Tandem mass spectrometry fragmentation was performed using the following parameters. Collision energy voltages were set on the fly by the mass spectrometer for individual precursor ions (steps at 20, 25, and 30 V). The capillary voltage and the temperature of the heated transfer capillary were set to 4000 V and 300 °C, respectively. Positive ions were generated by electrospray, and the Orbitrap was operated in data-dependent acquisition mode. A survey scan of 100–

1500 *m/z* or 300 – 2500 *m/z* was acquired (resolution = 60,000, with an accumulation target value of 3,000,000 ions). Up to 10 of the most abundant ions (>1.7 × 10^5 ions), with charge state +1 were sequentially isolated and fragmented. An AGC setting of 100,000 was used for MS^2 mode. Ions selected for MS^2 were dynamically excluded for 4 s. Xcalibur (version B.07.01) software was used for data acquisition and processing. Experimentally determined *m/z* values were used with allowable δ = 5 ppm to generate EICs.

Recombinant Expression and Purification of *St*DesD

This method was adapted from the literature.[1] *S. tropica* DesD variant was expressed with an *N*-terminal Histidine tag (6 x His) in *E. coli* C41 (DE3) cells in LB. An aliquot (5 mL) of 10 mL day culture containing 50 µg mL^{-1} kanamycin was used to inoculate a 300 mL culture supplemented with the same concentration of antibiotic. The culture was grown at 180 rpm at 37 °C overnight. An aliquot (15 mL) of the overnight culture was then used to inoculate 6 x 1 L of (LB) media supplemented with 50 µg mL^{-1} kanamycin. The cultures were then grown at 180 rpm at 37 °C until an OD600 value of 0.6 was reached, and the expression was induced through the addition of 1 mM IPTG with overnight expression at 180 rpm at 37 °C. The cells were harvested by centrifugation (6000 rpm for 12 min) and resuspended in lysis buffer (20 mM Tris/HCl pH 8, 100 mM NaCl, 10% glycerol, 100 µM AEBSF, 0.004 mg mL^{-1} DNase, 5 mM $MgCl_2$). The cell pellet was lysed using an Emulsiflex C5 high-pressure homogenizer (Avestin). The insoluble debris was removed by centrifugation (8000 rpm for 30 min).

The supernatant was bound to a pre-equilibrated 5 mL HisTrap FF pre-packed column (Cytiva) and washed with 5 CV of wash buffer 1 (20 mM Tris/HCl pH 8, 100 mM NaCl, 10% glycerol, 25 mM imidazole) and 5 CV of wash buffer 2 (20 mM Tris/HCl pH 8, 100 mM NaCl, 10% glycerol, 40 mM imidazole). The purified protein was eluted from the HisTrap resin with 3 CV of elution buffer (20 mM Tris/HCl pH 8, 100 mM NaCl, 10% glycerol, 250 mM imidazole). The eluted protein was concentrated to a final volume of 4 mL using an Amicon Ultra concentrator (Millipore) (M_r 30 kDa cut-off). The His-tag was cleaved with the addition of 200 µL of TEV protease (1 mg mL^{-1}) and dialyzed overnight at 4 °C against dialysis buffer (20 mM Tris/HCl pH 8, 300 mM NaCl, 10% glycerol). Protein was then purified by gel filtration chromatography using a Superdex 75 Hi-load 16/600 column (GE Healthcare) equilibrated with 20 mM Tris/HCl pH 8, 100 mM NaCl, 10% glycerol. Protein was concentrated to 10 – 15 mg mL^{-1} for functional analysis using an Amicon Ultra concentrator (M_r 30 kDa cut-off). The protein concentration was determined by measuring the absorbance at 280 nm using a Nanodrop 2000C Spectrophotometer (Thermo Fisher Scientific). Protein was stored at -80 °C.

Chemoenzymatic Reactions

These methods were adapted from the literature.[1-3] Substrates (2.4 mM) were incubated in varying combinations with $MgCl_2$ (15 mM), ATP (4 mM), Tris/HCl (145 mM, pH 8), and *St*DesD (1 mg mL^{-1}) in a final volume of 200 µL for 24 h at 37 °C. The reaction was terminated by the addition of 2 µL of 10% formic acid. Samples were then centrifuged at 15,000 rpm for 15 min, and the supernatant liquid was filtered using a PTFE syringe filter (0.45 µm, 5 mm) before analysis. Control experiments were conducted containing no enzyme.

Standard curves were generated by LC-HRMS/MS using solutions of **1**, **2**, **3**, **4**, **5**, and **6** in concentrations of 0.3, 0.5, 1.0, 1.5, 2.0, 2.5 and 3.0 mM in Milli-Q water.

Coordination Chemistry Screening

An aliquot of terminated enzyme incubation (approximately 2 mM substrate) was incubated with either Zr(IV)Cl_4 (10 mM) or Ga(III)$(NO_3)_3$ (6 mM) at 37 °C for 2h. Samples were then centrifuged at 15000 rpm for 15 min, and supernatant liquid was filtered using a PTFE syringe filter (0.45 µm, 5 mm) prior to analysis by LC-HRMS/MS.

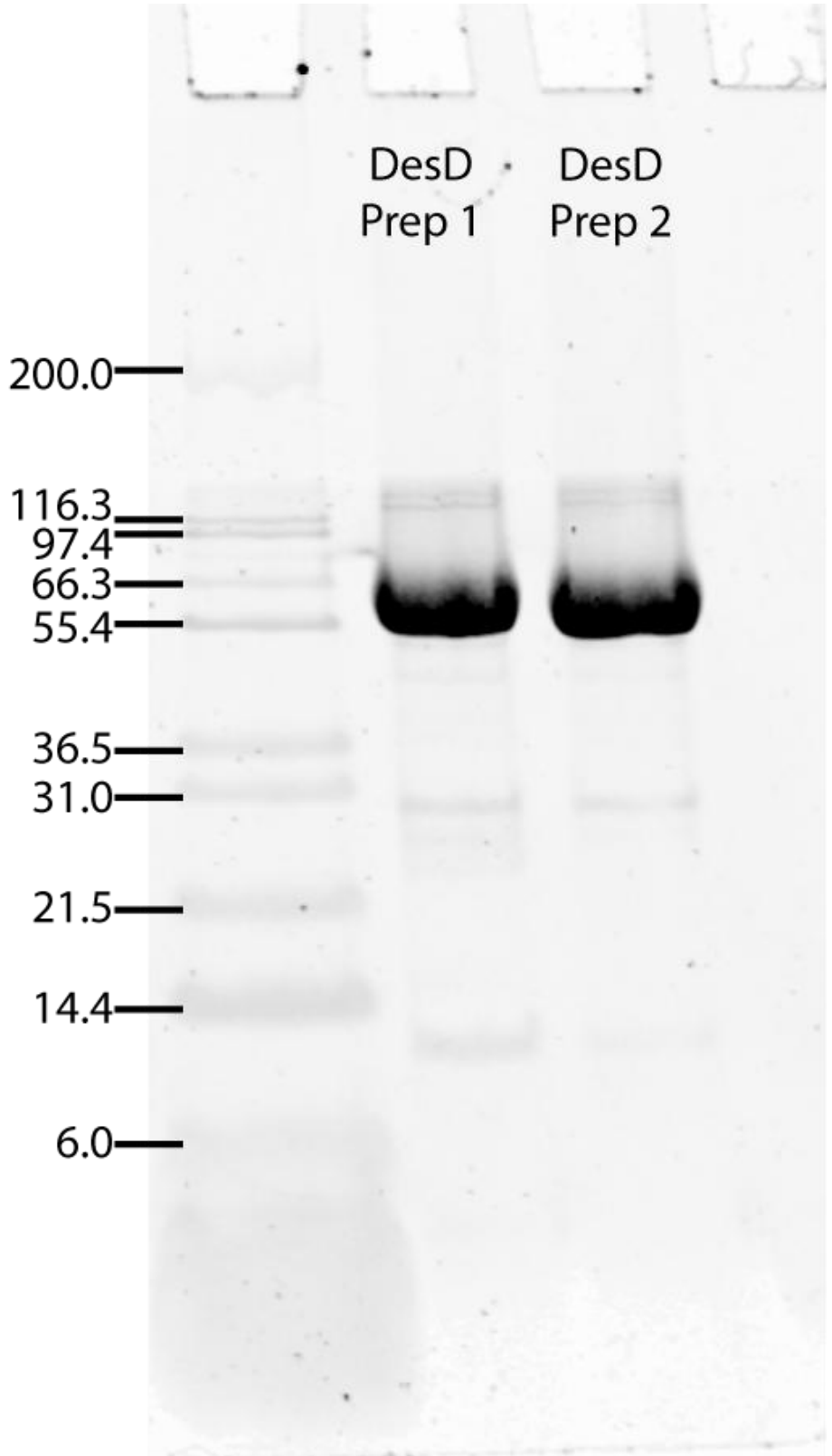

Figure S21. SDS-PAGE gel for *St*DesD (Mark 12 Unstained Standard ladder). Each well contains 15 µL of a 1 mg mL^{-1} solution of StDesD.

Synthetic Procedures

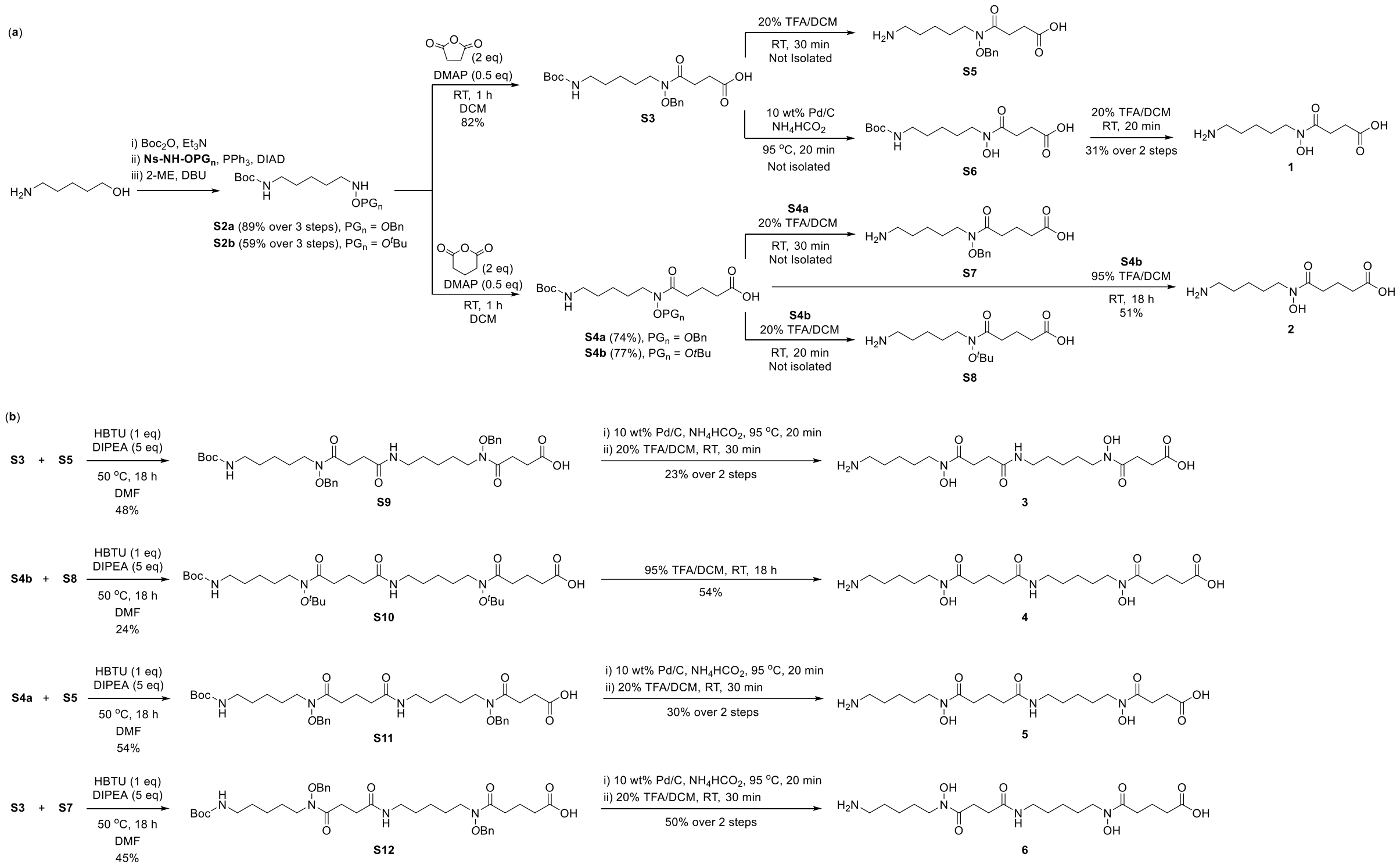

Scheme S6. Chemical synthesis of monomeric (**a**) and dimeric (**b**) substrates for chemoenzymatic reactions with *St*DesD.

General Mitsunobu-Fukuyama protocol

Triphenylphosphine (1.5 eq) and *N*-(benzyloxy)-4-nitrobenzenesulfonamide (1.0 eq) or *N*-(*tert*-butoxy)-4-nitrobenzenesulfonamide (1 eq) were added to a solution of *N*-Boc-5-aminopentan-1-ol **2-1** (1.0 eq) in THF. The mixture was stirred at room temperature for 15 min to ensure complete dissolution. The resulting mixture was cooled to 0 ºC and DIAD (1.3 eq) was added dropwise. Following complete addition, the mixture was stirred at 0 ºC for 30 min. The reaction mixture was then heated at 50 ºC and left to stir overnight. The solvent was then removed *in vacuo* and the crude material was reconstituted with diethyl ether. The resulting mixture was then stirred at 0 ºC over an ice bath and the resulting precipitate was removed by filtration. The solvent was removed *in vacuo* and the crude material was semi-purified by auto flash chromatography (Reveleris; 1.5 CV: 9% EtOAc in hex, 16 CV: 9-28% EtOAc in hex, 3 CV: 28% EtOAc in hex) yielding the (5-((*N*-(benzyloxy)-4-nitrophenyl)sulfonamido)pentyl)carbamate derivative.

The semi-purified *tert*-butyl (5-((*N*-4-nitrophenyl)sulfonamido)pentyl)carbamate or *tert*-butyl (5-((N-(tert-butoxy)-4-nitrophenyl)sulfonamido)pentyl)carbamate (1.0 eq) was dissolved in DMF and 2-mercaptoethanol (1.0 eq) and DBU (1.0 eq) were added sequentially. The resulting mixture was stirred at room temperature overnight. The solvent was removed *in vacuo* and the crude material was reconstituted in DCM and washed with H_2O (5 x 100 mL). The DCM fraction was dried over $MgSO_4$ and the solvent was removed *in vacuo* and the crude material purified by auto-flash chromatography.

General anhydride acylation protocol

DMAP (0.5 eq) and the appropriate anhydride (2.0 eq) were added to a solution of *tert*-butyl (5-((benzyloxy)amino)pentyl)carbamate **2-4$_1$** (1.0 eq) or *tert*-butyl (5-(*tert*-butoxyamino)pentyl)carbamate **2-4$_2$** (1.0 eq) in DCM. The resulting mixture was allowed to stir at room temperature for 1 h, before being diluted with DCM and washed with hydrochloric acid (1 M). The DCM fraction was then dried over $MgSO_4$ and the solvent was removed *in vacuo*. The crude material was purified by auto-flash chromatography (Biotage; 2 CV: 35% EtOAc in hex, 15 CV: 35-65% EtOAc in hex, 6 CV: 65% EtOAc in hex) to yield the desired protected monomeric intermediate.

General amide coupling protocol
HBTU (1.0 eq) and DIPEA (5.0 eq) were added to a solution of *N*-Boc protected monomer (1.0 eq) in anhydrous DMF. The resulting mixture was allowed to stir at room temperature for 1 hour, before a solution of free amine monomer (1.1 eq) in anhydrous DMF was added. The resulting mixture was heated at 50 °C overnight, the solvent was then removed *in vacuo* and the crude material was reconstituted in DCM and washed with saturated sodium bicarbonate (5 x 50 mL). The DCM fraction was washed with brine and dried over $MgSO_4$. The solvent was removed *in vacuo* and the crude material was purified by preparative HPLC and lyophilized to give the protected dimeric intermediate.

General *N*-Boc deprotection protocol
N-Boc protected intermediate was stirred in TFA/DCM (20% v/v) for 30 min. The solvent was removed *in vacuo* and the residue reconstituted, and the solvent removed *in vacuo* in EtOAc (3 x 10 mL) followed by toluene (3 x 10 mL) to give the *N*-Boc deprotected intermediate that was used without further purification, unless otherwise stated.

General O^tBu deprotection protocol
O^tBu protected intermediate was stirred in TFA/DCM (95% v/v) overnight. The solvent was removed *in vacuo* and the residue reconstituted, and the solvent removed *in vacuo* in EtOAc (3 x 10 mL) followed by toluene (3 x 10 mL). Preparative HPLC purification and lyophilization was then performed to yield the O^tBu deprotected intermediate.

General catalytic transfer hydrogenation protocol
*O*Bn protected intermediate (1 eq) was dissolved in ethanol and added to a suspension of Pd/C (10 wt%) in ethanol. Ammonium formate (20 eq) was then added to the mixture and was refluxed under an N_2 atmosphere for 15 min. The reaction mixture was cooled and filtered over Celite. The solvent was removed *in vacuo* and the crude material was purified by preparative HPLC and lyophilized, unless otherwise stated, to yield the *O*Bn deprotected intermediate.

tert-butyl (5-hydroxypentyl)carbamate (**S1**)[4]

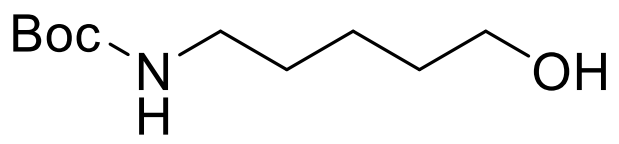

5-aminopentan-1-ol (15.0 g, 145 mmol.) was dissolved in dichloromethane (50 mL). Di-*tert*-butyl dicarbonate (31.7 g, 145 mmol.) and triethylamine (14.2 mL, 102 mmol.) were then added and the resulting solution was left to stir at room temperature for 3 h. The solvent was removed *in vacuo* to yield *N*-Boc-5-aminopentan-1-ol **S1** (29.5 g, 99%) as a colourless oil without the need for further purification.

^{1}H NMR (600 MHz, $CDCl_3$) δ 4.57 (1H, brs), 3.63 (2H, dd, *J* = 6.4, 11.9 Hz), 3.12 (2H, dd, *J* = 5.9, 12.2 Hz), 1.58 (2H, m), 1.50 (2H, m), 1.43 (9H, brs), 1.37 (2H, m). ^{13}C (150 MHz, $CDCl_3$) δ: 156.0, 79.1, 62.6, 40.4, 32.2, 29.8, 28.4, 22.9. HRMS calculated for $C_{10}H_{21}NO_3$ $[M+H]^+$: 204.1560, observed 204.1596.

N-(benzyloxy)-2-nitrobenzenesulfonamide (**Ns-NH-*O*Bn**)[4]

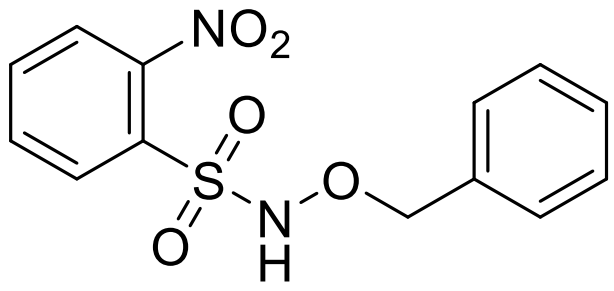

A solution of 2-nitrobenzylsulfonyl chloride (28.1 g, 127 mmol.) in pyridine (50 mL) was added dropwise to a stirring solution of *O*-benzylhydroxylamine hydrochloride (20.2 g, 127 mmol.) in pyridine (100 mL) at 0 °C. Following complete addition, the reaction mixture was stirred at 0 °C for 30 min before being warmed to room temperature and allowed to stir overnight. The reaction was quenched with water (60 mL) and the mixture was concentrated *in vacuo*. The resulting aqueous mixture was further diluted with water (200 mL) and extracted with EtOAc (100 mL). The EtOAc fraction was then

sequentially washed with hydrochloric acid (1 M), H_2O, and sat. sodium bicarbonate (100 mL each). The EtOAc fraction was dried over $MgSO_4$ and the solvent removed *in vacuo* to give *N*-(benzyloxy)-2-nitrobenzenesulfonamide (30.5 g, 78%) as a brown-yellow solid that was used without further purification.

N-(tert-butoxy)-2-nitrobenzenesulfonamide (**Ns-NH-*O*^{t}Bu**)[5]

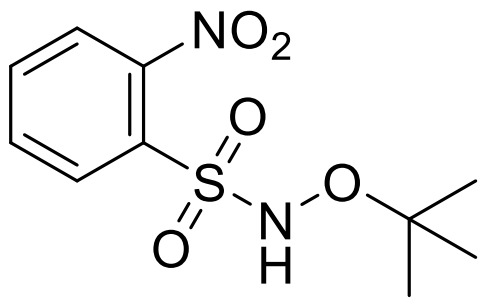

Triethylamine (39.6 mL, 391 mmol.) was added dropwise to a stirring solution of *O*-*tert*-butylhydroxylamine hydrochloride (24.6 g, 196 mmol.) in chloroform (100 mL) at 0 ºC. A solution of 2-nitrobenzylsulfonyl chloride (43.3 g, 195 mmol.) in chloroform (50 mL) was then added dropwise and the resulting reaction mixture was stirred at 0 ºC for 2 h before being warmed to room temperature and allowed to stir overnight. The reaction was then diluted with chloroform (50 mL) and was then sequentially washed with H_2O, hydrochloric acid (1M), H_2O, sodium bicarbonate (5% v/v), and H_2O (100 mL each). The chloroform fraction was dried over $MgSO_4$ and the solvent removed *in vacuo* to give *N*-(*tert*-butoxy)-2-nitrobenzenesulfonamide (33.5 g, 62%) as a white solid that was used without further purification.

tert-butyl (5-((benzyloxy)amino)pentyl)carbamate (**S2a**)[4]

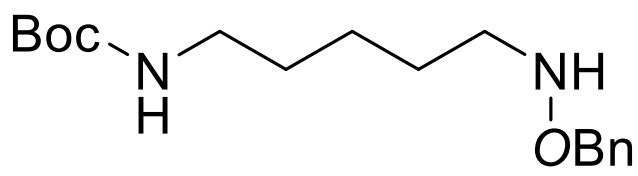

tert-butyl (5-((benzyloxy)amino)pentyl)carbamate **S2a** was prepared using the general Mitsunobu-Fukuyama procedure using triphenylphosphine (59.1 g, 225 mmol.), *N*-(benzyloxy)-2-nitrobenzenesulfonamide (45.0 g, 146 mmol.), DIAD (38.0 mL, 194 mmol.), and *N*-Boc-5-aminopentan-1-ol **S1** (29.6 g, 146 mmol.), 2-mercaptoethanol (9.27 mL, 132 mmol.), and DBU (19.7 mL, 132 mmol.). *tert*-butyl (5-((benzyloxy)amino)pentyl)carbamate **S2a** was purified by flash column chromatography (Biotage; 1 CV: 15% EtOAc in hex, 25 CV: 15-40% EtOAc in hex, 10 CV: 40% EtOAc in hex.) and was obtained as a yellow oil (32.1 g, 89%).

^{1}H NMR (600 MHz, $CDCl_3$) δ 7.35–7.28 (5H, m), 5.52 (1H, brs), 4.69 (2H, s), 3.10 (2H, m), 2.92 (2H, t, *J* = 7.1 Hz), 1.53 (2H, m), 1.46 (2H, m), 1.43 (9H, s), 1.33 (2H, m). ^{13}C (150 MHz, $CDCl_3$) δ: 156.0, 138.0, 128.4, 127.8, 76.2, 52.0, 40.5, 30.0, 28.4, 27.1, 24.4, 22.0. HRMS calculated for $C_{17}H_{28}N_2O_3$ $[M+H]^+$: 309.2178, observed 309.2168.

tert-butyl (5-(tert-butoxyamino)pentyl)carbamate (**S2b**)[5]

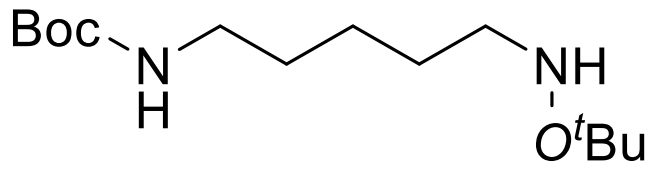

tert-butyl (5-(*tert*-butoxyamino)pentyl)carbamate **S2b** was prepared using the general Mitsunobu-Fukuyama procedure using triphenylphosphine (51.7 g, 197 mmol.), *N*-(*tert*-butoxy)-2-nitrobenzenesulfonamide (34.5 g, 126 mmol.), DIAD (32.0 mL, 164 mmol.), *N*-Boc-5-aminopentan-1-ol **S1** (29.6 g, 126 mmol.), 2-mercaptoethanol (8.86 mL, 126 mmol.), and DBU (18.8 mL, 126 mmol.). *tert*-butyl (5-(*tert*-butoxyamino)pentyl)carbamate **S2b** was purified using flash column chromatography (Biotage: 5 CV: 10% EtOAc in hex, 15 CV: 10-23% EtOAc in hex, 6 CV: 23% EtOAc in hex.) and was obtained as a yellow oil (20.5 g, 60%).

^{1}H NMR (600 MHz, $CDCl_3$) δ 3.00 (2H, m), 2.78 (2H, t, *J* = 7.1 Hz), 1.41 (4H, m), 1.38 (9H, s), 1.30 (2H, m), 1.11 (9H, s). ^{13}C (150 MHz, $CDCl_3$) δ 156.0, 79.0, 76.7, 52.9, 40.5, 30.0, 28.5, 27.0, 26.8, 24.6. HRMS calculated for $C_{14}H_{30}N_2O_3$ $[M+H]^+$: 275.2335, observed 275.2329.

4-((benzyloxy)(5-((tert-butoxycarbonyl)amino)pentyl)amino)-4-oxobutanoic acid (**S3**)

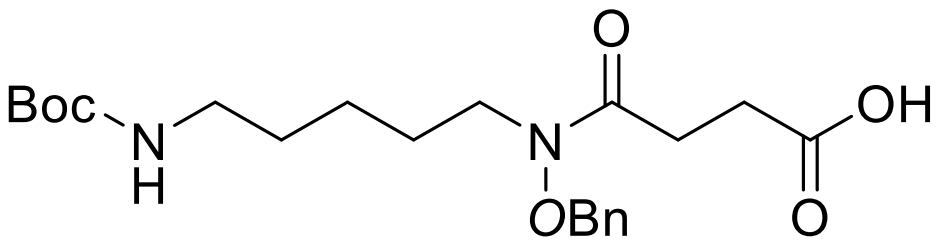

4-((benzyloxy)(5-((*tert*-butoxycarbonyl)amino)pentyl)amino)-4-oxobutanoic acid **S3** was prepared using the general acylation method using DMAP (534 mg, 4.37 mmol.), succinic anhydride (1.75 g, 17.5 mmol.), and *tert*-butyl (5-((benzyloxy)amino)pentyl)carbamate **S2a** (2.70 g, 8.75 mmol.). 4-((benzyloxy)(5-((*tert*-butoxycarbonyl)amino)pentyl)amino)-4-oxobutanoic acid **S3** was purified by flash column chromatography and was isolated as a yellow-white solid (2.93 g, 82%).

^{1}H NMR (600 MHz, DMSO-*d*$_6$) δ 7.44–7.37 (5H, m) 6.74 (1H, t, *J* = 5.4 Hz), 4.88 (2H, s), 3.56 (2H, t, *J* = 6.9 Hz), 2.87 (2H, dd, *J* = 6.7, 12.9 Hz), 2.62 (2H, m), 2.42 (2H, t, *J* = 6.9 Hz), 1.51 (2H, m), 1.36 (9H, s), 1.33 (2H, m), 1.18 (2H, m). ^{13}C (150 MHz, DMSO-*d*$_6$) δ 173.8, 155.6, 134.9, 129.3, 128.7, 128.5, 77.3, 75.4, 29.1, 28.3, 28.3, 26.8, 26.1, 23.4. Note: Overlapping signals at 28.3. HRMS calculated for $C_{18}H_{34}N_2O_6$ [M+H]$^+$: 409.2333, observed 409.2330.

5-(tert-butoxy(5-((tert-butoxycarbonyl)amino)pentyl)amino)-5-oxopentanoic acid (**S4a**)

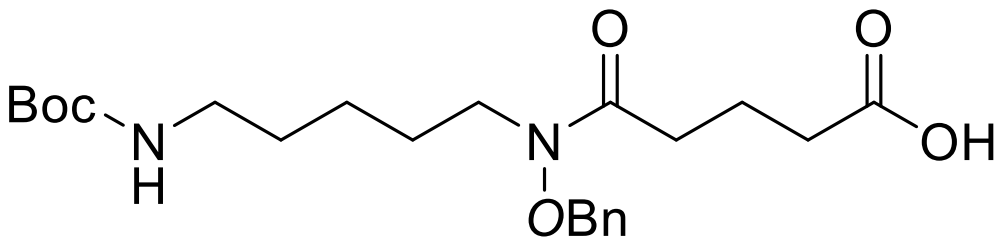

5-(*tert*-butoxy(5-((*tert*-butoxycarbonyl)amino)pentyl)amino)-5-oxopentanoic acid **S4a** was prepared using the general anhydride acylation procedure using DMAP (309 mg, 2.53 mmol.), glutaric anhydride (1.16 g, 10.1 mmol.), and *tert*-butyl (5-(*tert*-butoxyamino)pentyl)carbamate **S2a** (1.39 g, 5.06 mmol.). 5-(*tert*-butoxy(5-((*tert*-butoxycarbonyl)amino)pentyl)amino)-5-oxopentanoic acid **S4a** was purified by flash column chromatography and was isolated as a colourless oil (1.52 g, 77%).

^{1}H NMR (600 MHz, $CDCl_3$) δ 7.39 (5H, m), 4.78 (2H, s), 4.64 (1H, s), 3.63 (2H, s), 3.07 (2H, m), 2.47 (2H, t, *J* = 6.7 Hz), 2.37 (2H, t, *J* = 7.0 Hz), 1.91 (2H, m), 1.62 (2H, m), 1.44 (11H, m), 1.28 (2H, m). ^{13}C (150 MHz, $CDCl_3$) δ 177.9, 174.3, 156.2, 132.5, 129.3, 129.1, 128.8, 76.4, 45.2, 41.5, 40.5, 33.3, 31.3, 29.6, 28.5, 26.6, 23.9, 19.7. HRMS calculated for $C_{22}H_{34}N_2O_6$ [M+H]$^+$: 423.2495, observed 423.2490.

5-((benzyloxy)(5-((tert-butoxycarbonyl)amino)pentyl)amino)-5-oxopentanoic acid (**S4b**)[5]

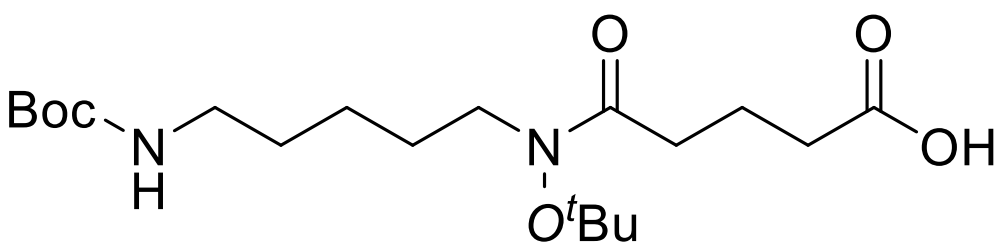

5-((benzyloxy)(5-((*tert*-butoxycarbonyl)amino)pentyl)amino)-5-oxopentanoic acid **S4b** was prepared using the general acylation method using DMAP (171 mg, 1.40 mmol.), glutaric anhydride (727 mg, 5.04 mmol.), and *tert*-butyl (5-((benzyloxy)amino)pentyl)carbamate **S2b** (669 mg, 2.44 mmol.). 5-((benzyloxy)(5-((*tert*-butoxycarbonyl)amino)pentyl)amino)-5-oxopentanoic acid **S4b** was purified by flash column chromatography and was isolated as a colourless oil (699 mg, 74%).

^{1}H NMR (600 MHz, $CDCl_3$) δ 4.66 (1H, s), 3.07 (2H, m), 2.50 (2H, brs), 2.38 (2H, t, *J* = 7.1 Hz), 1.91 (2H, quin, *J* = 7.2 Hz), 1.43 (11H, m), 1.27 (9H, s), 1.23 (2H, m). ^{13}C (150 MHz, $CDCl_3$) δ 177.8, 171.4, 156.2, 82.8, 79.3, 49.7, 40.5, 33.3, 30.1, 29.7, 28.5, 27.9, 26.0, 24.0, 19.9. HRMS calculated for $C_{19}H_{36}N_2O_6$ [M+H]$^+$: 389.2649, observed 389.2652.

4-((5-aminopentyl)(hydroxy)amino)-4-oxobutanoic acid (**1**)[6]

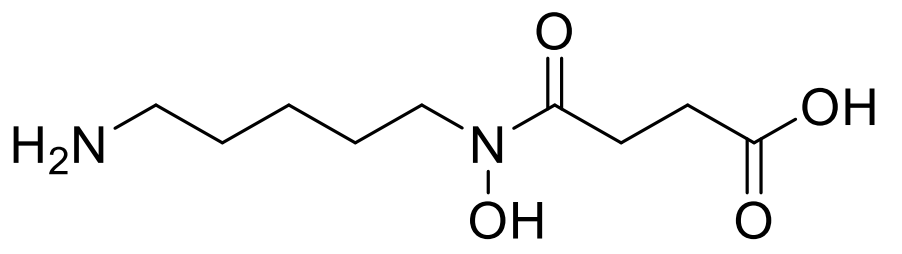

4-((5-aminopentyl)(benzyloxy)amino)-4-oxobutanoic acid **S6** was prepared using the general transfer hydrogenation procedure using Pd/C (60.2 mg, 10 wt%), ammonium formate (2.64 g, 41.8 mmol.), and 4-((benzyloxy)(5-((*tert*-butoxycarbonyl)amino)pentyl)amino)-4-oxobutanoic acid **S3** (569 mg, 1.39 mmol.). 4-((5-aminopentyl)(benzyloxy)amino)-4-oxobutanoic acid **S6** was isolated as a colourless oil and was used without further purification. 4-((5-aminopentyl)(hydroxy)amino)-4-oxobutanoic acid **1** was then prepared using the general *N*-Boc deprotection method using TFA/DCM (20% v/v, 5 mL) and 4-((5-aminopentyl)(benzyloxy)amino)-4-oxobutanoic acid (443 mg, 1.39 mmol.). 4-((5-aminopentyl)(hydroxy)amino)-4-oxobutanoic acid **1** was purified by preparative HPLC (0-10% solvent B over 20 min, product eluted at 7.2 min), lyophilized and was isolated as a yellow oil (94 mg, 31%).

^{1}H NMR (600 MHz, D_2O) δ 3.65 (2H, t, *J* = 4.8 Hz), 2.99 (2H, m), 2.76 (2H, t, *J* = 6.8 Hz), 2.54 (2H, m), 1.68 (4H, m), 1.36 (2H, m). ^{13}C (150 MHz, D_2O) δ 179.8, 174.6, 47.4, 39.3, 31.0, 30.8, 27.7, 26.2, 25.2, 22.5. HRMS calculated for $C_9H_{18}N_2O_4$ $[M+H]^+$: 219.1345, observed 219.1337.

5-((5-aminopentyl)(hydroxy)amino)-5-oxopentanoic acid (**2**)

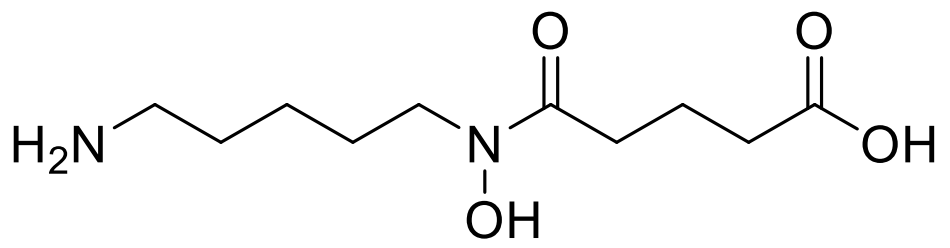

5-((5-aminopentyl)(hydroxy)amino)-5-oxopentanoic acid **2** was prepared using the general O^tBu deprotection method using TFA/DCM (95% v/v, 10 mL) and 5-(*tert*-butoxy(5-((*tert*-butoxycarbonyl)amino)pentyl)amino)-5-oxopentanoic acid **S4b** (1.52 g, 3.35 mmol.). 5-((5-aminopentyl)(hydroxy)amino)-5-oxopentanoic acid **2** was purified by preparative HPLC (0-12% solvent B over 30 min, product eluted at 11.7 min), lyophilized and was isolated as a colourless oil (401 mg, 51%).

^{1}H NMR (600 MHz, D_2O) δ 3.67 (2H, t, *J* = 6.9 Hz), 3.01 (2H, m), 2.58 (2H, t, *J* = 7.5 Hz), 2.45 (2H, m), 1.89 (2H, m), 1.69 (2H, m), 1.38 (2H, m). ^{13}C (150 MHz, D_2O) δ 178.0, 175.1. 50.5, 47.6, 39.3, 33.1, 30.9, 26.3, 25.2, 22.6, 19.7. HRMS calculated for $C_{10}H_{20}N_2O_4$ $[M+H]^+$: 233.1501, observed: 233.1492.

11,22-bis(benzyloxy)-2,2-dimethyl-4,12,15,23-tetraoxo-3-oxa-5,11,16,22-tetraazahexacosan-26-oic acid (**S9**)[7]

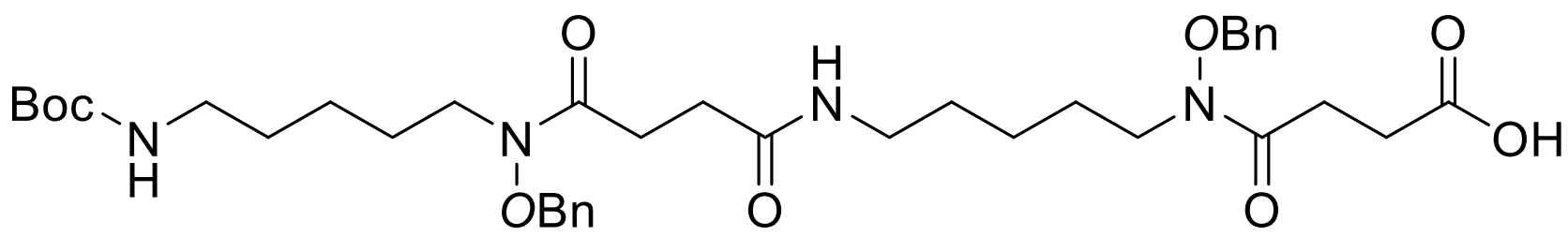

4-((5-aminopentyl)(benzyloxy)amino)-4-oxobutanoic acid **S5** was prepared using the general *N*-Boc deprotection procedure using TFA/DCM (20% v/v, 5 mL) and 4-((benzyloxy)(5-((*tert*-butoxycarbonyl)amino)pentyl)amino)-4-oxobutanoic acid **S3** (203 mg, 0.639 mmol.). 4-((5-aminopentyl)(benzyloxy)amino)-4-oxobutanoic acid **S5** was isolated as a colourless oil and was used without further purification.

11,22-bis(benzyloxy)-2,2-dimethyl-4,12,15,23-tetraoxo-3-oxa-5,11,16,22-tetraazahexacosan-26-oic acid **S9** was prepared using the general amide coupling procedure using DIPEA (1.81 mL, 10.4 mmol.), HBTU (785 mg, 2.08 mmol.), 4-((benzyloxy)(5-((*tert*-butoxycarbonyl)amino)pentyl)amino)-4-oxobutanoic acid **S3** (850 mg, 2.08 mmol.), and 4-((5-aminopentyl)(benzyloxy)amino)-4-oxobutanoic acid **S5** (706 mg, 2.29 mmol.). 11,22-bis(benzyloxy)-2,2-dimethyl-4,12,15,23-tetraoxo-3-oxa-

5,11,16,22-tetraazahexacosan-26-oic acid **S9** was purified by preparative HPLC (50-59% solvent B over 12 min, product eluted at 9.3 min), lyophilized and was isolated as a yellow oil (657 mg, 45%).

^{1}H NMR (600 MHz, DMSO-d_6) δ 7.76 (1H, t, *J* = 5.5 Hz), 7.40 (10H, m), 6.73 (1H, t, *J* = 5.4 Hz), 4.87 (4H, m), 3.54 (4H, dd, *J* = 7.2, 17.5 Hz), 2.99 (2H, dd, *J* = 6.8, 12.8 Hz), 2.87 (2H, dd, *J* = 6.7, 12.9 Hz), 2.62 (4H, bs), 2.42 (2H, t, *J* = 6.5 Hz), 2.30 (2H, t, *J* = 7.1 Hz), 1.50 (4H, m), 1.36 (13H, m), 1.20 (4H, m). ^{13}C (150 MHz, DMSO-d_6) δ 173.8, 172.9, 172.6, 171.0, 155.6, 134.9, 129.3, 129.3, 128.7, 128.5, 128.5, 77.3, 75.4, 44.5, 40.1, 38.4, 29.7, 28.8, 28.4, 28.3, 27.2, 26.9, 26.2, 23.5, 23.4. Note: Overlapping signals at 129.3. HRMS calculated for $C_{37}H_{54}N_4O_9$ $[M+H]^+$: 699.3969, observed: 699.3952.

4-((5-(4-((5-aminopentyl)(hydroxy)amino)-4-oxobutanamido)pentyl)(hydroxy)amino)-4-oxobutanoic acid (**3**)[7]

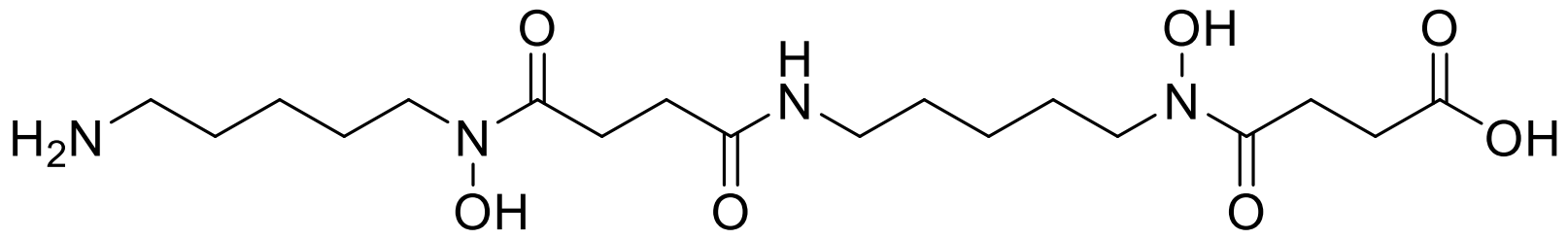

4-((5-(4-((5-aminopentyl)(hydroxy)amino)-4-oxobutanamido)pentyl)(hydroxy)amino)-4-oxobutanoic acid **3** was prepared using the general transfer hydrogenation procedure using Pd/C (40.2 mg, 10 wt%), ammonium formate (773 mg, 11.5 mmol.), and 11,22-bis(benzyloxy)-2,2-dimethyl-4,12,15,23-tetraoxo-3-oxa-5,11,16,22-tetraazahexacosan-26-oic acid **S9** (402 mg, 0.575 mmol.) followed immediately by the general *N*-Boc deprotection procedure using TFA/DCM (20% v/v, 5 mL). 4-((5-(4-((5-aminopentyl)(hydroxy)amino)-4-oxobutanamido)pentyl)(hydroxy)amino)-4-oxobutanoic acid **3** was then purified by preparative HPLC (0-15% solvent B over 25 min, product eluted at 13.7 min), lyophilized, and was isolated as a white solid (53.9 mg, 23%).

^{1}H NMR (600 MHz, D_2O) δ 3.62 (4H, dd, *J* = 7.1, 14.3 Hz), 3.16 (2H, t, *J* = 6.7 Hz), 2.97 (2H, t, 7.6 Hz), 2.79 (2H, t, *J* = 6.9 Hz), 2.71 (2H, t, *J* = 7.3 Hz), 2.48 (2H, t, *J* = 7.0 Hz), 2.44 (2H, t, *J* = 7.4 Hz), 1.65 (6H, m), 1.50 (2H, m), 1.35 (2H, m), 1.28 (2H, m). ^{13}C (150 MHz, D_2O) δ 181.2, 174.8, 174.8, 173.9, 47.7 47.5, 39.3, 39.1, 32.2, 30.3, 28.4, 27.8, 27.5, 26.2, 25.4, 25.2, 22.9, 22.6. Note: Overlapping signals at 174.8 due to *E/Z* isomers about the hydroxamic acid in D_2O. HRMS calculated for $C_{18}H_{34}N_4O_7$ $[M+H]^+$: 419.2505, observed: 419.2497

11,23-di-tert-butoxy-2,2-dimethyl-4,12,16,24-tetraoxo-3-oxa-5,11,17,23-tetraazaoctacosan-28-oic acid (**S10**)

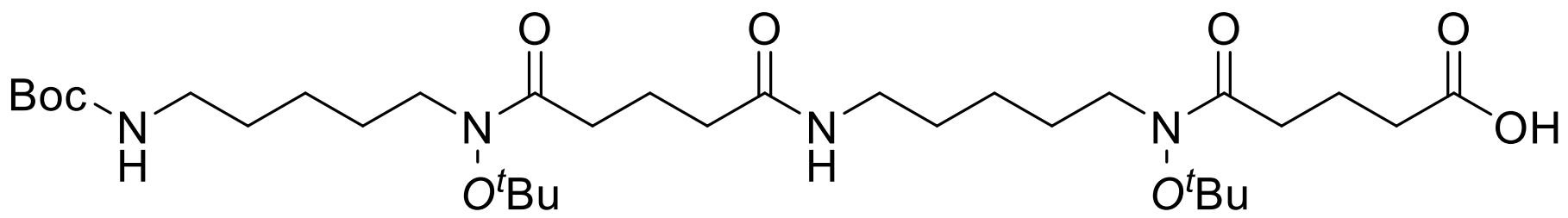

5-((5-aminopentyl)(*tert*-butoxy)amino)-5-oxopentanoic acid **S8** was generated using the general *N*-Boc deprotection procedure using TFA/DCM (20% v/v, 5 mL), and 5-(*tert*-butoxy(5-((*tert*-butoxycarbonyl)amino)pentyl)amino)-5-oxopentanoic acid **S4b** (350 mg, 0.901 mmol.). 5-((5-aminopentyl)(*tert*-butoxy)amino)-5-oxopentanoic acid **S8** was isolated as a colourless oil and was used without further purification.

11,23-di-*tert*-butoxy-2,2-dimethyl-4,12,16,24-tetraoxo-3-oxa-5,11,17,23-tetraazaoctacosan-28-oic acid **S10** was prepared using the general amide coupling procedure using DIPEA (0.588 mL, 3.38 mmol.), HBTU (271 mg, 0.716 mmol.), 5-((5-aminopentyl)(*tert*-butoxy)amino)-5-oxopentanoic acid **S4b** (214 mg, 0.743 mmol.), and 5-(*tert*-butoxy(5-((*tert*-butoxycarbonyl)amino)pentyl)amino)-5-oxopentanoic acid **S8** (262 mg, 0.675 mmol.). 11,23-di-tert-butoxy-2,2-dimethyl-4,12,16,24-tetraoxo-3-oxa-5,11,17,23-tetraazaoctacosan-28-oic acid **S10** was purified by preparative HPLC (50-62% solvent B over 12 min, product eluted at 6.7 min), lyophilized and was isolated as a colourless oil (108 mg, 24%).

^{1}H NMR (600 MHz, DMSO-*d₆*) δ 7.73 (1H, t, *J* = 5.6 Hz), 6.74 (1H, t, *J* = 5.5 Hz), 2.98 (2H, dd, *J* = 6.8, 12.8 Hz), 2.87 (2H, dd, *J* = 6.8 Hz, 12.9 Hz), 2.40 (2H, m), 2.34 (2H, m), 2.22 (2H, t, *J* = 7.3 Hz), 2.05 (2H, t, *J* = 7.2 Hz), 1.68 (4H, m), 1.51 (4H, brs), 1.36 (13H, m), 1.23 (18H, m), 1,16 (4H, m). ^{13}C (150 MHz, DMSO-d_6) δ 174.2, 171.5, 163.0, 155.6, 81.9, 81.8, 77.3, 38.3, 34.6, 32.9, 31.7, 31.5, 29.1, 28.8, 28.3, 27.3, 25.6, 23.6, 23.5, 20.6, 19.9. HRMS calculated for $C_{33}H_{62}N_4O_9$ $[M+H]^+$: 659.4595, observed: 659.4583.

5-((5-(5-((5-aminopentyl)(hydroxy)amino)-5-oxopentanamido)pentyl)(hydroxy)amino)-5-oxopentanoic acid (**4**)

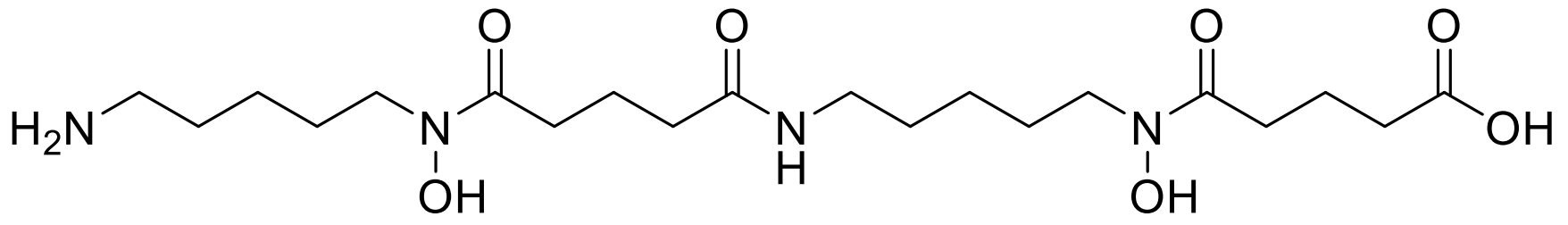

5-((5-(5-((5-aminopentyl)(hydroxy)amino)-5-oxopentanamido)pentyl)(hydroxy)amino)-5-oxopentanoic acid **4** was prepared using the general *O*^{t}Bu deprotection procedure using TFA/DCM (95% v/v, 5 mL), and 11,23-di-*tert*-butoxy-2,2-dimethyl-4,12,16,24-tetraoxo-3-oxa-5,11,17,23-tetraazaoctacosan-28-oic acid **S10** (108 mg, 0.164 mmol.). 5-((5-(5-((5-aminopentyl)(hydroxy)amino)-5-oxopentanamido)pentyl)(hydroxy)amino)-5-oxopentanoic acid **4** was purified by preparative HPLC (0-10% solvent B over 15 min, product eluted at 12.7 min), lyophilized and was isolated as a white solid (36 mg, 54%).

^{1}H NMR (600 MHz, DMSO-d_6) δ 3.64 (4H, m), 3.18 (2H, t, *J* = 6.7 Hz), 2.99 (2H, t, *J* = 7.5 Hz), 2.56 (2H, t, *J* = 7.7 Hz), 2.52 (2H, t, *J* = 7.9 Hz), 2.28 (2H, t, *J* = 7.1 Hz), 1.87 (4H, m), 1.69 (6H, m), 1.53 (2H, m), 1.37 (2H, m), 1.30 (2H, m). ^{13}C (150 MHz, D_2O) δ 178.2, 175.7, 175.0, 47.7, 47.6, 39.3, 39.1, 25.1, 33.2, 30.9, 27.8, 26.3, 25.4, 25.2, 23.0, 22.6, 21.0, 19.9. HRMS calculated for $C_{20}H_{38}N_4O_7$ $[M+H]^+$: 447.2819, observed: 447.2813.

11,23-bis(benzyloxy)-2,2-dimethyl-4,12,16,24-tetraoxo-3-oxa-5,11,17,23-tetraazaheptacosan-27-oic acid (**S11**)

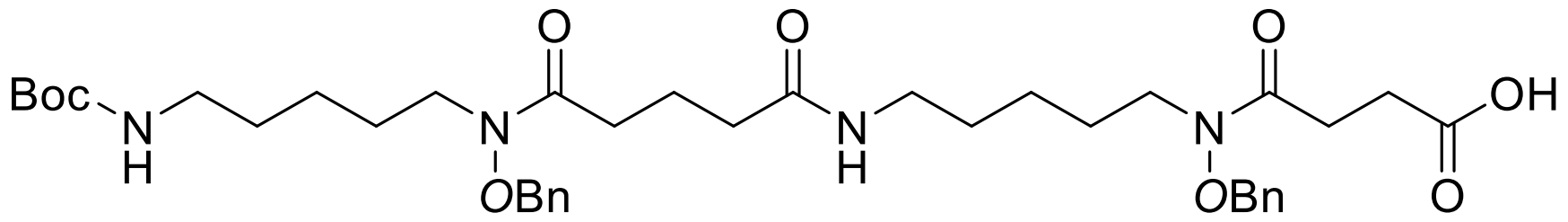

4-((5-aminopentyl)(benzyloxy)amino)-4-oxobutanoic acid **S5** was prepared using the general *N*-Boc deprotection procedure using TFA/DCM (20% v/v, 5 mL) and 4-((benzyloxy)(5-((*tert*-butoxycarbonyl)amino)pentyl)amino)-4-oxobutanoic acid **S3** (203 mg, 0.639 mmol.). 4-((5-aminopentyl)(benzyloxy)amino)-4-oxobutanoic acid **S5** was isolated as a colourless oil and was used without further purification.

11,23-bis(benzyloxy)-2,2-dimethyl-4,12,16,24-tetraoxo-3-oxa-5,11,17,23-tetraazaheptacosan-27-oic acid **S11** was prepared using the general amide coupling procedure using DIPEA (0.790 mL, 4.53 mmol.), HBTU (345 mg, 0.907 mmol.), 4-((5-aminopentyl)(benzyloxy)amino)-4-oxobutanoic acid **S4a** (308 mg, 0.998 mmol.), and 5-((benzyloxy)(5-((*tert*-butoxycarbonyl)amino)pentyl)amino)-5-oxopentanoic acid **S5** (383 mg, 0.907 mmol.). 11,23-bis(benzyloxy)-2,2-dimethyl-4,12,16,24-tetraoxo-3-oxa-5,11,17,23-tetraazaheptacosan-27-oic acid **S11** was purified by preparative HPLC (50-65% solvent B over 12, product eluted at 8.6 min), lyophilized, and was isolated as a yellow oil (360 mg, 54%).

^{1}H NMR (600 MHz, DMSO-d_6) δ 7.73 (1H, t, *J* = 5.4 Hz), 7.40 (10H, m), 6.75 (1H, t, 5.3 Hz), 4.87 (2H, s), 4.82 (2H, s), 3.55 (4H, m), 2.98 (2H, dd, *J* = 6.1, 12.7 Hz), 2.87 (2H, dd, *J* = 6.3, 12.7 Hz), 2.63 (2H, bs), 2.42 (2H, t, *J* = 6.5 Hz), 2.36 (2H, t, *J* = 6.3 Hz), 2.05 (2H, t, *J* = 7.2 Hz), 1.68 (2H, m), 1.49 (4H, m), 1.36 (13H, m), 1.19 (4H, m). ^{13}C (150 MHz, DMSO-d_6) δ 173.8, 173.1, 172.5, 171.6, 156.6,

134.9, 129.4, 129.3, 128.7, 128.5, 128.5, 77.3, 75.4, 40.0, 38.3, 34.6, 30.9, 29.1, 28.8, 28.3, 28.2, 26.9, 26.1, 23.5, 23.4, 20.4. HRMS calculated for $C_{38}H_{56}N_4O_9$ [M+H]$^+$: 713.4126, observed: 713.4108.

4-((5-(5-((5-aminopentyl)(hydroxy)amino)-5-oxopentanamido)pentyl)(hydroxy)amino)-4-oxobutanoic acid (**5**)

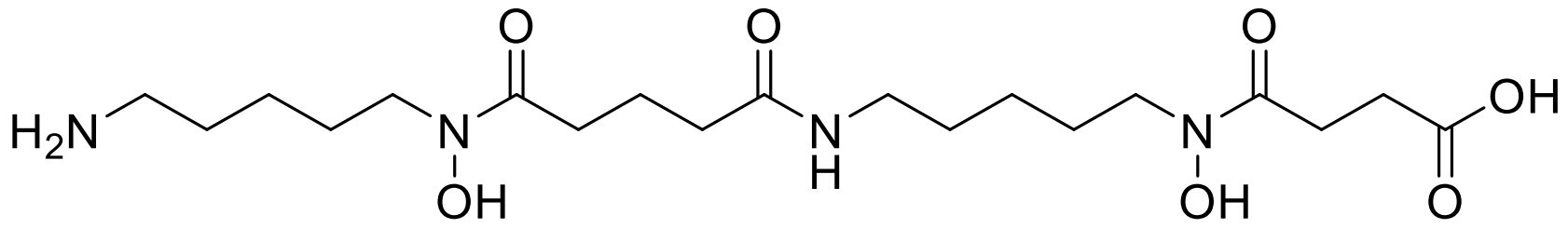

4-((5-(5-((5-aminopentyl)(hydroxy)amino)-5-oxopentanamido)pentyl)(hydroxy)amino)-4-oxobutanoic acid **5** was prepared using the general transfer hydrogenation method using Pd/C (36 mg, 10 wt%), ammonium formation (637 mg, 10.1 mmol.), and 11,23-bis(benzyloxy)-2,2-dimethyl-4,12,16,24-tetraoxo-3-oxa-5,11,17,23-tetraazaheptacosan-27-oic acid **S11** (360 mg, 0.506 mmol.) followed immediately by the general *N*-Boc deprotection procedure using TFA/DCM (20% v/v, 5 mL). 4-((5-(5-((5-aminopentyl)(hydroxy)amino)-5-oxopentanamido)pentyl)(hydroxy)amino)-4-oxobutanoic acid **5** was purified by preparative HPLC (0-15% solvent B over 20 min, product eluted at 15.1 min), lyophilized, and was isolated as a white solid (12 mg, 30%).

^{1}H NMR (600 MHz, D_2O) δ 3.62 (4H, m), 3.17 (2H, t, *J* = 7.0 Hz), 2.98 (2H, t, *J* = 7.5 Hz), 2.71 (2H, t, *J* = 7.2 Hz), 2.51 (2H, t, *J* = 7.7 Hz), 2.45 (2H, t, *J* = 7.5 Hz), 2.26 (2H, t, *J* = 7.2 Hz), 1.85 (2H, m), 1.66 (8H, m), 1.51 (1H, m), 1.35 (2H, m), 1.29 (2H, m). ^{13}C (150 MHz, D_2O) δ 180.8, 175.7, 175.0, 174.7, 47.7, 47.5, 39.3, 39.1, 35.1, 31.9, 30.9, 28.3, 28.3, 27.8, 26.3, 25.4, 25.2, 23.0, 22.6, 20.9. Note: Overlapping signals at 28.3. HRMS calculated for $C_{19}H_{36}N_4O_7$ [M+H]$^+$: 433.2662, observed: 433.2656.

11,22-bis(benzyloxy)-2,2-dimethyl-4,12,15,23-tetraoxo-3-oxa-5,11,16,22-tetraazaheptacosan-27-oic acid (**S12**)

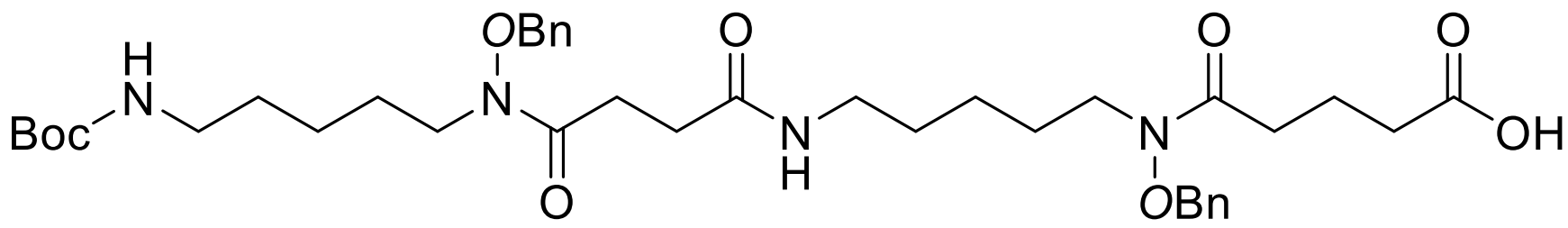

5-((5-aminopentyl)(benzyloxy)amino)-5-oxopentanoic acid **S7** was prepared using the general *N*-Boc deprotection method using TFA/DCM (20% v/v, 5 mL), and 5-((benzyloxy)(5-((*tert*-butoxycarbonyl)amino)pentyl)amino)-5-oxopentanoic acid **S4a** (350 mg, 0.901 mmol.). 5-((5-aminopentyl)(benzyloxy)amino)-5-oxopentanoic acid **S7** was isolated as a colourless oil and was used without further purification.

11,22-bis(benzyloxy)-2,2-dimethyl-4,12,15,23-tetraoxo-3-oxa-5,11,16,22-tetraazaheptacosan-27-oic acid **S12** was prepared using the general amide coupling procedure using DIPEA (0.715 mL, 4.11 mmol.), HBTU (311 mg, 0.821 mmol.), 5-((5-aminopentyl)(benzyloxy)amino)-5-oxopentanoic acid **S3** (291 mg, 0.903 mmol.), and 4-((benzyloxy)(5-((*tert*-butoxycarbonyl)amino)pentyl)amino)-4-oxobutanoic acid **S7** (335 mg, 0.821 mmol.). 11,22-bis(benzyloxy)-2,2-dimethyl-4,12,15,23-tetraoxo-3-oxa-5,11,16,22-tetraazaheptacosan-27-oic acid **S12** was purified by preparative HPLC (50-65% solvent B over 12 min, product eluted at 8.9 min), lyophilized, and was isolated as a white solid (280 mg, 46%).

^{1}H NMR (600 MHz, D_2O) δ 7.77 (1H, t, *J* = 5.2 Hz), 7.40 (10H, m), 6.74 (1H, t, *J* = 5.3 Hz), 4.87 (2H, s), 4.84 (2H, s), 3.56 (4H, m), 2.99 (2H, dd, *J* = 6.2, 12.7 Hz), 2.87 (2H, dd, *J* = 6.4, 12.7 Hz), 2.62 (2H, m), 2.40 (2H, t, *J* = 6.9 Hz), 2.30 (2H, t, *J* = 7.0 Hz), 2.21 (2H, t, *J* = 7.3 Hz), 1.68 (2H, m), 1.50 (4H, m), 1.36 (13H, m), 1.20 (4H, m). ^{13}C (150 MHz, D_2O) δ 174.3, 173.0, 172.9, 171.0, 155.6, 129.4, 129.3, 128.7, 128.6, 128.5, 77.3, 75.4, 40.0, 38.4, 32.9, 30.7, 29.7, 29.1, 28.8, 28.3, 27.2, 26.2, 23.5, 23.4, 19.6. HRMS calculated for $C_{38}H_{56}N_4O_9$ [M+H]$^+$: 713.4126, observed: 713.4109.

5-((5-(4-((5-aminopentyl)(hydroxy)amino)-4-oxobutanamido)pentyl)(hydroxy)amino)-5-oxopentanoic acid (**6**)

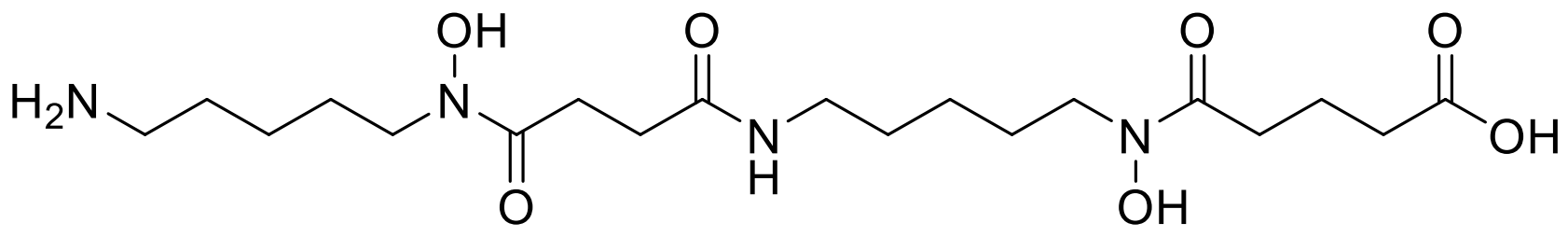

-((5-(4-((5-aminopentyl)(hydroxy)amino)-4-oxobutanamido)pentyl)(hydroxy)amino)-5-oxopentanoic acid **6** was prepared using the general transfer hydrogenation method using Pd/C (28.0 mg, 10 wt%), ammonium formation (495 mg, 7.86 mmol.), and 11,22-bis(benzyloxy)-2,2-dimethyl-4,12,15,23-tetraoxo-3-oxa-5,11,16,22-tetraazaheptacosan-27-oic acid **S12** (280 mg, 0.393 mmol.) followed immediately by the general *N*-Boc deprotection procedure using TFA/DCM (20% v/v, 5 mL). 5-((5-(4-((5-aminopentyl)(hydroxy)amino)-4-oxobutanamido)pentyl)(hydroxy)amino)-5-oxopentanoic acid **6** was purified by preparative HPLC (0-15% solvent B over 20 min, product eluted at 15.1 min), lyophilized, and was isolated as a white solid (21 mg, 50%).

^{1}H NMR (600 MHz, D_2O) δ 3.64 (4H, m), 3.17 (2H, t, *J* = 7.2 Hz), 2.99 (1H, t, *J* = 7.6 Hz), 2.80 (2H, t, *J* = 6.9 Hz), 2.51 (4H, m), 2.23 (2H, t, *J* = 7.5 Hz), 1.83 (2H, m), 1.66 (6H, m), 1.52 (2H, m), 1.36 (2H, m), 1.30 (2H, m). ^{13}C (150 MHz, D_2O) δ 182.3, 175.4, 174.8, 173.9, 47.7, 47.6, 39.3, 39.1, 36.6, 31.4, 30.3, 27.8, 27.5, 26.3, 25.4, 25.2, 23.0, 22.6, 21.4. HRMS calculated for $C_{19}H_{36}N_4O_7$ $[M+H]^+$: 433.2662, observed: 433.2656.

Characterisation Data of Synthesized Compounds

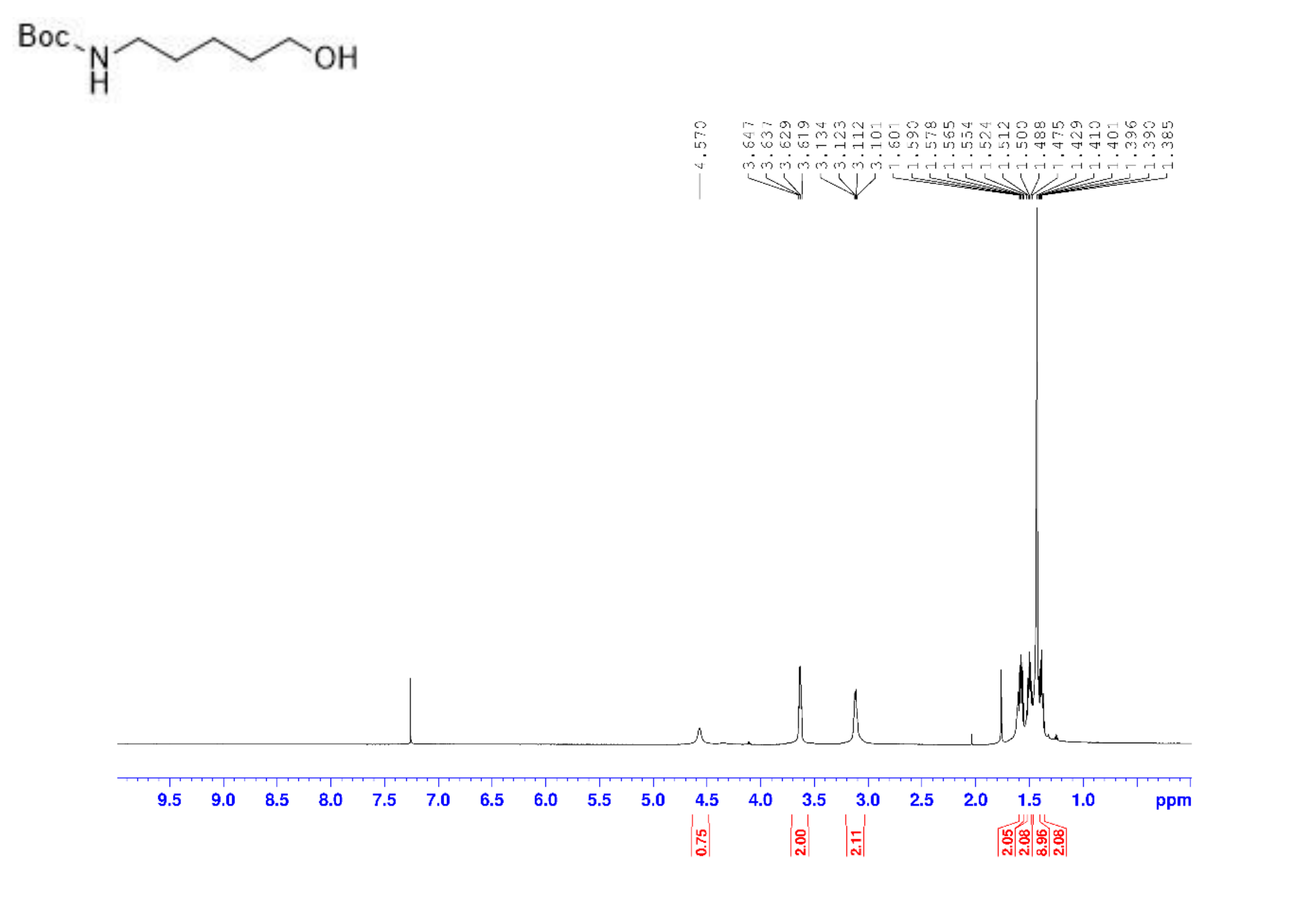

Figure S22. ^{1}H NMR (600 MHz, $CDCl_3$) spectrum of **S1**

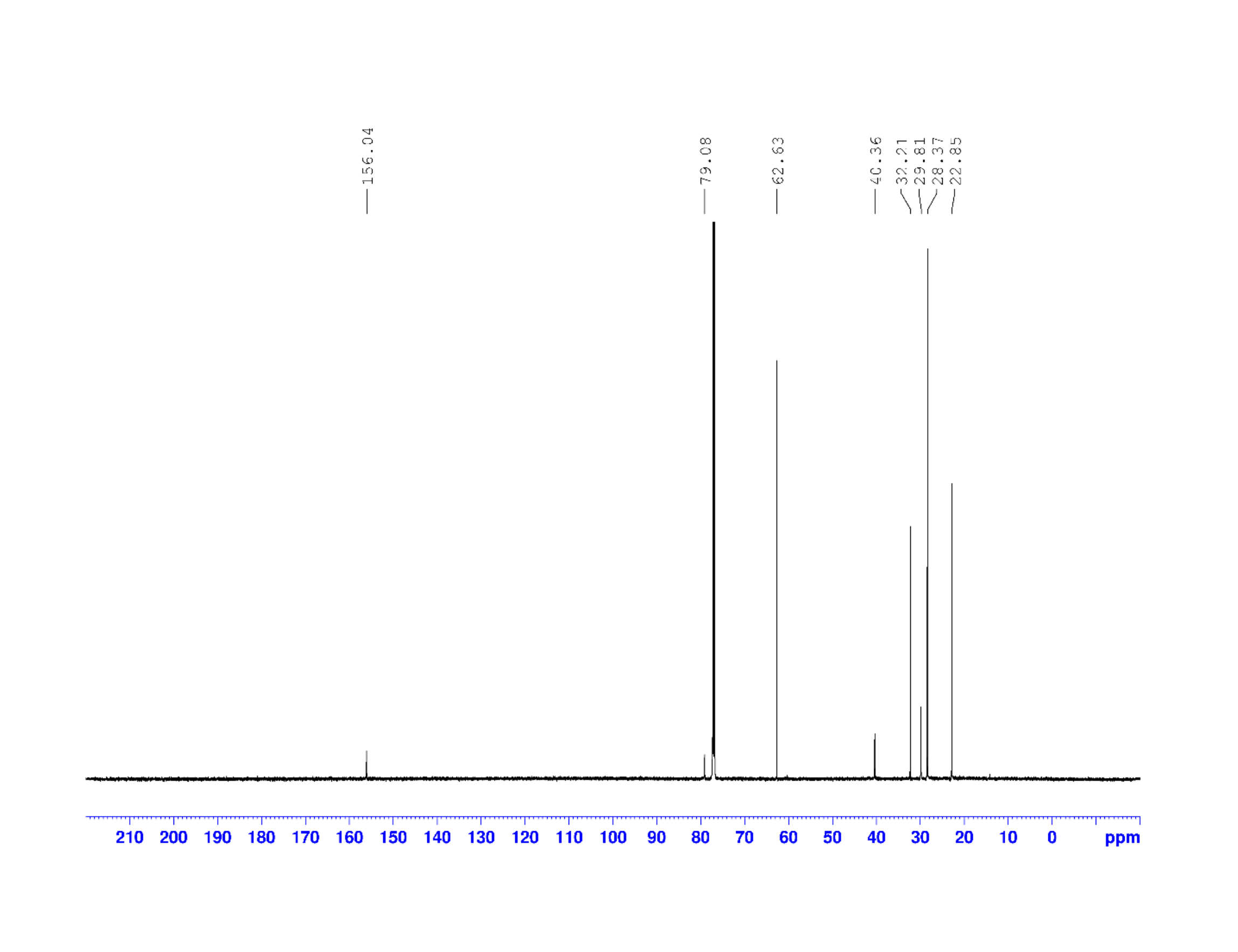

Figure S23. ^{13}C NMR (150 MHz, $CDCl_3$) spectrum of **S1**

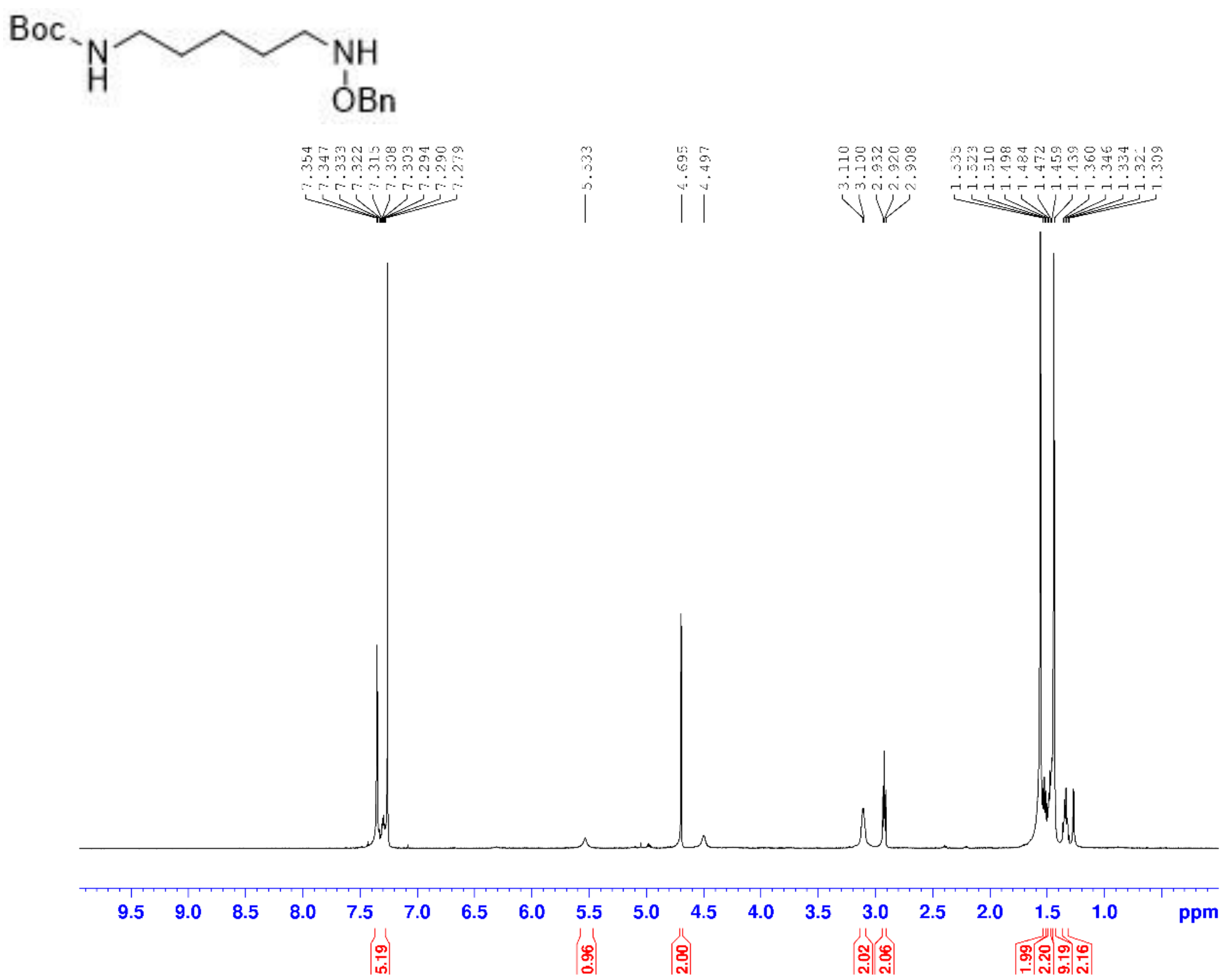

Figure S24. ^{1}H NMR (600 MHz, $CDCl_3$) spectrum of **S2a**

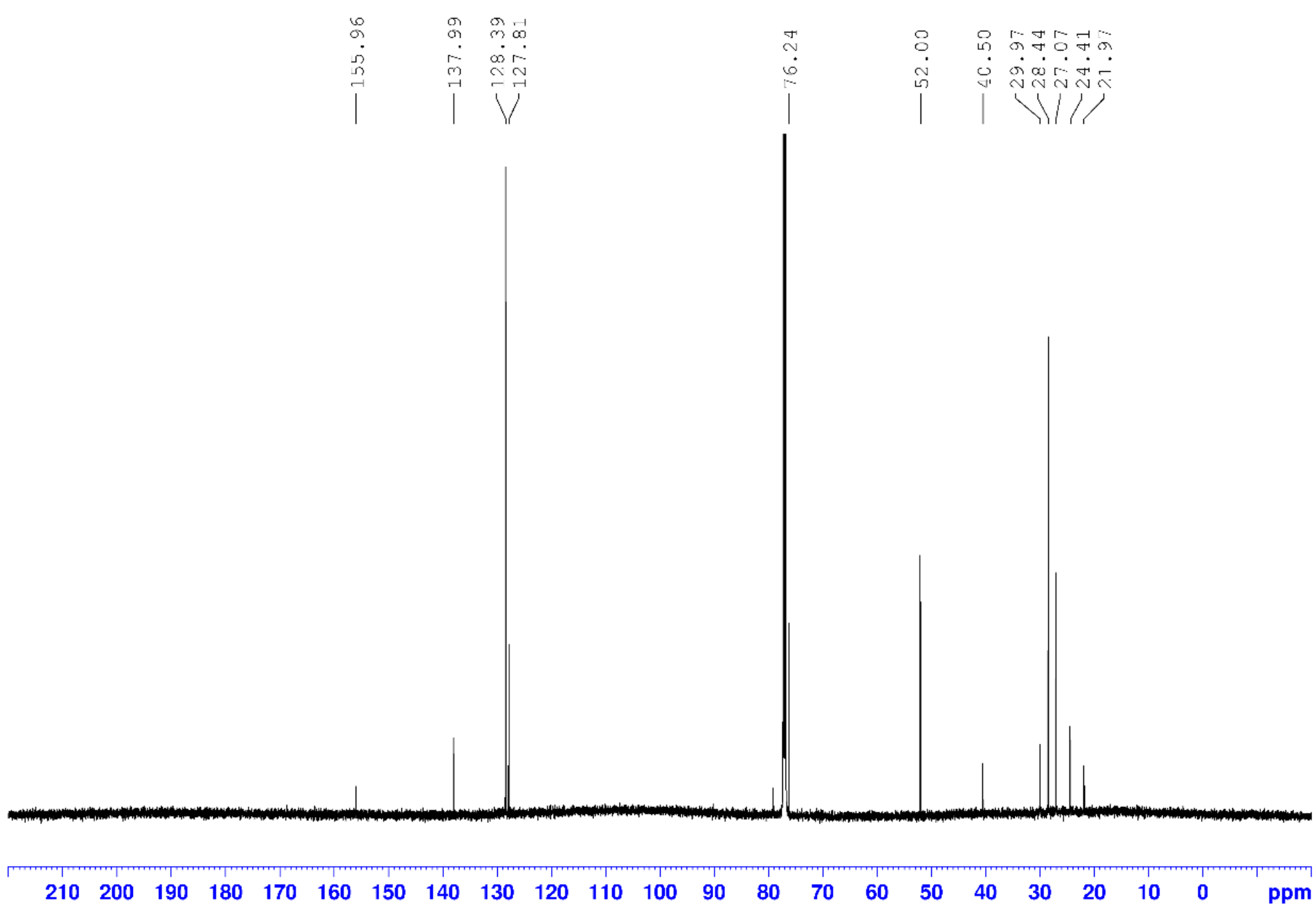

Figure S25. ^{13}C NMR (150 MHz, $CDCl_3$) spectrum of **S2a**

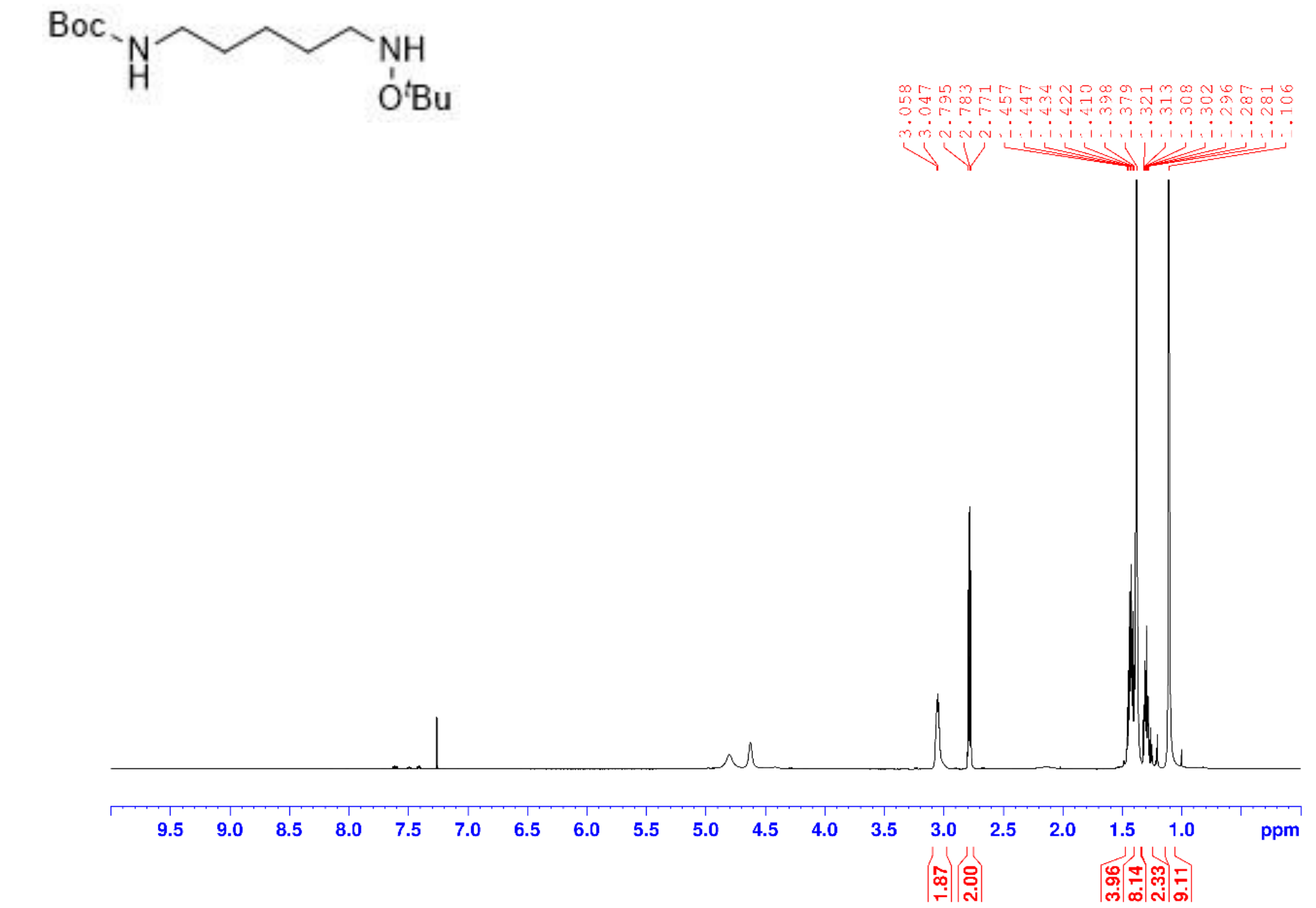

Figure S26. ^{1}H NMR (600 MHz, $CDCl_3$) spectrum of **S2b**

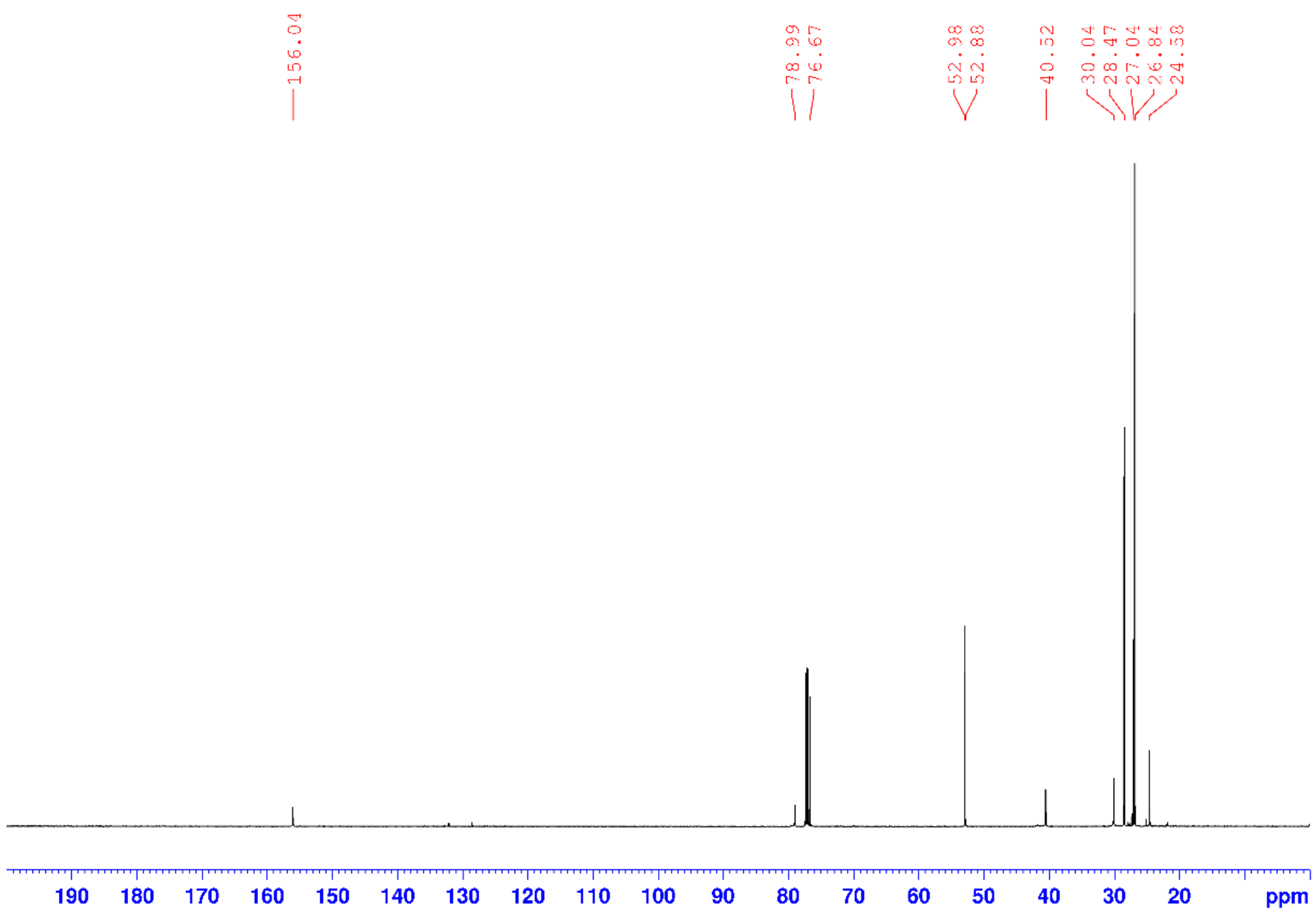

Figure S27. ^{13}C NMR (150 MHz, $CDCl_3$) spectrum of **S2b**

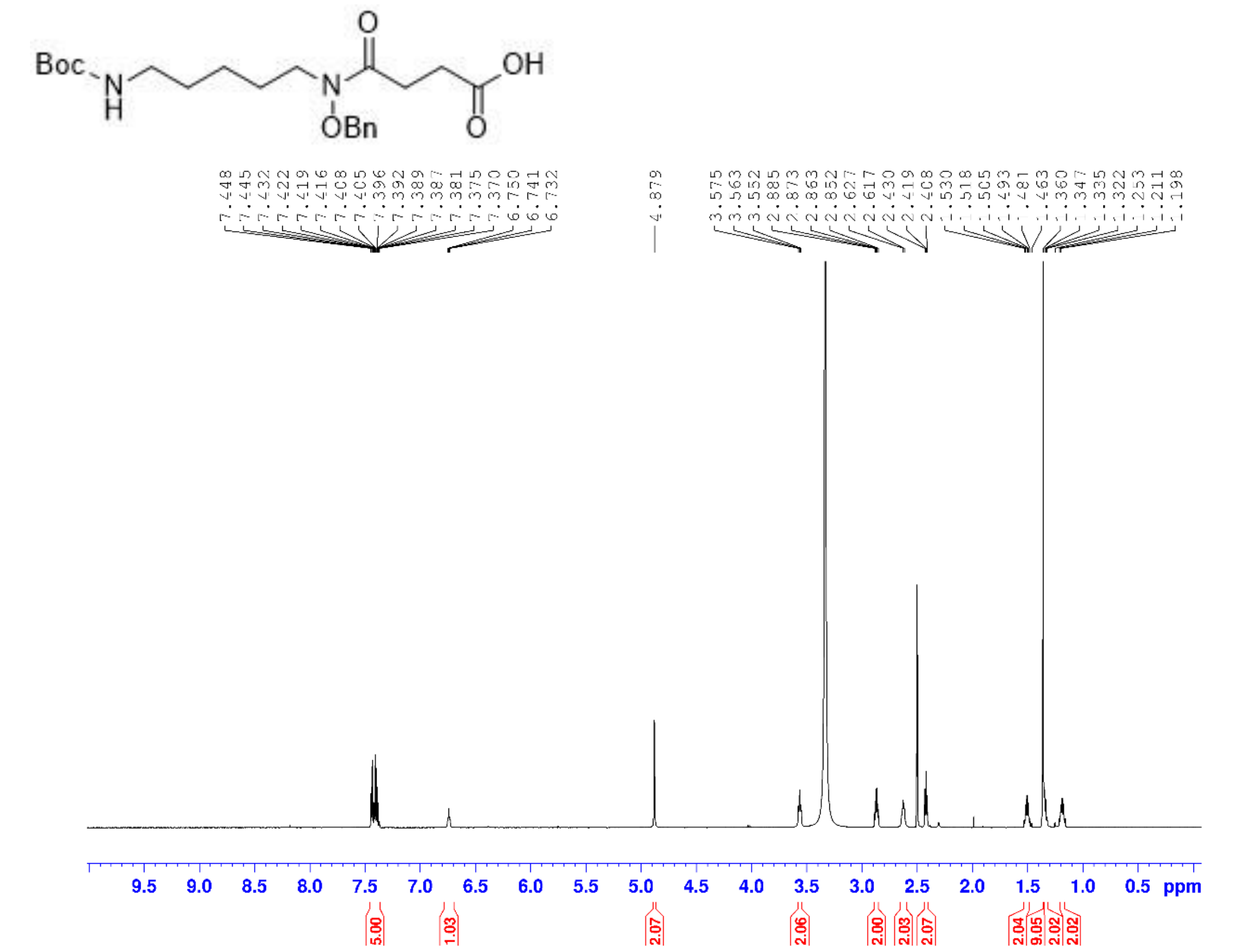

Figure S28. ^{1}H NMR (600 MHz, DMSO-d_6) spectrum of **S3**

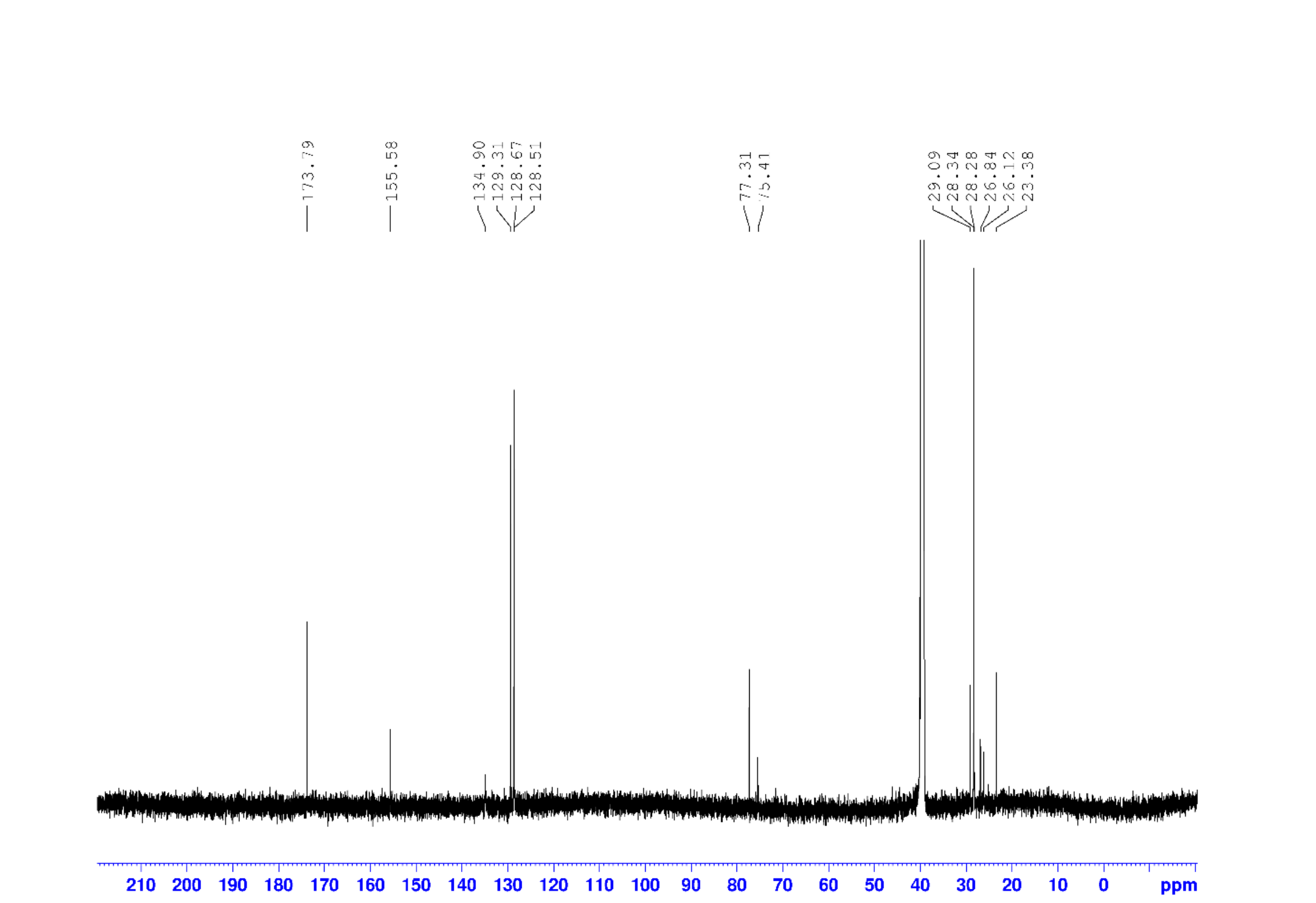

Figure S29. ^{13}C NMR (150 MHz, DMSO-d_6) spectrum of **S3**

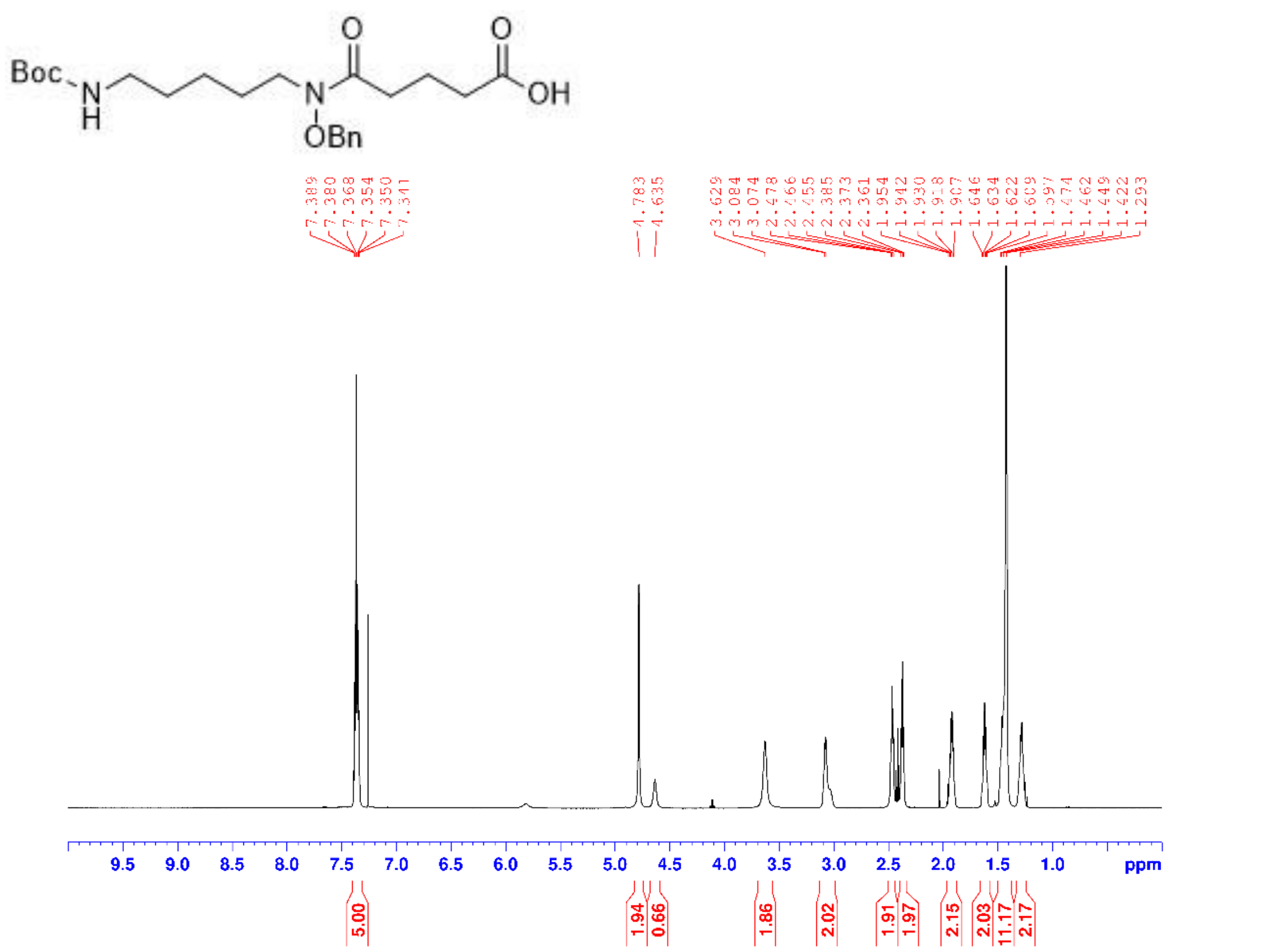

Figure S30. ^{1}H NMR (600 MHz, $CDCl_3$) spectrum of **S4a**

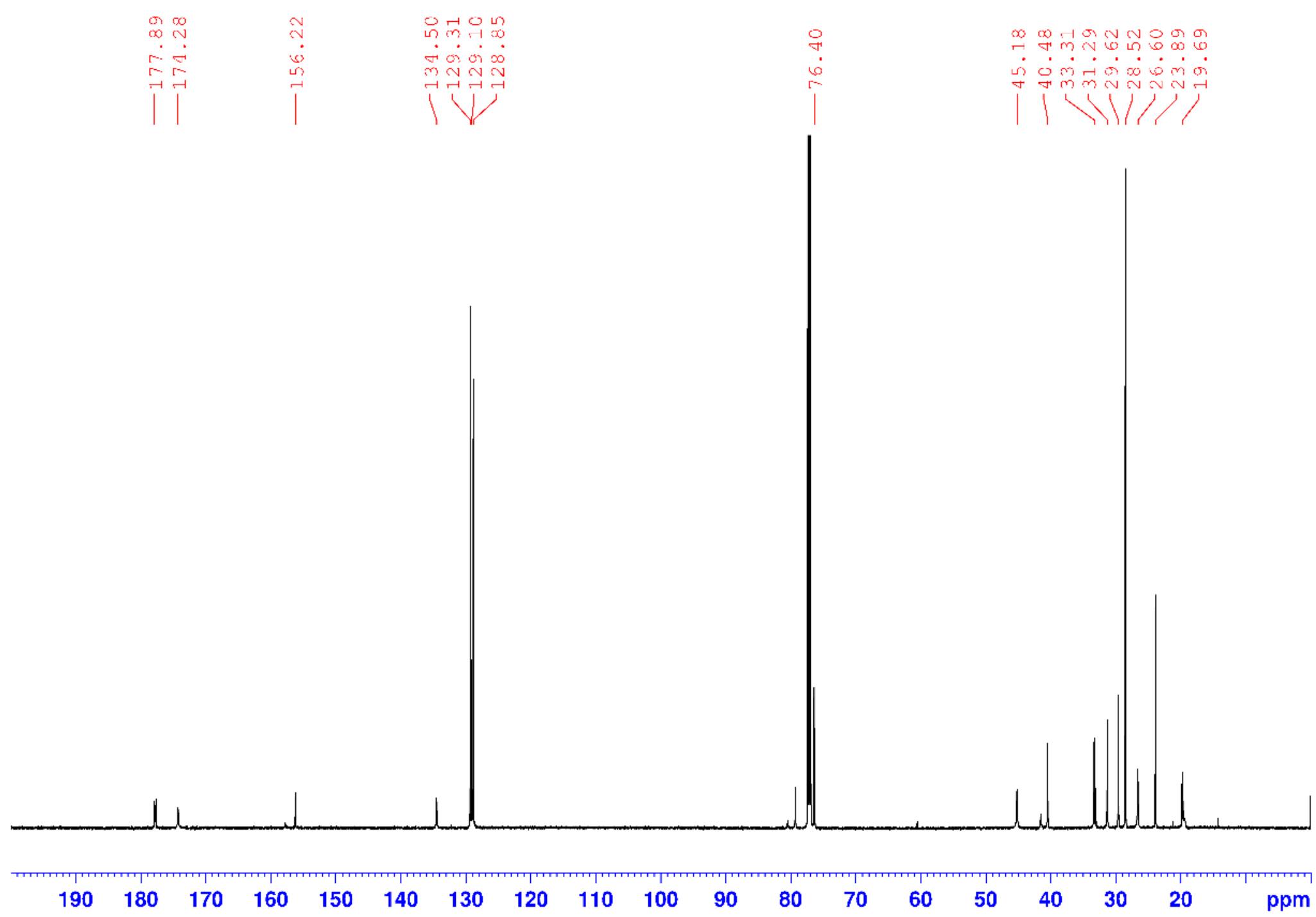

Figure S31. ^{13}C NMR (150 MHz, $CDCl_3$) spectrum of **S4a**

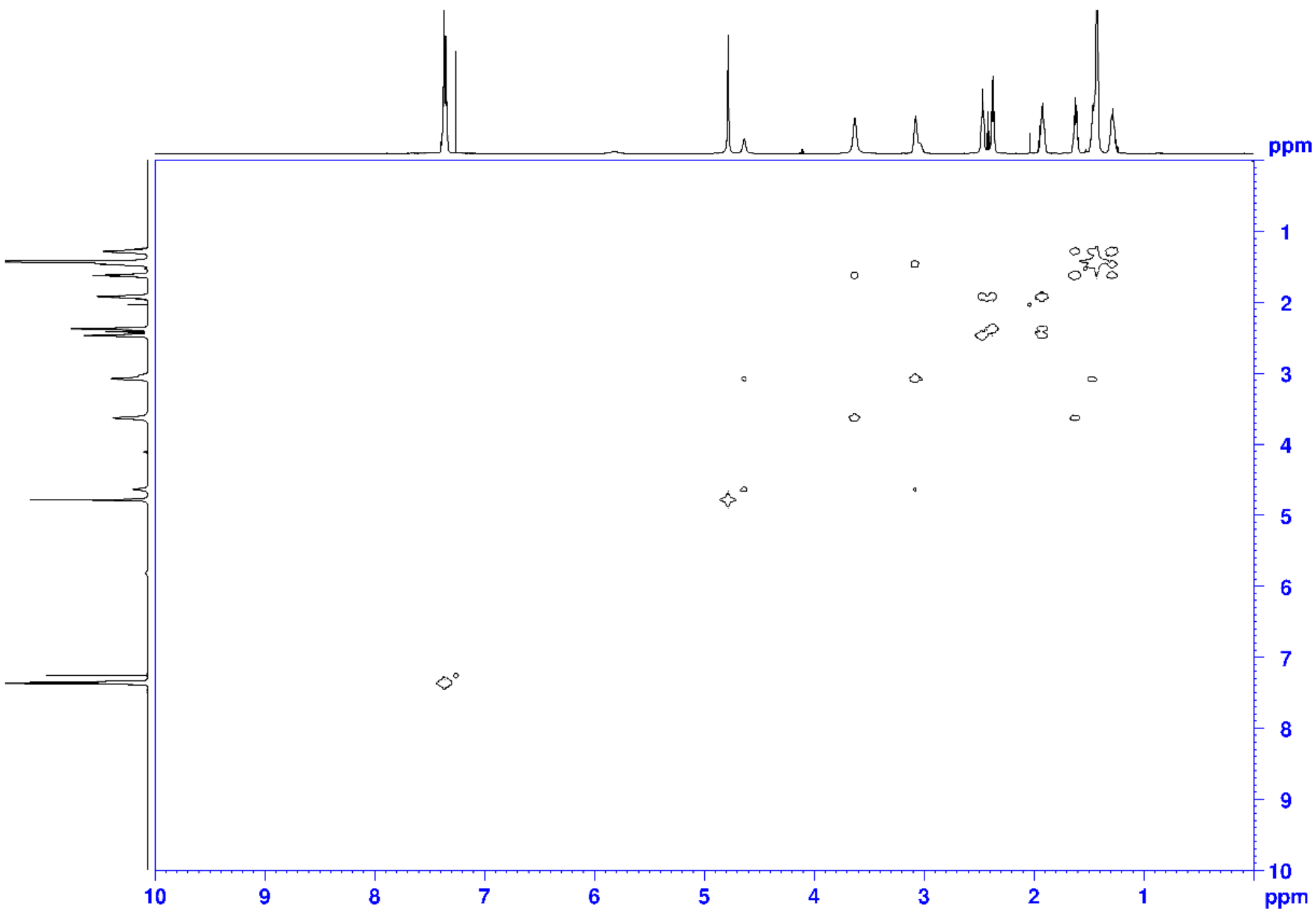

Figure S32. COSY NMR (600 MHz, $CDCl_3$) spectrum of **S4a**

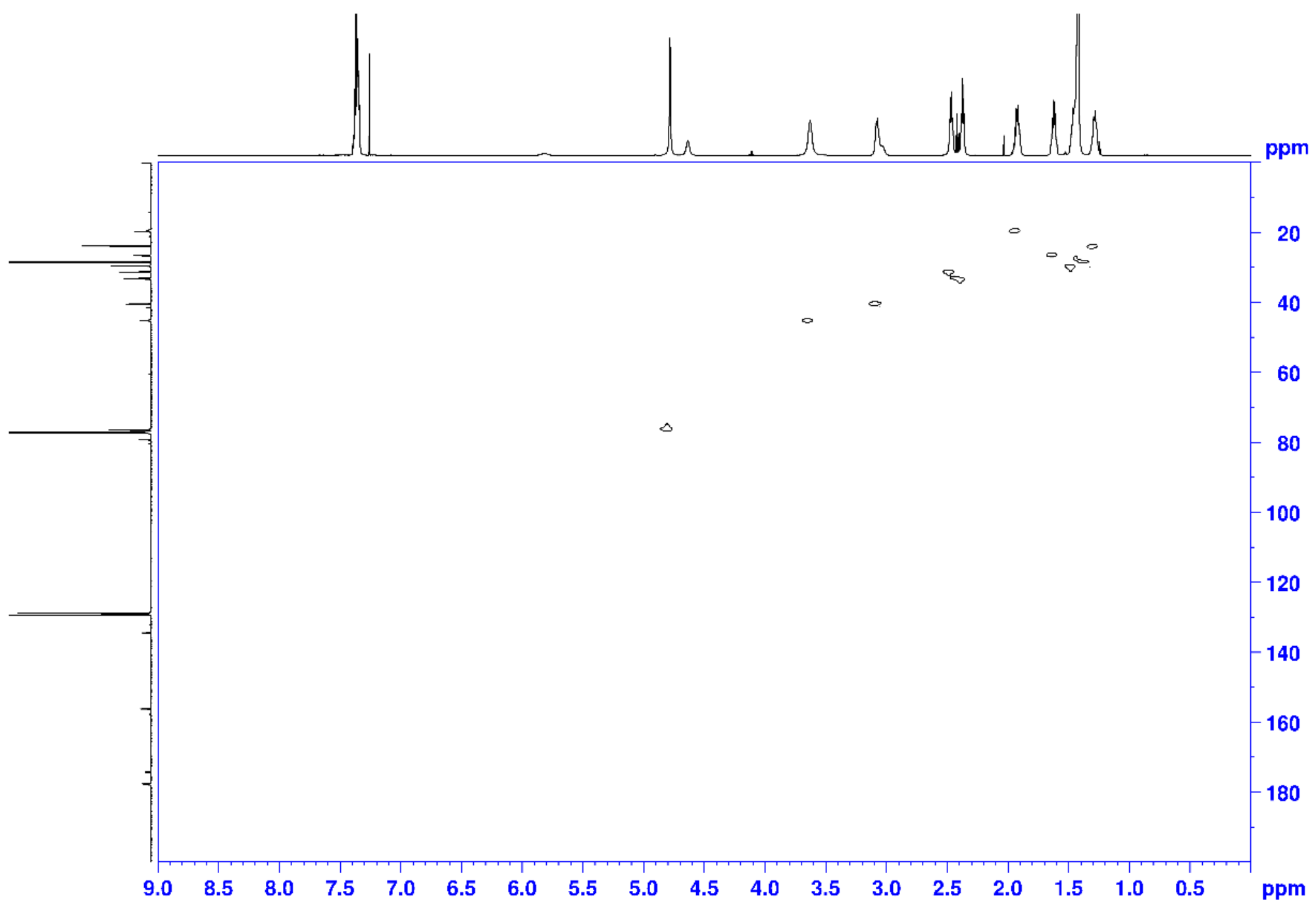

Figure S33. HSQC NMR (600 MHz, $CDCl_3$) spectrum of **S4a**

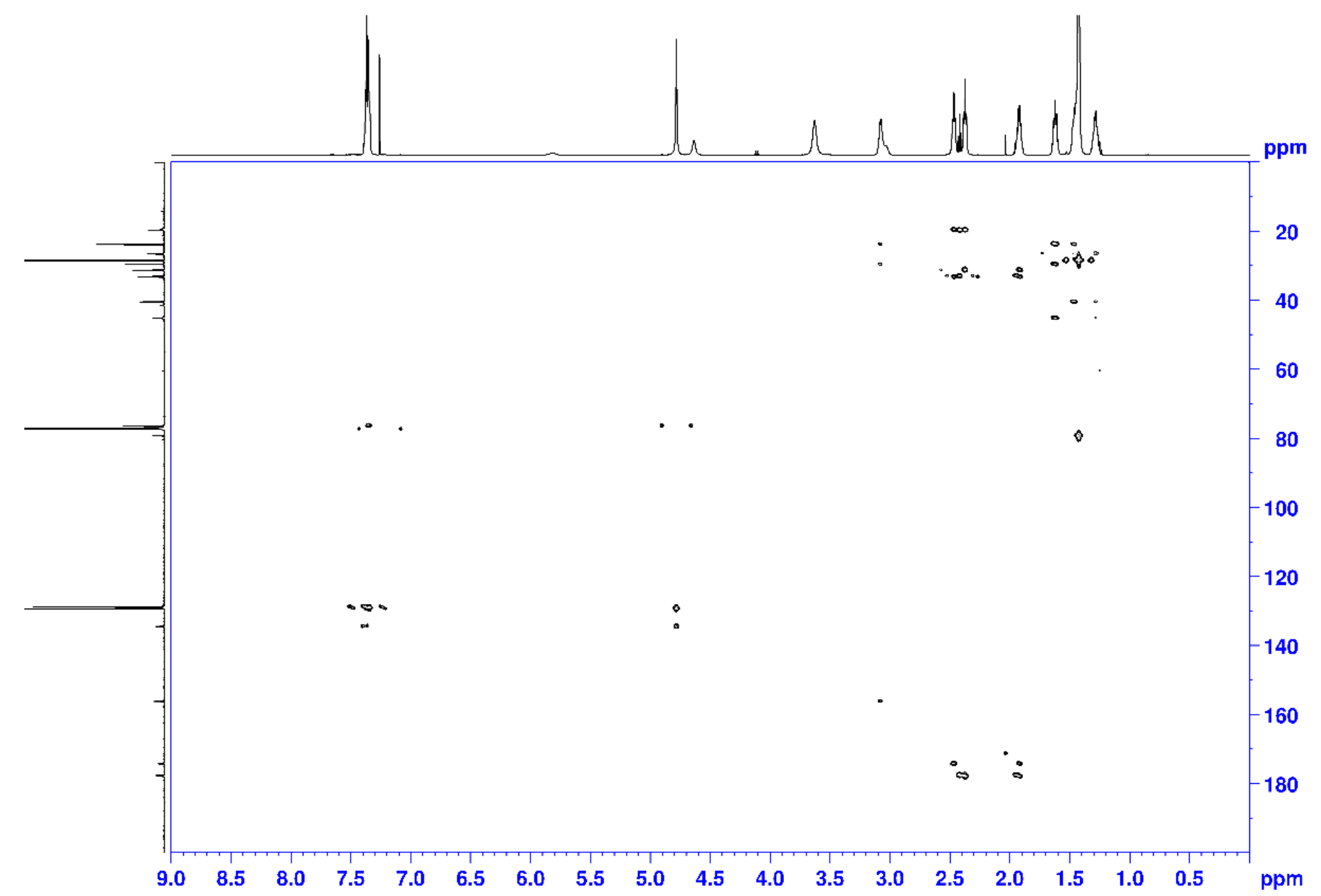

Figure S34. HMBC NMR (600 MHz, $CDCl_3$) spectrum of **S4a**

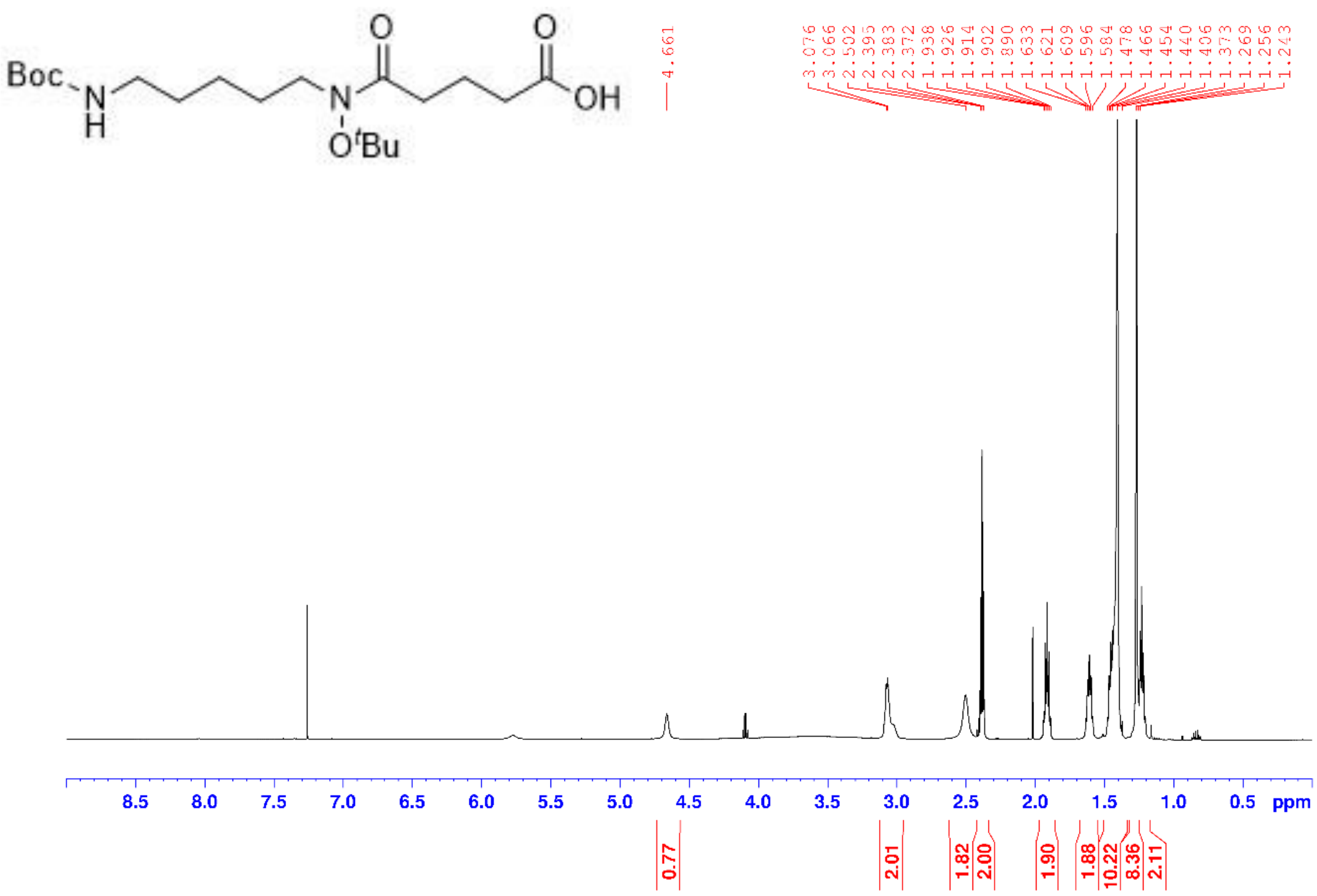

Figure S35. ^{1}H NMR (600 MHz, $CDCl_3$) spectrum of **S4b**

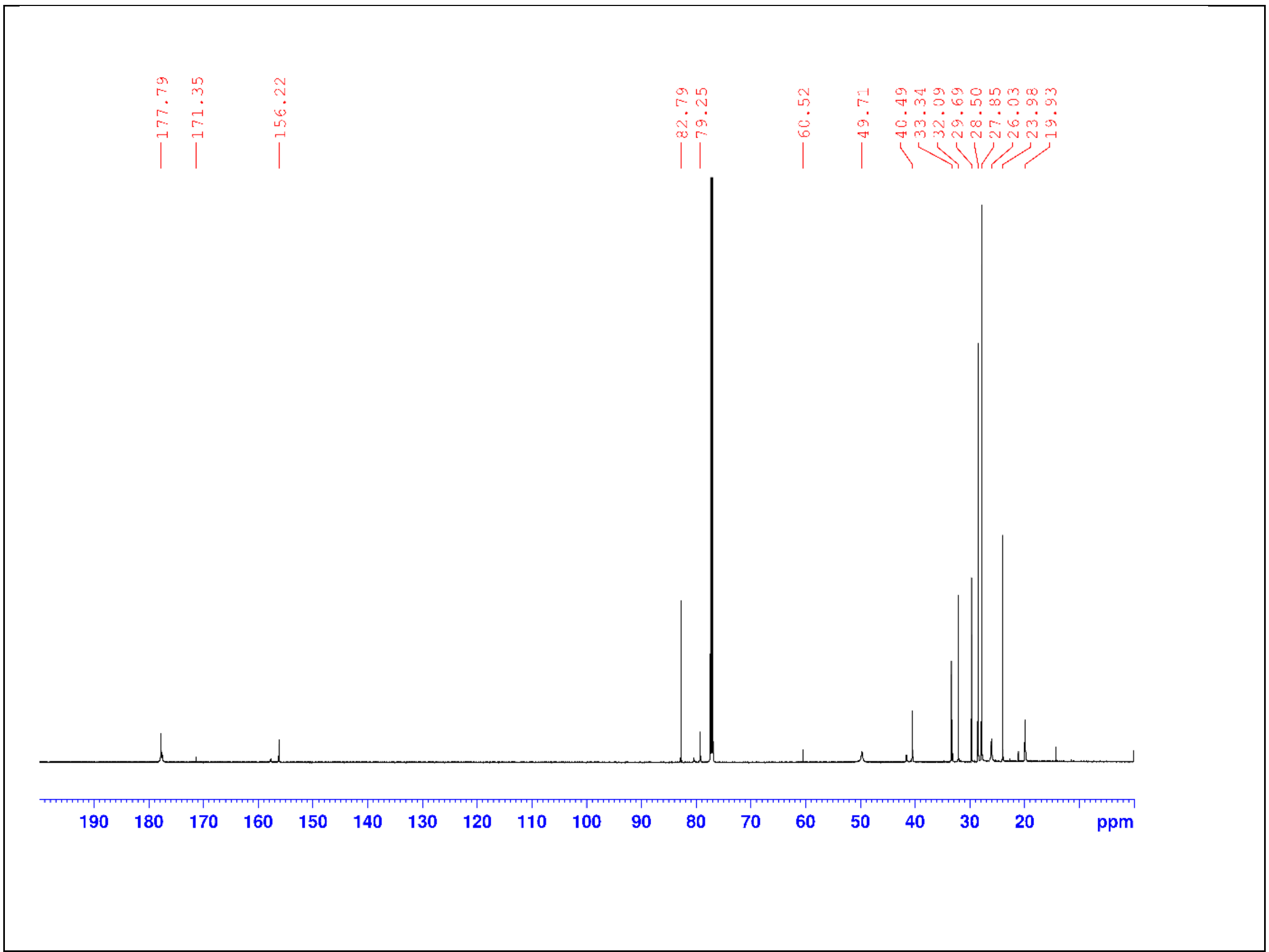

Figure S36. ^{13}C NMR (150 MHz, $CDCl_3$) spectrum of **S4b**

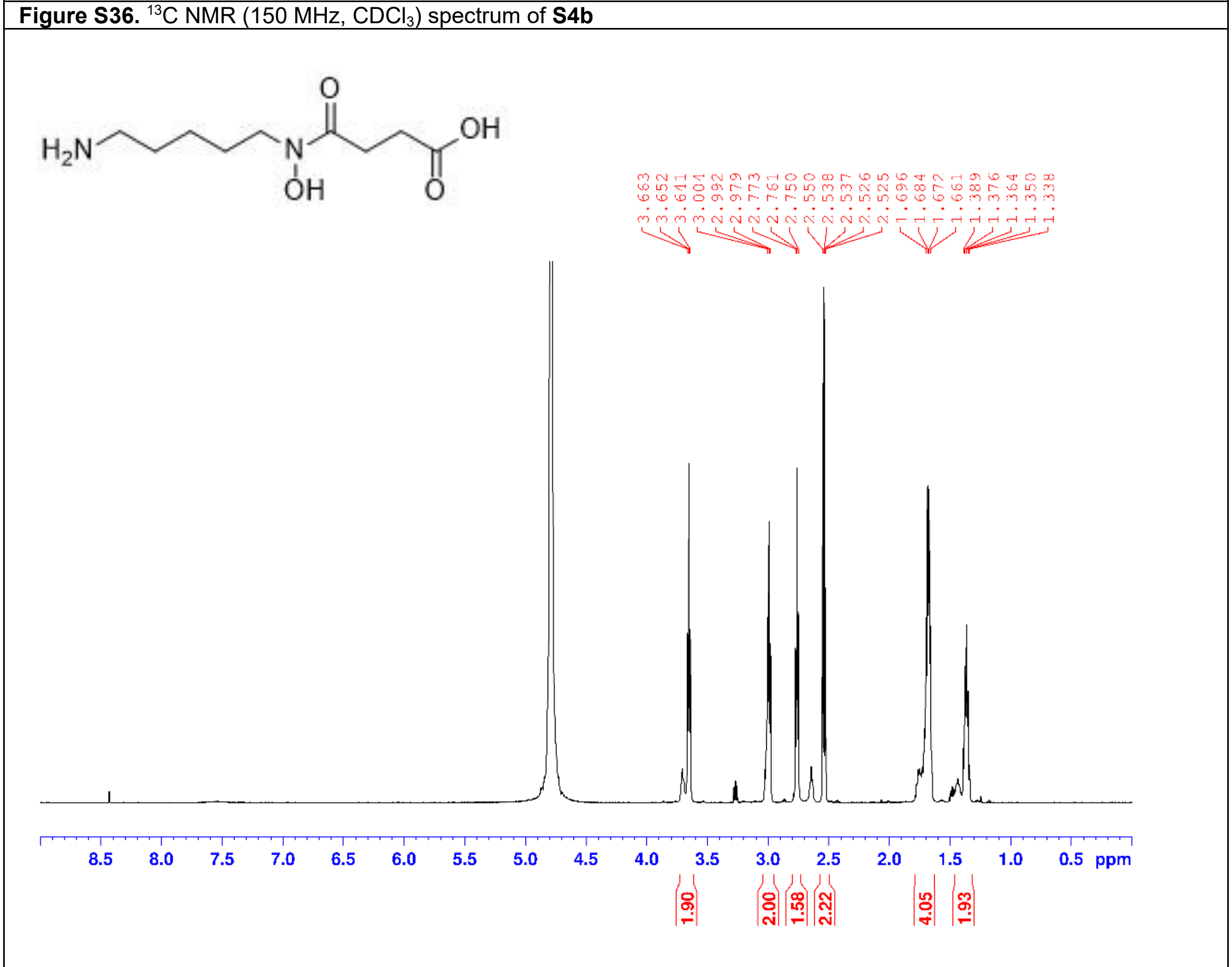

Figure S37. ^{1}H NMR (600 MHz, D_2O) spectrum of **1**. Peak at 3.3 ppm due to *E/Z* conformation of the hydroxamic acid.[4]

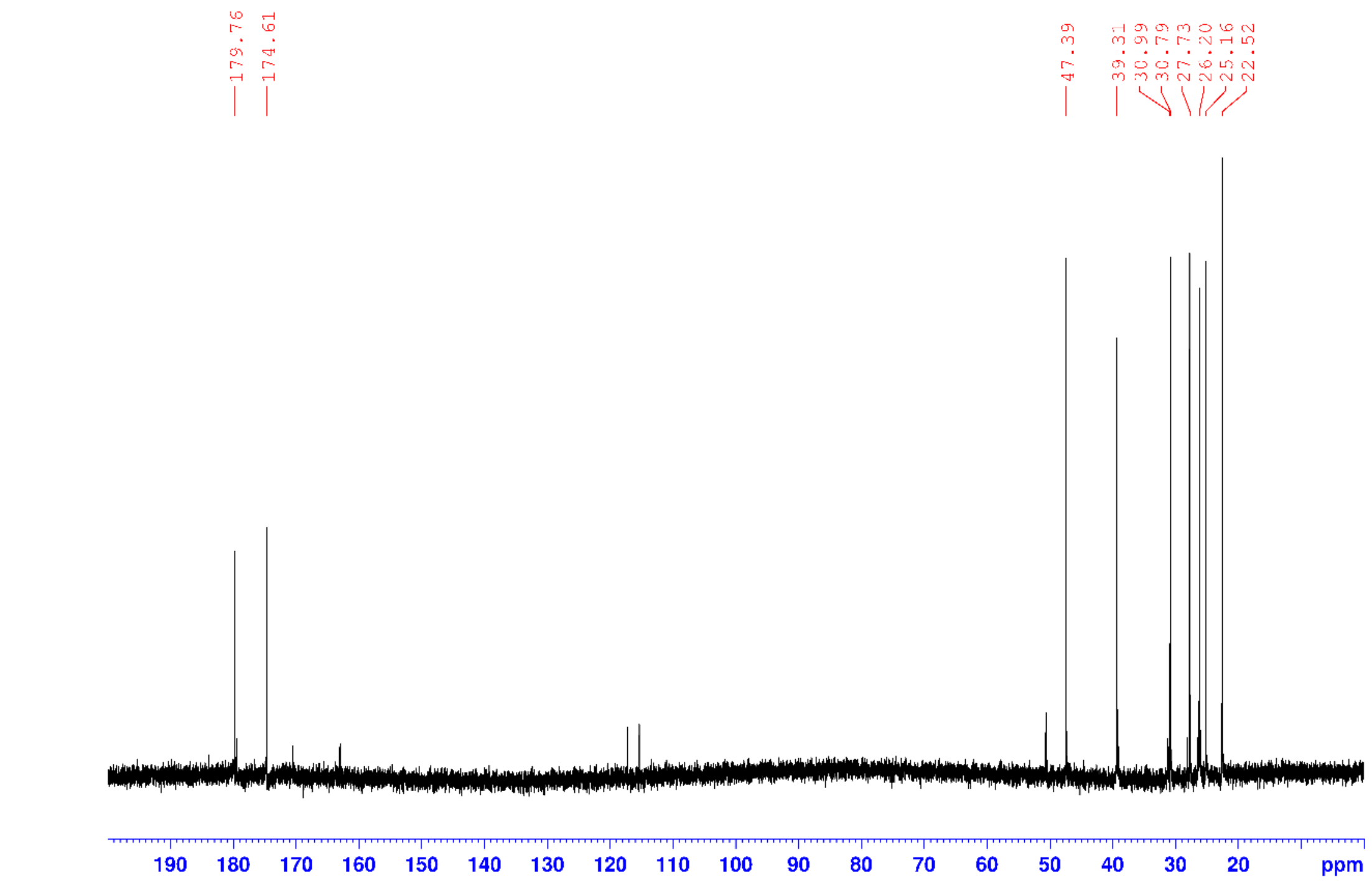

Figure S38. ^{13}C NMR (150 MHz, D_2O) spectrum of **1.** Peak at 30.99 ppm due to *E/Z* conformation of the hydroxamic acid.[4]

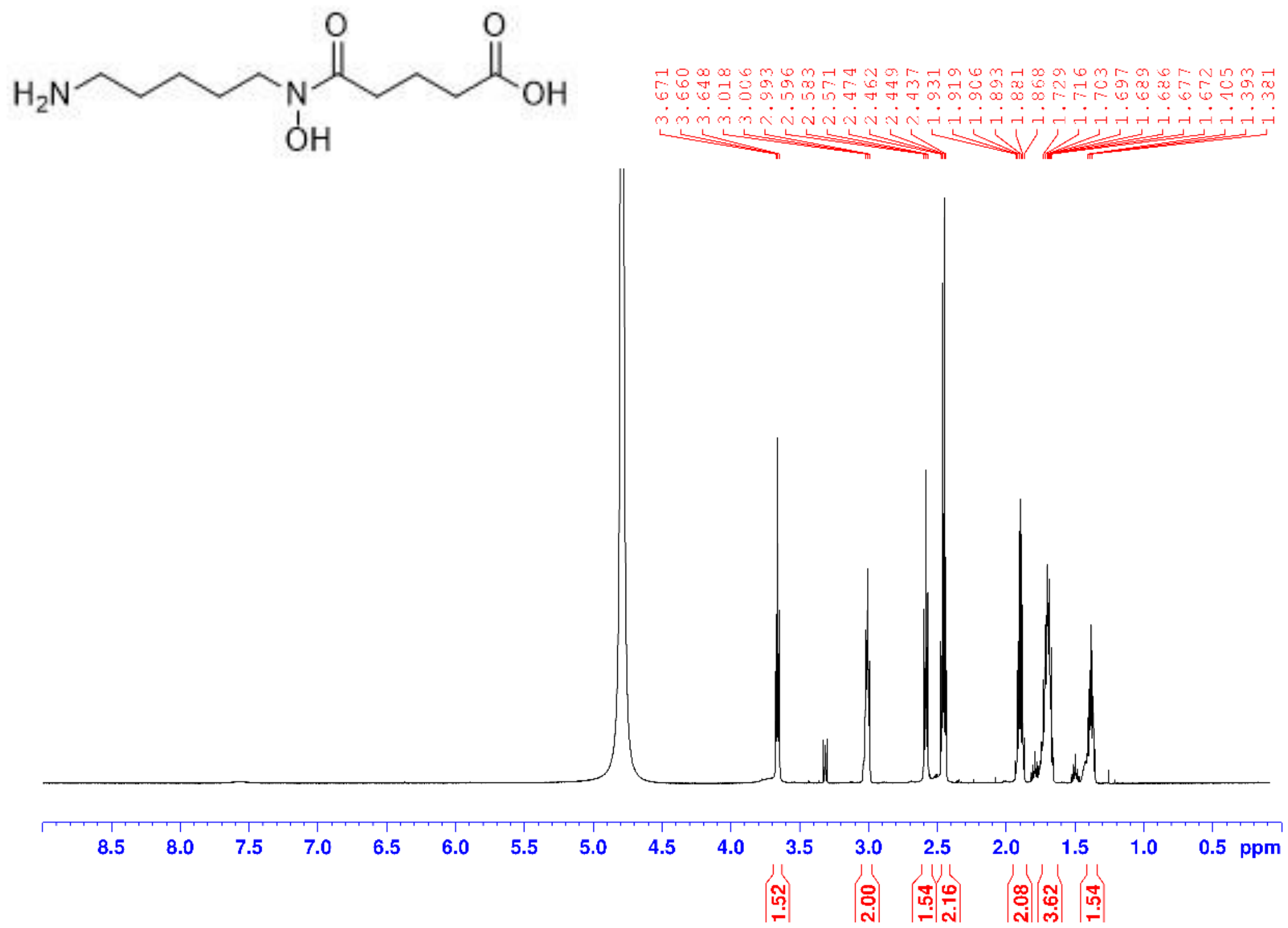

Figure S39. ^{1}H NMR (600 MHz, D_2O) spectrum of **2**

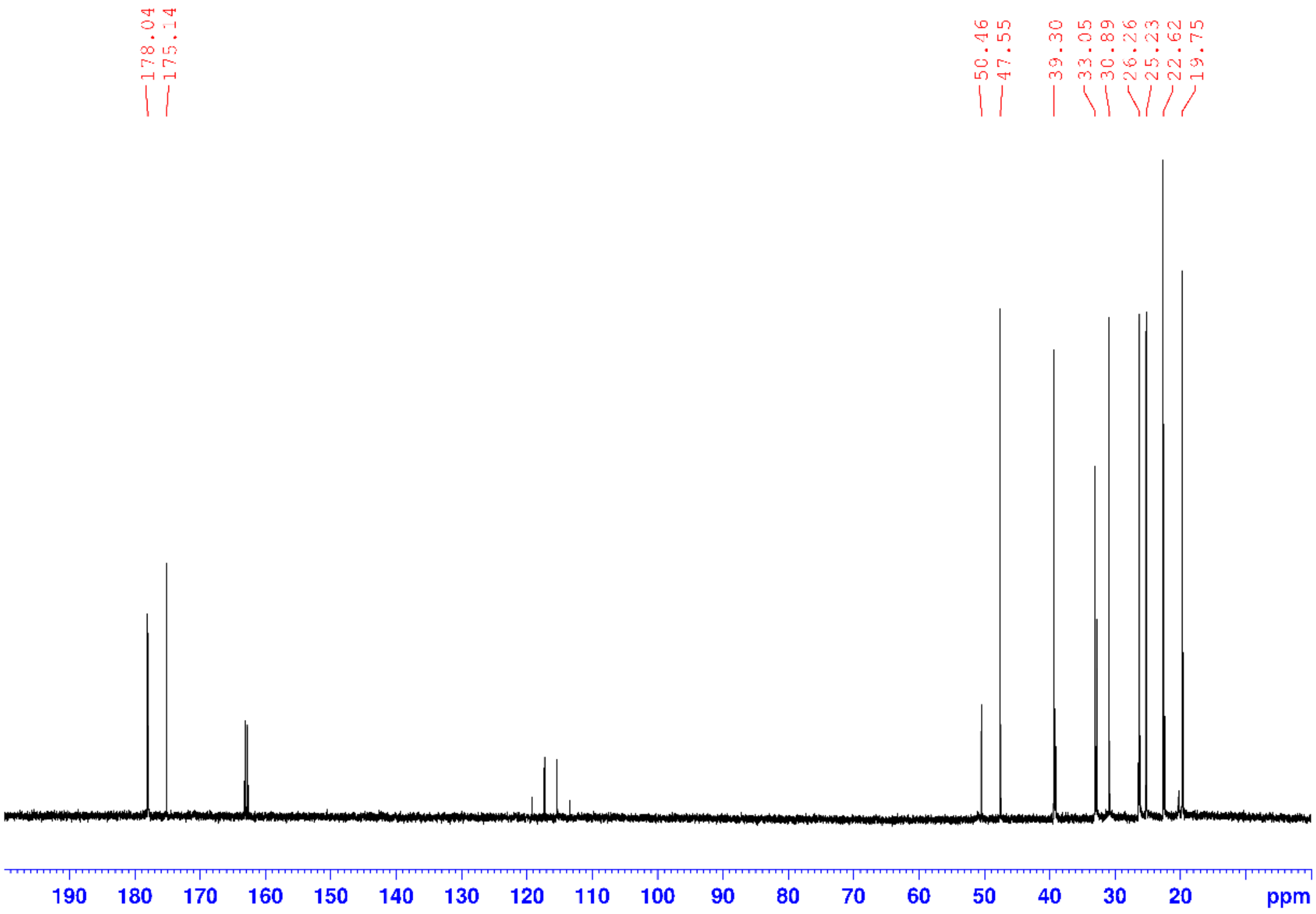

Figure S40. ^{13}C NMR (150 MHz, D_2O) spectrum of **1.** Peak at 50.46 ppm due to *E/Z* conformation of the hydroxamic acid.[4]

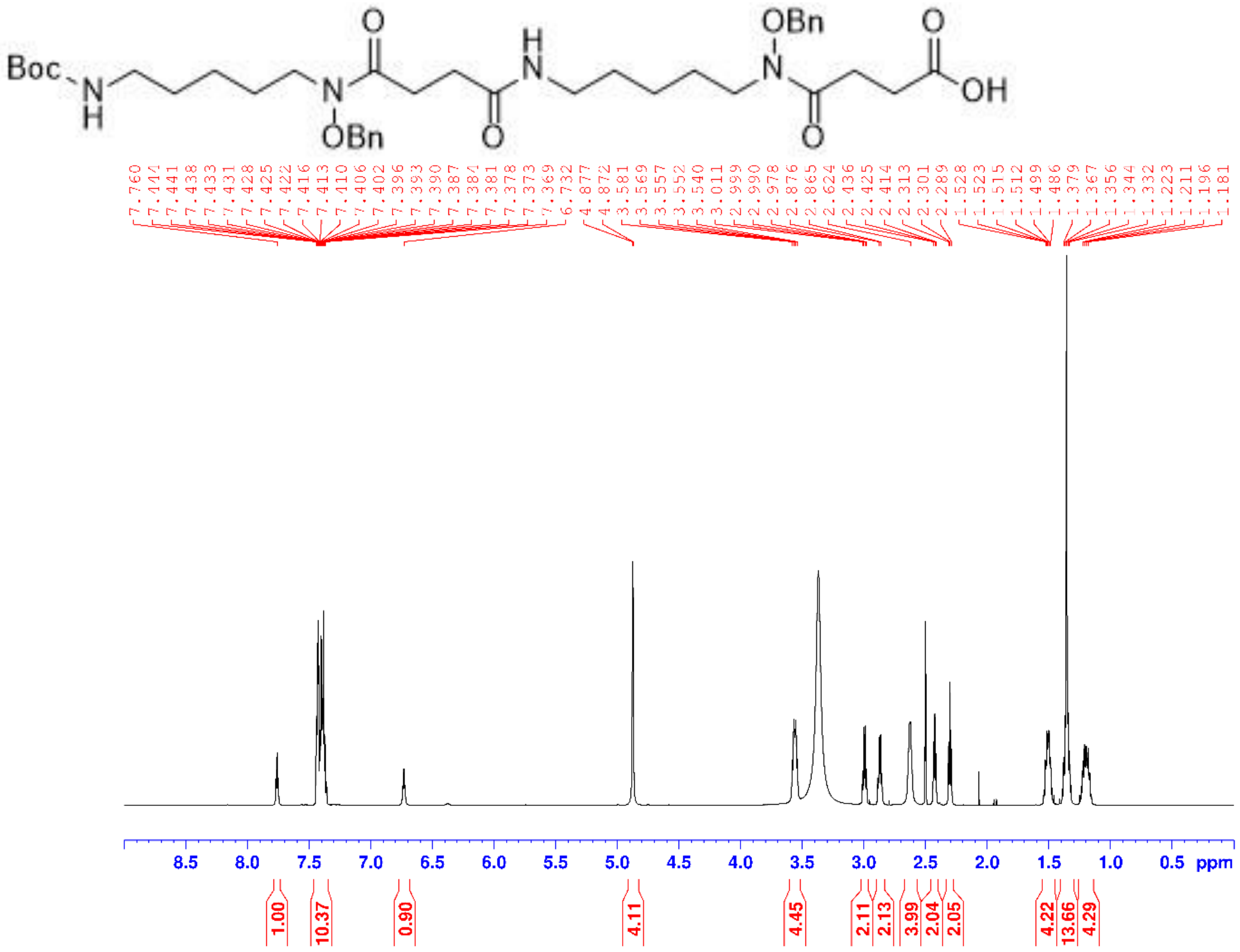

Figure S41. ^{1}H NMR (600 MHz, DMSO-d_6) spectrum of **S9**

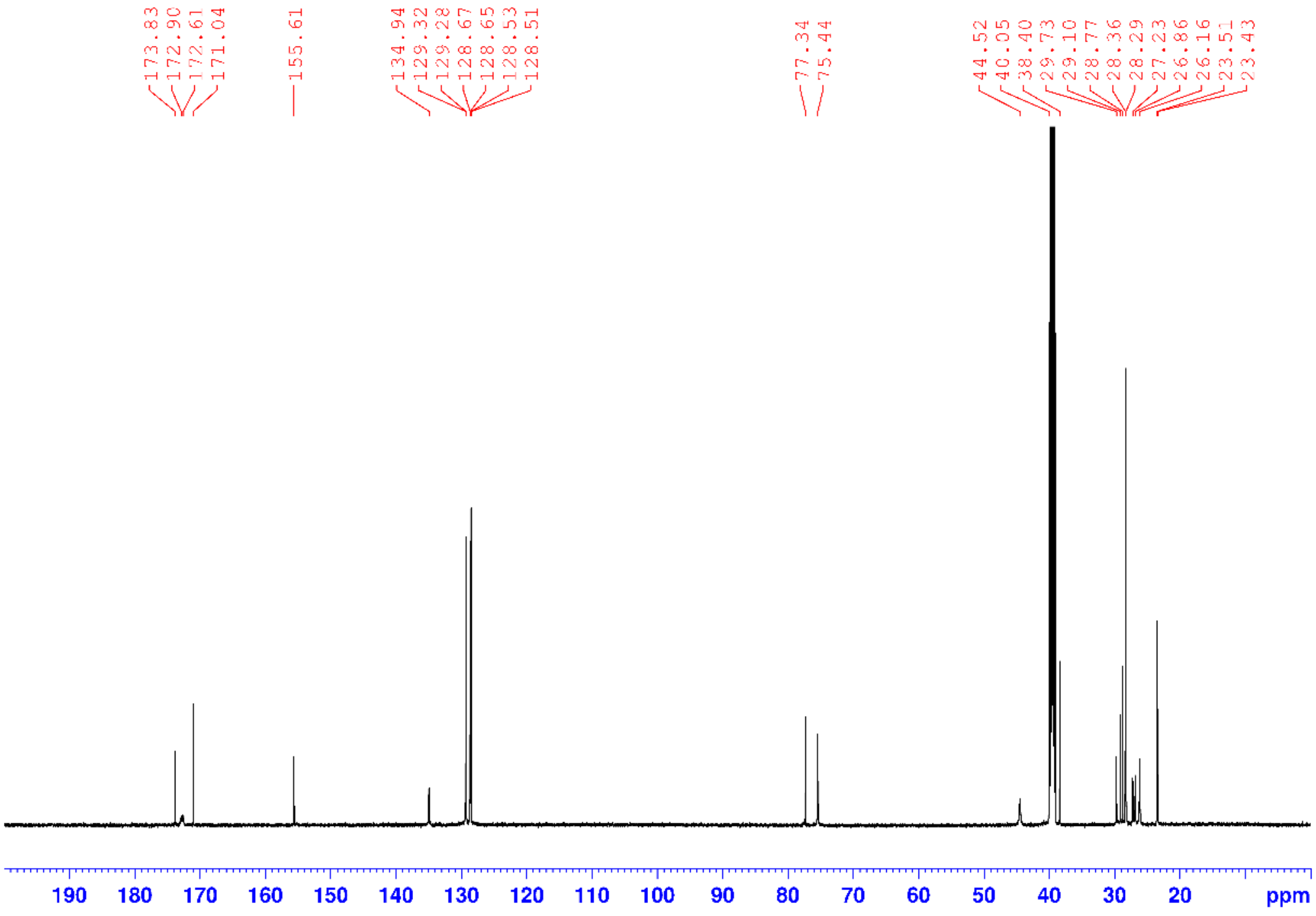

Figure S42. ^{13}C NMR (150 MHz, DMSO-d_6) spectrum of **S9**

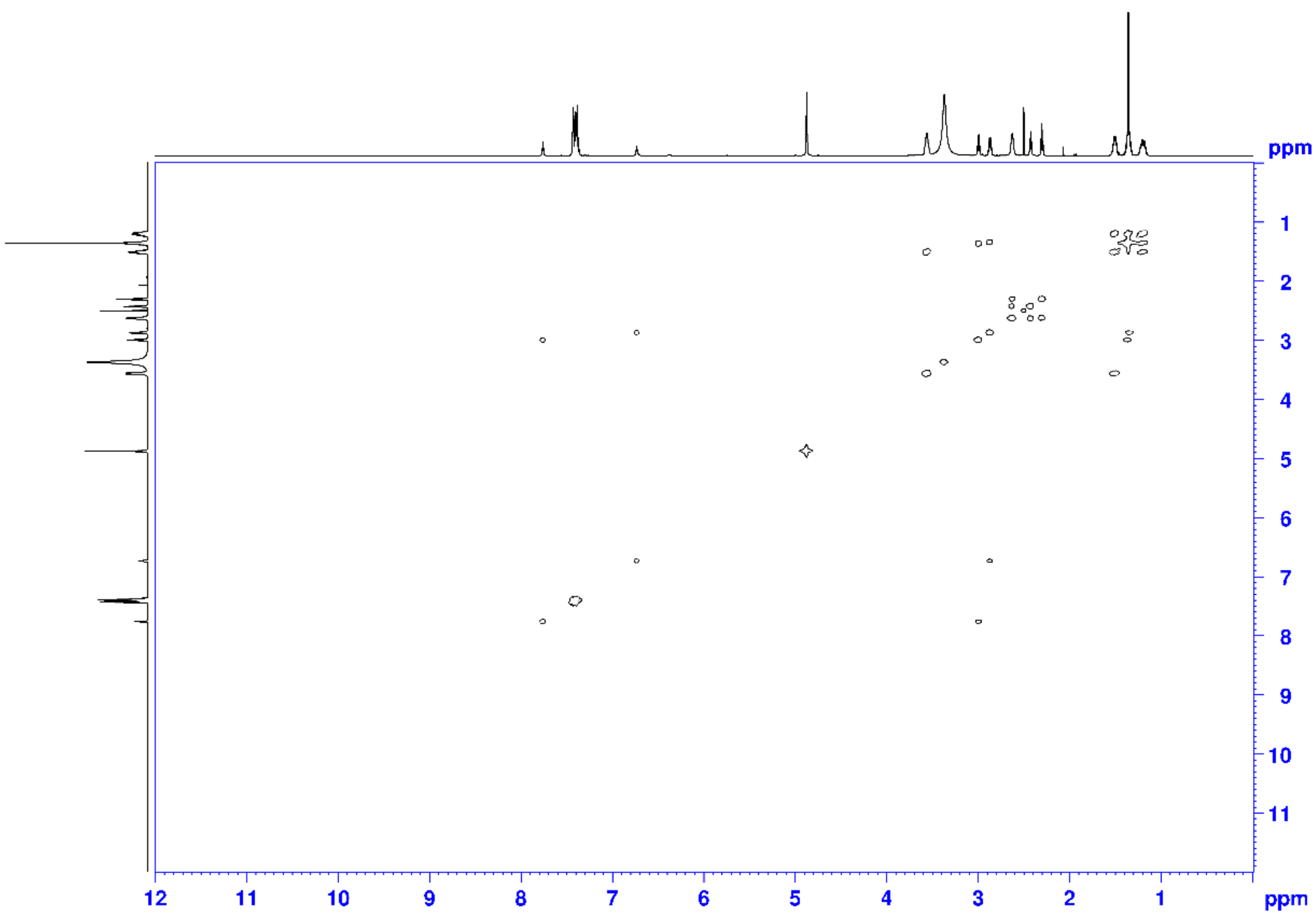

Figure S43. COSY NMR (600 MHz, DMSO-d_6) spectrum of **S9**

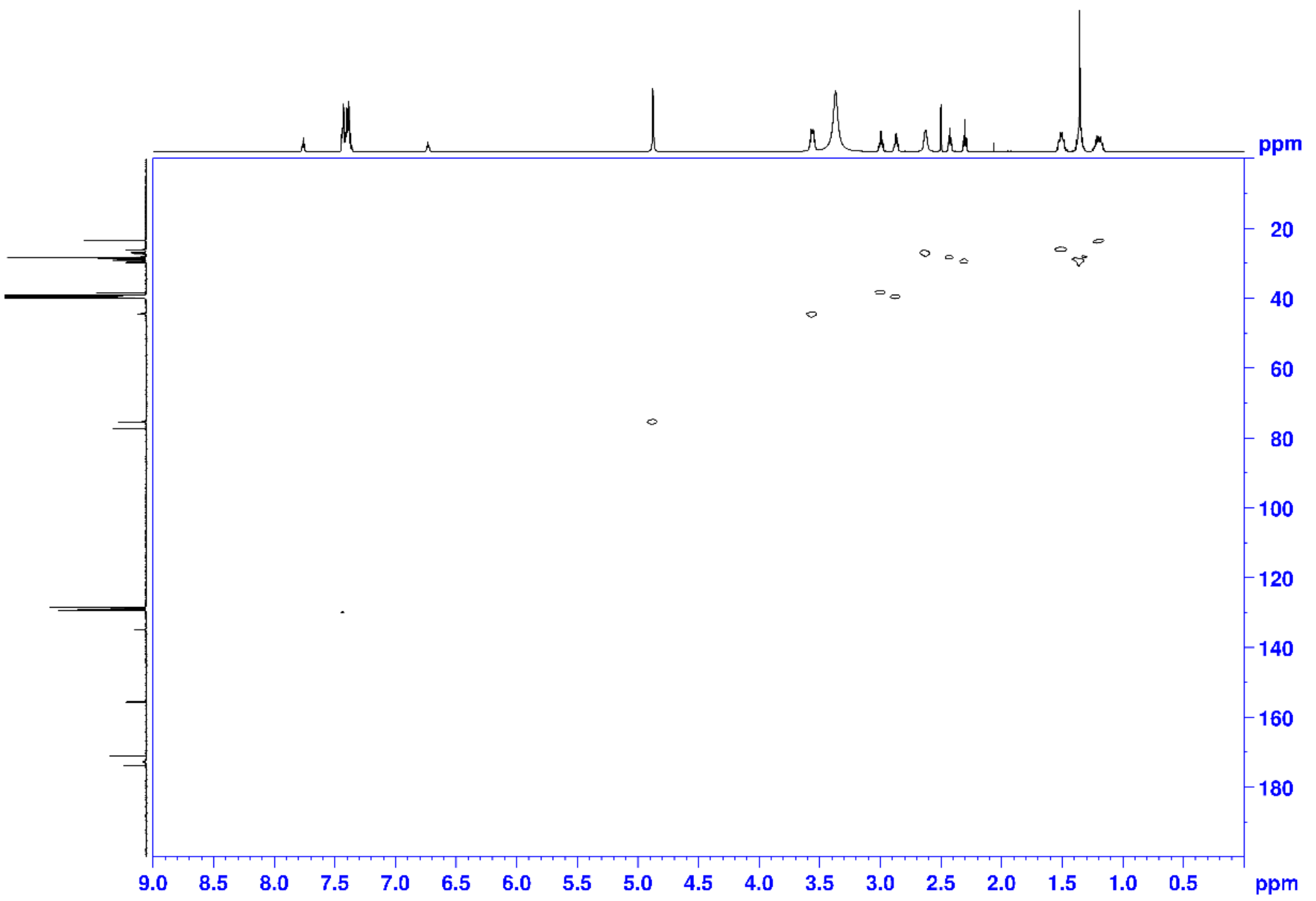

Figure S44. HSQC NMR (600 MHz, DMSO-d_6) spectrum of **S9**

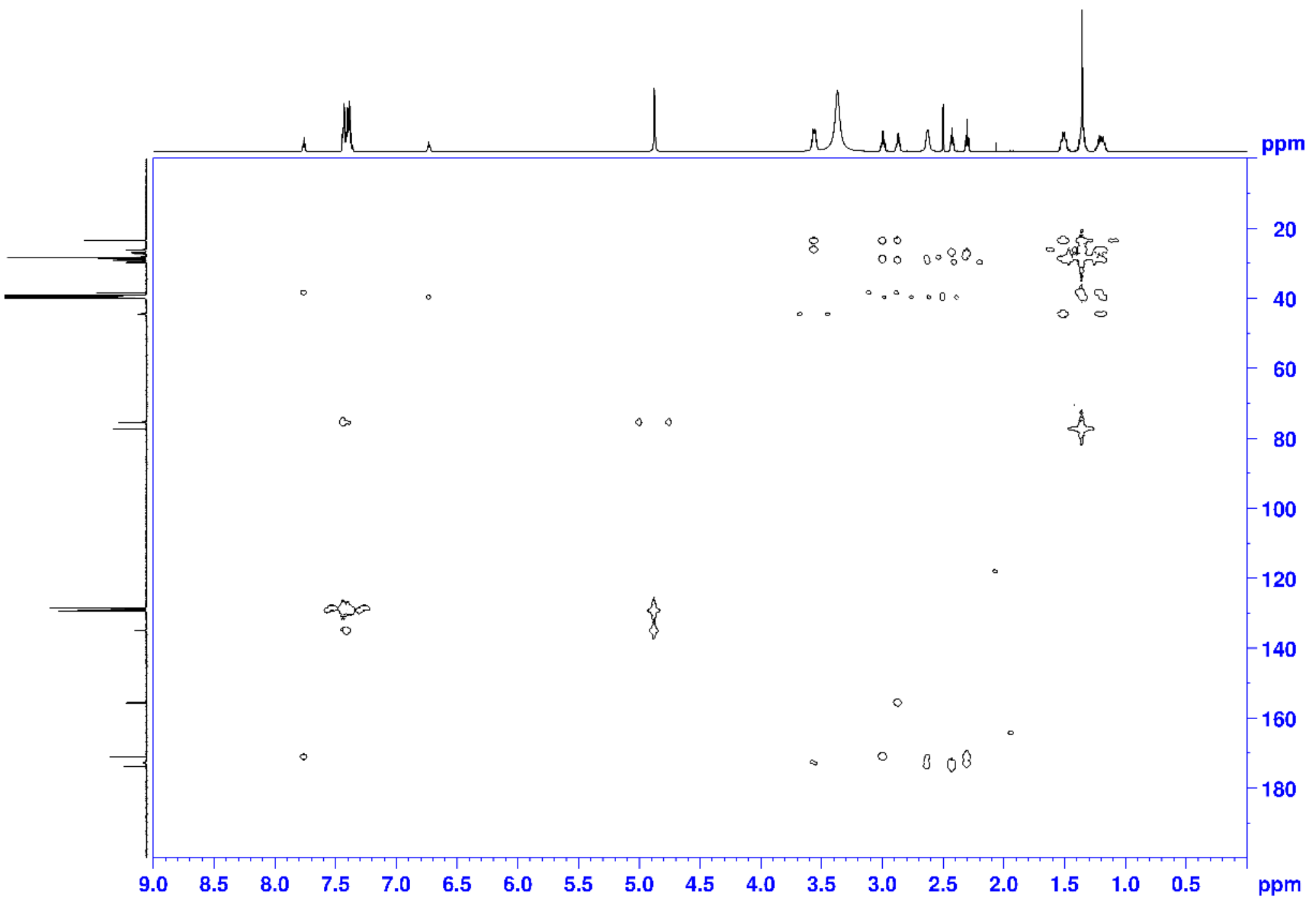

Figure S45. HMBC NMR (600 MHz, DMSO-d_6) spectrum of **S9**

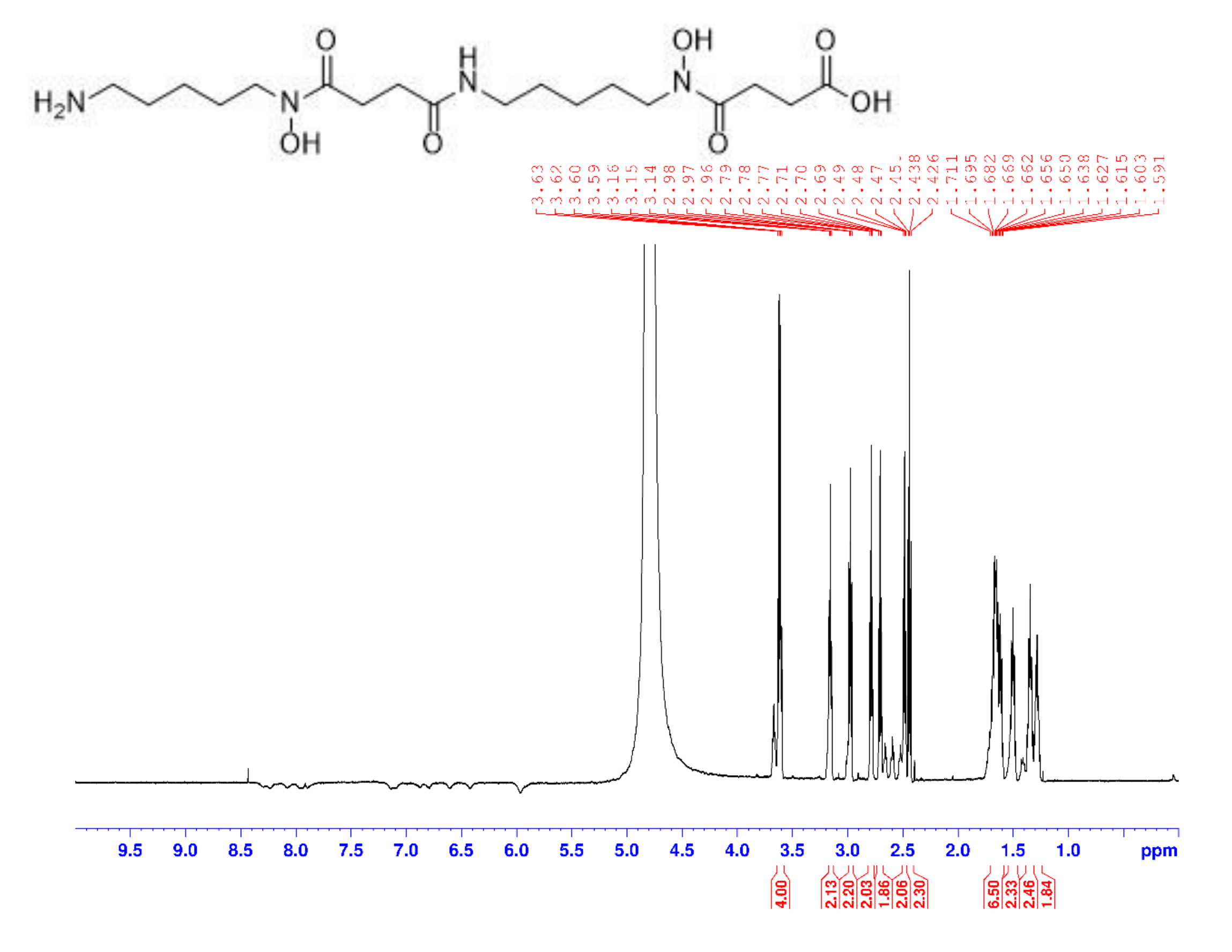

Figure S46. ^{1}H NMR (600 MHz, D_2O) spectrum of **3**

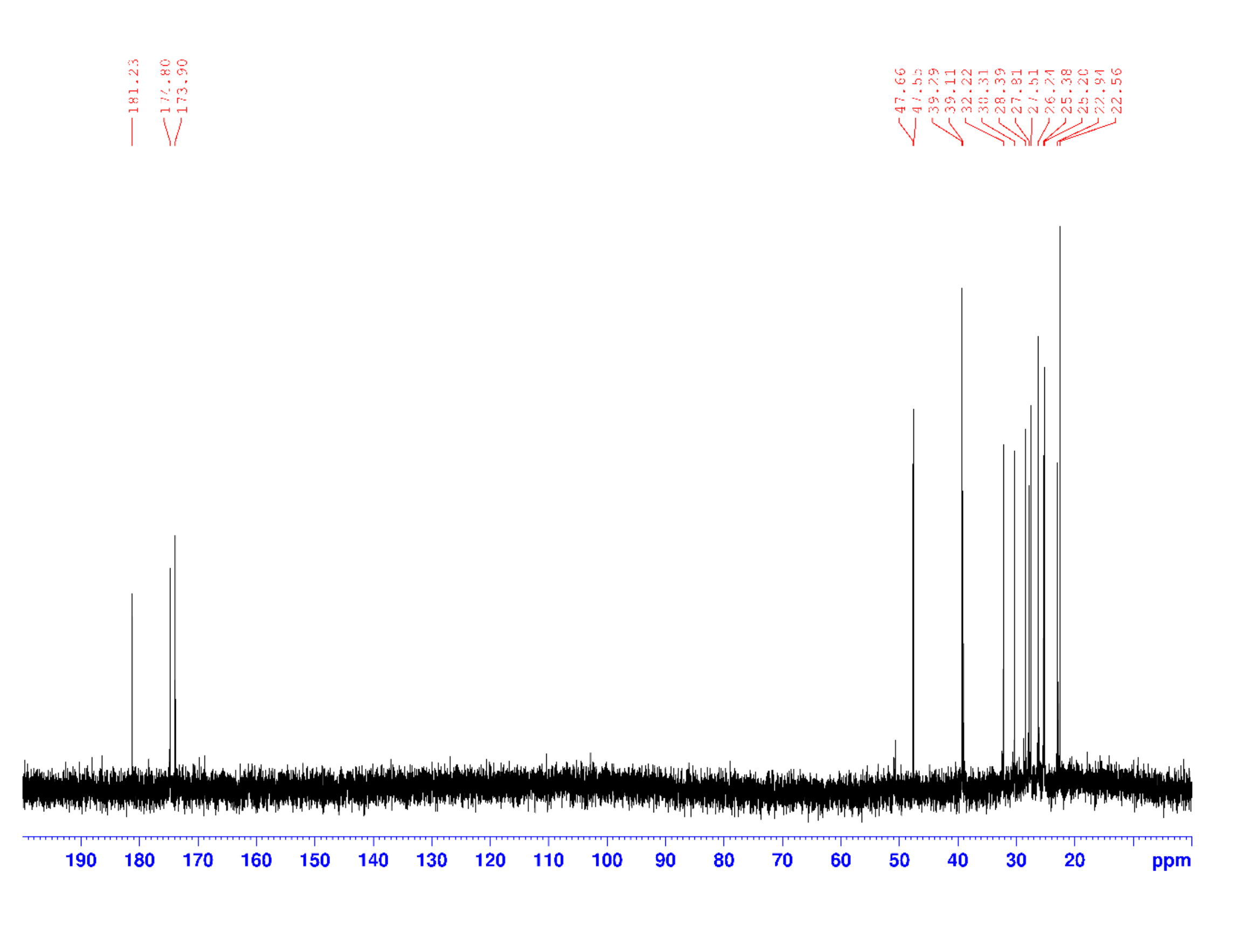

FigureS47. ^{13}C NMR (150 MHz, D_2O) spectrum of **3**

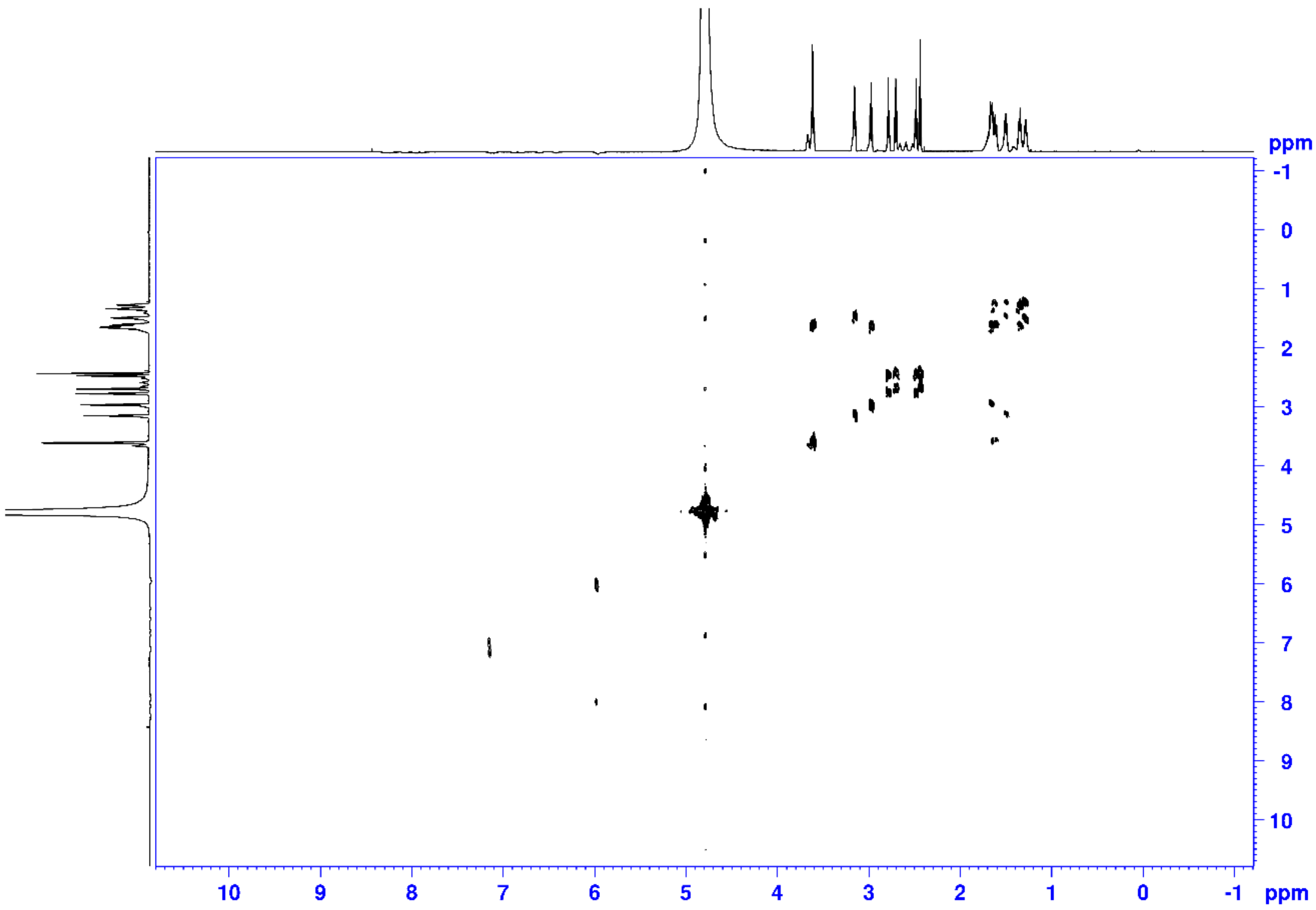

Figure S48. COSY NMR (600 MHz, D_2O) spectrum of **3**

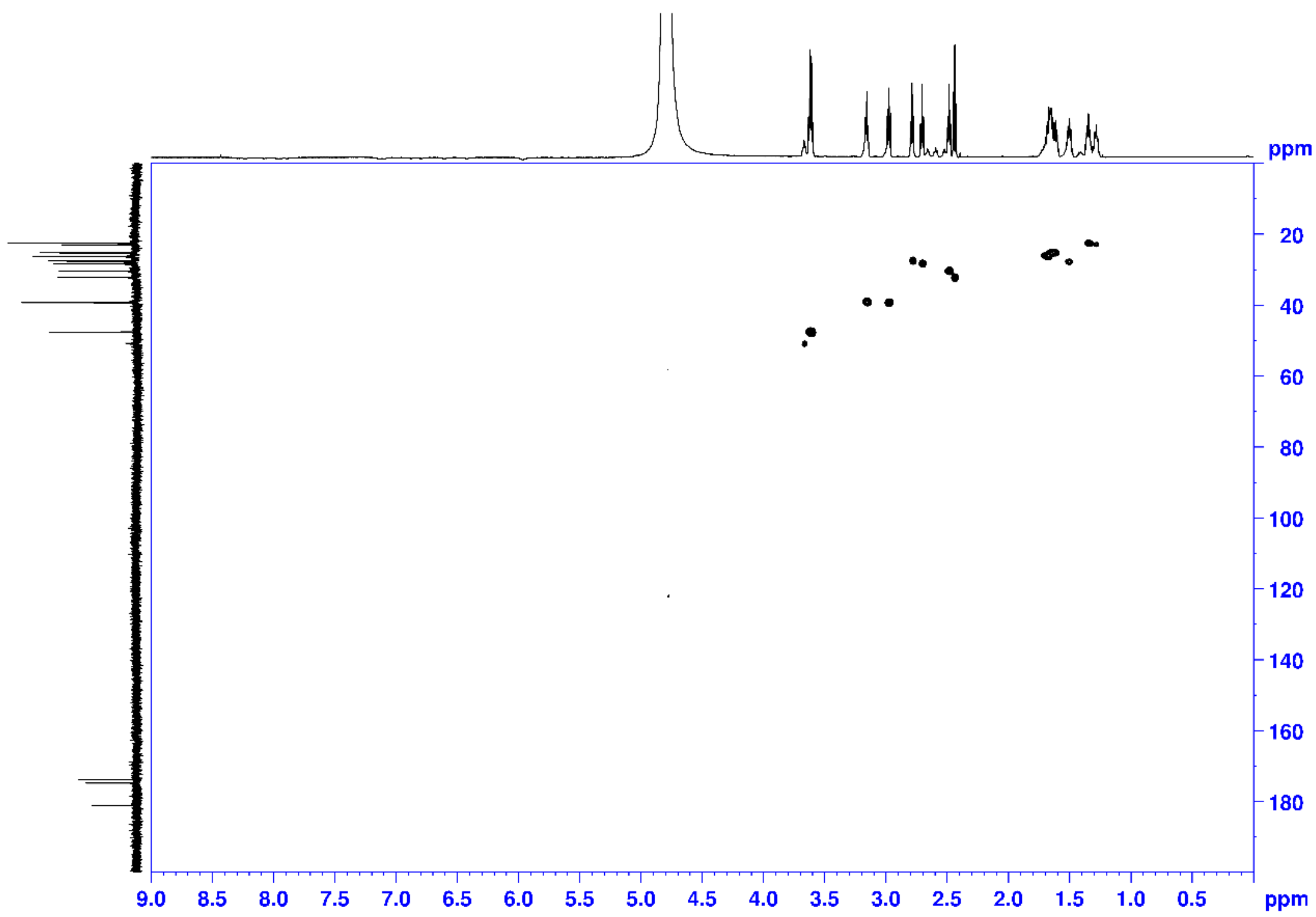

Figure S49. HSQC NMR (600 MHz, D_2O) spectrum of **3**

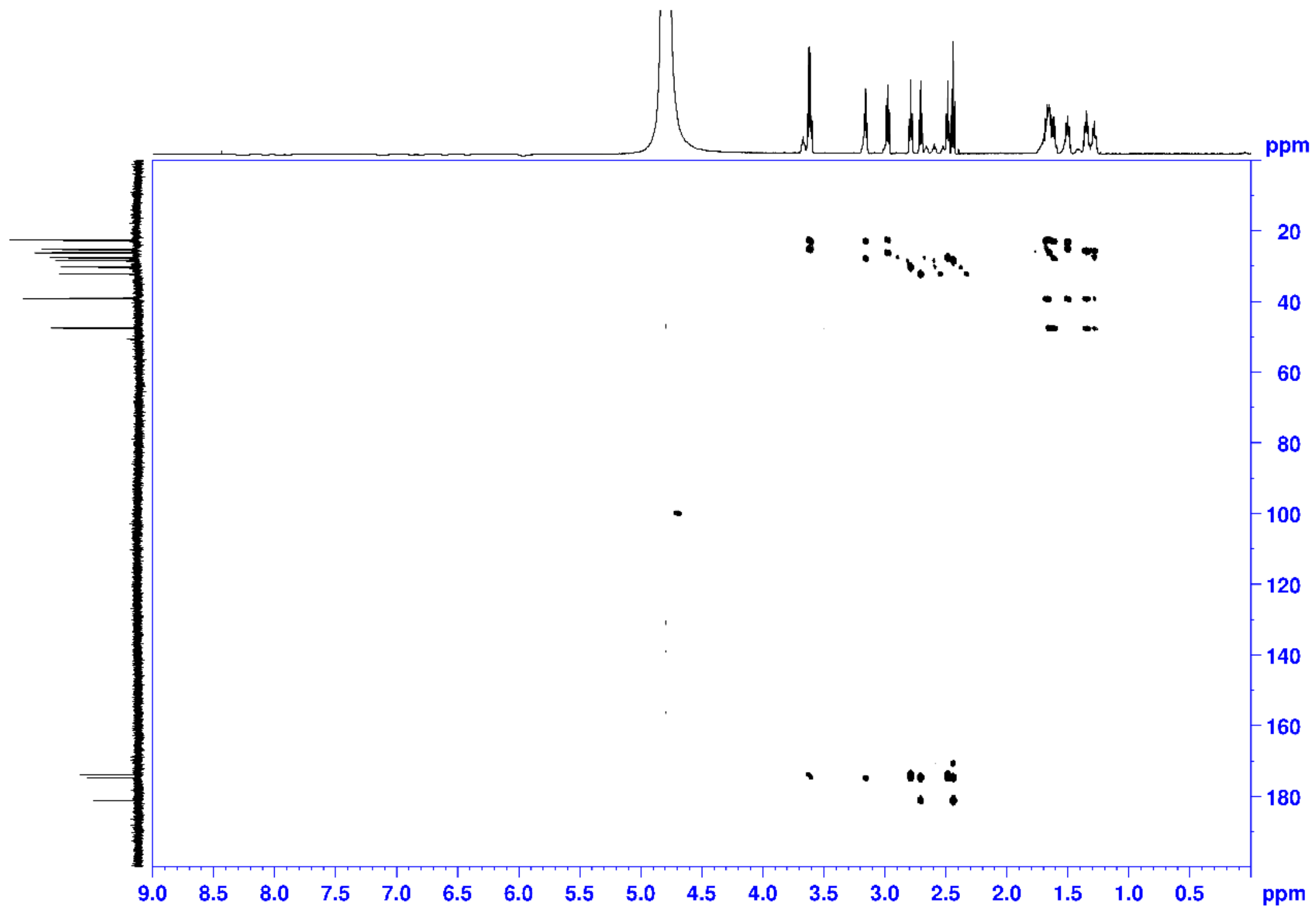

Figure S50. HMBC NMR (600 MHz, D_2O) spectrum of **3**

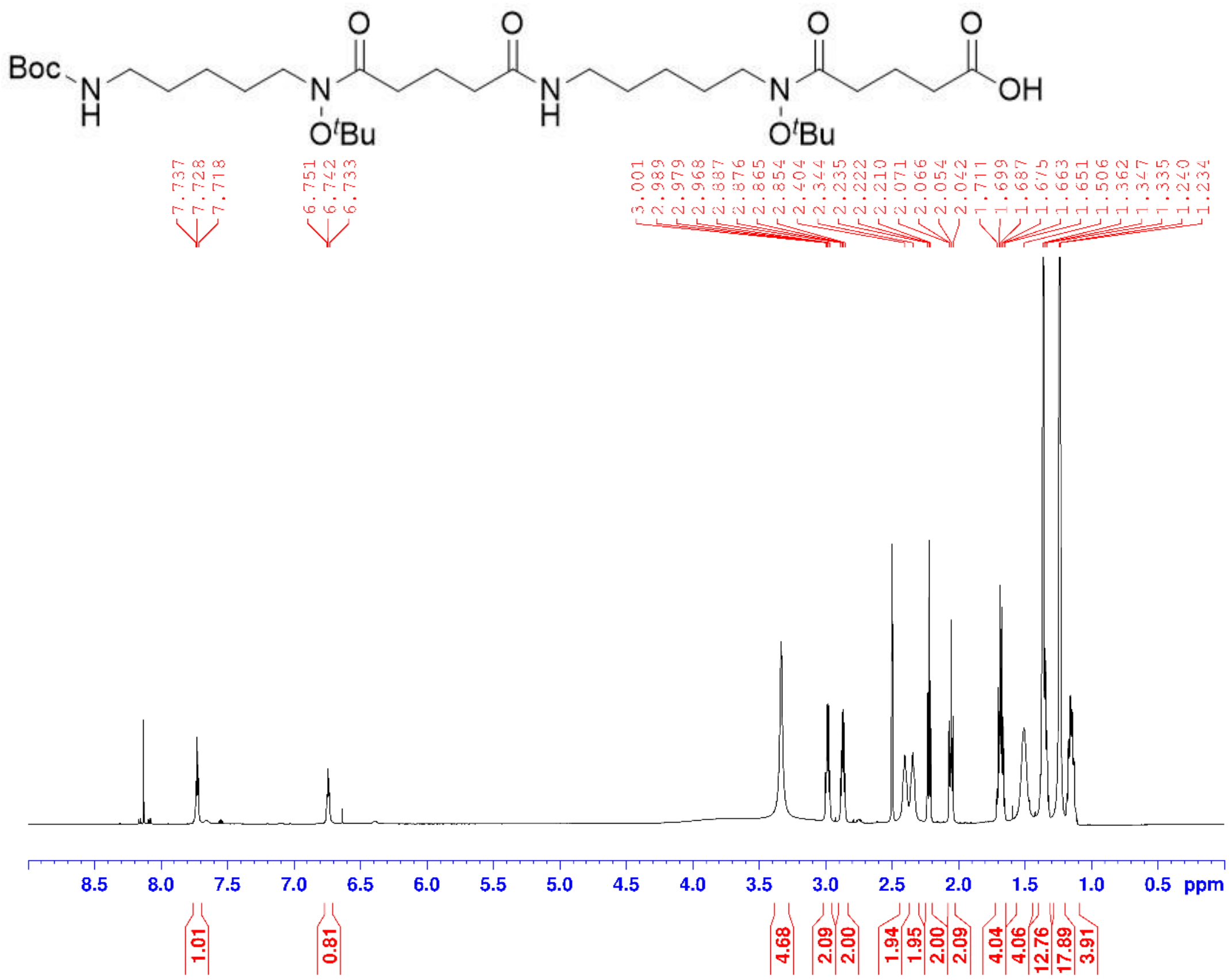

Figure S51. ^{1}H NMR (600 MHz, DMSO-d_6) spectrum of **S10**

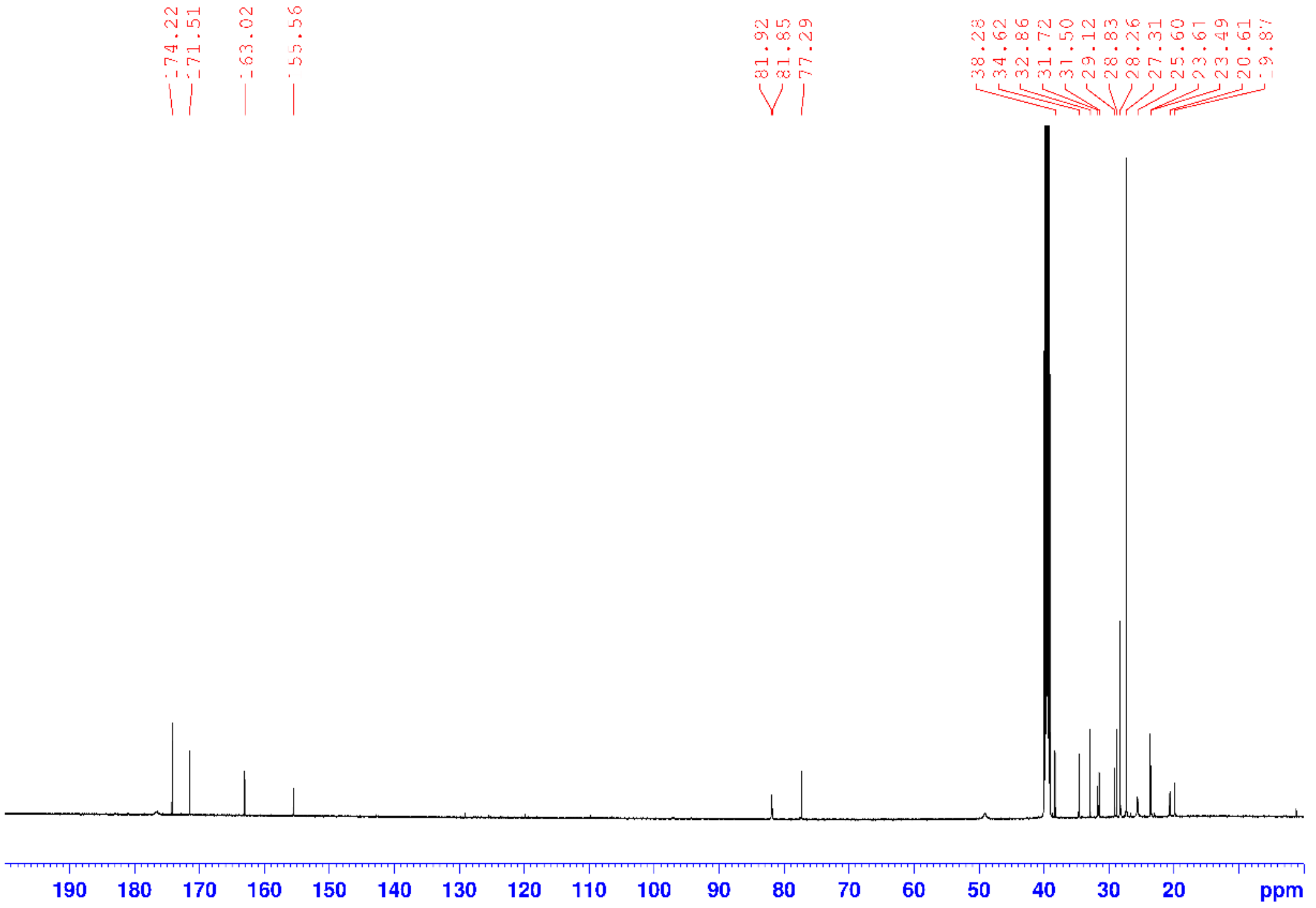

Figure S52. ^{13}C NMR (150 MHz, DMSO-d_6) spectrum of **S10**

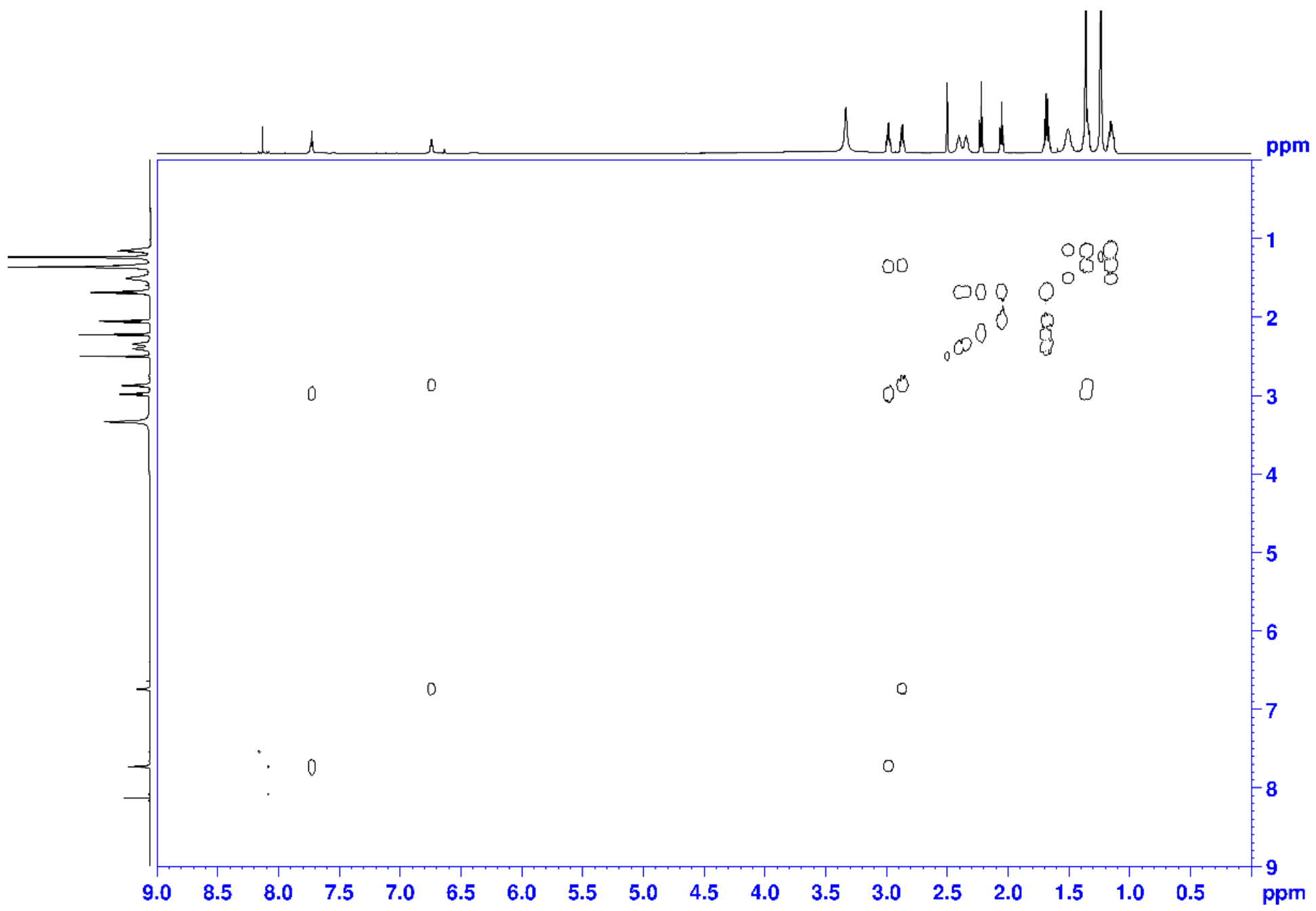

Figure S53. COSY NMR (600 MHz, DMSO-d_6) spectrum of **S10**

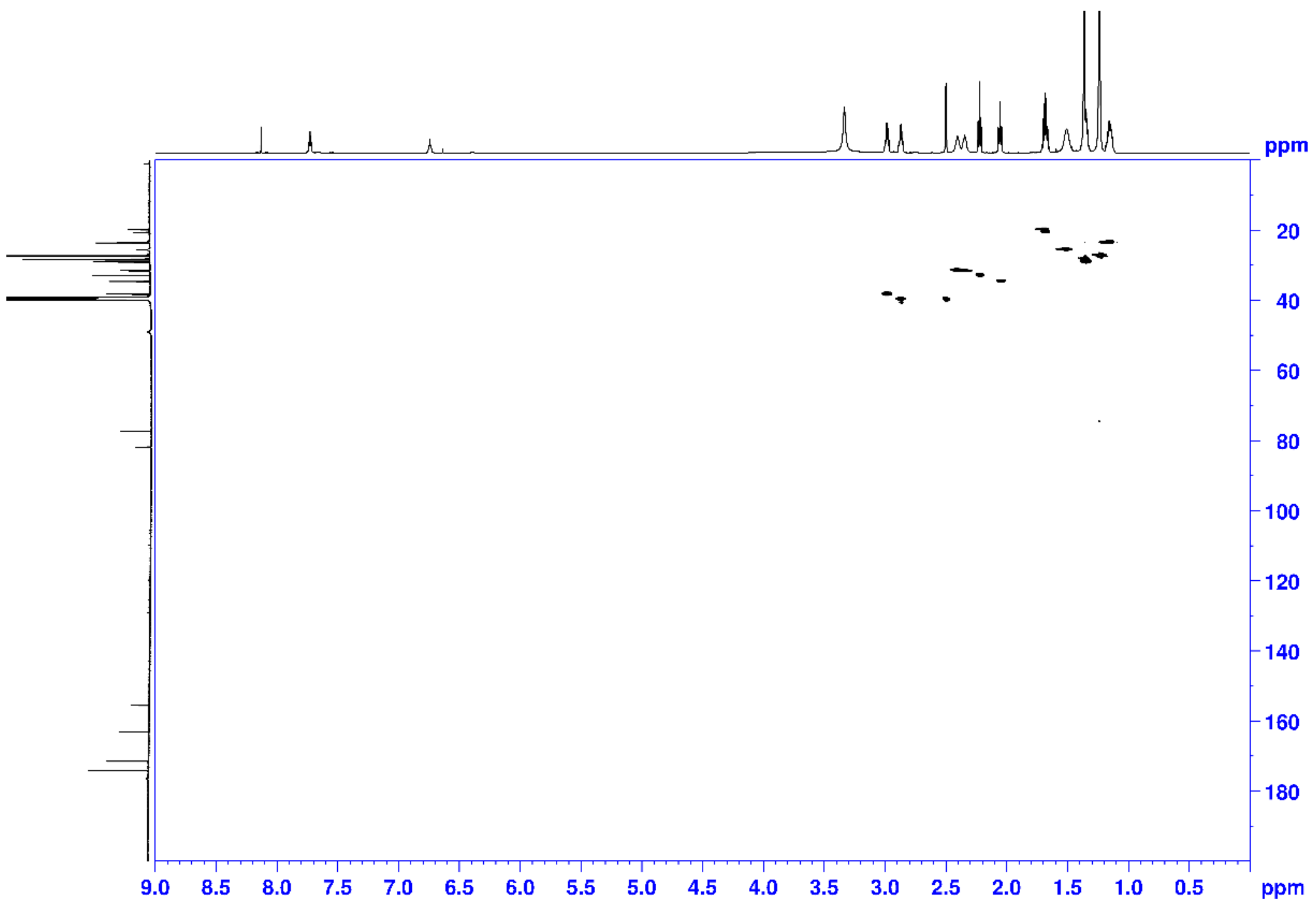

Figure S54. HSQC NMR (600 MHz, DMSO-d_6) spectrum of **S10**

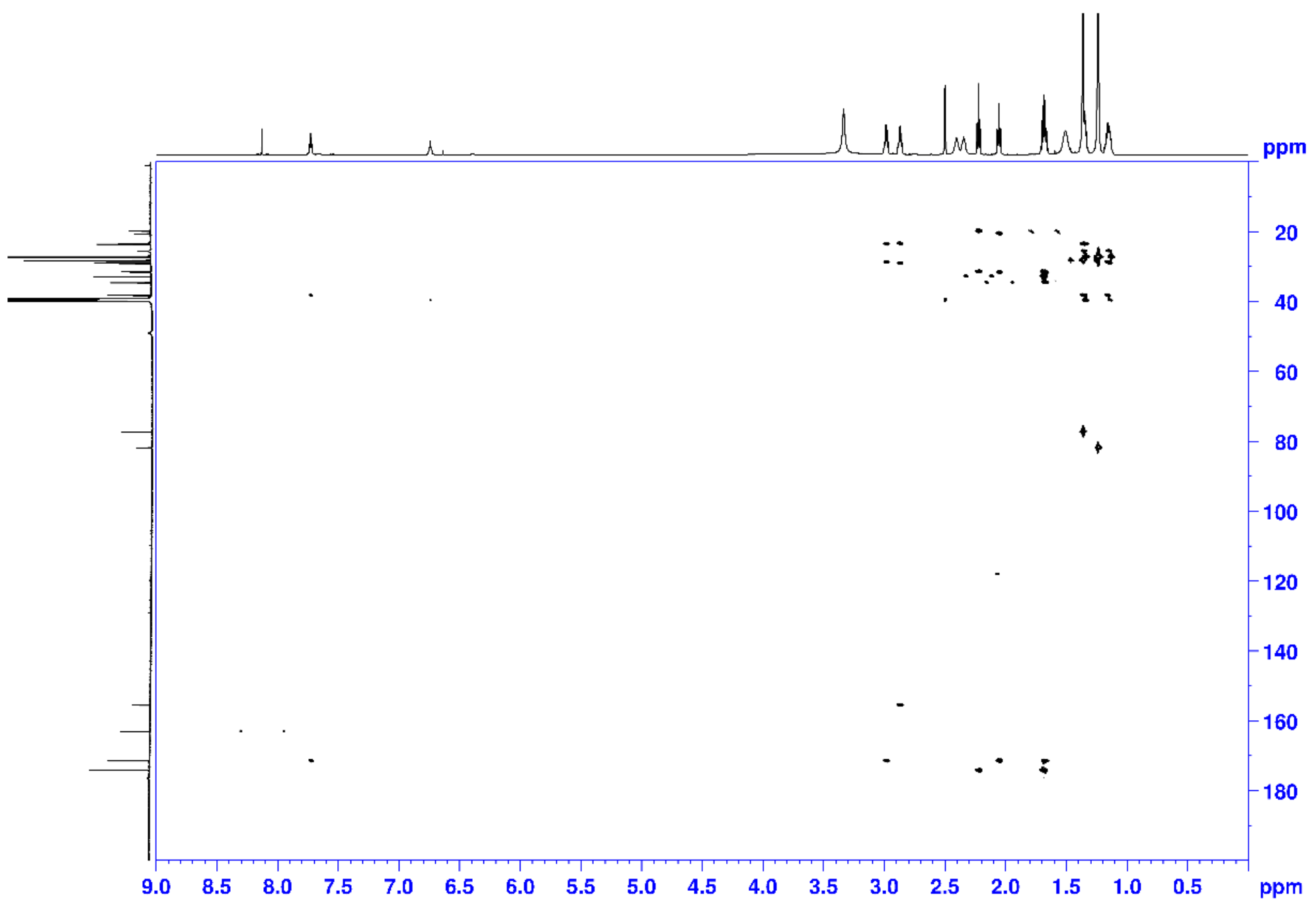

Figure S55. HMBC NMR (600 MHz, DMSO-d_6) spectrum of **S10**

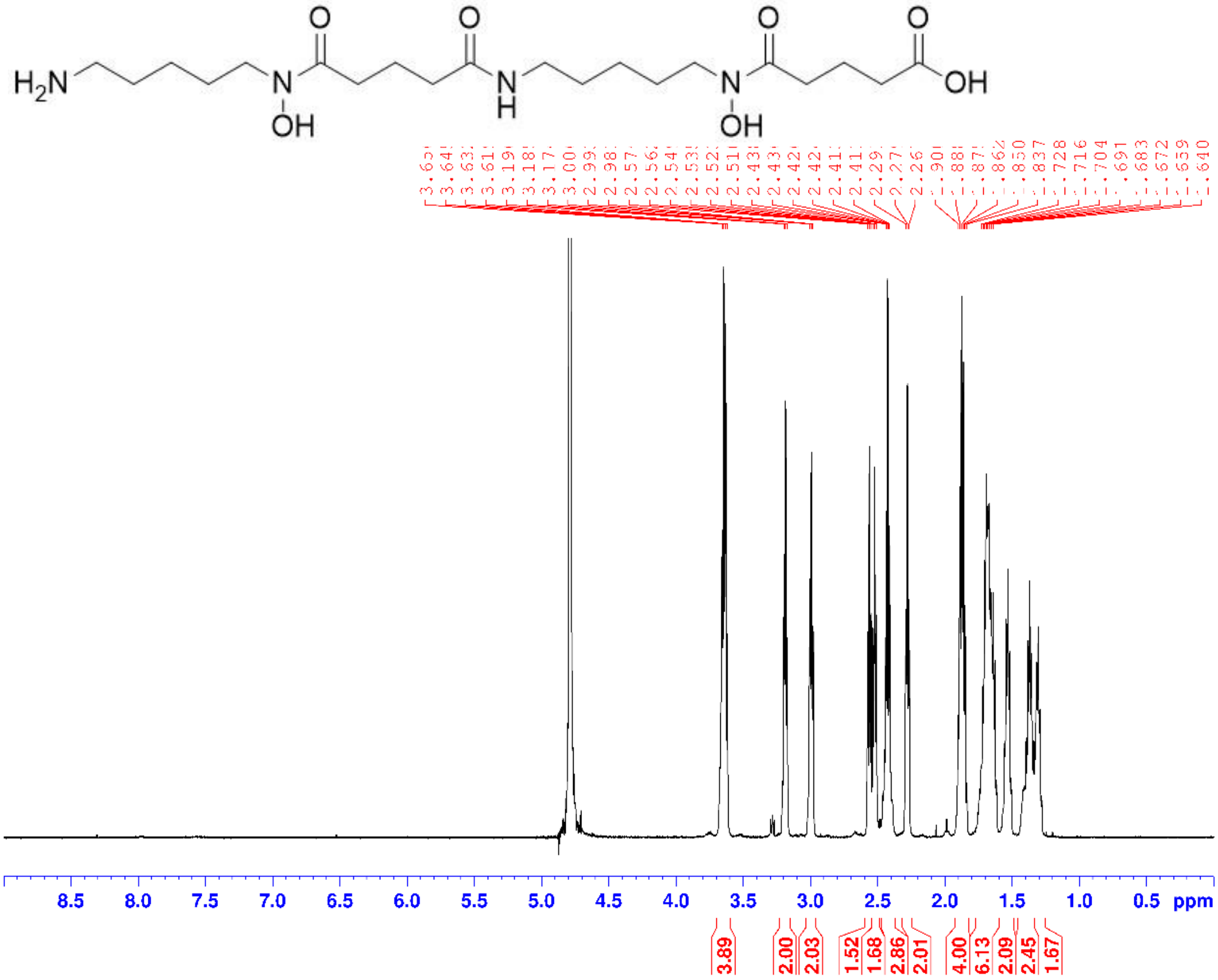

Figure S56. ^{1}H NMR (600 MHz, D_2O) spectrum of **4**

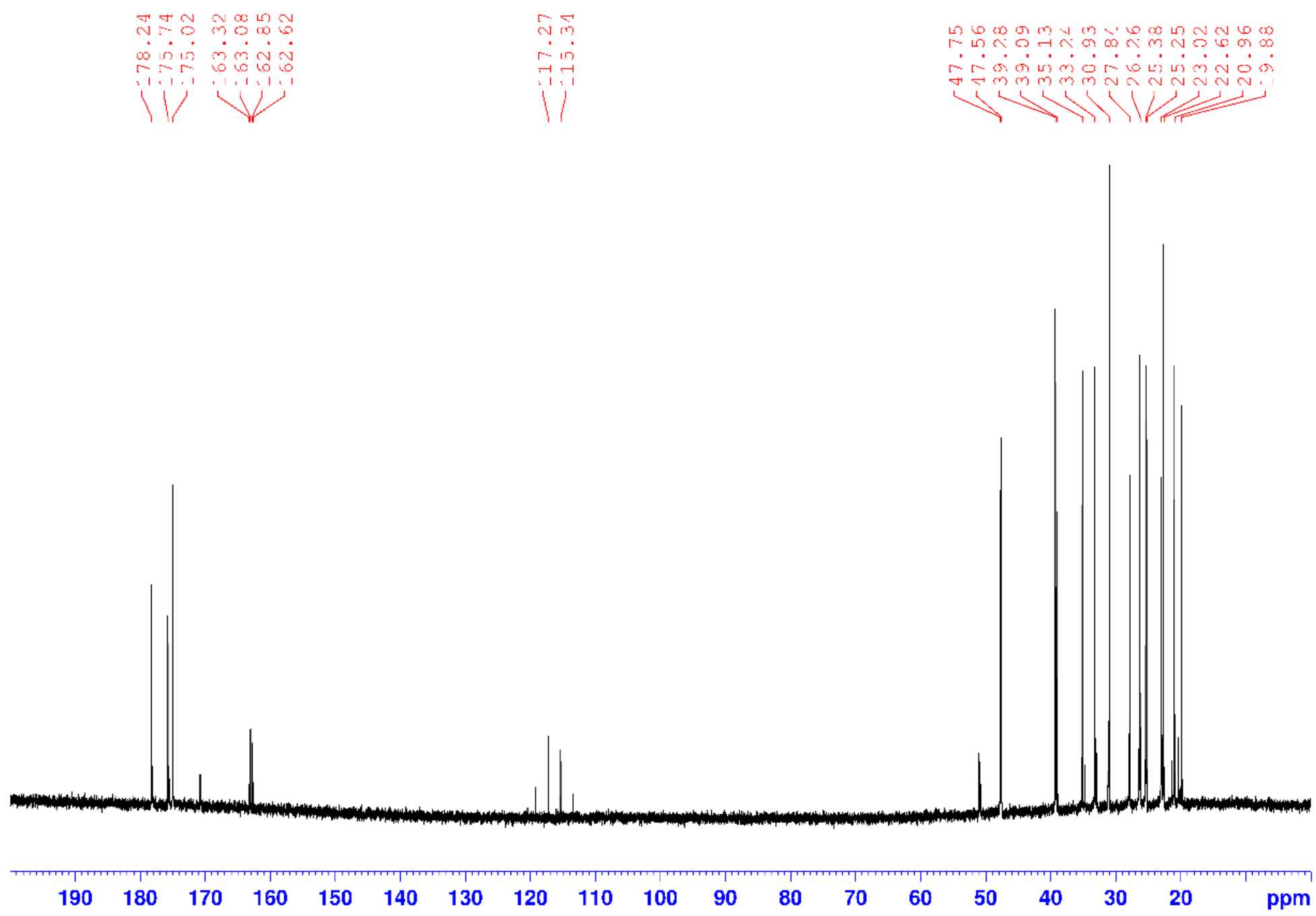

Figure S57. ^{13}C NMR (150 MHz, D_2O) spectrum of **4**. Overlapping signal at 178.24 ppm and 30.93 ppm.

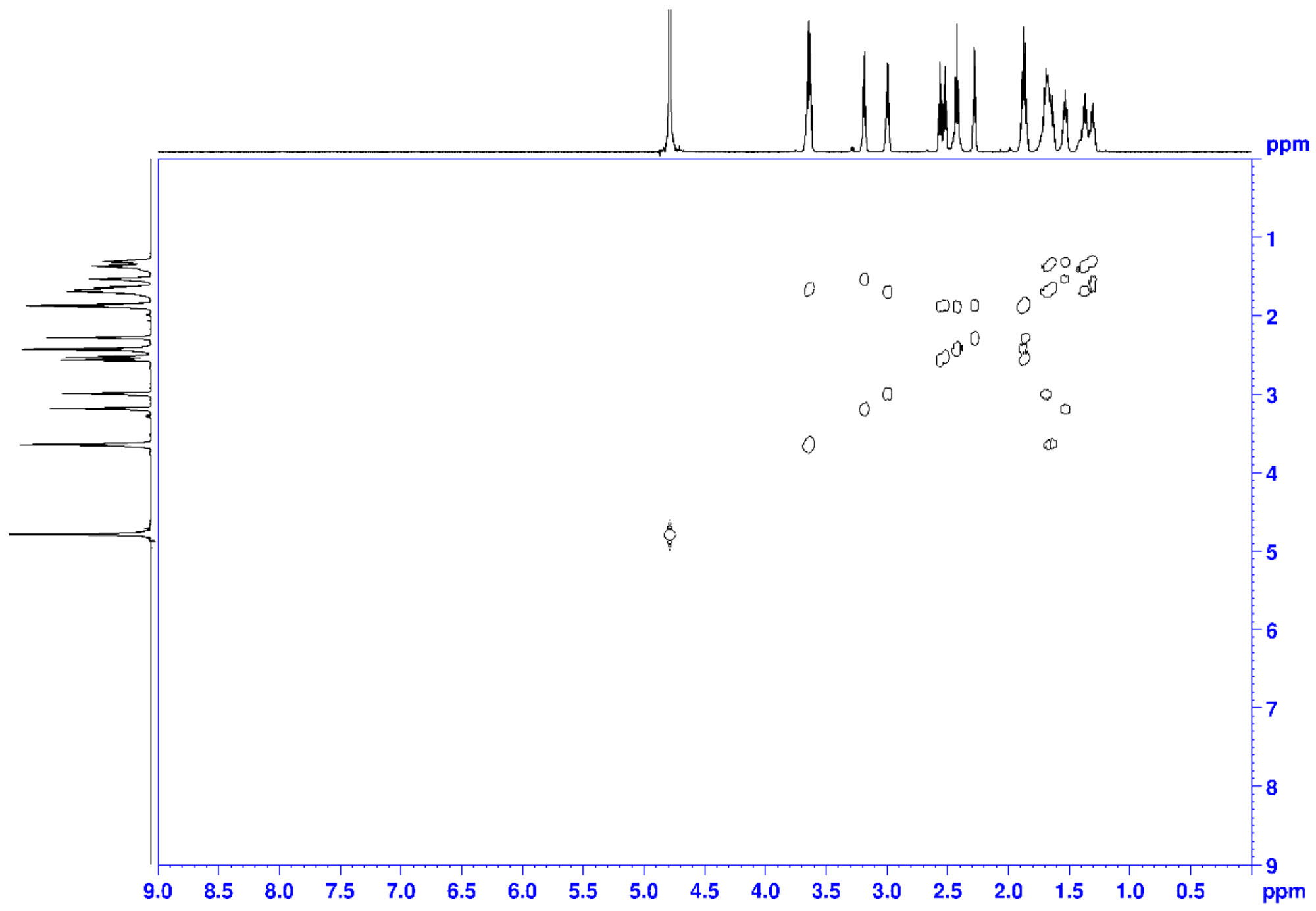

Figure S58. COSY NMR (600 MHz, D_2O) spectrum of **4**

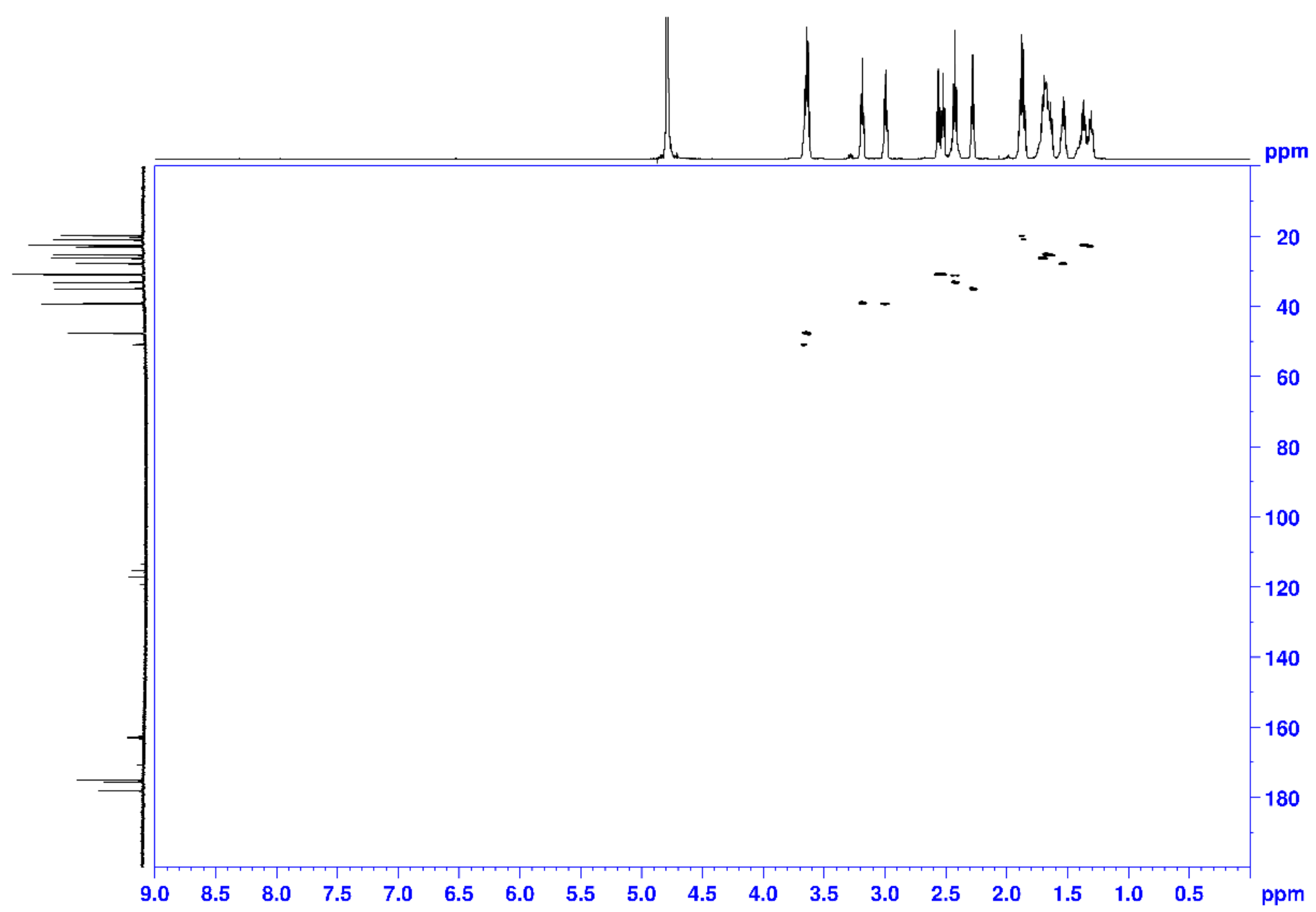

Figure S59. HSQC NMR (600 MHz, D_2O) spectrum of **4**

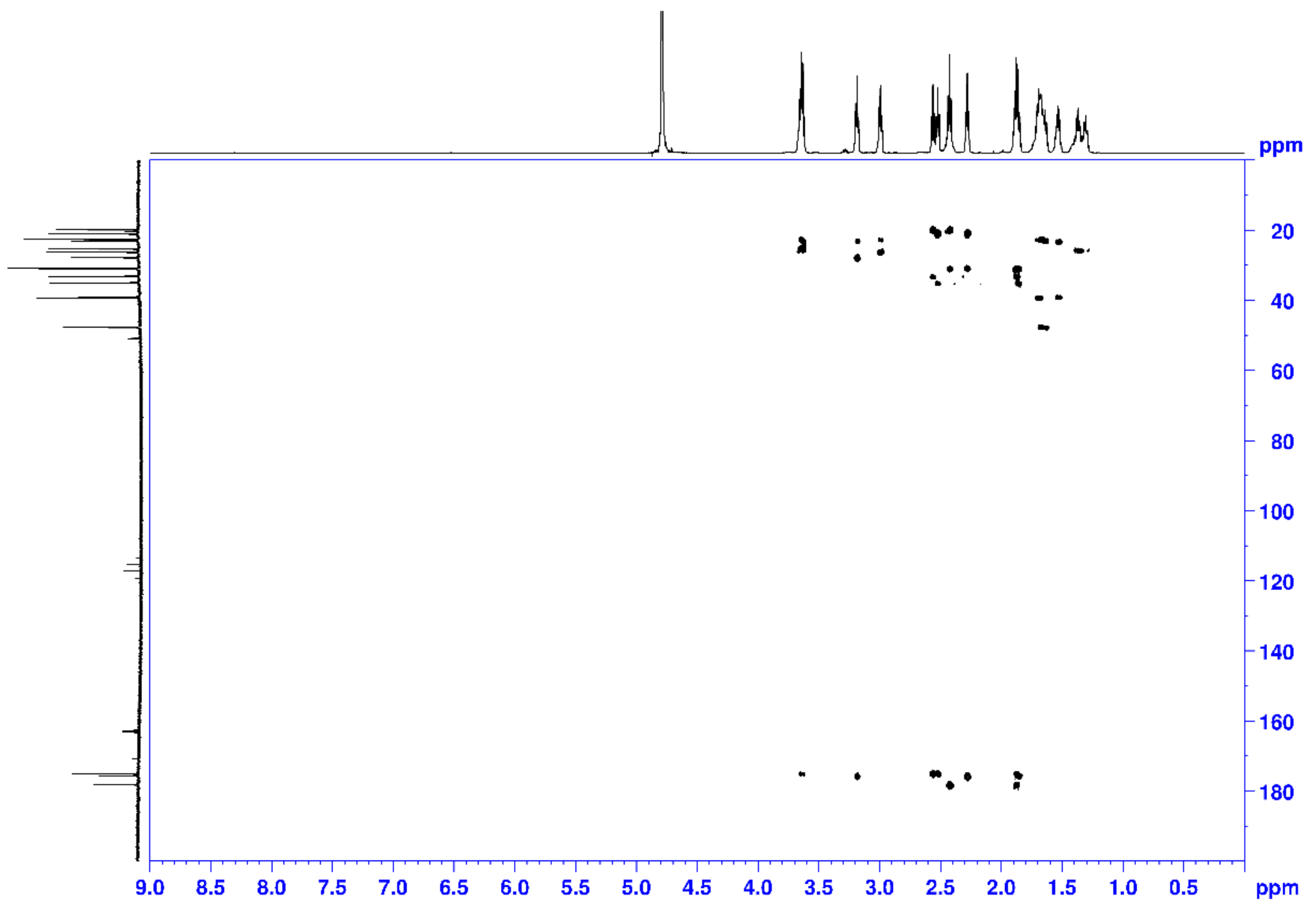

Figure S60. HMBC NMR (600 MHz, D_2O) spectrum of **4**

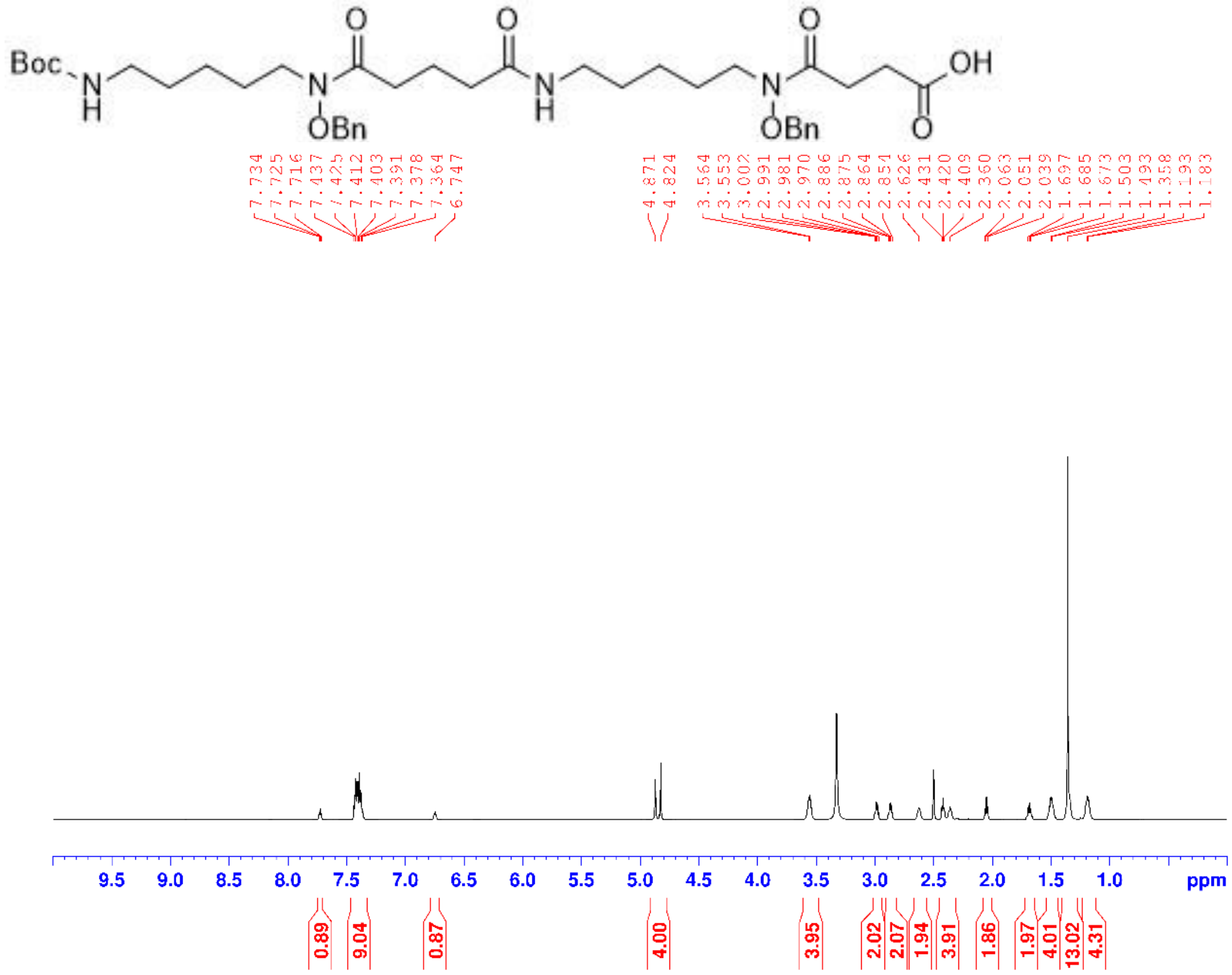

Figure S61. ^{1}H NMR (600 MHz, DMSO-d_6) spectrum of **S11**

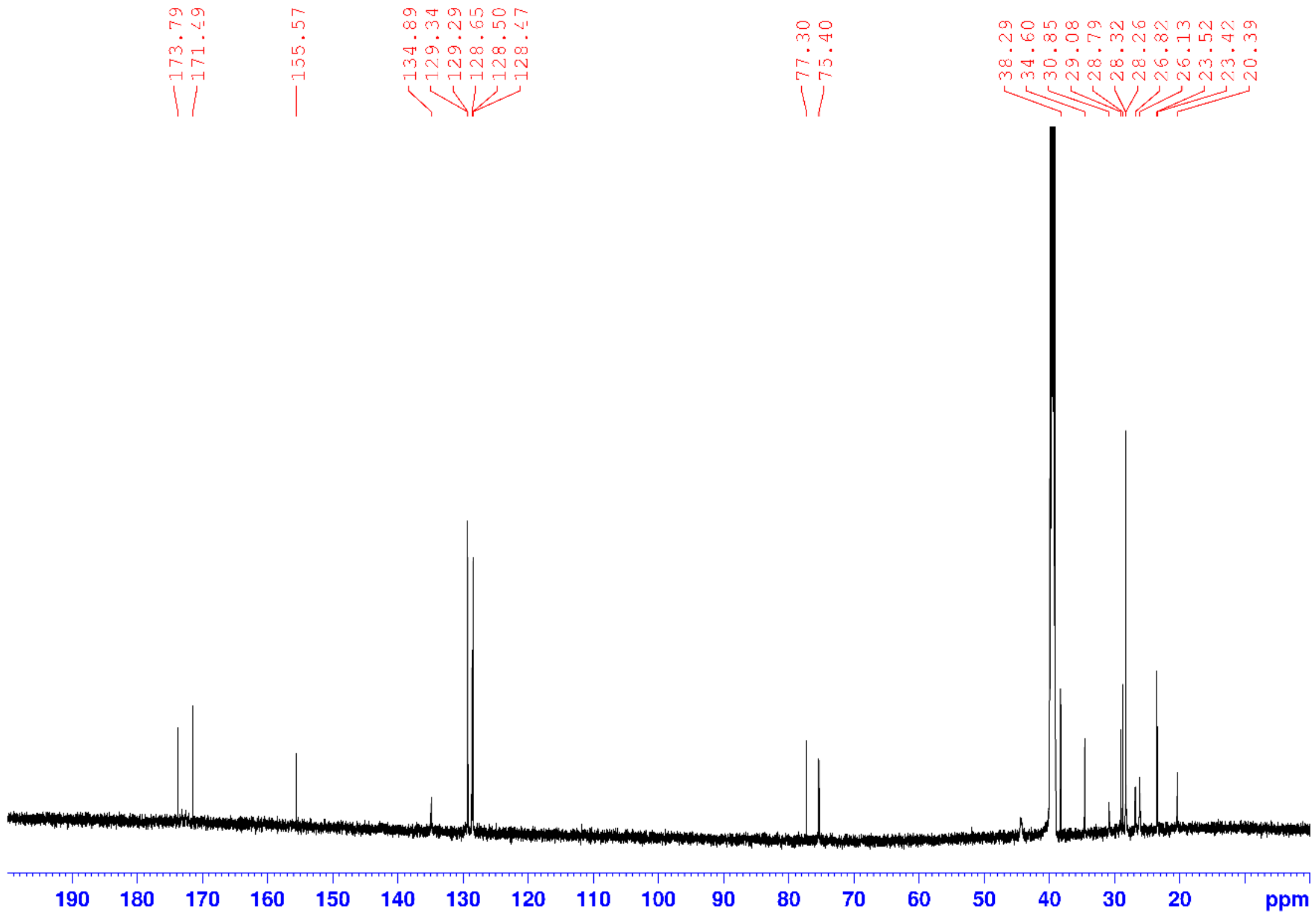

Figure S62. ^{13}C NMR (150 MHz, DMSO-d_6) spectrum of **S11**

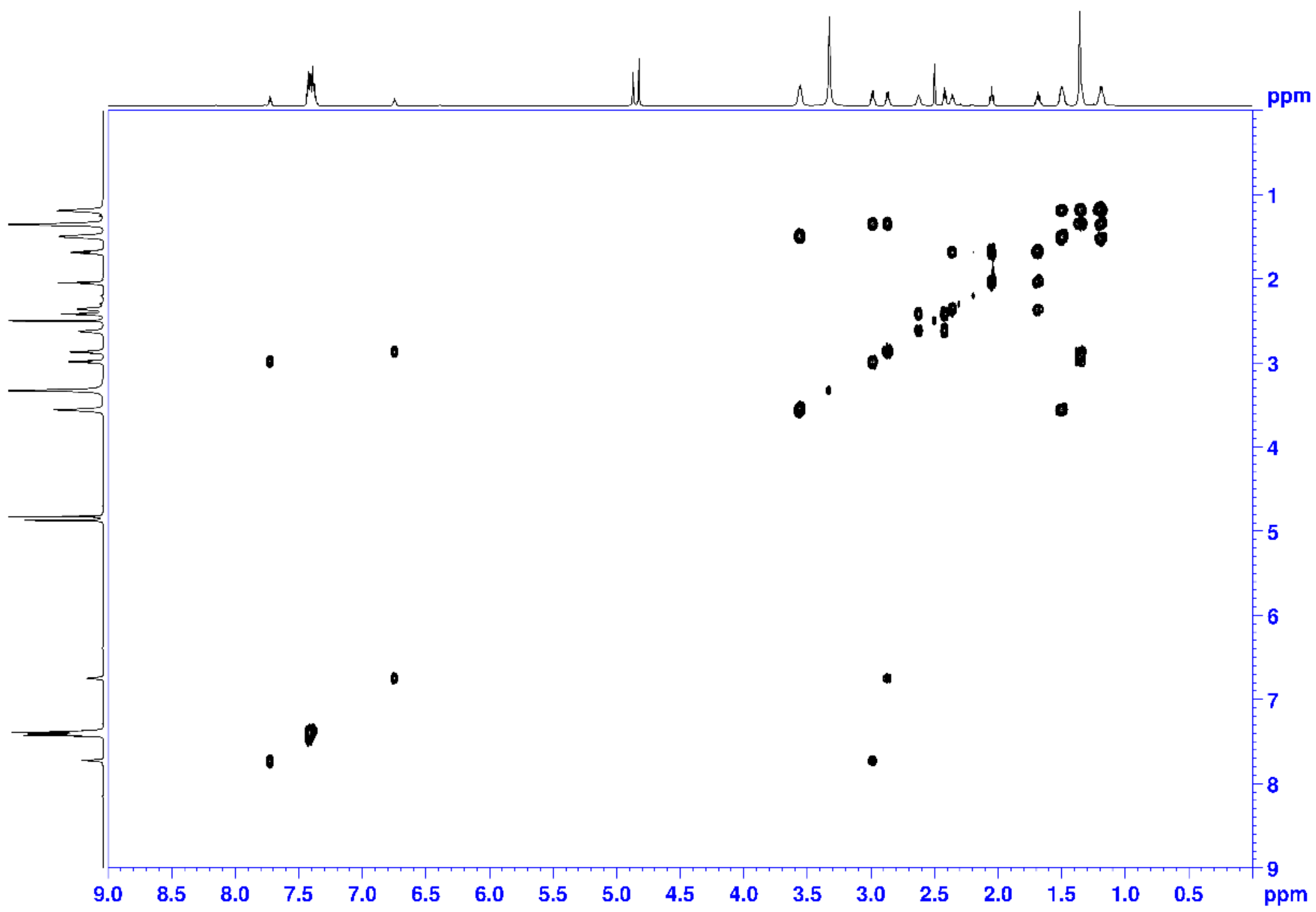

Figure S63. COSY NMR (600 MHz, DMSO-d_6) spectrum of **S11**

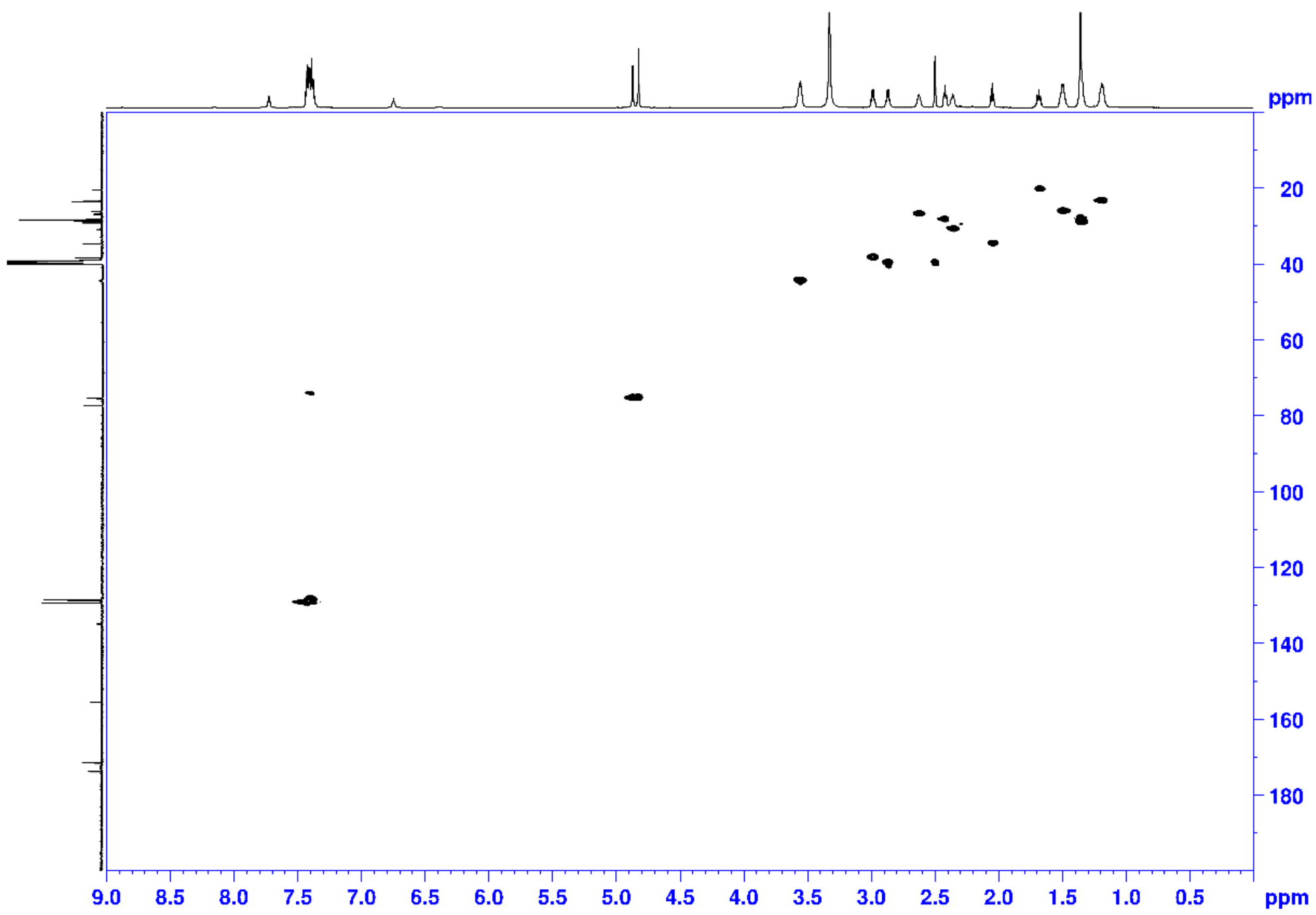

Figure S64. HSQC NMR (600 MHz, DMSO-d_6) spectrum of **S11**

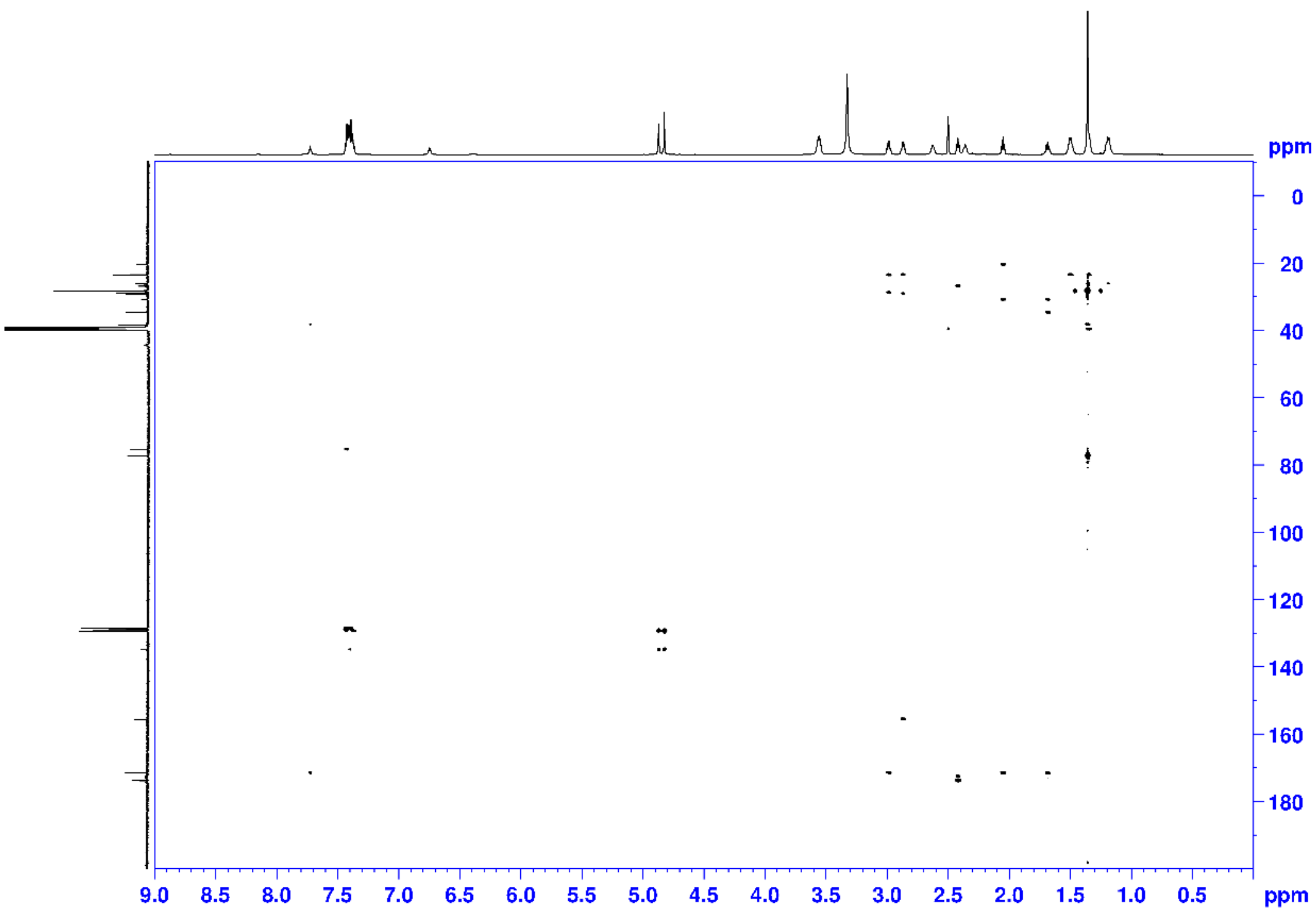

Figure S65. HMBC NMR (600 MHz, DMSO-d_6) spectrum of **S11**

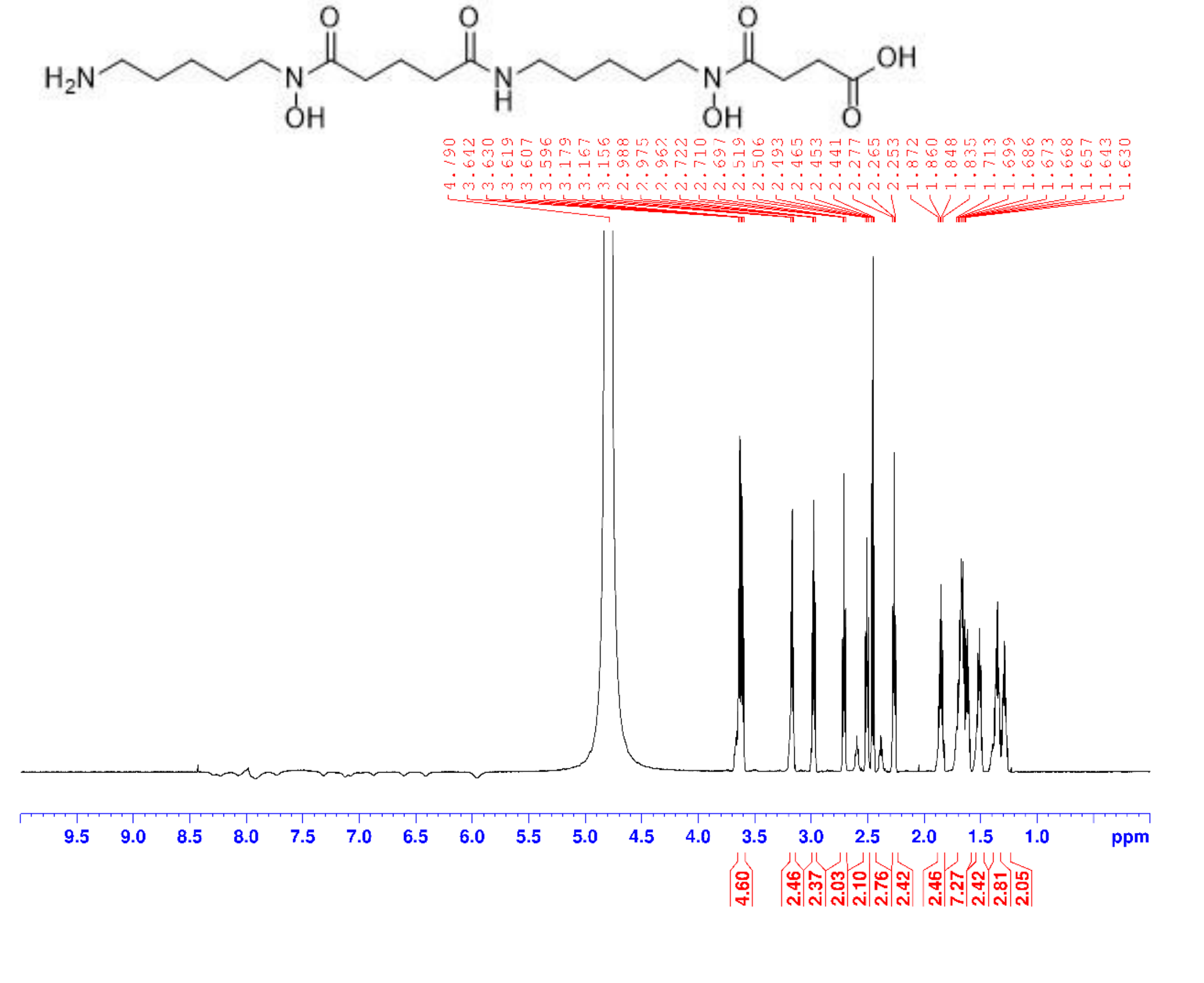

Figure S66. ^1H NMR (600 MHz, D_2O) spectrum of **5**

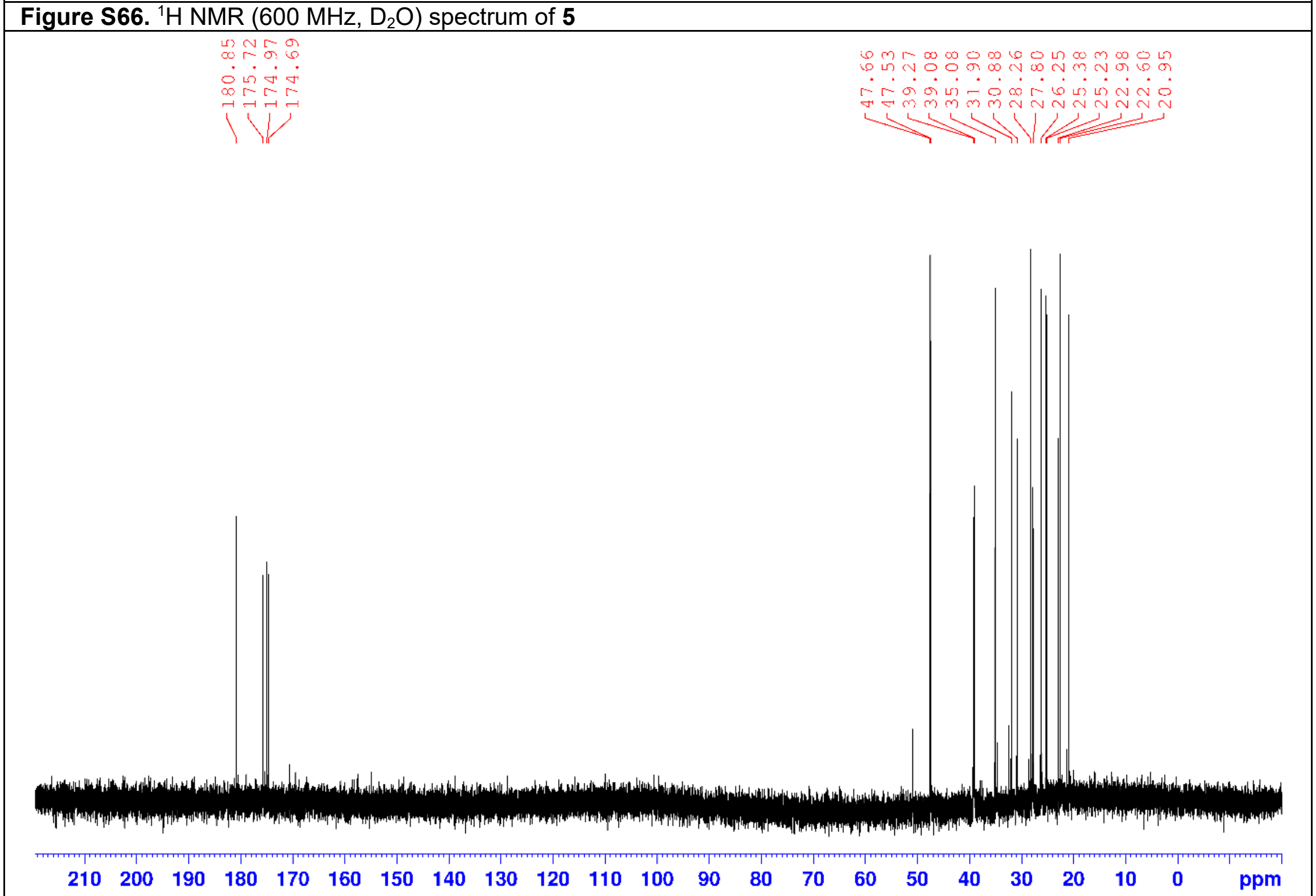

Figure S67. ^{13}C NMR (150 MHz, D_2O) spectrum of **5**. Mixture of E/Z conformers additional signal at 50.92 ppm.

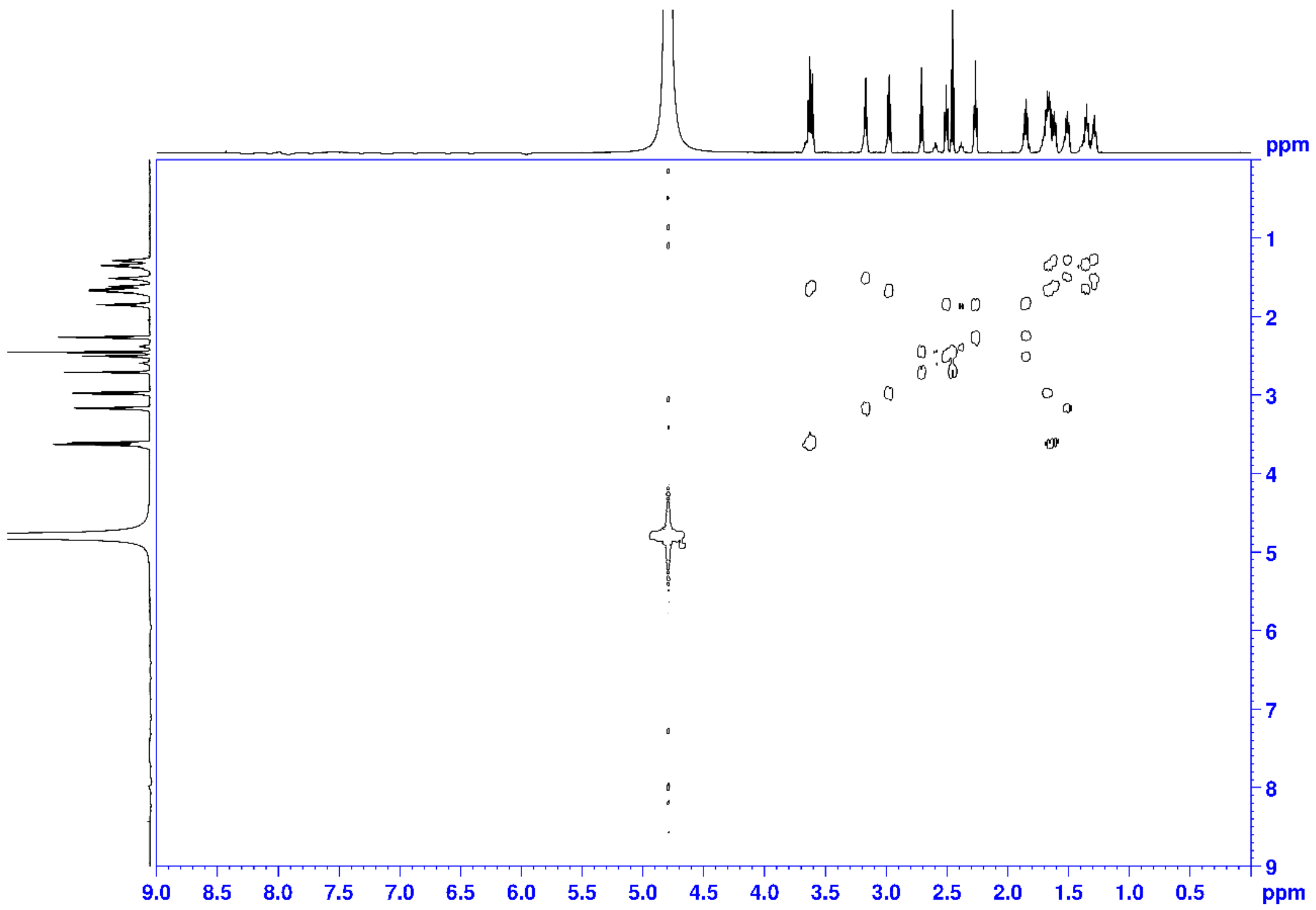

Figure S68. COSY NMR (600 MHz, D_2O) spectrum of **5**

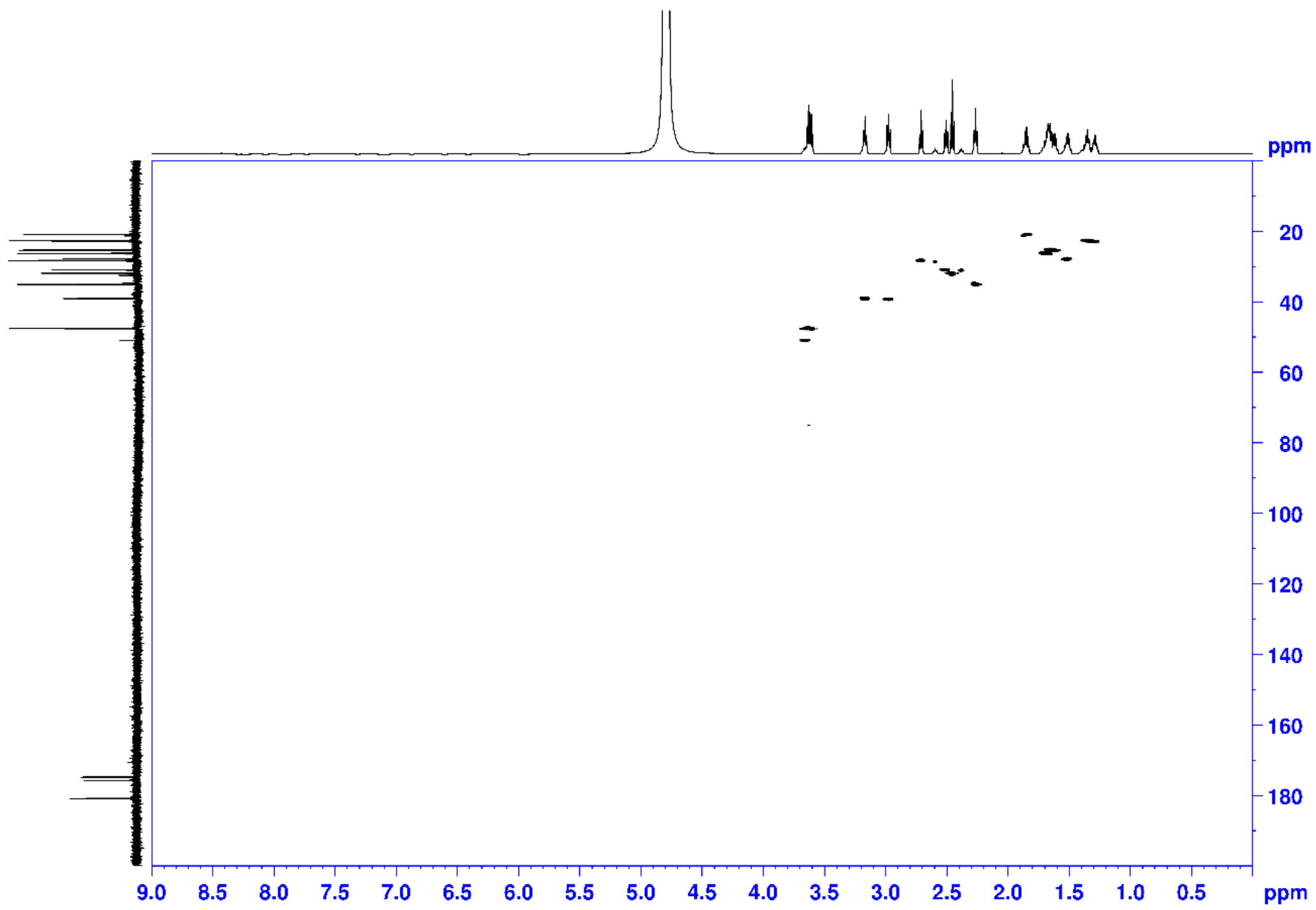

Figure S69. HSQC NMR (600 MHz, D_2O) spectrum of **5**

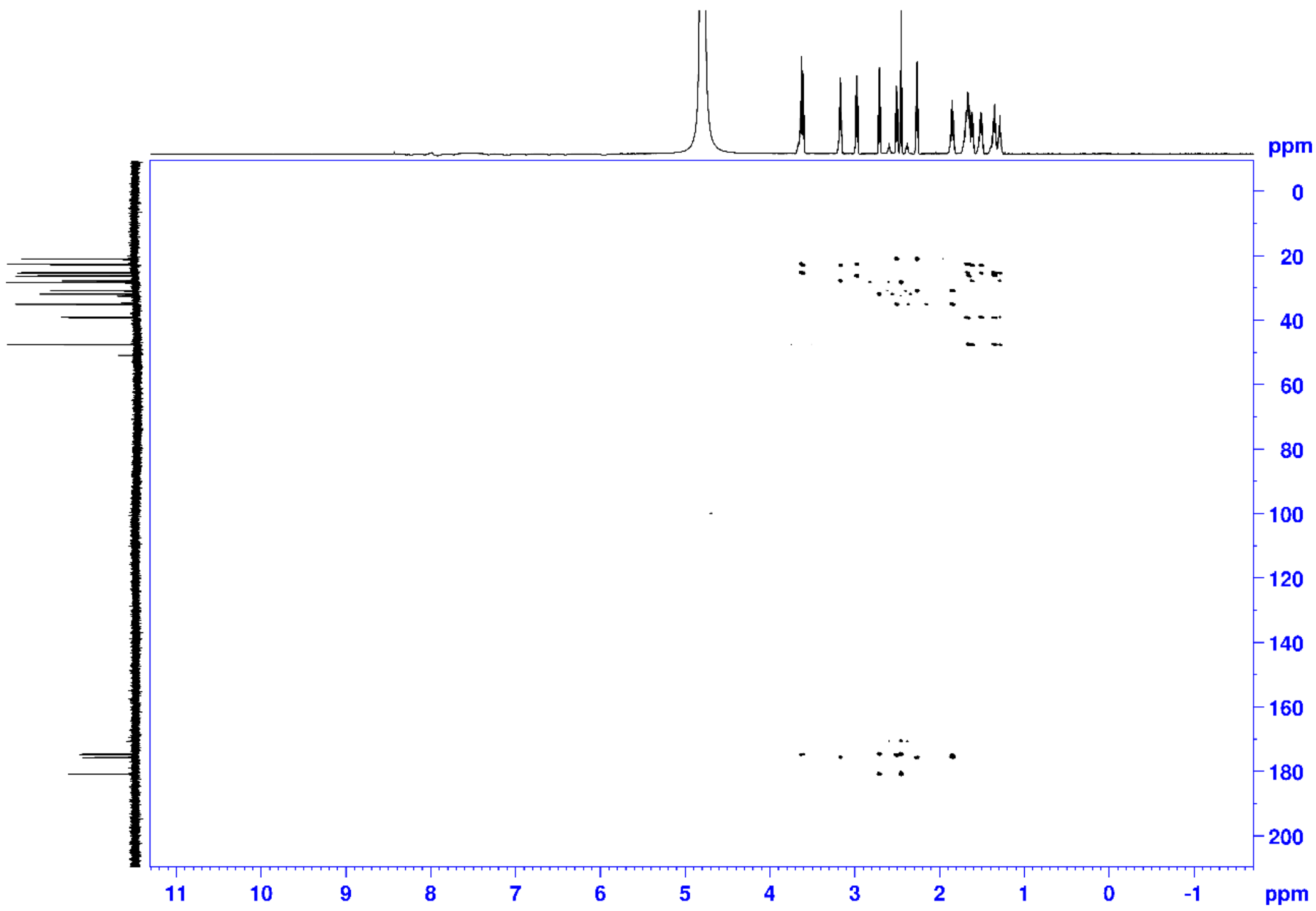

Figure S70. HMBC NMR (600 MHz, D_2O) spectrum of **5**

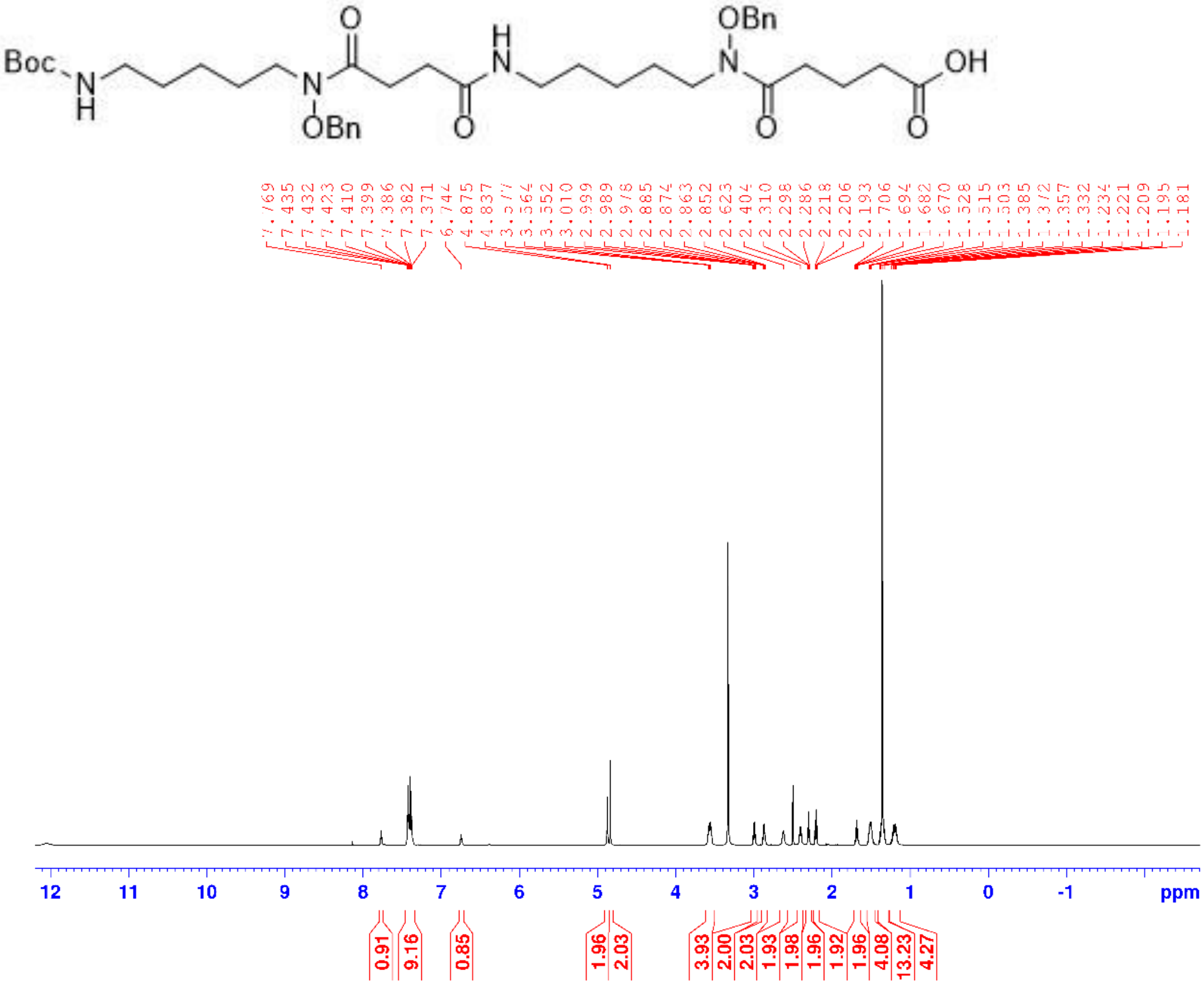

Figure S71. ^{1}H NMR (600 MHz, DMSO-d_6) spectrum of **S12**

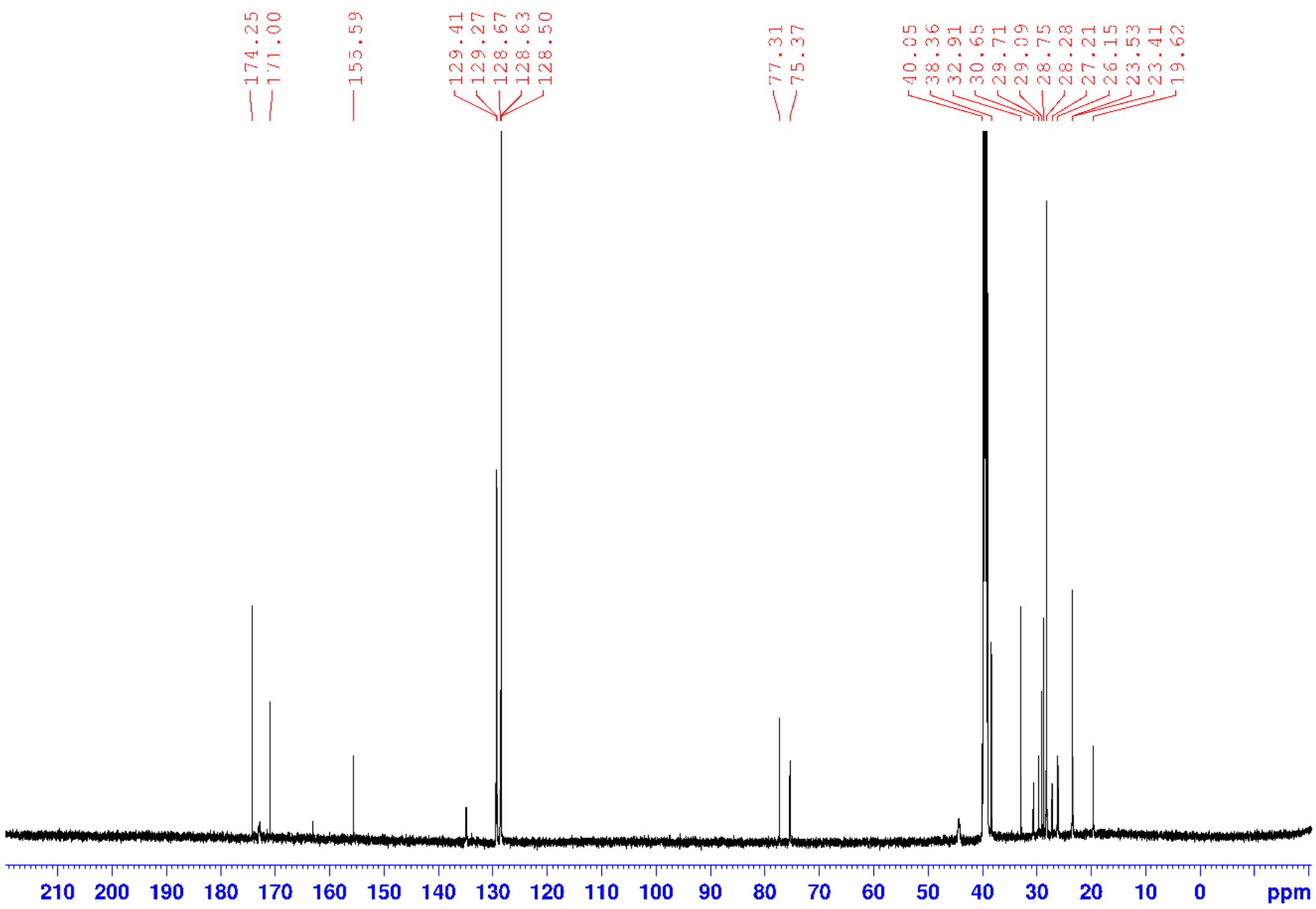

Figure S72. ^{13}C NMR (150 MHz, DMSO-d_6) spectrum of **S12**

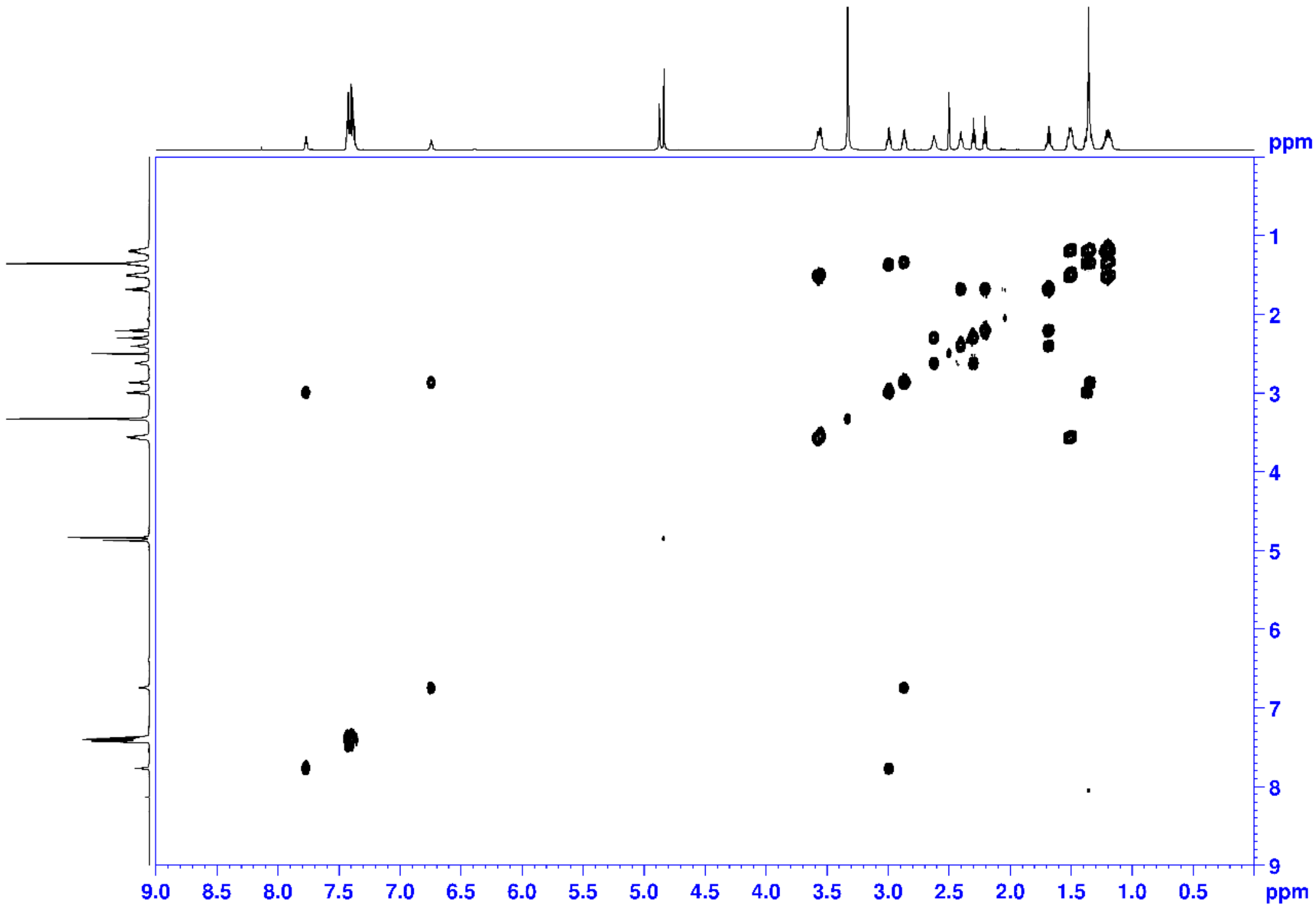

Figure S73. COSY NMR (600 MHz, DMSO-d_6) spectrum of **S12**

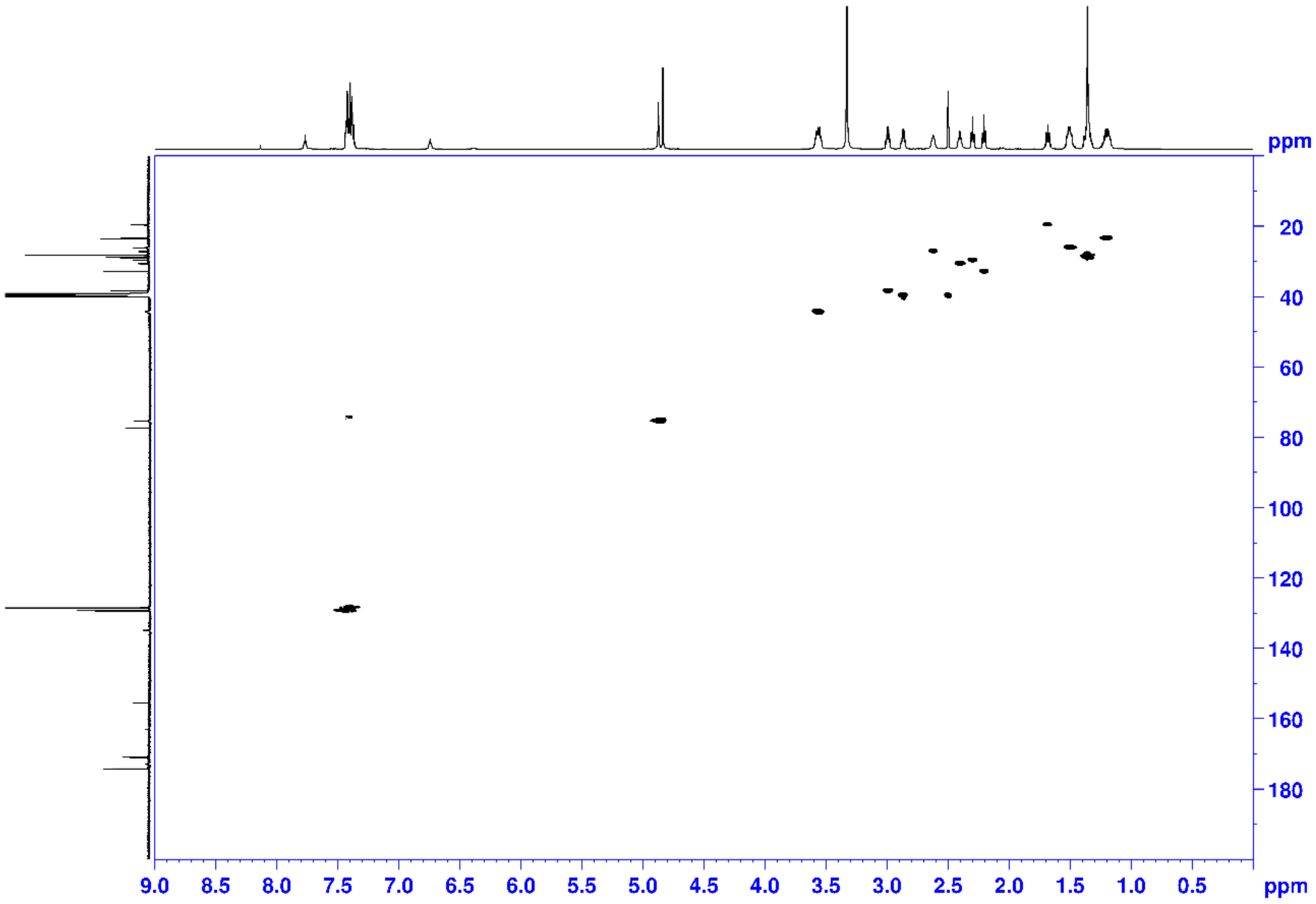

Figure S74. HSQC NMR (600 MHz, DMSO-d_6) spectrum of **S12**

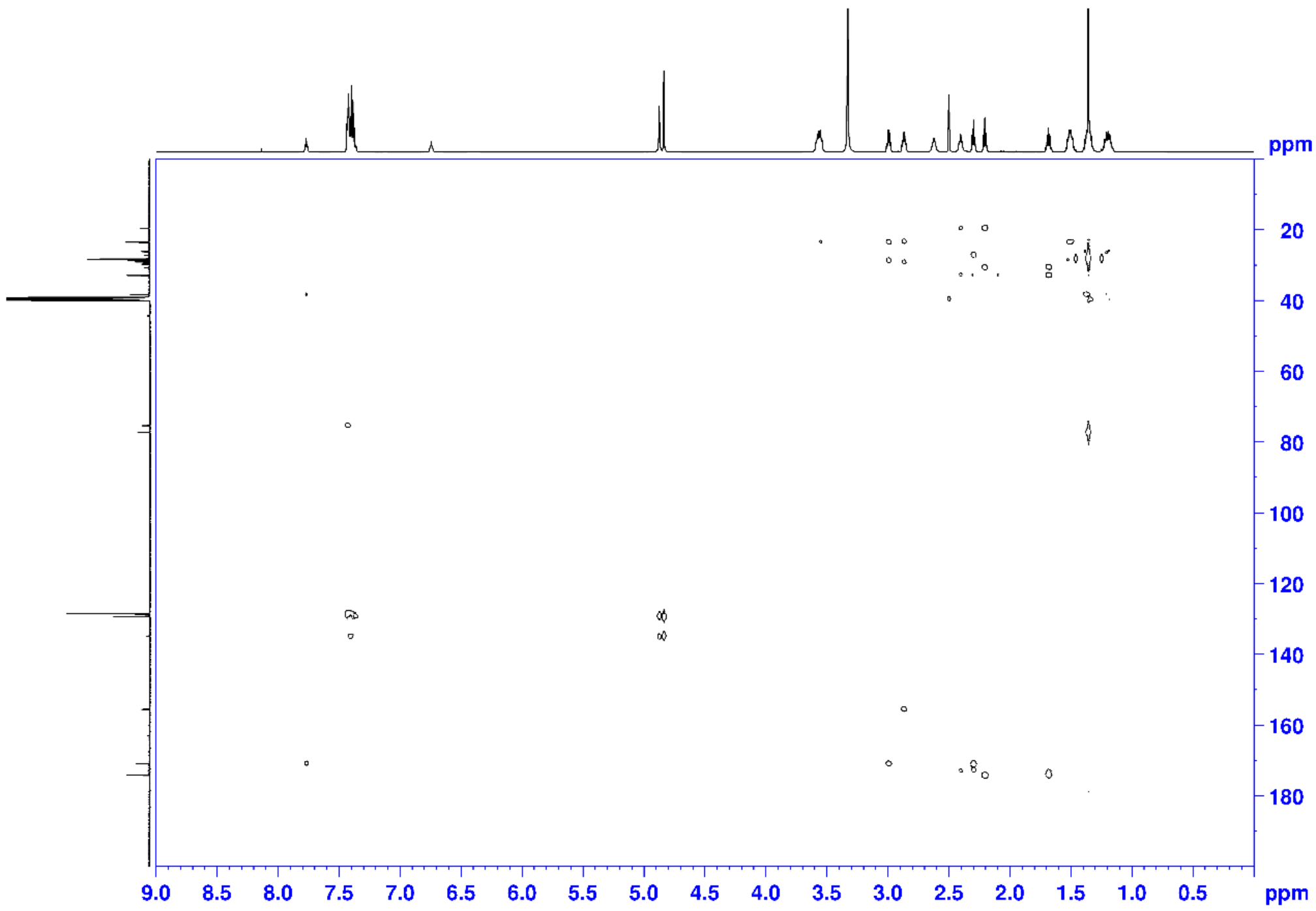

Figure S75. HMBC NMR (600 MHz, DMSO-d_6) spectrum of **S12**

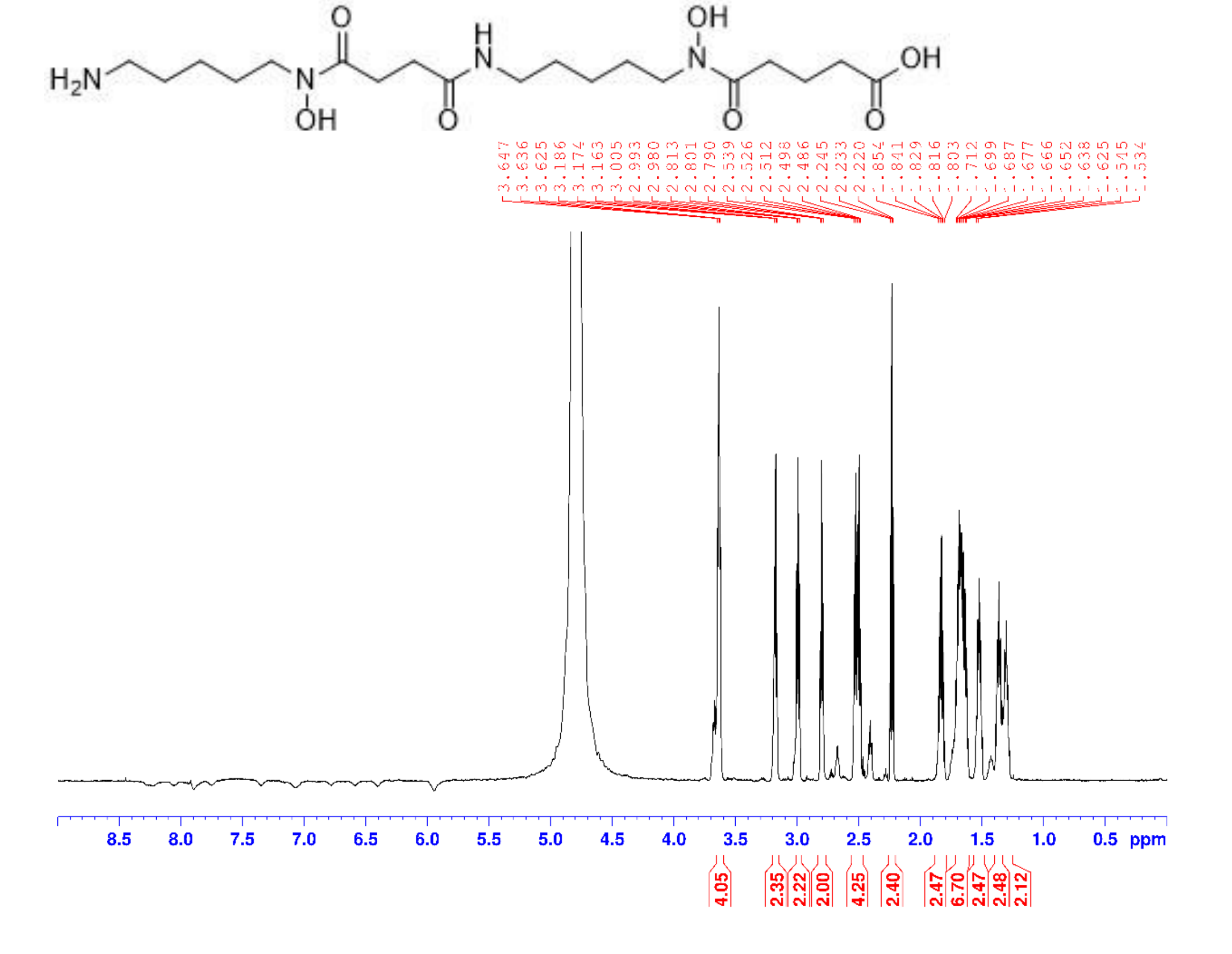

Figure S76. ^{1}H NMR (600 MHz, D_2O) spectrum of **6**

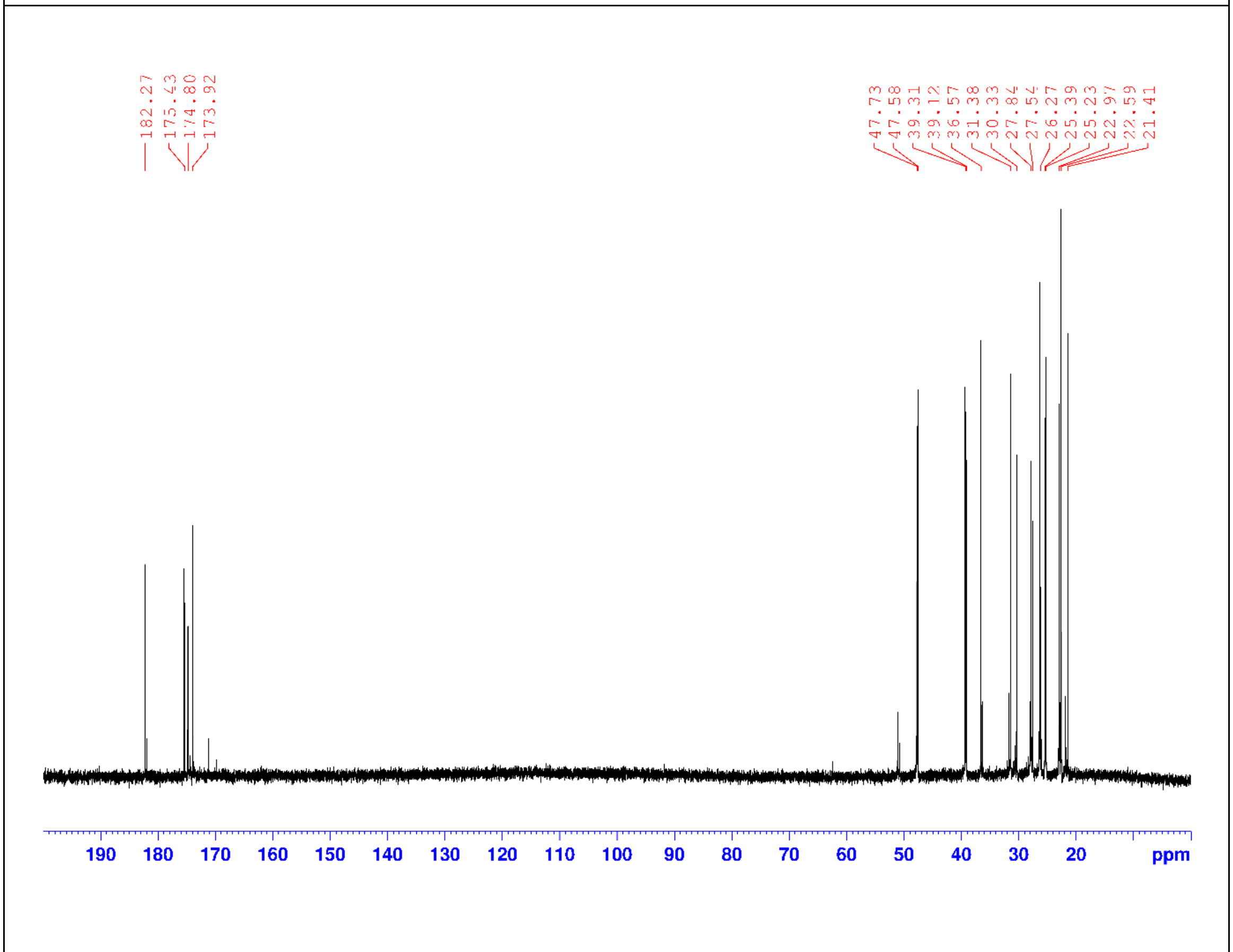

Figure S77. ^{13}C NMR (150 MHz, D_2O) spectrum of **6**

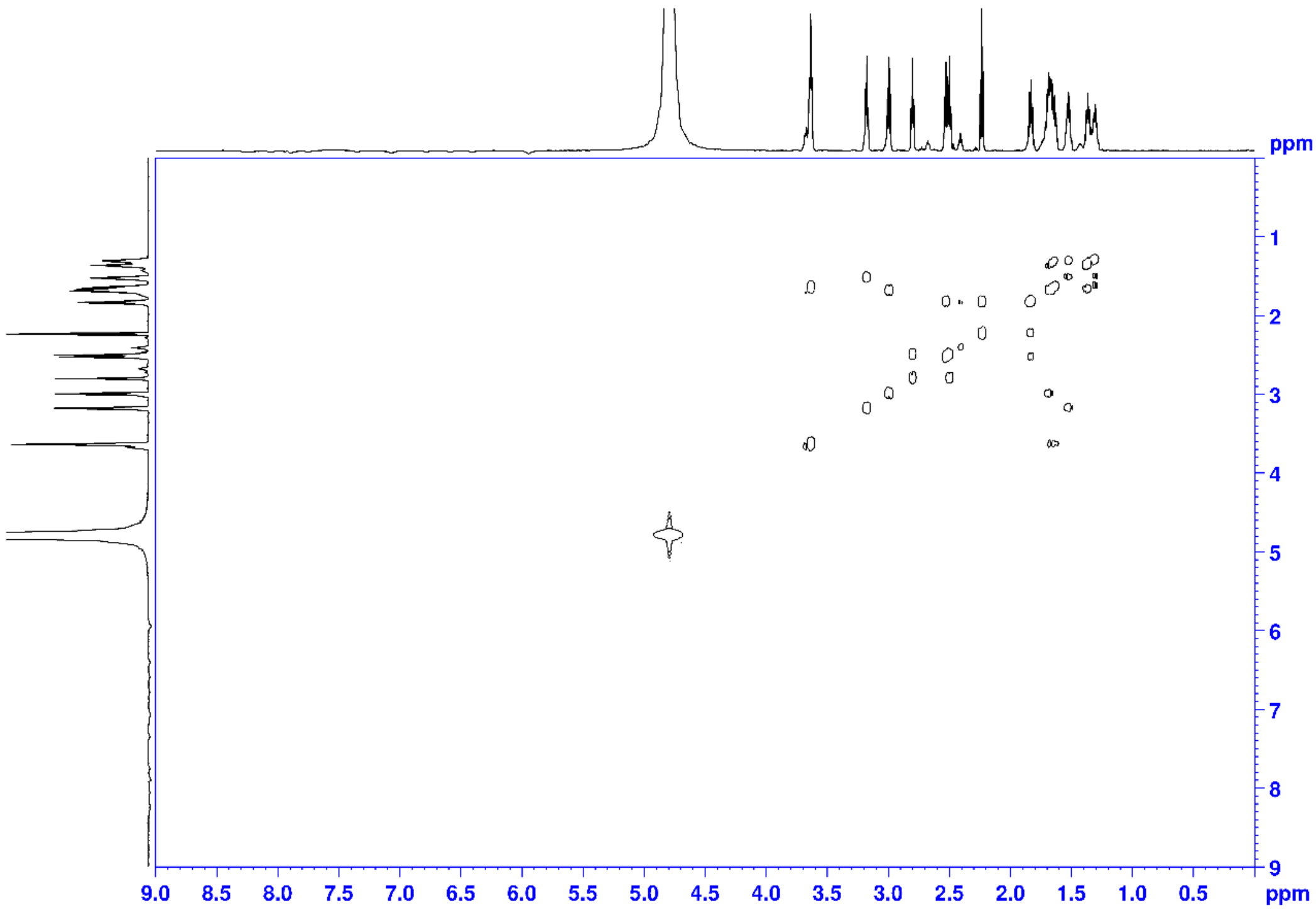

Figure S78. COSY NMR (600 MHz, D_2O) spectrum of **6**

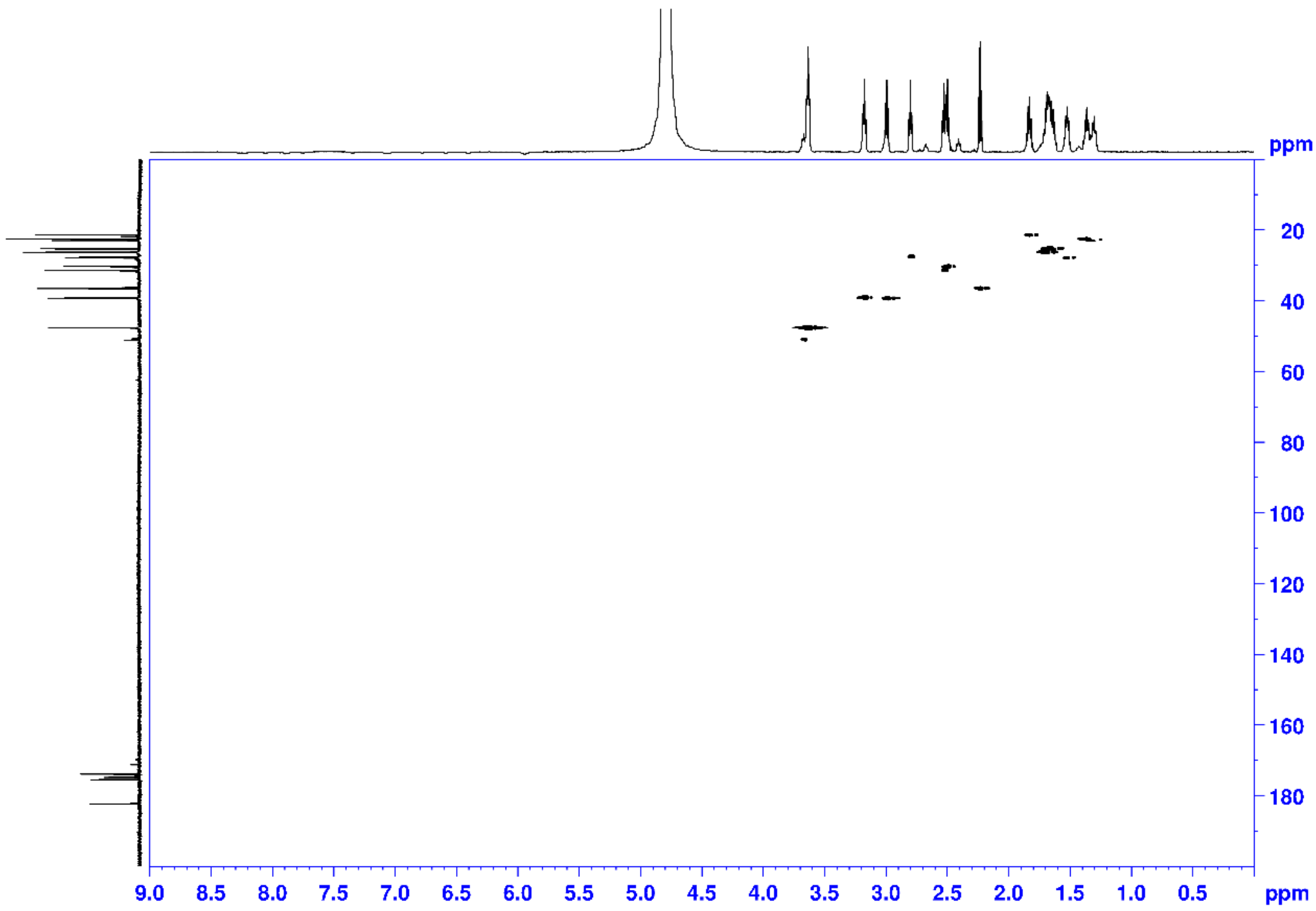

Figure S79. HSQC NMR (600 MHz, D_2O) spectrum of **6**

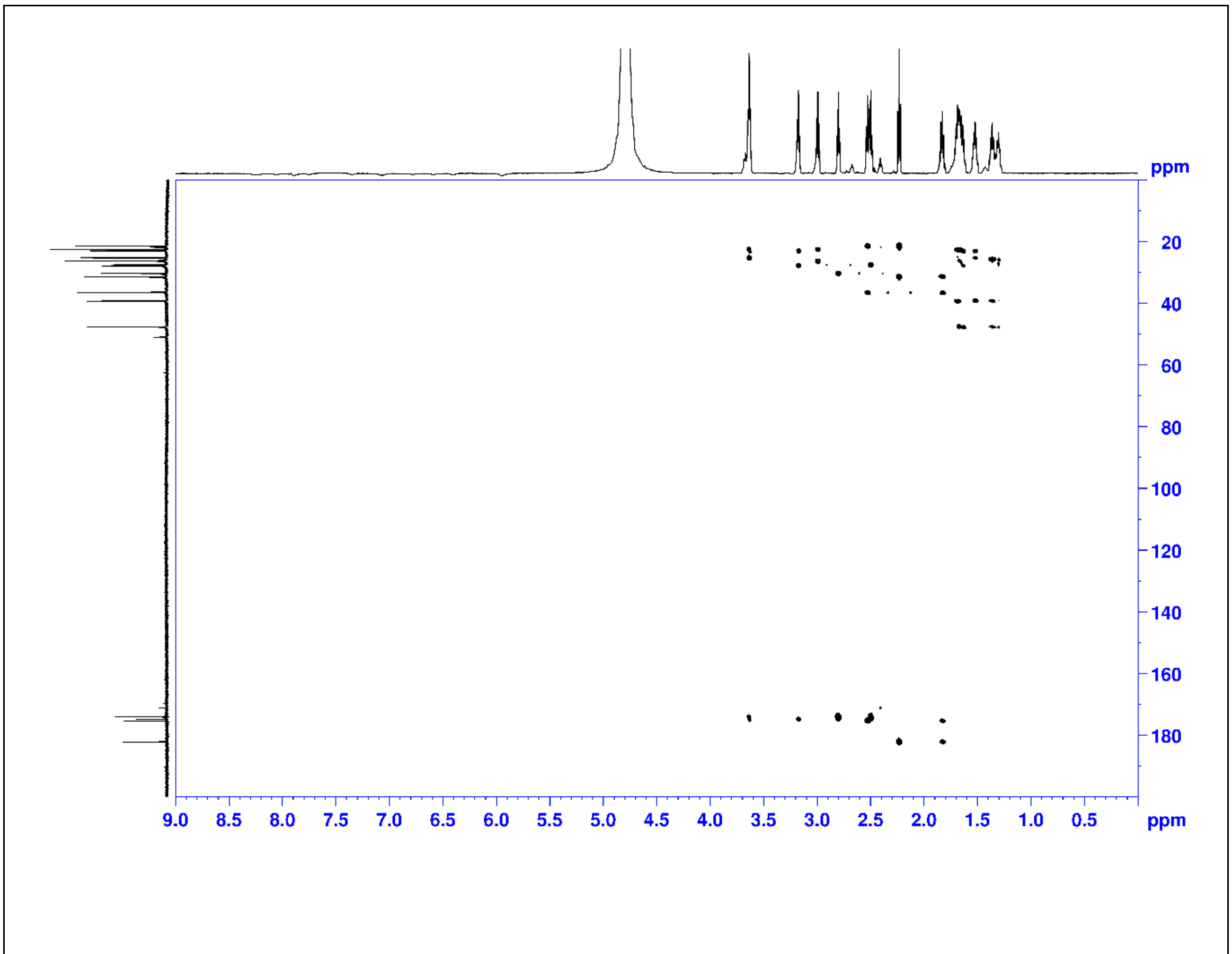

Figure S80. HMBC NMR (600 MHz, D_2O) spectrum of **6**

References

[1] K. P. Nolan, J. Font, A. Sresutharsan, M. P. Gotsbacher, C. J. M. Brown, R. M. Ryan, R. Codd, Acetyl-CoA-Mediated Post-Biosynthetic Modification of Desferrioxamine B Generates N- and N-O-Acetylated Isomers Controlled by a pH Switch. *ACS. Chem. Biol.* **2022**, *17*, 426-437.

[2] N. Kadi, D. Oves-Costales, F. Barona-Gomez, G. L. Challis, A new family of ATP-dependent oligomerization-macrocyclization biocatalysts. *Nat. Chem. Biol.* **2007**, *3*, 652-656.

[3] S. Rütschlin, T. Böttcher, Dissecting the Mechanism of Oligomerization and Macrocyclization Reactions of NRPS-Independent Siderophore Synthetases. *Chem. Eur. J.* **2018**, *24*, 16044-16051.

[4] T. E. Markham, R. Codd, A Mild and Modular Approach to the Total Synthesis of Desferrioxamine B. *J. Org. Chem.* **2024**, *89*, 5118-5125.

[5] C. J. Brown, M. P. Gotsbacher, J. P. Holland, R. Codd, endo-Hydroxamic acid monomers for the assembly of a suite of non-native dimeric macrocyclic siderophores using metal-templated synthesis. *Inorg. Chem.* **2019**, *58*, 13591-13603.

[6] K. P. Nolan, C. A. Rosser, J. L. Wood, J. Font, A. Sresuthasan, J. Wang, T. E. Markham, R. M. Ryan, R. Codd, An elastic siderophore synthetase and rubbery substrates assemble multimeric linear and macrocyclic hydroxamic acid metal chelators. *Chem. Sci.* **2025**, *16*, 2180-2190.

[7] T. E. Markham, A. Sresutharsan, C. A. Rosser, R. Codd, Directing the chemoenzymatic assembly of desferrioxamine B as a single product using N-tert-butoxycarbonyl-protected substrates. *Org. Biomol. Chem.* **2025**, *23*, 7181-7187.
